# The EduNutriCRC Questionnaire: A Pilot Knowledge–Attitudes–Practices Study on Nutritional Prevention of Colorectal Cancer in Romanian Adults

**DOI:** 10.3390/nu18142293

**Published:** 2026-07-13

**Authors:** Andreea-Adriana Neamțu, Alina Anton, Laura Maghiar, Andrada Iftode, Anca-Maria Căpraru, Cristina Dumitrescu, Andreea-Mihaela Kis, Ramona Amina Popovici, Cristina-Adriana Dehelean, Teodor-Andrei Maghiar

**Affiliations:** 1 Department of Toxicology, “Victor Babeș” University of Medicine and Pharmacy, Eftimie Murgu Square, No. 2, 300041 Timișoara, Romania; andreea.neamtu@umft.ro (A.-A.N.); dolghi.alina@umft.ro (A.A.); cristina.grosu@umft.ro (C.D.); cadehelean@umft.ro (C.-A.D.); 2 Research Centre for Pharmaco-Toxicological Evaluation, “Victor Babeș” University of Medicine and Pharmacy, Eftimie Murgu Square, No. 2, 300041 Timișoara, Romania; 3 Department of Pathology, Clinical County Emergency Hospital of Arad, Andrenyi Karoly Str., No. 2–4, 310037 Arad, Romania; 4 Department of Pathology, “Pius Brinzeu” Clinical County Emergency Hospital Timișoara, Liviu Rebreanu Boulevard, No. 156, 300723 Timișoara, Romania; 5 Department of Psycho-Neurosciences and Rehabilitation, Faculty of Medicine and Pharmacy, University of Oradea, Universității Str., No. 1, 410087 Oradea, Romania; 6 Department of Dermatovenerology, Clinical County Emergency Hospital Bihor, 410169 Oradea, Romania; 7 Doctoral School, University of Medicine and Pharmacy of Craiova, 200349 Craiova, Romania; anca.mitran@umfcv.ro; 8 Department of Pharmacy, Poiana Mare Psychiatry Hospital, Gării Str., No. 40, 207470 Poiana Mare, Romania; 9 Department of Management and Communication in Dental Medicine, “Victor Babeș” University of Medicine and Pharmacy, Eftimie Murgu Square, No. 2, 300041 Timișoara, Romania; kis.andreea@umft.ro (A.-M.K.); ramona.popovici@umft.ro (R.A.P.); 10 Department of Surgery, Faculty of Medicine and Pharmacy, University of Oradea, Universității Str., No. 1, 410087 Oradea, Romania; teodor.maghiar@yahoo.com; 11 Department of Surgery, Pelican Hospital, Corneliu Coposu Str., No. 2, 410450 Oradea, Romania

**Keywords:** colorectal cancer, nutritional prevention, knowledge–attitudes–practices (KAP), questionnaire, nutrition education, dietary risk factors, cancer prevention, health literacy, pilot validation study

## Abstract

Background/Objectives: Colorectal cancer (CRC) disproportionately affects Romania, where mortality exceeds the EU average and screening uptake remains low. Diet is among the most modifiable CRC risk factors, yet no validated instrument for assessing nutritional knowledge, attitudes, and practices (KAP) related to CRC prevention exists for the Romanian population. We developed and pilot-tested the EduNutriCRC questionnaire and characterised the corresponding nutritional KAP profile of Romanian adults. Methods: In a cross-sectional pilot study (April–May 2026), a convenience sample of 301 Romanian adults aged 18–74 years completed the 51-item self-administered questionnaire. The instrument was evaluated for internal consistency and dimensional structure (exploratory factor analysis), with non-parametric tests used for group comparisons. Results: The composite knowledge score (C1–C9; maximum 9) was 6.12 ± 2.25 with wide item-level variation. Internal consistency was acceptable, and the Attitudes subscale resolved into two factors (Motivation & Self-Efficacy and Perceived Barriers). Screening engagement was critically low (92.4% never tested; 81.7% unaware of the national programme). Although 64.1% expressed willingness to change their diet, 46.2% reported consuming processed meat at least weekly. Motivation & Self-Efficacy, but not the knowledge score, was inversely correlated with risk-food consumption (ρ = −0.39, *p* < 0.001). Conclusions: EduNutriCRC demonstrated acceptable psychometric properties at the pilot stage. The study identified prevalent nutritional misconceptions, a marked knowledge–behaviour gap, low screening awareness, and a structural mismatch between information sources used and those trusted. Attitudes and self-efficacy, rather than factual knowledge, were the more proximal correlates of dietary behaviour, supporting the development of self-efficacy-focused, culturally adapted educational interventions for CRC prevention in Romania, with EduNutriCRC serving as a baseline and monitoring instrument.

## 1. Introduction

Colorectal cancer (CRC) remains one of the heaviest oncological burdens worldwide. According to GLOBOCAN 2022, CRC ranked third in incidence and second in cancer-related mortality, with 1,926,425 new cases and 904,019 deaths globally—close to one in ten of all incident cancers [1,2]. Under unchanged incidence trajectories, the global burden is projected to reach 2.36 million new cases annually by 2050, with the steepest increases anticipated in countries undergoing rapid dietary and lifestyle transition [2]. Despite advances in screening, molecular characterisation, and targeted therapy, age-standardised incidence has not declined uniformly across regions, and substantial East–West disparities persist within Europe in both incidence and mortality [3].

Romania exemplifies the unfavourable end of this gradient. The 2025 OECD/European Commission country profile reports CRC mortality approximately 35% higher in men and 13% higher in women than the EU average, with an incidence trajectory rising more steeply than in Western European countries and avoidable-mortality gaps between Romania and the EU widening over the past decade [3,4]. Particularly concerning is the marked global increase in early-onset CRC (below 50 years of age), a trend now consistently documented across high- and middle-income countries and strongly associated with shifts in dietary patterns, ultra-processed food consumption, obesity, and lifestyle changes [5,6,7,8,9]. Romanian hospital-based evidence reinforces this profile: in a five-year retrospective cohort study from western Romania, emergency presentation accounted for up to 83.95% of new CRC admissions during the early pandemic period, with patients frequently presenting at advanced stage and with complications such as peritumoral adhesion, occlusion, and perforation [10]. These patterns reflect persistent structural barriers—late-stage presentation at diagnosis, fragmented preventive services, and limited public awareness of modifiable risk factors [4,10]. Romania introduced its first regional CRC screening programme (ROCCAS) only in 2020, with preliminary indicators meeting EU recommendations but with colonoscopy acceptance and overall population coverage still well below those of Western European peers [11,12]. The Romanian National Cancer Prevention and Control Plan 2023–2030 sets targets for earlier detection and improved health literacy [13], yet the extent to which these targets translate into structured, evidence-informed nutritional education programmes has not been demonstrated [14].

The role of diet in colorectal carcinogenesis is supported by a converging and increasingly mechanistic body of evidence. A recent comprehensive meta-analysis confirmed dose-dependent associations between red and processed meat consumption and CRC risk, with consistent effects across colon and rectal subsites [15]. The principal carcinogenic pathways include the formation of heterocyclic amines and polycyclic aromatic hydrocarbons during high-temperature cooking, endogenous generation of *N*-nitroso compounds catalysed by haem-iron, and lipid peroxidation in the colonic mucosa, producing DNA adducts and chronic low-grade inflammation [16,17]. Conversely, dietary fibre, fruits, vegetables, legumes, and whole grains exert protective effects: pooled data demonstrate a ~10% reduction in CRC risk per 10 g/day increment in dietary fibre, mediated by accelerated bowel transit, dilution of luminal carcinogens, and the production of short-chain fatty acids (SCFAs)—most notably butyrate—by colonic microbial fermentation [18]. Recent meta-analyses confirm robust inverse associations between adherence to Mediterranean-style dietary patterns and CRC incidence, with pooled risk reductions of 5–16% across diverse populations [19,20]. Butyrate, the principal SCFA produced from fibre fermentation, exerts well-characterised antineoplastic effects through histone deacetylase inhibition, induction of apoptosis and cell-cycle arrest in transformed colonocytes, and reinforcement of mucosal barrier integrity [21,22]. Ultra-processed food intake, by contrast, has been independently associated with increased risk of early-onset colorectal adenomas and CRC, plausibly through microbiota disruption, additive exposure, and obesogenic effects [8,9].

The gut microbiota has emerged as a central effector linking diet, host metabolism, and CRC risk. Dysbiosis profiles enriched for *Fusobacterium nucleatum*, enterotoxigenic *Bacteroides fragilis*, and colibactin-producing *Escherichia coli* increase epithelial permeability, amplify pro-inflammatory signalling, and generate genotoxic metabolites that contribute to tumorigenesis [23,24,25]. Diet, microbiome, and colorectal carcinogenesis are therefore tightly interrelated, and the population-level adoption of protective dietary patterns represents one of the most cost-effective preventive levers in oncology [20,26].

On this basis, the World Health Organization, the World Cancer Research Fund, the International Agency for Research on Cancer, and the European Commission have all positioned nutritional education as a central pillar of cancer prevention [26]. The European Code Against Cancer (5th edition, 2025) and Europe’s Beating Cancer Plan (2021) jointly call for healthier dietary behaviours at the population level, reduced exposure to avoidable risk factors, and improved cancer-related health literacy [27,28]. Translating these recommendations into effective, context-adapted interventions, however, requires reliable instruments to assess the baseline knowledge, attitudes, and self-reported practices (KAP) of the target population, instruments that are valid in the local cultural and food context, not merely translated from elsewhere [29].

International KAP studies on CRC and nutrition have consistently reported moderate to low recognition of dietary risk factors, persistent misconceptions concerning the role of red and processed meat, fibre, and alcohol, and a substantial gap between declared knowledge and actual dietary practice. In a recent Saudi study (*n* = 1040), 77.8% of adults displayed low knowledge of dietary and lifestyle-related CRC risk factors [30]; comparable patterns of low knowledge, negative attitudes, and poor practices have been documented in Jordanian university students [31] and in southern Italian adults [29]. In the Romanian context specifically, per-capita red and processed meat consumption is among the highest in the European Union, and previous work by our group has demonstrated that a CRC-preventive dietary model emphasising red meat reduction and increased fibre and micronutrient intake is broadly acceptable to Romanian adults aged 18–50, though approximately 62% of respondents reported difficulty in reducing red meat intake [14]. General-population studies further indicate that Romanian adults exhibit moderate nutrition knowledge with marked socioeconomic gradients and low food literacy in disadvantaged groups [32,33] and that no validated tool currently exists for nutritional CRC prevention specifically. Existing international KAP instruments are anchored in cultural and food contexts that differ substantially from those of Eastern Europe, limiting their direct transferability. This dual gap—the absence of a validated instrument and the absence of population-level KAP data on dietary CRC prevention in Romania—limits both the design of evidence-informed educational interventions and the monitoring of behavioural change at the population level.

To address this gap, we developed and piloted the EduNutriCRC questionnaire, a structured 51-item KAP instrument designed specifically for the assessment of nutritional knowledge, attitudes, and self-reported practices relevant to CRC prevention in Romanian adults. We hypothesised that nutritional knowledge would be heterogeneous across sociodemographic strata, that key misconceptions—particularly concerning red and processed meat, fibre, alcohol, and ultra-processed foods—would be prevalent, and that a substantial knowledge–behaviour gap would be observed. The aims of the present study were therefore threefold: (i) to develop the EduNutriCRC instrument following established methodological standards for scale construction in health sciences [34] and to evaluate its psychometric properties in a cross-sectional pilot sample of Romanian adults; (ii) to characterise the nutritional knowledge, attitudes, and self-reported dietary practices related to CRC prevention in that sample and to identify the most prevalent misconceptions and behavioural gaps across sociodemographic strata; and (iii) to generate baseline data that can inform the design and evaluation of larger educational interventions targeting dietary cancer prevention in Romania, in alignment with the objectives of the National Cancer Prevention and Control Plan 2023–2030 and as a next step in the research line initiated by the diet-acceptability study of our group [14].

## 2. Materials and Methods

### 2.1. Study Design and Setting

We conducted a cross-sectional observational pilot study aimed at the development and preliminary psychometric evaluation of a structured knowledge, attitudes, and practices (KAP) questionnaire on nutritional prevention of colorectal cancer (EduNutriCRC) and at characterising the corresponding KAP profile of Romanian adults (see Supplementary Materials Table S1 for anonymised individual-level response data for the pilot EduNutriCRC questionnaire (*n* = 301), and Table S2 for the full text of the EduNutriCRC questionnaire—items, response formats, and response options). Data collection was carried out between 20 April and 20 May 2026 via a self-administered online questionnaire. The study was developed within the academic framework of “Victor Babeș” University of Medicine and Pharmacy, Timișoara, Romania, as part of a research line on nutritional education, preventive oncology, and population-level health promotion and constitutes a methodological extension of our previously published work on the acceptability of a CRC-preventive dietary model in Romanian adults [14].

### 2.2. Development of the EduNutriCRC Questionnaire

#### 2.2.1. Item Generation

Item generation followed the framework proposed by Boateng et al. for scale development in health and behavioural research [34], proceeding in three stages:

(i) identification of the relevant theoretical domain,
(ii) generation of an initial item pool, and
(iii) content evaluation.

The theoretical domain was defined through a structured narrative review of the current literature on CRC epidemiology, dietary risk and protective factors, and preventive oncology, supplemented by the principal international guidance documents—the WCRF/AICR Third Expert Report [26], the European Code Against Cancer 5th edition [27], and Europe’s Beating Cancer Plan [28]. To capture not only factual knowledge but also affective and behavioural dimensions of dietary change, the conceptual framework integrated four behavioural-science models: the Health Belief Model [35], the Theory of Planned Behaviour [36], Social Cognitive Theory [37], and the Transtheoretical Model of Behaviour Change [38].

Candidate items were generated by the research team and grouped under seven a priori content domains:

- sociodemographic and anthropometric characteristics;
- baseline awareness of CRC and screening;
- knowledge of CRC nutritional risk and protective factors;
- attitudes toward preventive eating and barriers to dietary change;
- self-reported dietary habits and food frequency;
- information sources and trust; and
- familiarity with national and European cancer prevention policies.

Three response formats were used: multiple-choice items, five-point Likert scales (1 = strongly disagree to 5 = strongly agree), and food-frequency items with six ordered categories ranging from “never or almost never” to “daily.”

#### 2.2.2. Content Validation

Content validity was assessed by a multi-disciplinary expert team drawn from fundamental and applied toxicology research, clinical-oncology-related specialities, nutrition and dietetics, and public health. The experts independently reviewed each item for relevance, clarity, and representativeness of the target construct, and the questionnaire was iteratively revised by consensus until full agreement was reached on the final item pool [14,34]. To ensure adequate content coverage, every candidate item was additionally mapped against both the seven a priori content domains and the four behavioural-science frameworks, so that the knowledge, attitudinal, and practice constructs were each explicitly represented, and items judged redundant, ambiguous, or insufficiently relevant were reworded or removed during the consensus rounds. Together with the subsequent pilot examination of item difficulty, corrected item-total correlations, internal consistency, and dimensional structure (Section 2.5.4), this procedure provided layered evidence of content, internal-structure, and reliability validity appropriate to a pilot-stage instrument, whereas criterion and predictive validity against objective dietary or screening outcomes remain to be established.

#### 2.2.3. Final Instrument

The final EduNutriCRC questionnaire comprises 51 items distributed across the seven content domains described above. The instrument was implemented in Romanian on the Google Forms platform and organised into nine sequential sections, preceded and ended by a mandatory electronic informed-consent screen. Estimated completion time was 10–12 min.

### 2.3. Participants and Recruitment

Eligible participants were adults aged 18 to 74 years, living in Romania, fluent in Romanian, with access to a digital device and an internet connection, and able to provide electronic informed consent. The upper age limit of 74 years was set to align with the target age range of the European Union population-based colorectal cancer screening recommendations and of the Romanian National Cancer Screening Programme (ROCCAS), for which primary-prevention educational interventions are most directly actionable [11]. Individuals who were unable to complete the questionnaire independently or who declined to provide informed consent were excluded. Recruitment used convenience sampling. The study link was disseminated through personal and professional networks of the research team, including dedicated Facebook groups focused on health, nutrition, and preventive medicine, as well as WhatsApp groups and contacts. Participation was voluntary, unpaid, and fully anonymous. Each Google account could submit the questionnaire only once, which restricted accidental duplicate submissions while preserving respondent anonymity.

The minimum sample size for the present pilot phase was determined according to two complementary criteria. First, following Boateng et al. [34], a ratio of five respondents per scale item was applied (51 items × 5 = 255 participants) as the conventional lower bound for exploratory psychometric analysis of a newly developed instrument. Second, for descriptive estimation of population-level proportions in the Romanian adult population, Cochran’s formula with a finite-population correction yields a minimum of 384 participants at a 95% confidence level and a ±5% margin of error, assuming maximum response variability (*p* = 0.5) [14]. Because the present study was designed as a pilot focused on instrument development and preliminary characterisation of the KAP profile rather than as a definitive prevalence survey, the lower threshold of 255 was adopted as the binding target; the higher 384-participant benchmark was acknowledged a priori as the threshold to be addressed in a subsequent confirmatory study with stratified probability sampling.

**Figure 1:**
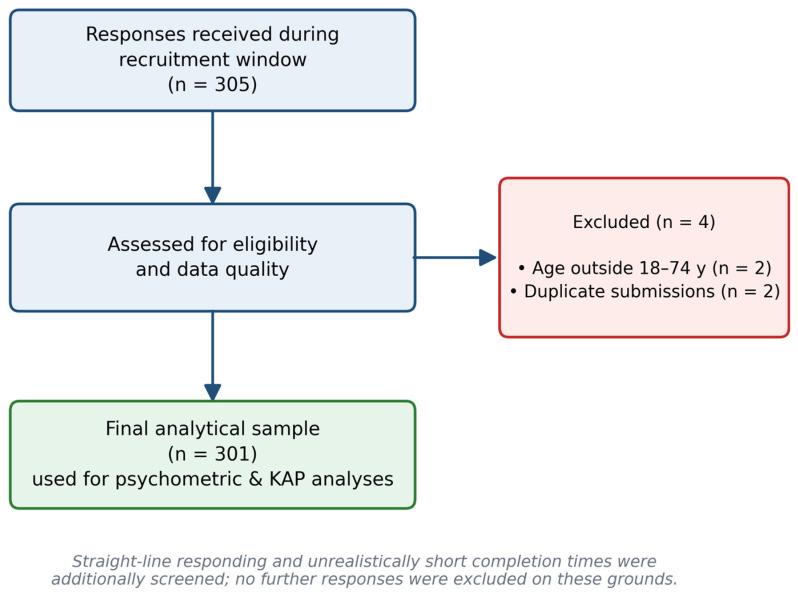
Recruitment and exclusion process for the pilot EduNutriCRC Questionnaire.

A total of 305 responses were received during the recruitment window (20 April and 20 May 2026). Four responses were excluded prior to analysis: two respondents outside the eligible age range (one below 18 years and one above 74 years) and two duplicate submissions identified by exact record matching (in both cases, two consecutive entries from the same respondent submitted within seconds of each other). Submissions were additionally screened for straight-line responding across the food-frequency section and for unrealistically short completion times; no further responses were excluded on these grounds. The final analytical sample comprised *n* = 301 participants. The recruitment and exclusion process is summarised in Figure 1.

### 2.4. Ethical Considerations

The study was reviewed and approved by the Ethics Committee of Pelican Hospital, Oradea, Romania (approval number 269, dated 18 February 2026). The study was conducted in accordance with the principles of the Declaration of Helsinki and complied with the General Data Protection Regulation (Regulation EU 2016/679). No personally identifiable information (name, contact details, IP address, or geolocation) was collected at any point. Prior to accessing the questionnaire, all participants were presented with an electronic informed-consent screen detailing the voluntary nature of participation, the right to withdraw at any time without justification, the anonymous handling of data, and the exclusive use of responses for scientific research and publication. Submission of the questionnaire was conditional on repeating the explicit electronic consent.

### 2.5. Statistical Analysis

Statistical analyses were conducted using R Studio version 2025.05.0+496 (Posit, Boston, MA, USA) with the necessary packages, GraphPad Prism version 10 (GraphPad Software, San Diego, CA, USA), and Microsoft Excel (Microsoft 365, Microsoft Corp., Redmond, WA, USA) for data cleaning and preliminary tabulation.

#### 2.5.1. Descriptive Statistics

Categorical variables are reported as frequencies and percentages; continuous variables are reported as mean ± standard deviation (SD) when normally distributed and as median and interquartile range (IQR) otherwise. For composite scores derived from multi-item subscales (Nutritional Knowledge, Motivation & Self-Efficacy, Perceived Barriers), both summary statistics are reported to facilitate comparison with other KAP instruments. Normality of continuous variables was assessed using the Shapiro–Wilk test, complemented by visual inspection of histograms and Q–Q plots.

#### 2.5.2. Knowledge Scoring

A composite nutritional-knowledge score was computed from items C1–C9 of the Knowledge subscale (maximum 9 points). For each item, the response “True” was scored as correct (1 point); the responses “False” and “Don’t know” were scored as incorrect (0 points). Item C10 (“Carbonated water increases the risk of cancer”), which represents a common, scientifically unsupported public misconception rather than a guideline-derived risk or protective factor, was retained in the questionnaire as a misconception-detection item but excluded from the composite score; this decision was supported a posteriori by internal-consistency analysis (Section 3.2.1).

#### 2.5.3. Attitudes Scoring

For the Attitudes subscale (items D1–D7), responses were coded on a five-point Likert scale (1 = strongly disagree to 5 = strongly agree). Items D5, D6, and D7 reflect perceived barriers to dietary change (contradictory dietary information, cost, and time, respectively) and were retained on the original direction; the dimensional structure of the subscale was examined empirically rather than assumed (Section 2.5.4).

#### 2.5.4. Psychometric Evaluation

Internal consistency of the Knowledge and Attitudes subscales was assessed using Cronbach’s α coefficient (Kuder–Richardson 20 equivalent for the binary-scored knowledge items), with values ≥ 0.70 considered acceptable for research instruments and ≥0.60 acceptable for short scales at the pilot stage [34]. For each subscale, corrected item-total correlations were computed; items showing values <0.20 were flagged for review. For the Knowledge subscale, item difficulty was calculated as the proportion of respondents answering correctly. In response to the ordinal measurement level of the items, internal-consistency reliability was additionally estimated as ordinal α, computed from the polychoric correlation matrix for the Likert-type attitudes items and from the tetrachoric correlation matrix for the dichotomously scored knowledge items, as recommended for categorical indicators [39].

The dimensional structure of the Attitudes subscale was examined by exploratory factor analysis. The suitability of the data for factor analysis was confirmed by the Kaiser–Meyer–Olkin (KMO) measure of sampling adequacy (acceptable threshold ≥ 0.60) and Bartlett’s test of sphericity (significant at *p* < 0.05). The number of factors to retain was determined by the Kaiser criterion (eigenvalues > 1) corroborated by inspection of the scree plot. Factor extraction was performed by principal-axis factoring, with varimax rotation applied to facilitate interpretation of the resulting factor structure. Loadings ≥ 0.40 were considered salient.

#### 2.5.5. Inferential Statistics

All composite scores deviated from normality on Shapiro–Wilk testing (all *p* < 0.001); non-parametric tests were therefore used for inferential comparisons. Associations between knowledge, attitudes, and practices scores and sociodemographic variables (age group, sex, educational attainment, urban/rural residence, self-reported household income, and first-degree family history of CRC) were tested as follows: for comparisons between two independent groups, the Mann–Whitney U test was used; for comparisons across three or more groups, the Kruskal–Wallis test was used, with Dunn’s post hoc test and Bonferroni correction for multiple comparisons applied whenever the omnibus test reached statistical significance. Associations between categorical variables were tested using the χ^2^ test or Fisher’s exact test when expected cell counts were below 5. Correlations between continuous and ordinal variables were assessed with Spearman’s rank-order correlation coefficient. Where one variable was binary and the other ordinal (e.g., awareness of a policy framework across ordered educational strata), monotonic association was tested with Spearman’s rank-order correlation as a test for ordered trend, in addition to the omnibus χ^2^ test. All tests were two-tailed, with statistical significance set at *p* < 0.05.

#### 2.5.6. Mediation Analysis of the KAP Framework

To formally evaluate the sequential knowledge → attitudes → practices assumption that underlies the KAP paradigm, a simple mediation model was specified with the composite nutritional-knowledge score (C1–C9) as the independent variable, the Motivation & Self-Efficacy subscale (D1–D4) as the hypothesised mediator, and the frequency of risk-food consumption as the outcome; a parallel model substituted protective-food intake as the outcome. Standardised path coefficients were estimated by ordinary least-squares regression for the a path (knowledge → attitudes), the b path (attitudes → practice, adjusted for knowledge), the direct effect c′ (knowledge → practice, adjusted for attitudes), and the total effect c (knowledge → practice). The indirect (mediated) effect (a × b) was evaluated with a bias-corrected, accelerated non-parametric bootstrap (10,000 resamples), used in preference to the normal-theory Sobel test [40] because tests based on the product of coefficients can be imprecise [41]; the indirect effect was considered statistically significant when its bias-corrected bootstrap 95% confidence interval excluded zero. Given the cross-sectional design, these estimates were interpreted as a statistical decomposition of association rather than as evidence of a causal mechanism [42,43]. Because the attitudes construct comprised two distinct factors, both the Motivation & Self-Efficacy and the Perceived Barriers subscales were examined as candidate mediators, and inference followed the joint-significance criterion, under which a mediated effect requires both the a path and the b path to reach statistical significance [43].

### 2.6. Use of Generative Artificial Intelligence (GenAI)

During the preparation of this manuscript, the authors used ChatGPT (OpenAI, Thinking, 1.2026.118 version) for image generation, Claude (Anthropic, Opus 4.7 Adaptive, 1.8555.2 (a476c3) version) for language refinement and structural review of the manuscript draft, and Gemini (Google, 3.5 Flash, 1.60.2.323 version) for language refinement assistance. The authors have reviewed and edited all output and take full responsibility for the content of this publication.

## 3. Results

### 3.1. Sample Characteristics

Prior to inferential analyses, normality of all continuous and composite scores was screened with the Shapiro–Wilk test, supplemented by histogram and Q–Q plot inspection. The composite knowledge score (W = 0.925, *p* < 0.001), the Motivation & Self-Efficacy subscale (W = 0.912, *p* < 0.001), and the Perceived Barriers subscale (W = 0.970, *p* < 0.001) all deviated significantly from normality; non-parametric tests were therefore used throughout the inferential analyses reported below.

Of 305 responses received during the recruitment window, four were excluded prior to analysis (two outside the eligible age range and two duplicate submissions), yielding a final analytical sample of *n* = 301 participants. The full sociodemographic profile of the sample is summarised in Table 1.

Participants were predominantly female (*n* = 241, 80.1%) and resided in urban settings (*n* = 249, 82.7%). Mean age was 34.8 ± 13.3 years (range 19–71), with the majority distributed across the two younger age strata (18–29 years, *n* = 117, 38.9%; 30–44 years, *n* = 116, 38.5%). Educational attainment was high: 201 participants (66.8%) reported a university-level degree or higher, while 82 (27.2%) had completed upper secondary or vocational education only. Among the 297 participants with valid anthropometric data, mean BMI was 24.4 ± 4.8 kg/m^2^, with 56.9% in the normal-weight range and 37.4% overweight or obese. Thirty-five respondents (11.6%) reported a first-degree family history of colorectal cancer.

Geographically, responses were concentrated in western Romania, with Bihor (*n* = 129, 42.9%), Timiș (*n* = 48, 15.9%), Dolj (*n* = 43, 14.3%), and Arad (*n* = 30, 10.0%) accounting for 83.1% of the sample. A further 17 counties were represented in smaller numbers, and the geographic concentration is acknowledged as a limitation of the convenience sampling approach.

### 3.2. Psychometric Properties of the EduNutriCRC Instrument

Before describing the substantive findings, the internal consistency and structural properties of the two principal multi-item subscales of the EduNutriCRC instrument, the Knowledge subscale (Section C) and the Attitudes subscale (Section D), were evaluated.

#### 3.2.1. Knowledge Subscale (C1–C9)

Cronbach’s α for the nine-item composite knowledge score (C1–C9) was 0.710, meeting the conventional 0.70 threshold for a research instrument. Inclusion of the misconception-detection item C10 (“Carbonated water increases the risk of cancer”) decreased α to 0.669, providing empirical support for the a priori decision to retain C10 in the questionnaire as a misconception probe but to exclude it from the composite score. All nine items in the composite showed corrected item-total correlations above the conventional 0.20 threshold (range 0.29–0.46), indicating that each contributed meaningfully to the overall scale. Item difficulty (proportion correctly answered) ranged from 44.2% for C9 (the role of moderate dairy intake) to 80.1% for C1 (processed meat as a risk factor), spanning a range appropriate for a knowledge instrument applied to a general-population sample. The ordinal α for the Knowledge subscale, computed from the tetrachoric correlation matrix, was 0.836, higher than the corresponding Pearson-based estimate—as expected when reliability is estimated from the tetrachoric rather than the Pearson correlation matrix—and comfortably above the 0.70 criterion [39].

#### 3.2.2. Attitudes Subscale (D1–D7)

Suitability of the attitudes items for factor analysis was strongly supported by both the Kaiser–Meyer–Olkin measure of sampling adequacy (KMO = 0.769, well above the 0.60 threshold) and Bartlett’s test of sphericity (χ^2^ = 958.81, *df* = 21, *p* < 0.001). Principal-axis factor extraction yielded two factors with eigenvalues above unity (3.29 and 1.53), jointly accounting for 68.7% of the total-item variance, with the scree-plot inflection point further supporting a two-factor solution.

After varimax rotation, the structure showed a clean simple-structure pattern (Table 2): items D1–D4 loaded strongly on the first factor (loadings 0.79–0.92) and weakly on the second (|loadings| ≤ 0.14), while items D5–D7 loaded strongly on the second factor (loadings 0.69–0.79) and weakly on the first (loadings ≤ 0.21). Substantively, the first factor was labelled Motivation & Self-Efficacy and grouped items expressing positive appraisal of one’s own diet, belief in the preventive potential of dietary change, willingness to make changes, and confidence in cooking healthy meals. The second factor was labelled Perceived Barriers and grouped items reflecting contradictory information, cost, and time as obstacles to healthy eating. Internal consistency was excellent for the Motivation & Self-Efficacy subscale (Cronbach’s α = 0.891) and acceptable for the three-item Perceived Barriers subscale (α = 0.652), the latter being a value commonly accepted for short scales at the pilot stage. These two subscales were retained as the analytical units for subsequent attitude-related comparisons. Ordinal α values derived from the polychoric correlation matrix were 0.913 for the Motivation & Self-Efficacy subscale and 0.688 for the Perceived Barriers subscale; both exceeded their Pearson-based counterparts and remained within their respective acceptable ranges [39].

### 3.3. Baseline Colorectal Cancer Awareness and Screening Engagement

**Figure 2:**
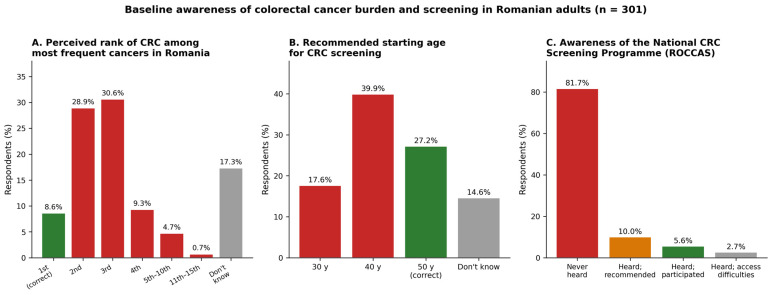
Baseline awareness of colorectal cancer epidemiology and screening in Romanian adults (*n* = 301). (**A**) Perceived rank of CRC among the most frequently diagnosed cancers in Romania; (**B**) perceived recommended starting age for population-based CRC screening; (**C**) self-reported awareness of and engagement with the National CRC Screening Programme (ROCCAS). Green bars indicate guideline-concordant responses (correct rank, 50 years as screening age, having participated), red bars indicate incorrect or non-engaged responses, the amber bar in panel C indicates respondents who had heard of the programme and had been recommended to participate, and grey bars indicate “Don’t know” responses.

Baseline awareness of the population-level burden of colorectal cancer in Romania was strikingly poor. When asked to identify the rank of CRC among the most frequently diagnosed cancers in Romania, only 26 of 301 participants (8.6%) selected the correct response (first place), despite CRC being the most commonly diagnosed cancer in Romania in 2022, accounting for 13,541 new cases (12.9% of all incident cancers) ahead of breast and lung cancer [1,10]. The majority of respondents underestimated the burden: 87 (28.9%) ranked CRC second, 92 (30.6%) ranked it third, 28 (9.3%) ranked it fourth, 14 (4.7%) placed it between fifth and tenth, and 2 (0.7%) between eleventh and fifteenth. A further 52 participants (17.3%) reported not knowing (Figure 2A).

Knowledge of the recommended screening start age was particularly poor. Only 82 participants (27.2%) correctly identified 50 years as the recommended starting age for population-based CRC screening, in line with both European Union recommendations and the Romanian ROCCAS programme. The most common response was 40 years (*n* = 120, 39.9%), suggesting a tendency to overestimate the urgency of early screening, while 53 participants (17.6%) selected 30 years and 44 (14.6%) reported not knowing (Figure 2B).

Recognition of CRC warning signs (multi-select item B3) was relatively strong for the three classical symptoms: 267 participants (88.7%) identified rectal bleeding, 234 (77.7%) identified unexplained weight loss, and 214 (71.1%) identified persistent changes in bowel transit. Conversely, 23 participants (7.6%) declared not knowing any warning sign, and a small but non-negligible proportion endorsed unrelated symptoms such as joint pain (*n* = 12, 4.0%) or hair loss (*n* = 20, 6.6%).

Awareness of the National Colorectal Cancer Screening Programme was low: 246 participants (81.7%) reported never having heard of it, 30 (10.0%) had been recommended to participate, 17 (5.6%) had participated, and 8 (2.7%) reported difficulty accessing the programme despite having heard of it (Figure 2C). Consistent with these awareness levels, only 23 participants (7.6%) reported ever having taken a CRC screening test (FIT, colonoscopy, or both), while 278 (92.4%) had not.

### 3.4. Nutritional Knowledge of Colorectal Cancer Risk and Protective Factors

#### 3.4.1. Item-Level Findings

**Figure 3:**
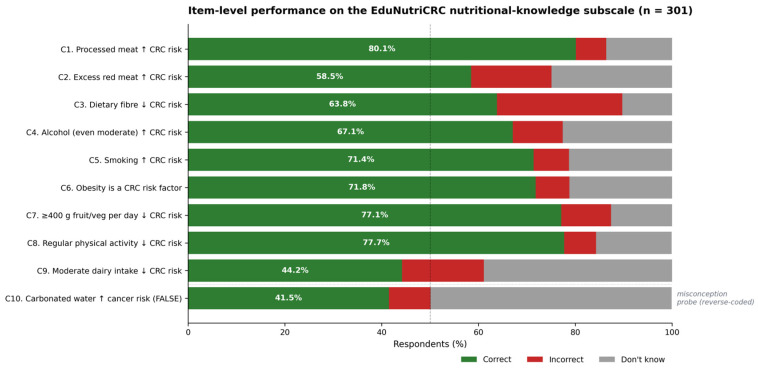
Item-level performance on the EduNutriCRC nutritional-knowledge subscale (*n* = 301). For each item, stacked bars show the percentage of respondents giving the correct response (green), an incorrect response (red), or “Don’t know” (grey). Items C1–C9 contribute to the composite knowledge score; the misconception-detection item C10 (“Carbonated water increases the risk of cancer”, reverse-coded) is shown separately and was excluded from the composite on conceptual and empirical grounds (Cronbach’s α decreased from 0.710 to 0.669 when C10 was added). The dashed vertical line marks the 50% threshold. ↑ represents an increase, while the ↓ represents a decrease.

Item-level analysis of the Knowledge subscale (Table 3; Figure 3) revealed a heterogeneous pattern of factual recognition. The highest accuracy was observed for processed meat as a risk factor (C1: 80.1% correct), physical activity as a protective factor (C8: 77.7%), and the protective role of ≥ 400 g per day of fruit and vegetables (C7: 77.1%), where all three correspond to clearly publicised public-health messages. Intermediate levels of recognition were observed for smoking (C5: 71.4%), obesity (C6: 71.8%), alcohol (C4: 67.1%), and fibre (C3: 63.8%). The lowest accuracy was observed for excessive red meat consumption (C2: 58.5% correct) and for the role of moderate dairy intake (C9: 44.2%), the latter being further accompanied by the highest “Don’t know” rate of the subscale (38.9%).

The misconception-detection item C10 (“Carbonated water increases the risk of cancer”) was correctly rejected by only 41.5% of respondents; 49.8% reported not knowing, and 8.6% endorsed the false statement, illustrating the persistence of a folk belief unsupported by scientific evidence.

#### 3.4.2. Composite Knowledge Score and Sociodemographic Variation

The composite knowledge score (C1–C9, range 0–9) had a mean of 6.12 ± 2.25 and a median of 6 (interquartile range 5–8). The distribution was left-skewed, with 49 participants (16.3%) achieving the maximum score of 9, a further 98 (32.6%) scoring 7 or 8, and 64 participants (21.3%) scoring 4 or below. Ten participants (3.3%) answered every item incorrectly or with “Don’t know”.

**Figure 4:**
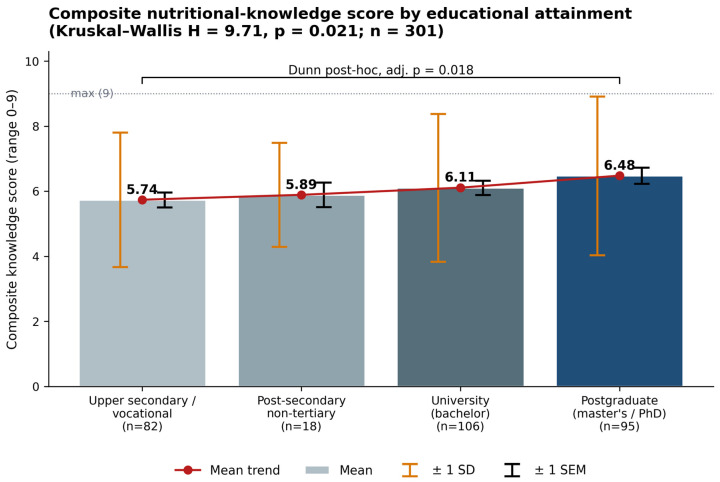
Composite nutritional-knowledge score (range 0–9) by educational attainment, *n* = 301. Bars show group means; orange whiskers represent ±1 standard deviation (within-group dispersion), and black whiskers represent ±1 standard error of the mean (precision of the group-mean estimate). The red line traces the mean across the educational gradient. Educational attainment was the only sociodemographic variable significantly associated with the composite knowledge score (Kruskal–Wallis H = 9.71, *p* = 0.021); pairwise Dunn’s post hoc comparison with Bonferroni correction localised the effect to the contrast between postgraduate and upper-secondary/vocational respondents (adjusted *p* = 0.018), as indicated by the bracket.

Stratified analyses (Table 4) identified educational attainment as the only sociodemographic variable significantly associated with the knowledge score (Kruskal–Wallis H = 9.71, *p* = 0.021). Mean scores rose monotonically from 5.74 ± 2.07 in participants with upper secondary or vocational education to 6.48 ± 2.44 in those with postgraduate qualifications. Dunn’s post hoc pairwise comparisons with Bonferroni correction localised the effect to the contrast between postgraduate and upper-secondary/vocational respondents (adjusted *p* = 0.018); pairwise differences between adjacent educational strata did not reach significance (all adjusted *p* > 0.5). A non-significant trend toward higher scores in the oldest age group was observed (60–74 years: 7.17 ± 1.50; *p* = 0.067 across age groups). Knowledge scores did not differ significantly by sex (*p* = 0.81), urban versus rural residence (*p* = 0.14), self-reported household income (*p* = 0.18), or first-degree family history of CRC (*p* = 0.99), with the absence of a family-history effect being particularly notable given that this group might be expected to be more health-information-seeking (Figure 4).

### 3.5. Attitudes Toward Dietary Change and Perceived Barriers

Item-level analyses of the Attitudes subscale showed broad endorsement of the preventive potential of diet alongside more cautious self-appraisals. Belief in the preventive role of dietary change (D2) and willingness to modify one’s diet to reduce cancer risk (D3) were strongly endorsed, with 64.1% and 63.5% of participants, respectively, selecting “partial agreement” or “total agreement”. Self-efficacy in cooking healthy meals regularly (D4) was similarly endorsed by 56.8%. Self-appraisal of one’s current diet as healthy (D1) was more reserved: only 48.2% expressed agreement, while 28.2% expressed disagreement and the remainder were neutral. The composite Motivation & Self-Efficacy subscale (D1–D4, range 4–20) had a mean score of 14.28 ± 4.41 (median 16, IQR 11–18), reflecting overall favourable but variable attitudes.

Perceived barriers were less prevalent but non-trivial. Contradictory and difficult-to-apply nutrition information (D5) was endorsed as a barrier (partial or total agreement) by 24.6% of participants; the cost of healthy eating (D6) by 20.3%; and the time required for healthy eating (D7) by 32.5%. The composite Perceived Barriers subscale (D5–D7, range 3–15) had a mean score of 8.08 ± 2.75 (median 8, IQR 6–10). Importantly, the Spearman correlation between the Motivation & Self-Efficacy and Perceived Barriers subscales was modest (ρ = 0.20, *p* < 0.001), reaffirming that these two dimensions captured conceptually and empirically distinct aspects of attitudes.

No statistically significant differences in either attitudinal subscale were observed between male and female respondents (Motivation & Self-Efficacy: *p* = 0.85; Perceived Barriers: *p* = 0.22) or across other major sociodemographic strata.

### 3.6. Self-Reported Dietary Practices and Smoking Behaviour

**Figure 5:**
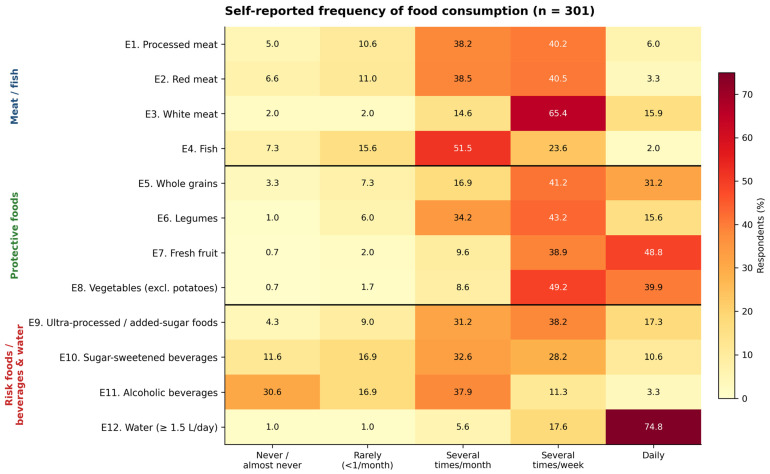
Self-reported frequency of consumption for twelve food groups (E1–E12) in the EduNutriCRC sample (*n* = 301). Cells report row-wise percentages and sum to 100% across the five frequency categories. Cell colour intensity is proportional to the percentage. Items are arranged in three thematic blocks: meat and fish (E1–E4), protective plant foods (E5–E8), and risk foods, beverages, and water (E9–E12).

Self-reported food-frequency patterns (Figure 5) revealed a profile broadly consistent with the dietary patterns of Eastern European populations and only partially aligned with current CRC-preventive recommendations. Consumption of processed meat (E1) and red meat (E2) was high: 46.2% of participants reported eating processed meat at least once a week (“several times a week” or “daily”), and 43.8% reported similar weekly or higher consumption of red meat. By contrast, fish consumption (E4) was rare, with only 25.6% reporting at least weekly intake, and 22.9% consuming fish less than once a month or never.

Intake of protective foods was relatively favourable for vegetables and fruit. Daily fruit consumption (E7) was reported by 48.8% and daily vegetable consumption (E8) by 39.9%. Daily intake of whole grains (E5) was reported by 31.2% and of legumes (E6) by 15.6%, with a further 41.2% and 43.2%, respectively, consuming these items several times a week. Adequate daily water intake (≥ 1.5 L/day, E12) was reported by 74.8% of participants. Ultra-processed and added-sugar foods (E9) were nonetheless consumed at least weekly by 55.5% and daily by 17.3%; sugar-sweetened beverages (E10) were consumed at least weekly by 38.8%. Alcohol intake was generally moderate: only 14.6% reported weekly or daily consumption, while 47.5% reported never or rare consumption.

With respect to smoking (E13), 198 participants (65.8%) reported not smoking at all; 18 (6.0%) reported occasional use (< 1 cigarette/day), 42 (14.0%) reported 1–10 cigarettes/day, 33 (11.0%) reported 11–20 cigarettes/day, and 10 (3.3%) reported more than 20 cigarettes/day.

### 3.7. Information Sources and Trust

Reported sources of nutrition and health information were dominated by digital channels. Social-media platforms (Facebook, Instagram, TikTok, YouTube) were each cited by 153 participants (50.8%) and scientific articles or academic sources by 150 (49.8%). General-purpose websites were cited by 120 (39.9%), family and friends by 117 (38.9%), and dedicated mobile health applications by 103 (34.2%). Conventional healthcare professionals were cited less often: the family doctor by 80 participants (26.6%), the dietitian or nutritionist by 77 (25.6%), specialist physicians (oncologist, gastroenterologist) by 63 (20.9%), and the pharmacist by only 26 (8.6%).

**Figure 6:**
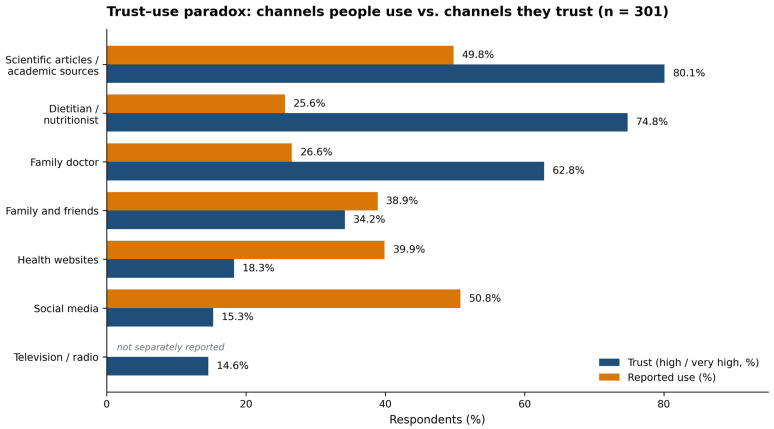
Use vs. trust of information sources on nutrition and health (*n* = 301). Reported use indicates the proportion of respondents citing each source as a channel through which they obtain nutrition-related information (multi-select item F1); trust indicates the proportion reporting “high” or “very high” trust on a 1–5 Likert scale. The mismatch is most pronounced for social media (50.8% use vs. 15.3% high trust) and is inverted for dietitians/nutritionists and family doctors (lower use, higher trust). Reported use of television/radio was not collected as a separate item.

Trust in information sources, however, followed a markedly different hierarchy from frequency of use (Table 5, Figure 6). Scientific articles received the highest mean trust score (4.18 ± 0.98 on a 1–5 scale; 80.1% of respondents reporting “high” or “very high” trust), followed by the dietitian/nutritionist (4.02 ± 0.91; 74.8% high or very high trust) and the family doctor (3.68 ± 1.01; 62.8% high or very high trust). At the lower end of the trust hierarchy were social media (2.65 ± 0.92; 15.3% high or very high trust) and television/radio (2.63 ± 0.90; 14.6% high or very high trust). Health websites occupied an intermediate position (2.84 ± 0.82; 18.3% high or very high trust). The discrepancy between the high reported use of social-media platforms (50.8%) and the low trust placed in them (15.3%) is a noteworthy finding with direct implications for nutritional-education campaigns (Section 4).

### 3.8. Awareness of Cancer Prevention Policies and Prior Dietary Counselling

**Figure 7:**
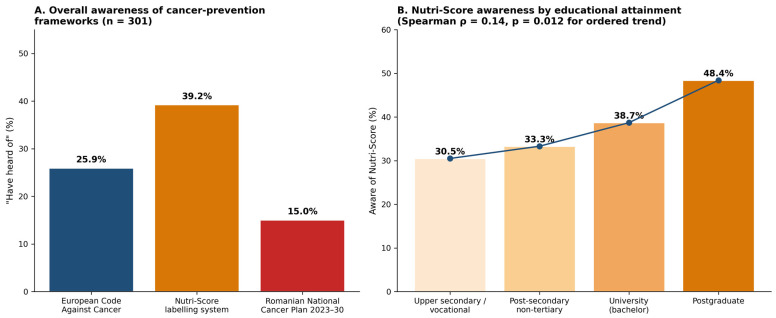
Awareness of European and Romanian cancer-prevention policy frameworks (*n* = 301). (A) Overall proportion of respondents reporting having heard of each framework: the European Code Against Cancer, the Nutri-Score front-of-pack labelling system, and the Romanian National Cancer Prevention and Control Plan 2023–2030. (B) Educational gradient for Nutri-Score awareness: the proportion of respondents aware of the system rises monotonically across ordered educational strata, with a significant ordered trend (Spearman ρ = 0.14, *p* = 0.012), although pairwise comparisons between adjacent strata did not reach significance.

Awareness of the principal European and national cancer prevention frameworks was low. Only 78 participants (25.9%) reported having heard of the European Code Against Cancer (G1), 118 (39.2%) of the Nutri-Score front-of-pack nutrition labelling system (G2), and 45 (15.0%) of the Romanian National Cancer Prevention and Control Plan 2023–2030 (G3) (Figure 7A). Familiarity with Nutri-Score increased monotonically across educational strata, from 30.5% in upper-secondary/vocational respondents to 33.3% in post-secondary non-tertiary, 38.7% in university, and 48.4% in postgraduate respondents, with a significant trend across ordered educational levels (Spearman ρ = 0.14, *p* = 0.012); the omnibus four-group χ^2^ did not reach significance (χ^2^ = 6.27, *df* = 3, *p* = 0.099), consistent with a graded rather than threshold effect. Awareness of the Romanian National Plan, by contrast, was uniformly low across all educational strata (11.1–17.9%), with no significant trend (Spearman ρ = 0.04, *p* = 0.49; Fisher’s exact *p* = 0.779).

When asked whether they had ever received dietary advice for cancer prevention from a healthcare professional (G4), 90 participants (29.9%) responded affirmatively, while 211 (70.1%) had not. The proportion having received such advice was higher among postgraduate (33.7%) and university-educated (36.8%) respondents than among those with post-secondary non-tertiary (16.7%) or upper-secondary (19.5%) education, suggesting that, in the present sample, primary-prevention dietary counselling reaches more highly educated subgroups disproportionately.

### 3.9. Knowledge–Attitude–Practice Linkages

A central question of the present pilot study was whether nutritional knowledge translated into dietary practices and attitudes consistent with CRC prevention. The data did not support a straightforward translation. The Spearman correlation between the composite knowledge score and the frequency of protective-food intake was negligible (ρ = 0.04, *p* = 0.48); the correlation between knowledge and frequency of risk-food intake was likewise negligible (ρ = −0.02, *p* = 0.77). Knowledge was also uncorrelated with the Motivation & Self-Efficacy subscale (ρ = 0.01, *p* = 0.91) and with the Perceived Barriers subscale (ρ = −0.05, *p* = 0.43).

By contrast, the Motivation & Self-Efficacy subscale showed a moderate and statistically significant inverse correlation with the frequency of risk-food intake (ρ = −0.39, *p* < 0.001), indicating that respondents with stronger positive attitudes toward dietary change reported substantially lower consumption of processed meat, red meat, ultra-processed and sugar-sweetened items, and alcohol. Notably, Motivation & Self-Efficacy was not significantly correlated with protective-food intake (ρ = 0.01, *p* = 0.84), suggesting that the attitudinal route to dietary modification, at least in this sample, operates primarily through reduction of risk foods rather than through increased uptake of protective foods.

**Figure 8:**
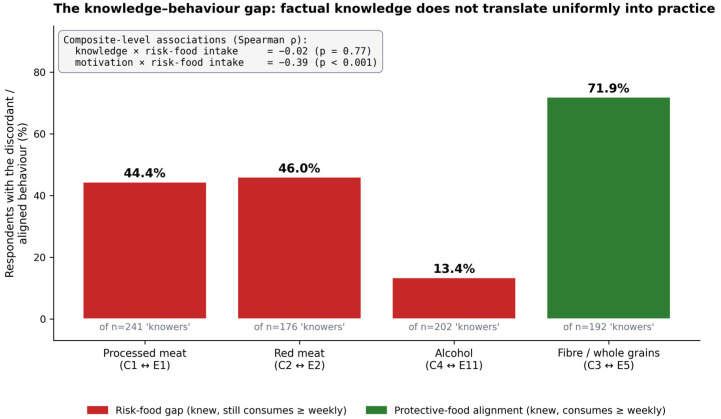
The knowledge–behaviour gap in CRC-preventive nutrition (*n* = 301). Each bar represents respondents who correctly identified a given dietary risk or protective factor on the Knowledge subscale (the “knowers”); the bar shows the proportion of that subgroup whose self-reported practice was discordant with the corresponding recommendation (red, for risk foods) or aligned with it (green, for the protective fibre/whole-grain recommendation). Composite-level Spearman correlations between the knowledge score, the Motivation & Self-Efficacy subscale, and risk-food intake are shown in the inset.

Item-level cross-tabulations confirmed and quantified the knowledge–behaviour gap (Figure 8). Among the 241 participants who correctly identified processed meat as a risk factor (C1), 44.4% nonetheless reported eating processed meat at least once a week. Among the 176 participants who correctly identified excessive red-meat consumption as a risk factor (C2), 46.0% reported eating red meat at least once a week. Among the 202 participants who correctly identified alcohol as a risk factor (C4), 13.4% reported weekly or daily alcohol consumption. Conversely, among the 192 participants who correctly identified fibre as protective (C3), 71.9% reported consuming whole grains at least once a week, with 30.7% reporting daily intake—a comparatively favourable alignment between knowledge and behaviour for this particular protective recommendation.

Taken together, these analyses indicate that, in this Romanian adult sample, factual knowledge of nutritional CRC risk and protective factors is a necessary but insufficient condition for the adoption of preventive dietary practices and that attitudinal dimensions, particularly perceived motivation and self-efficacy, represent more proximal and potentially more actionable correlates of behaviour.

### 3.10. Mediation Analysis of the KAP Framework

To test the mediational logic implicit in the KAP framework—namely that nutritional knowledge influences dietary practice through its effect on attitudes—a simple mediation model was estimated with the composite knowledge score as the independent variable, the Motivation & Self-Efficacy subscale as the mediator, and risk-food consumption as the outcome (Table 6). The model did not support mediation. The a path linking knowledge to Motivation & Self-Efficacy was negligible and non-significant (β = −0.01, *p* = 0.83), whereas the b path linking Motivation & Self-Efficacy to risk-food intake was moderate and highly significant (β = −0.38, *p* < 0.001). Consequently, the indirect (mediated) effect of knowledge on risk-food intake through attitudes was statistically indistinguishable from zero (a × b = 0.005; bias-corrected bootstrap 95% CI −0.04 to 0.05, 10,000 resamples), as were the direct effect (c′ = −0.01, *p* = 0.87) and the total effect (c = 0.00, *p* = 0.94). A parallel model with protective-food intake as the outcome was likewise non-significant at every path (indirect effect a × b ≈ 0.000; bias-corrected bootstrap 95% CI −0.01 to 0.005). To confirm that this null finding was not an artefact of the particular attitudinal mediator chosen, the Perceived Barriers subscale was additionally tested as an alternative mediator; the a path from knowledge to Perceived Barriers was also negligible and non-significant (β = −0.06, 95% CI −0.18 to 0.05, *p* = 0.27), so that, under the joint-significance criterion, the indirect effect through Perceived Barriers was likewise null. Mediation therefore failed through both attitudinal dimensions, locating the breakdown of the KAP chain specifically at the knowledge → attitudes (a) link rather than at any single attitudinal route.

Substantively, the mediation analysis indicates that the classic KAP causal chain does not operate in this sample: because knowledge is uncorrelated with attitudes, it can exert no measurable effect on practice through the attitudinal route. The only operative pathway is the direct, knowledge-independent association between Motivation & Self-Efficacy and reduced risk-food consumption. These results reposition attitudes and self-efficacy, rather than factual knowledge, as the proximal correlate of dietary behaviour, and are interpreted further in Section 4.5.

## 4. Discussion

### 4.1. Summary of Principal Findings

In this cross-sectional pilot study, we developed and provided initial psychometric evidence for the EduNutriCRC questionnaire, a 51-item Romanian-language instrument for the assessment of knowledge, attitudes, and self-reported practices (KAP) related to nutritional prevention of CRC, and we used it to characterise the corresponding KAP profile of 301 Romanian adults. Four findings are particularly relevant in this context:

(i) Internal consistency of the Knowledge subscale and a clean two-factor structure of the Attitudes subscale support the EduNutriCRC instrument as a usable starting point for population-level work, with psychometric properties broadly comparable to other recent CRC and nutrition KAP instruments [29,30,31].
(ii) Baseline awareness of CRC epidemiology and screening in this Romanian sample was strikingly poor across all three indicators assessed—the leading cancer site in Romania, the recommended screening start age, and awareness of the national screening programme.
(iii) While overall nutritional knowledge was moderate, it was both heterogeneous across items and largely decoupled from self-reported practice, with only educational attainment significantly associated with knowledge.
(iv) The Motivation & Self-Efficacy subscale, but not the knowledge score, was inversely associated with consumption of risk foods, reinforcing a behavioural-science reading in which attitudes and self-efficacy, rather than factual knowledge alone, are the more proximal correlates of dietary behaviour [35,36,37].

### 4.2. Psychometric Properties in International Perspective

The internal consistency observed for the EduNutriCRC Knowledge subscale (α = 0.710) is consistent with values reported for KAP instruments on CRC and dietary cancer prevention in other settings. Sessa et al. reported Cronbach’s α in the 0.71–0.75 range for the composite knowledge component of their southern Italian CRC KAP instrument [29], Khraiwesh et al. reported α = 0.77 across the knowledge component in Jordanian university students [31], and Alkhaldy reported α values in the 0.69–0.84 range across the various subscales of the Saudi adult CRC KAP instrument [30]. Our values therefore fall comfortably within the established range for general-population KAP scales of comparable length and item difficulty [34,44]. The exploratory factor analysis on the attitudes items followed methodological recommendations regarding sampling adequacy (KMO = 0.769), Bartlett’s test (*p* < 0.001), principal-axis extraction, varimax rotation, and the use of both the Kaiser criterion and scree-plot inspection for factor retention [45,46]. These psychometric results should be interpreted as preliminary, pilot-stage evidence rather than definitive validation. The two-factor attitudes solution was derived by exploratory factor analysis in a single sample and remains provisional pending confirmatory analysis; the Perceived Barriers subscale comprises only three items and reached only modest internal consistency, so its scores warrant cautious interpretation; and the acceptable but not high reliability of the Knowledge subscale is consistent with a deliberately heterogeneous content domain rather than a tightly unidimensional construct, so the composite knowledge score is best treated as a summary index rather than as a measure of a single latent trait. Construct validity beyond internal structure—including convergent and criterion validity against independent dietary or clinical measures—remains to be established.

The two-factor structure of the Attitudes subscale, separating Motivation & Self-Efficacy from Perceived Barriers, is theoretically congruent with the dual structure of the Health Belief Model (perceived benefits vs. perceived barriers) [35] and with the distinction between behavioural attitudes and perceived behavioural control in the Theory of Planned Behaviour [36]. Comparable two-factor or higher-order attitudinal structures have been documented in dietary KAP work in other European populations [33]. The empirical decision to exclude the misconception-detection item C10 (“Carbonated water increases the risk of cancer”) from the composite score is supported both by content-validity considerations and by the observed drop in the Cronbach’s α coefficient (0.710 → 0.669) and is consistent with the methodological recommendation to handle misconception probes as diagnostic items rather than as construct indicators [34]. A confirmatory factor analysis (CFA) in an independent and larger sample remains the appropriate next psychometric step, ideally accompanied by a test of measurement invariance across major sociodemographic strata [47].

### 4.3. Baseline Awareness of CRC Burden and Screening

The baseline awareness findings of the present study are alarming when set against the actual epidemiology of CRC in Romania [3,10]. CRC has been the most commonly diagnosed cancer in Romania since 2020 [1,3,10], yet only 8.6% of respondents correctly identified it as the leading site, and 81.7% had never heard of the National Colorectal Cancer Screening Programme. These figures are markedly worse than those reported in European populations with established screening infrastructure and align with the broader pattern of late-stage diagnosis and avoidable-mortality excess documented for Romania in the most recent OECD/European Commission country profile [3,4]. International comparative analyses across nine European countries demonstrate that the proportion of CRC diagnosed at an early, screen-detected stage is critically dependent on the maturity and population reach of organised screening programmes [48,49], with downstream consequences for mortality that explain a substantial fraction of the East–West gradient within the EU [48,50].

A particularly notable observation is that 39.9% of respondents believed screening should start at age 40, suggesting not simple ignorance but a pattern of misinformation, plausibly reflecting heterogeneous online messaging in the context of the documented global rise in early-onset CRC [5,6,7,51,52]. The shifting age distribution of CRC has direct implications for both clinical risk stratification and public communication: while the recommended population screening start age in the EU remains 50 years, the rising incidence of early-onset disease has prompted reconsideration of risk-adapted screening pathways [52,53]. The absence of any effect of first-degree family history on knowledge scores in our sample (*p* = 0.99) suggests that the educational reach of clinical encounters in high-risk relatives remains limited, an issue with direct implications for cascade screening and primary-care follow-up of CRC families [53]. Only 7.6% of our sample reported ever having taken a CRC screening test; this is broadly compatible with the low first-round colonoscopy uptake reported for the ROCCAS regional pilots [11] and with the structural and individual barriers to CRC screening identified across the wider European and eastern Mediterranean regions [12,53]. More recent guidance outside the EU has begun to lower the average-risk screening start age to 45 years in response to the rising incidence of early-onset disease, and individuals with a first-degree family history are generally advised to begin earlier still—commonly at 40 years, or ten years before the age at diagnosis of the youngest affected relative—so the EduNutriCRC screening-knowledge items could usefully be expanded to capture these risk-adapted thresholds [52,53,54]. Emerging blood-based CRC screening assays may further improve participation by reducing practical barriers, although their place within the Romanian screening pathway remains to be defined [53]. Within Romania’s statutory, near-universal health-insurance system, screening offered through the national and regional programmes is intended to be provided without direct cost to the participant, so the dominant barriers to uptake are organisational and informational rather than financial, in contrast to the reimbursement-driven access barriers that characterise some other health systems.

### 4.4. Nutritional Knowledge: Patterns and Misconceptions

The pattern of item-level knowledge in our sample replicates a finding now reported across multiple international settings: factual recognition of CRC dietary risk and protective factors is heterogeneous, with relatively strong recognition of items that are clearly publicised at the population level (processed meat as a risk factor, fruit and vegetables as protective, physical activity, smoking) and considerably weaker recognition of items that are either less intuitive or that contradict cultural eating habits (excessive red meat, dietary fibre, alcohol, dairy) [29,30,31]. The recognition of processed meat as a CRC risk factor in 80.1% of respondents likely reflects the lasting public-health impact of the 2015 IARC classification of processed meat as a Group 1 carcinogen [55], an association subsequently reaffirmed in large prospective meta-analyses of red and processed meat intake [15,56] and consolidated in umbrella reviews of dietary risk factors for CRC [57].

Conversely, the comparatively low recognition of excessive red meat consumption as a risk factor (58.5%) and of dietary fibre as protective (63.8%) is concerning in the Romanian context, where per-capita red and processed meat consumption is among the highest in the European Union and habitual fibre intake is among the lowest [14,58]. This dietary profile, combined with the cultural centrality of red meat in traditional Eastern European cuisine, makes the persistence of low risk perception for red meat a meaningful obstacle to primary prevention. The protective role of fibre and whole grains has been further reaffirmed in recent large-cohort analyses such as the NIH-AARP Diet and Health Study, in which higher whole-grain intake was associated with lower CRC incidence in a dose-dependent manner [59], and in umbrella-level reviews [57]. Diet quality more broadly, including indices of overall pattern adherence, has been independently associated with the risk of early-onset CRC precursors [60], a pattern reinforced by recent evidence linking ultra-processed food intake to both colorectal adenomas and CRC [8,9,61].

Recognition of alcohol as a risk factor (67.1%) is consistent with the broad mid-range reported across European KAP studies [29] but remains below what would be expected given decades of WHO and IARC communications and the population-level burden of alcohol-attributable cancers in Europe; Rumgay and colleagues estimated more than 740,000 alcohol-attributable cancer cases globally in 2020, with CRC contributing a substantial fraction [62]. The very low recognition of dairy as protective (44.2%) and the high “Don’t know” rate for this item (38.9%) reflect the genuinely complex and continually evolving evidence base on dairy and CRC, in which inverse associations with total dairy and milk intake have been reported in pooled cohort analyses [57] but where messaging remains less straightforward than for meat or fibre [26,27]. The finding that one in twelve respondents (8.6%) endorsed the false claim that carbonated water increases cancer risk, with another half declaring not knowing, illustrates the persistence of folk beliefs based on limited official communication. Cancer-related health misinformation has been documented to be particularly prevalent on the digital platforms most accessed by general adult populations [63]. The broader European nutritional and obesogenic context, in which traditional dietary patterns are eroded by ultra-processed food availability, is also providing a fertile ground for such misconceptions to persist [58]. It is worth underscoring that the protective dairy–CRC association is attributed chiefly to calcium rather than to dairy per se [57]; because dairy also carries other nutritional considerations, non-dairy, low-oxalate calcium sources—for example, certain leafy green vegetables—can supply calcium of comparable or greater bioavailability together with fibre, magnesium, vitamin K, and antioxidant phytochemicals, so future versions of the instrument and its accompanying educational messages could frame this protective factor as calcium-rich foods rather than dairy specifically.

### 4.5. The Knowledge–Behaviour Gap

Arguably the most actionable finding of the present study is the dissociation between knowledge and self-reported dietary practice. As reported in Section 3.9, knowledge was statistically unrelated to either protective- or risk-food intake, whereas the Motivation & Self-Efficacy subscale was moderately and inversely associated with risk-food consumption.

This pattern is one of the most reproducible findings in nutritional behavioural science: information alone is rarely sufficient to change behaviour, and effective interventions need to target capability, opportunity, and motivation in combination [64,65]. The updated Medical Research Council framework for developing and evaluating complex interventions explicitly identifies the integration of behavioural theory, context, and stakeholder engagement as core elements precisely because population-level dietary change cannot be expected to follow from message dissemination alone [65]. The dominant predictors of dietary behaviour change in cancer prevention, as in other lifestyle domains, are perceived self-efficacy, outcome expectancies, perceived barriers, social norms, and food environment exposure [36,37,64], and addressing structural drivers of health inequity in parallel is essential to avoid widening existing dietary gradients [66]. Our finding that motivation and self-efficacy are associated with reduced consumption of risk foods but not with increased consumption of protective foods deserves emphasis: it suggests that, at least in this population, the affective route to behavioural change operates by inhibition of risky habits more than by initiation of new protective habits. From an interventional standpoint, this argues that campaigns built solely around the promotion of “healthy add-ons” may underperform compared with strategies that explicitly address the substitution and reduction of risk foods [64,65].

This dissociation was confirmed by a formal mediation analysis of the KAP framework (Section 3.10; Table 6). Contrary to the sequential knowledge → attitudes → practices logic that the KAP paradigm assumes, knowledge exerted neither a direct nor an attitude-mediated effect on dietary practice: the indirect path through Motivation & Self-Efficacy was statistically indistinguishable from zero (a × b = 0.005, 95% CI −0.04 to 0.05), because knowledge and attitudes were themselves uncorrelated (β = −0.01, *p* = 0.83). The only operative association was the direct, knowledge-independent link between Motivation & Self-Efficacy and lower risk-food consumption (β = −0.38, *p* < 0.001). In behavioural-science terms, this argues against a simple information-deficit model and in favour of frameworks in which self-efficacy and motivation act as the proximal determinants of behaviour, largely decoupled from factual knowledge [36,37,64]. It also carries a methodological implication for KAP research: the customary assumption that the three KAP components form a causal chain should be tested rather than presumed, and composite KAP indices that aggregate knowledge, attitudes, and practices into a single score may obscure exactly the dissociation that is most relevant for intervention design. This conclusion was robust to the choice of attitudinal mediator: because knowledge was uncorrelated with both Motivation & Self-Efficacy and Perceived Barriers, the indirect path was null through either attitudinal route, placing the failure of the KAP chain unambiguously at the knowledge → attitudes link.

### 4.6. Attitudes, Self-Efficacy, and Perceived Barriers

The two-factor structure of the Attitudes subscale provides a parsimonious mapping of where intervention leverage lies. Across our sample, willingness to modify diet to reduce cancer risk (D3, 63.5% endorsement) and belief in the preventive role of dietary change (D2, 64.1%) were both high, while self-appraisal of the current diet as healthy (D1, 48.2%) and self-efficacy in cooking healthy meals (D4, 56.8%) lagged somewhat behind. This pattern—strong motivation and outcome expectancy with comparatively weaker self-efficacy and self-appraisal—is mirrored in dietary KAP studies in other European populations [29,33] and is theoretically consistent with the contemplation-to-action transition described in stage-based models of behaviour change [38].

Perceived barriers were less prevalent than motivation, but their composition is instructive. Time was the most frequently cited barrier (D7, 32.5% endorsement), followed by contradictory information (D5, 24.6%) and cost (D6, 20.3%). The relatively low influence of cost in this educated, mostly urban sample contrasts with European data showing that price is a primary barrier to fruit, vegetable, and fish consumption in lower-income strata and probably reflects a bias of selection in our convenience respondent sample [58,66]. The salience of “contradictory information” is, by contrast, an unusually direct expression of the information environment that has been documented in studies of food and cancer messaging on social media [63,67], where the prevalence of misinformation has been shown to be substantial and disproportionately affects topics related to lifestyle and chronic disease [63]. The modest but non-trivial cross-domain correlation between the Motivation & Self-Efficacy and Perceived Barriers subscales (ρ = 0.20) confirms that these are empirically distinct dimensions and supports the value of measuring both for intervention targeting [35,64]. The European Health Literacy Survey HLS19, which documented limited health literacy in a substantial minority of adults across seventeen European countries, provides further context for our findings on contradictory information as a perceived barrier [68].

### 4.7. Information Sources and the Trust Paradox

One of the most operationally relevant findings of this study is the discrepancy between the channels through which Romanian adults obtain nutritional information and the channels they trust. Social-media platforms were cited by about half of respondents but trusted by only a small minority—an approximately 3:1 use-to-trust ratio—whereas scientific articles and dietitians showed the inverse pattern of high trust but comparatively low use (Table 5). This trust-vs.-use asymmetry has been documented in nutritional and cancer-information studies across multiple settings and points to a structural mismatch between the supply and the perceived credibility of health information [63,67,68].

Two implications follow. First, public-health communication on dietary cancer prevention in Romania cannot rely on traditional broadcast channels (television and radio together received the lowest trust scores in our sample) and must instead engage with the digital platforms that the population actually uses, using credible messengers and evidence-based content [67]. The systematic review by Suarez-Lledo and Alvarez-Galvez documented substantial prevalence of health misinformation on the same social-media platforms most frequently used by our respondents, with cancer and dietary topics being among the most affected [63]. Second, the substantial trust placed in family doctors (62.8% high or very high) despite their relatively low citation as an information source (26.6%) represents an underused channel: family physicians remain the trusted broker of preventive information even when they are not the most frequent one. Strengthening structured dietary-counselling competencies in primary care, and embedding short, evidence-based prevention conversations into routine encounters, is therefore likely to be a high-leverage intervention [65,66]. The finding that only 29.9% of respondents reported ever having received dietary advice for cancer prevention from a healthcare professional, with this figure further stratified by educational attainment (33.7% in postgraduates vs. 19.5% in upper-secondary respondents), underscores both the unrealised potential and the equity problem of the current state of primary-prevention counselling [66,69].

### 4.8. Policy Awareness and the National Cancer Prevention Agenda

Awareness of the principal European and Romanian cancer-prevention policy frameworks was uniformly low and not commensurate with their scale of effort or strategic importance. Only 25.9% of respondents had heard of the European Code Against Cancer [27], 39.2% of the Nutri-Score front-of-pack system [70,71], and 15.0% of the Romanian National Cancer Prevention and Control Plan 2023–2030 [13]. The graded effect of educational attainment on Nutri-Score recognition, with awareness rising from 30.5% in upper-secondary respondents to 48.4% in postgraduates, is consistent with European data showing that front-of-pack labels reach the most educated consumers first [70,71] and risks widening, rather than narrowing, social gradients in dietary health unless accompanied by deliberate equity-oriented dissemination [66,69]. The very low awareness of the Romanian National Plan across all educational strata (11.1–17.9%) is a more fundamental finding: a national plan that is not known to the population it aims to protect cannot mobilise behaviour at scale [13,69]. International benchmarking of non-communicable disease policy implementation has further demonstrated that policy adoption on paper is a weak predictor of implementation in practice and that strengthening the awareness–implementation–monitoring chain is essential for chronic disease prevention objectives to be achievable [72].

### 4.9. Implications for Public-Health Practice

The combined results of this pilot study have several concrete implications for the design of nutritional educational interventions on CRC prevention in Romania, with broader relevance to other Central and Eastern European settings undergoing comparable dietary and oncological transitions [3,4,58].

First, education should be conceived not as the transfer of facts but as the support of behaviour change. Given the dissociation between knowledge and practice and the much stronger correlation of attitudes with behaviour, interventions should explicitly target self-efficacy (e.g., cooking skills, meal planning) and perceived barriers (time, cost, contradictory information) alongside factual content [37,64,65]. Recent ontological work on behaviour change interventions has emphasised the importance of standardised reporting of intervention components, mode of delivery, and mechanisms of action to permit cumulative evidence synthesis [73].

Second, content priorities should reflect the observed gaps. The strongest knowledge deficits in our sample concerned excess red meat, alcohol, fibre, and dairy. Romanian dietary recommendations should be communicated with explicit linkage to CRC prevention, with culturally adapted messaging that takes into account the centrality of meat in traditional Romanian cuisine and offers concrete substitution suggestions (e.g., partial replacement with legumes, white meat, and fish; promotion of traditional plant-based recipes) [14,58].

Third, communication should match the channels people use with the channels they trust. This argues for collaborations between scientific institutions, dietetic professional bodies, and primary-care networks to produce evidence-based content for social media and for parallel investment in primary-care counselling competencies and tools (brief interventions, decision aids, structured dietary-advice templates) [65,66,67]. Relatedly, the very low screening uptake observed here underscores the role of primary-care physicians in proactively recommending screening and in initiating dietary counselling; a concrete, testable model is structured physician referral to a dietitian—including via telehealth where in-person access is limited—supported by strengthening nutrition training within medical curricula [65,66].

Fourth, the EduNutriCRC instrument itself, beyond its primary descriptive use, can serve as a needs-assessment and monitoring tool for educational programmes. The two-factor attitudes structure permits stratification of interventions by Motivation & Self-Efficacy and Perceived Barriers profiles, allowing the targeting of subgroups most likely to benefit and the empirical evaluation of which behavioural levers an intervention activates [65].

### 4.10. Strengths and Limitations

The strengths of the present study include the structured methodological development of the EduNutriCRC instrument following established scale-development guidance [34,44], the integration of four complementary behavioural-science models in item generation [35,36,37,38], an adequate sample size for a pilot psychometric study, and the explicit anchoring in the Romanian dietary, epidemiological, and policy context [3,10,13,14].

Several limitations should be acknowledged. First, the sample was obtained by convenience and online recruitment and is biased toward urban, female, educated respondents; the present results therefore characterise a relatively favourable end of the Romanian KAP gradient, and the absolute prevalences reported here likely overestimate population-level awareness and knowledge. The next steps of this research line should include probability-based or quota-based sampling stratified by region, education, and rurality to provide nationally representative estimates [65]. Second, the cross-sectional design precludes causal inference; the associations observed between attitudes and behaviour are consistent with several causal directions and with shared third causes, and the formal causal frameworks now standard in observational epidemiology emphasise the limits of cross-sectional correlation as evidence for behavioural mechanisms [42]. Third, dietary practices were captured by self-reported food-frequency items rather than a validated dietary assessment, with the well-known limitations of social-desirability and recall bias; future versions of the instrument may benefit from coupling KAP measurement with a short validated food-frequency instrument calibrated to Eastern European populations. Fourth, factor structure was evaluated by exploratory factor analysis on a single sample; confirmatory factor analysis in an independent and larger sample, with a formal test of measurement invariance across sociodemographic strata, is required before the proposed two-factor attitudes structure can be considered established [45,47]. Fifth, the present study does not include an objective behavioural measure (e.g., 24 h recall, biomarker) and cannot adjudicate the extent to which self-reported behaviour reflects actual dietary intake. Sixth, several of the attitudes items (Section D)—particularly the self-appraisal of one’s own diet (D1), self-efficacy in meal preparation (D4), and the perceived barriers items (D5–D7)—are framed around general dietary and health orientation rather than colorectal-cancer-specific attitudes; only items D2 and D3 reference cancer risk explicitly. The Motivation & Self-Efficacy and Perceived Barriers factors therefore capture a broadly health-oriented attitudinal disposition as much as a disease-specific one, which limits the construct specificity of the Attitudes subscale and may contribute to its observed dissociation from knowledge; subsequent versions of EduNutriCRC should add CRC-specific attitude items and re-examine the dimensional structure of the subscale accordingly. Seventh, although the present pilot is underpowered for multi-group item-response-theory analyses of differential item functioning, covariate-adjusted (MIMIC) confirmatory models and single-group measurement-invariance checks are feasible with these data and are planned for the next phase. Finally, the survey did not capture insurance coverage, out-of-pocket cost, or other structural and access barriers to screening, which are recognised determinants of uptake and an important target for future instrument development.

Beyond enumerating these limitations, it is important to consider how the composition of the sample shapes the interpretation of the specific findings. Because respondents were disproportionately female, urban, university-educated, and drawn from four western counties—groups that consistently display higher health literacy and screening engagement than the general population—the awareness, knowledge, and screening figures reported here are most plausibly read as best-case estimates, with the true population-level deficits likely to be larger; this strengthens rather than weakens the public-health case for intervention. The knowledge–behaviour dissociation should be read in the same light: demonstrating that knowledge fails to track behaviour even in a comparatively high-knowledge group is a conservative test of the gap, and the configuration of attitudes, self-efficacy, and practice may differ in men, rural residents, and less-educated groups, in whom both the determinants of and the barriers to dietary change are likely to differ. Lastly, the exploratory factor structure and reliability coefficients are properties of this particular sample and cannot be assumed to hold in demographically different populations, so establishing measurement invariance across sex, education, and residence is a precondition for using EduNutriCRC to compare groups. These considerations do not invalidate the present results but bound their generalisability and identify the subgroups in which confirmation is most needed.

### 4.11. Future Directions

This pilot study lays the methodological foundation for several subsequent steps. A nationally representative survey using the EduNutriCRC instrument, ideally embedded in or aligned with existing health-monitoring infrastructures, would provide the population-level baseline that the Romanian National Cancer Prevention and Control Plan 2023–2030 currently lacks for nutritional CRC prevention [13]. Stratified analyses by region, age, education, and risk profile would identify priority subgroups for intervention. A confirmatory factor analysis and a test of measurement invariance across sociodemographic strata would strengthen the psychometric foundation of the instrument [45,47]. Finally, the EduNutriCRC instrument is well suited to serve as an outcome measure in interventional studies. We have previously demonstrated, in a methodologically related study, that a CRC-preventive dietary model emphasising red-meat reduction and increased fibre intake is acceptable to Romanian adults [14]; the natural next step is to develop and evaluate, in pragmatic or randomised designs aligned with the updated MRC framework for complex interventions, a structured educational intervention targeting the precise gaps identified here, with the EduNutriCRC instrument serving as a primary measure of pre–post change in knowledge, motivation, self-efficacy, perceived barriers, and dietary behaviour [65,73]. Future iterations of the instrument could also incorporate behaviour-change determinants not captured here—such as behavioural intention, habit strength, implementation intentions, and food-environment exposure—that may help explain why knowledge alone does not translate into practice [64,65]. Because part of this gap may reflect not knowing how to apply recommendations rather than not knowing them, intervention research should test the operationalisation of dietary guidance through practical, low-cost, and time-efficient plant-forward cooking skills, with explicit attention to food cost and palatability.

## 5. Conclusions

This pilot study developed and provided initial psychometric evidence for the EduNutriCRC questionnaire, a 51-item Romanian-language KAP instrument for nutritional CRC prevention. Acceptable internal consistency across its main subscales and a theoretically coherent two-factor attitudinal structure support its suitability as a baseline and monitoring tool for population-level educational programmes on dietary CRC prevention in Romania.

Three findings converge on a single practical conclusion. Baseline CRC awareness and screening uptake were alarmingly low, with 81.7% of respondents unaware of the National CRC Screening Programme and 92.4% never tested. Factual nutritional knowledge was moderate but largely uncorrelated with self-reported dietary behaviour, whereas Motivation & Self-Efficacy was inversely associated with risk-food consumption (ρ = −0.39, *p* < 0.001). And the channels through which Romanians obtain nutritional information (predominantly social media) are not the ones they trust (scientific articles, dietitians, family physicians), leaving trusted messengers—particularly primary-care providers—currently underused as preventive channels.

Together, these results argue for a shift in CRC nutritional education in Romania: from knowledge-centric, broadcast-style communication to self-efficacy-focused, behaviourally grounded, and culturally adapted programmes that explicitly target the reduction of risk-food consumption, strengthen primary-care dietary counselling, and engage credible messengers on the digital platforms the population actually uses. The EduNutriCRC instrument provides a usable baseline measure for such programmes. The methodological next steps are a confirmatory factor analysis in an independent sample and a nationally representative survey with stratified probability sampling; the translational next step is an interventional study using the instrument as a primary outcome measure, aligned with the objectives of Romania’s National Cancer Prevention and Control Plan 2023–2030.

## Figures and Tables

**Table 1:** Sociodemographic, anthropometric, and clinical characteristics of the analytical sample (*n* = 301).

Characteristic	*n*	%
**Age**, years (mean ± SD)	34.8 ± 13.3	—
**Age**, years (range)	19–71	—
18–29 years	117	38.9%
30–44 years	116	38.5%
45–59 years	50	16.6%
60–74 years	18	6.0%
**Sex**		
Female	241	80.1%
Male	59	19.6%
Prefer not to say	1	0.3%
**Residence**		
Urban	249	82.7%
Rural	52	17.3%
**Educational attainment**		
Upper secondary/vocational	82	27.2%
Post-secondary non-tertiary	18	6.0%
University (bachelor)	106	35.2%
Postgraduate (master’s/PhD)	95	31.6%
**Occupational status**		
Public-sector employee	91	30.2%
Student	85	28.2%
Private-sector employee	69	22.9%
Self-employed/entrepreneur	32	10.6%
Retired	12	4.0%
Homemaker	9	3.0%
Unemployed/seeking work	3	1.0%
**Monthly net household income per person**		
Below 3000 RON	40	13.3%
3000–5000 RON	56	18.6%
5001–8000 RON	76	25.2%
8001–12,000 RON	44	14.6%
Above 12,000 RON	29	9.6%
Prefer not to say	56	18.6%
**Body mass index (*n* = 297) ^1^**		
Mean ± SD, kg/m^2^	24.4 ± 4.8	—
Underweight (<18.5 kg/m^2^)	17	5.7%
Normal weight (18.5–24.9 kg/m^2^)	169	56.9%
Overweight (25.0–29.9 kg/m^2^)	75	25.3%
Obese (≥30.0 kg/m^2^)	36	12.1%
**First-degree family history of CRC**		
Yes	35	11.6%
No	266	88.4%

^1^ Four participants were excluded from BMI analysis owing to implausible self-reported height values.

**Table 2:** Varimax-rotated factor loadings for the EduNutriCRC nutritional-attitudes subscale (D1–D7) following exploratory factor analysis (principal-axis extraction, *n* = 301).

Item	F1. Motivation & Self-Efficacy	F2. Perceived Barriers
D1. I believe my current diet is healthy	0.79	−0.02
D2. Changing my diet can significantly reduce my cancer risk	0.88	0.10
D3. I am willing to modify my diet to reduce my cancer risk	0.92	0.14
D4. I feel capable of cooking healthy meals regularly	0.87	0.06
D5. Nutrition information is contradictory and hard to apply	0.19	0.69
D6. The cost of healthy eating is a real barrier for me	0.05	0.79
D7. The time required for healthy eating is a real barrier for me	0.21	0.78
Eigenvalue	3.29	1.53
Variance explained, %	46.8	21.8
Cumulative variance, %	46.8	68.7
Cronbach’s α	0.891	0.652
Ordinal α	0.913	0.688

KMO = 0.769; Bartlett’s test of sphericity χ^2^ = 958.81, *df* = 21, *p* < 0.001.

**Table 3:** Item-level distribution of responses and corrected item-total correlations for the nutritional-knowledge subscale (C1–C10, *n* = 301).

Item	Correct *n* (%)	Incorrect *n* (%)	Don’t Know *n* (%)	Item-Total r ^2^
C1. Processed meat increases CRC risk	241 (80.1%)	19 (6.3%)	41 (13.6%)	0.43
C2. Excess red meat increases CRC risk	176 (58.5%)	50 (16.6%)	75 (24.9%)	0.39
C3. Dietary fibre reduces CRC risk	192 (63.8%)	78 (25.9%)	31 (10.3%)	0.29
C4. Alcohol (even moderate) increases CRC risk	202 (67.1%)	31 (10.3%)	68 (22.6%)	0.38
C5. Smoking increases CRC risk	215 (71.4%)	22 (7.3%)	64 (21.3%)	0.43
C6. Obesity is a risk factor for CRC	216 (71.8%)	21 (7.0%)	64 (21.3%)	0.38
C7. ≥400 g fruit/vegetables per day reduces CRC risk	232 (77.1%)	31 (10.3%)	38 (12.6%)	0.44
C8. Regular physical activity reduces CRC risk	234 (77.7%)	20 (6.6%)	47 (15.6%)	0.46
C9. Moderate dairy intake is associated with reduced CRC risk	133 (44.2%)	51 (16.9%)	117 (38.9%)	0.30
C10. Carbonated water increases cancer risk ^1^	125 (41.5%)	26 (8.6%)	150 (49.8%)	—

^1^ Reverse-coded misconception item; the correct response is *False*. For C1–C9, the correct response is *True*. ^2^ Corrected item-total correlation; values above 0.20 indicate acceptable contribution to the scale. Not reported for C10, which is excluded from the composite knowledge score.

**Table 4:** Composite knowledge score (C1–C9, range 0–9) by sociodemographic stratum (*n* = 301).

Stratum	*n*	Mean ± SD	*p*-Value
**Age group**			
18–29 years	117	5.87 ± 2.15	0.067 ^2^
30–44 years	116	6.26 ± 2.31	
45–59 years	50	5.98 ± 2.45	
60–74 years	18	7.17 ± 1.50	
**Sex ^1^**			
Female	241	6.19 ± 2.11	0.81 ^3^
Male	59	5.93 ± 2.64	
**Residence**			
Urban	249	6.20 ± 2.21	0.14 ^3^
Rural	52	5.69 ± 2.39	
**Education**			
Upper secondary/vocational	82	5.74 ± 2.07	0.021 ^2^
Post-secondary non-tertiary	18	5.89 ± 1.60	
University (bachelor)	106	6.11 ± 2.27	
Postgraduate	95	6.48 ± 2.44	
**Monthly household income per person**			
Below 3000 RON	40	5.80 ± 2.13	0.18 ^2^
3000–5000 RON	56	5.93 ± 2.11	
5001–8000 RON	76	6.47 ± 1.82	
8001–12,000 RON	44	6.50 ± 2.37	
Above 12,000 RON	29	6.10 ± 2.90	
**First-degree family history of CRC**			
Yes	35	6.11 ± 2.30	0.99 ^3^
No	266	6.12 ± 2.25	

^1^ One participant who selected “prefer not to say” was excluded from the sex comparison. ^2^ Kruskal–Wallis test. ^3^ Mann–Whitney U test. All tests are two-tailed.

**Table 5:** Trust in nutrition information sources, ranked by mean Likert score (1 = very low trust, 5 = very high trust; *n* = 301).

Source	Mean Trust ± SD	High or Very High Trust (≥4), %
Scientific articles/academic sources	4.18 ± 0.98	80.1%
Dietitian/nutritionist	4.02 ± 0.91	74.8%
Family doctor	3.68 ± 1.01	62.8%
Family and friends	3.17 ± 0.89	34.2%
Health websites	2.84 ± 0.82	18.3%
Social media	2.65 ± 0.92	15.3%
Television/radio	2.63 ± 0.90	14.6%

**Table 6:** Simple mediation analysis of the KAP framework: standardised path coefficients for the model Knowledge → Motivation & Self-Efficacy → risk-food intake (*n* = 301).

Path (Standardised)	Coefficient (β)	95% CI	*p*
a: Knowledge → Motivation & Self-Efficacy	−0.01	−0.13 to 0.10	0.83
b: Motivation & Self-Efficacy → risk-food intake †	−0.38	−0.48 to −0.27	<0.001
c′: Knowledge → risk-food intake (direct) †	−0.01	−0.12 to 0.10	0.87
c: Knowledge → risk-food intake (total)	0.00	−0.12 to 0.11	0.94
a × b: Indirect (mediated) effect	0.005	−0.04 to 0.05	— ‡
a (alternative mediator): Knowledge → Perceived Barriers	−0.06	−0.18 to 0.05	0.27

Note: β, standardised path coefficient (*n* = 301); The mediator is the Motivation & Self-Efficacy subscale (D1–D4) and the outcome is the composite frequency of risk-food consumption. † Coefficient adjusted for the other predictor in the regression of the outcome on knowledge and attitudes. ‡ The indirect effect was tested with a bias-corrected non-parametric bootstrap (10,000 resamples), a 95% confidence interval excluding zero indicating statistical significance; the 95% confidence interval for the indirect effect is the bias-corrected bootstrap interval (10,000 resamples). The total effect c equals the sum of the direct effect c′ and the indirect effect a × b. A parallel model with protective-food intake as the outcome yielded a non-significant indirect effect (a × b ≈ 0.000; bias-corrected bootstrap 95% CI −0.01 to 0.005). The final row reports the a path for the alternative mediator (Perceived Barriers subscale, D5–D7); because this path is non-significant, the corresponding indirect effect is null under the joint-significance criterion and is not tabulated separately.

## Data Availability

The original contributions presented in this study are included in the article/Supplementary Material. Further inquiries can be directed to the corresponding authors.

## Supplementary Materials

The following supporting information can be downloaded at: https://www.mdpi.com/article/10.3390/nu18142293/s1, Table S1: Anonymised individual-level response data for the pilot EduNutriCRC questionnaire (*n* = 301); Table S2. Full text of the EduNutriCRC questionnaire—items, response formats, and response options.
