# Supplementary material for: The EduNutriCRC Questionnaire: A Pilot Knowledge–Attitudes–Practices Study on Nutritional Prevention of Colorectal Cancer in Romanian Adults
Source: Nutrients. 2026 Jul 13;18(14):2293. doi: 10.3390/nu18142293 (PMC13414599; doi:10.3390/nu18142293)
Supplement: Supplementary file 1 [file nutrients-18-02293-s001.zip › Table_S1.pdf]

Table S1. Anonymised individual-level response data for the pilot EduNutriCRC questionnaire (n = 301).

This supplementary table contains the de-identified, item-level responses of all 301 participants who completed the EduNutriCRC questionnaire and met the eligibility criteria of the pilot validation study. The original Excel-exported dataset (n = 305) was filtered to apply the protocol-defined exclusions (two respondents outside the eligible age range of 18–74 years; two duplicate submissions, identified by exact record matching and retained as a single entry per respondent). Direct identifiers — Google Forms timestamp and the two electronic-consent confirmation columns — have been removed; each participant has been assigned a sequential anonymous identifier (P001 to P301). Body Mass Index (BMI, kg/m²) has been derived from self-reported height and weight; four participants with implausible self-reported height values are shown with a blank BMI cell. Responses are reported verbatim in their original Romanian form to preserve the integrity of the raw data. The table is presented in seven panels, one per questionnaire section, with the anonymous Participant ID repeated on each panel. A legend mapping each item code to its full question wording is provided below.

Item legend

Code	Question / item content
A1	Age (years)
A2	Height (cm)
A3	Weight (kg)
A4	Sex
A5	Residence (urban/rural)
A6	County of residence
A7	Highest level of education attained
A8	Current occupational status
A9	Monthly net household income per person
A10	First-degree family history of colorectal cancer
B1	Perceived rank of CRC incidence in Romania
B2	Recommended age to start CRC screening
B3	Recognised warning signs of CRC (multi-select)
B4	Heard of National CRC Screening Programme
B5	Ever taken a CRC screening test (FIT/colonoscopy)
C1	Regular processed-meat consumption increases CRC risk
C2	Excess red-meat consumption increases CRC risk
C3	Regular dietary fibre consumption reduces CRC risk
C4	Alcohol (even moderate) increases CRC risk
C5	Smoking (incl. alternative tobacco) increases CRC risk
C6	Obesity is a risk factor for CRC
C7	≥400 g/day fruit and vegetables reduces CRC risk
C8	Regular physical activity reduces CRC risk
C9	Moderate dairy intake is associated with reduced CRC risk
C10	Carbonated water increases the risk of cancer (misconception)
D1	I believe my current diet is healthy
D2	Diet change can significantly reduce my cancer risk
D3	I am willing to modify my diet to reduce my cancer risk
D4	I feel capable of cooking healthy meals regularly
D5	Nutrition information is contradictory and hard to apply
D6	The cost of healthy eating is a real barrier for me
D7	The time required for healthy eating is a real barrier for me
E1	Frequency: processed meat (cold cuts, sausages, ham)
E2	Frequency: red meat (beef, pork, lamb)
E3	Frequency: white meat (chicken, turkey)
E4	Frequency: fish
E5	Frequency: whole grains
E6	Frequency: legumes (beans, lentils, chickpeas, peas)
E7	Frequency: fresh fruit
E8	Frequency: vegetables (excl. potatoes)
E9	Frequency: ultra-processed / added-sugar foods
E10	Frequency: sugar-sweetened beverages
E11	Frequency: alcoholic beverages
E12	Frequency: water (≥ 1.5 L/day)
E13	Cigarettes smoked per day (on average)

Code	Question / item content
F1	Main sources of nutrition/health information (multi-select)
F2a	Trust in: family doctor
F2b	Trust in: dietitian / nutritionist
F2c	Trust in: health websites
F2d	Trust in: social media
F2e	Trust in: television / radio
F2f	Trust in: family and friends
F2g	Trust in: scientific articles / academic sources
G1	Heard of the European Code Against Cancer
G2	Heard of the Nutri-Score nutrition labelling system
G3	Aware of the Romanian National Cancer Plan 2023–2030
G4	Ever received dietary CRC prevention advice from a health professional
BMI	Body Mass Index, kg/m² (derived from A2 and A3)

Panel A — Sociodemographic and anthropometric characteristics

Participant_ID	A1	A2	A3	BMI	A4	A5	A6	A7	A8	A9	A10
P001	19	175	68	22.2	Feminin	Rural	Bihor	Învățământ gimnazial/liceal/profesional	Student(ă)	5.001–8.000 lei	Nu
P002	19	169	48	16.81	Feminin	Urban	Bihor	Învățământ gimnazial/liceal/profesional	Student(ă)	Prefer să nu precizez	Da
P003	19	165	80	29.38	Feminin	Rural	Bihor	Învățământ gimnazial/liceal/profesional	Student(ă)	Prefer să nu precizez	Da
P004	19	156	58	23.83	Feminin	Rural	Timiș	Învățământ gimnazial/liceal/profesional	Student(ă)	Prefer să nu precizez	Nu
P005	19	165	49	18.0	Feminin	Urban	Hunedoara	Învățământ gimnazial/liceal/profesional	Student(ă)	Prefer să nu precizez	Nu
P006	19	160	72	28.12	Feminin	Rural	Timiș	Învățământ gimnazial/liceal/profesional	Student(ă)	Prefer să nu precizez	Nu
P007	19	160	53	20.7	Feminin	Rural	Timiș	Învățământ gimnazial/liceal/profesional	Student(ă)	Prefer să nu precizez	Nu
P008	19	168	53	18.78	Feminin	Rural	Bihor	Învățământ gimnazial/liceal/profesional	Student(ă)	Sub 3.000 lei	Nu
P009	19	170	58	20.07	Feminin	Urban	Maramureș	Învățământ gimnazial/liceal/profesional	Student(ă)	Prefer să nu precizez	Nu
P010	19	165	57	20.94	Feminin	Urban	Iași	Învățământ gimnazial/liceal/profesional	Student(ă)	Prefer să nu precizez	Da
P011	19	187	80	22.88	Masculin	Urban	Bihor	Învățământ gimnazial/liceal/profesional	Student(ă)	5.001–8.000 lei	Nu
P012	19	176	57	18.4	Feminin	Urban	Bihor	Învățământ gimnazial/liceal/profesional	Student(ă)	Prefer să nu precizez	Da
P013	19	164	75	27.89	Feminin	Urban	Bihor	Învățământ gimnazial/liceal/profesional	Student(ă)	Prefer să nu precizez	Da
P014	19	188	83	23.48	Masculin	Urban	Bihor	Învățământ gimnazial/liceal/profesional	Student(ă)	Sub 3.000 lei	Nu
P015	19	186	74	21.39	Masculin	Urban	Bihor	Învățământ gimnazial/liceal/profesional	Student(ă)	Prefer să nu precizez	Nu
P016	19	166	60	21.77	Feminin	Rural	Bihor	Învățământ gimnazial/liceal/profesional	Student(ă)	Prefer să nu precizez	Nu
P017	19	160	68	26.56	Feminin	Rural	Maramureș	Învățământ gimnazial/liceal/profesional	Student(ă)	Sub 3.000 lei	Nu
P018	19	156	77	31.64	Feminin	Rural	Bihor	Învățământ universitar (licență)	Student(ă)	Sub 3.000 lei	Nu
P019	20	170	63	21.8	Feminin	Urban	Bihor	Învățământ gimnazial/liceal/profesional	Student(ă)	8.001–12.000 lei	Nu
P020	20	160	43	16.8	Feminin	Rural	Maramureș	Învățământ gimnazial/liceal/profesional	Student(ă)	3.000–5.000 lei	Nu
P021	20	158	56	22.43	Feminin	Urban	Bihor	Învățământ gimnazial/liceal/profesional	Student(ă)	Prefer să nu precizez	Nu
P022	20	169	54	18.91	Feminin	Urban	Bihor	Învățământ gimnazial/liceal/profesional	Student(ă)	3.000–5.000 lei	Nu
P023	20	162	76	28.96	Feminin	Rural	Bihor	Învățământ postliceal	Student(ă)	3.000–5.000 lei	Nu
P024	20	198	80	20.41	Masculin	Urban	Cluj	Învățământ gimnazial/liceal/profesional	Student(ă)	Sub 3.000 lei	Nu
P025	20	172	55	18.59	Feminin	Urban	Timiș	Învățământ gimnazial/liceal/profesional	Student(ă)	Sub 3.000 lei	Nu
P026	20	164	60	22.31	Feminin	Urban	Municipiul București	Învățământ gimnazial/liceal/profesional	Student(ă)	Sub 3.000 lei	Nu
P027	20	167	51	18.29	Feminin	Urban	Gorj	Învățământ gimnazial/liceal/profesional	Student(ă)	Prefer să nu precizez	Nu
P028	20	162	60	22.86	Feminin	Urban	Arad	Învățământ gimnazial/liceal/profesional	Student(ă)	5.001–8.000 lei	Nu
P029	20	160	61	23.83	Feminin	Urban	Gorj	Învățământ gimnazial/liceal/profesional	Student(ă)	3.000–5.000 lei	Nu
P030	20	160	60	23.44	Feminin	Rural	Timiș	Învățământ gimnazial/liceal/profesional	Student(ă)	Sub 3.000 lei	Nu
P031	20	160	53	20.7	Feminin	Rural	Bihor	Învățământ gimnazial/liceal/profesional	Student(ă)	Prefer să nu precizez	Nu
P032	20	165	65	23.88	Feminin	Urban	Caraș-Severin	Învățământ universitar (licență)	Student(ă)	5.001–8.000 lei	Da
P033	20	175	80	26.12	Feminin	Urban	Bihor	Învățământ gimnazial/liceal/profesional	Șomer(ă)/în căutarea unui loc de muncă	Prefer să nu precizez	Da
P034	20	170	65	22.49	Masculin	Urban	Satu Mare	Învățământ universitar (licență)	Student(ă)	8.001–12.000 lei	Nu
P035	20	157	57	23.12	Feminin	Rural	Bihor	Învățământ gimnazial/liceal/profesional	Student(ă)	Prefer să nu precizez	Nu
P036	20	180	83	25.62	Masculin	Urban	Bihor	Învățământ gimnazial/liceal/profesional	Student(ă)	Peste 12.000 lei	Nu
P037	20	170	75	25.95	Feminin	Rural	Bihor	Învățământ gimnazial/liceal/profesional	Student(ă)	Prefer să nu precizez	Da
P038	20	170	68	23.53	Masculin	Urban	Bihor	Învățământ postliceal	Student(ă)	Prefer să nu precizez	Nu
P039	20	156	66	27.12	Feminin	Rural	Bihor	Învățământ gimnazial/liceal/profesional	Student(ă)	Prefer să nu precizez	Nu
P040	20	155	60	24.97	Feminin	Urban	Bihor	Învățământ gimnazial/liceal/profesional	Student(ă)	Sub 3.000 lei	Da
P041	20	157	55	22.31	Feminin	Rural	Bihor	Învățământ gimnazial/liceal/profesional	Student(ă)	Prefer să nu precizez	Nu
P042	20	170	55	19.03	Feminin	Urban	Bihor	Învățământ gimnazial/liceal/profesional	Student(ă)	Prefer să nu precizez	Nu
P043	21	170	59	20.42	Feminin	Urban	Sibiu	Învățământ gimnazial/liceal/profesional	Student(ă)	Sub 3.000 lei	Nu
P044	21	165	50	18.37	Feminin	Urban	Arad	Învățământ gimnazial/liceal/profesional	Student(ă)	Prefer să nu precizez	Nu
P045	21	165	75	27.55	Feminin	Rural	Timiș	Învățământ gimnazial/liceal/profesional	Student(ă)	Prefer să nu precizez	Nu

Participant_ID	A1	A2	A3	BMI	A4	A5	A6	A7	A8	A9	A10
P046	21	168	56	19.84	Feminin	Urban	Timiș	Învățământ gimnazial/liceal/profesional	Student(ă)	3.000–5.000 lei	Nu
P047	21	165	50	18.37	Feminin	Urban	Mehedinți	Învățământ gimnazial/liceal/profesional	Student(ă)	Prefer să nu precizez	Nu
P048	21	180	51	15.74	Feminin	Urban	Mehedinți	Învățământ gimnazial/liceal/profesional	Student(ă)	Sub 3.000 lei	Nu
P049	21	148	38	17.35	Feminin	Urban	Timiș	Învățământ gimnazial/liceal/profesional	Student(ă)	Sub 3.000 lei	Nu
P050	21	168	68	24.09	Feminin	Rural	Timiș	Învățământ gimnazial/liceal/profesional	Student(ă)	Prefer să nu precizez	Da
P051	21	165	55	20.2	Feminin	Urban	Timiș	Învățământ gimnazial/liceal/profesional	Student(ă)	3.000–5.000 lei	Nu
P052	21	163	48	18.07	Feminin	Urban	Caraș-Severin	Învățământ gimnazial/liceal/profesional	Student(ă)	5.001–8.000 lei	Nu
P053	21	184	87	25.7	Masculin	Rural	Timiș	Învățământ universitar (licență)	Student(ă)	Prefer să nu precizez	Nu
P054	21	180	79	24.38	Masculin	Urban	Bihor	Învățământ gimnazial/liceal/profesional	Student(ă)	Peste 12.000 lei	Nu
P055	21	160	63	24.61	Feminin	Urban	Maramureș	Învățământ gimnazial/liceal/profesional	Student(ă)	3.000–5.000 lei	Nu
P056	22	168	62	21.97	Feminin	Rural	Maramureș	Învățământ gimnazial/liceal/profesional	Student(ă)	5.001–8.000 lei	Da
P057	22	174	80	26.42	Masculin	Urban	Arad	Învățământ gimnazial/liceal/profesional	Student(ă)	Sub 3.000 lei	Nu
P058	22	162	69	26.29	Feminin	Urban	Mehedinți	Învățământ gimnazial/liceal/profesional	Student(ă)	8.001–12.000 lei	Nu
P059	22	170	50	17.3	Feminin	Rural	Bihor	Învățământ universitar (licență)	Student(ă)	5.001–8.000 lei	Nu
P060	22	174	60	19.82	Feminin	Urban	Bihor	Învățământ universitar (licență)	Student(ă)	5.001–8.000 lei	Nu
P061	23	170	54	18.69	Feminin	Urban	Bihor	Învățământ universitar (licență)	Student(ă)	5.001–8.000 lei	Nu
P062	23	165	61	22.41	Feminin	Urban	Bihor	Învățământ universitar (licență)	Student(ă)	3.000–5.000 lei	Nu
P063	23	159	70	27.69	Feminin	Urban	Caraș-Severin	Învățământ gimnazial/liceal/profesional	Student(ă)	Sub 3.000 lei	Nu
P064	23	170	82	28.37	Masculin	Urban	Arad	Învățământ universitar (licență)	Student(ă)	Prefer să nu precizez	Nu
P065	23	170	58	20.07	Feminin	Urban	Caraș-Severin	Învățământ gimnazial/liceal/profesional	Student(ă)	Prefer să nu precizez	Da
P066	23	159	54	21.36	Feminin	Urban	Timiș	Învățământ gimnazial/liceal/profesional	Student(ă)	3.000–5.000 lei	Nu
P067	23	158	52	20.83	Feminin	Urban	Timiș	Învățământ universitar (licență)	Salariat(ă) în sectorul privat	Sub 3.000 lei	Nu
P068	23	158	61	24.44	Feminin	Rural	Arad	Învățământ gimnazial/liceal/profesional	Student(ă)	Prefer să nu precizez	Nu
P069	23	175	68	22.2	Feminin	Urban	Constanța	Învățământ gimnazial/liceal/profesional	Student(ă)	3.000–5.000 lei	Nu
P070	23	185	95	27.76	Masculin	Urban	Arad	Învățământ gimnazial/liceal/profesional	Student(ă)	Sub 3.000 lei	Da
P071	23	176	78	25.18	Masculin	Urban	Arad	Învățământ gimnazial/liceal/profesional	Student(ă)	Peste 12.000 lei	Nu
P072	23	165	59	21.67	Feminin	Rural	Dolj	Învățământ gimnazial/liceal/profesional	Student(ă)	8.001–12.000 lei	Nu
P073	24	173	58	19.38	Feminin	Urban	Maramureș	Învățământ universitar (licență)	Student(ă)	Prefer să nu precizez	Nu
P074	24	170	61	21.11	Feminin	Urban	Bihor	Învățământ universitar (licență)	Salariat(ă) în sectorul public	5.001–8.000 lei	Nu
P075	24	167	63	22.59	Feminin	Urban	Bihor	Studii postuniversitare (masterat/doctorat)	Casnic(ă)	Sub 3.000 lei	Nu
P076	24	168	60	21.26	Feminin	Urban	Bihor	Învățământ gimnazial/liceal/profesional	Student(ă)	3.000–5.000 lei	Nu
P077	24	170	53	18.34	Feminin	Urban	Timiș	Învățământ universitar (licență)	Student(ă)	Sub 3.000 lei	Da
P078	25	174	47	15.52	Feminin	Urban	Bihor	Învățământ universitar (licență)	Student(ă)	5.001–8.000 lei	Nu
P079	25	167	68	24.38	Feminin	Urban	Bihor	Învățământ universitar (licență)	Student(ă)	5.001–8.000 lei	Nu
P080	25	163	57	21.45	Feminin	Urban	Municipiul București	Învățământ universitar (licență)	Salariat(ă) în sectorul public	3.000–5.000 lei	Nu
P081	25	158	63	25.24	Feminin	Urban	Bihor	Învățământ universitar (licență)	Student(ă)	Sub 3.000 lei	Nu
P082	25	175	59	19.27	Feminin	Urban	Bihor	Învățământ universitar (licență)	Salariat(ă) în sectorul public	3.000–5.000 lei	Da
P083	25	158	45	18.03	Feminin	Urban	Bihor	Studii postuniversitare (masterat/doctorat)	Salariat(ă) în sectorul public	3.000–5.000 lei	Nu
P084	25	170	63	21.8	Feminin	Urban	Bihor	Învățământ universitar (licență)	Student(ă)	5.001–8.000 lei	Nu
P085	25	164	52	19.33	Feminin	Urban	Timiș	Studii postuniversitare (masterat/doctorat)	Salariat(ă) în sectorul public	3.000–5.000 lei	Nu
P086	25	174	98	32.37	Masculin	Urban	Bihor	Învățământ gimnazial/liceal/profesional	Student(ă)	Sub 3.000 lei	Nu
P087	26	164	61	22.68	Feminin	Urban	Bihor	Învățământ universitar (licență)	Salariat(ă) în sectorul public	3.000–5.000 lei	Nu
P088	26	165	56	20.57	Feminin	Urban	Bihor	Învățământ gimnazial/liceal/profesional	Salariat(ă) în sectorul privat	3.000–5.000 lei	Nu
P089	26	168	66	23.38	Feminin	Urban	Bihor	Învățământ universitar (licență)	Liber-profesionist(ă)/antreprenor(oare)	Prefer să nu precizez	Nu
P090	26	157	50	20.28	Feminin	Urban	Timiș	Învățământ universitar (licență)	Student(ă)	3.000–5.000 lei	Da
P091	26	170	64	22.15	Feminin	Urban	Bihor	Studii postuniversitare (masterat/doctorat)	Salariat(ă) în sectorul privat	5.001–8.000 lei	Nu

Participant_ID	A1	A2	A3	BMI	A4	A5	A6	A7	A8	A9	A10
P092	26	178	67	21.15	Masculin	Urban	Timiș	Învățământ universitar (licență)	Salariat(ă) în sectorul public	5.001–8.000 lei	Nu
P093	26	170	66	22.84	Feminin	Urban	Bihor	Învățământ universitar (licență)	Liber-profesionist(ă)/antreprenor(oare)	5.001–8.000 lei	Nu
P094	26	163	57	21.45	Feminin	Rural	Bihor	Învățământ postliceal	Student(ă)	Prefer să nu precizez	Nu
P095	26	172	78	26.37	Masculin	Urban	Hunedoara	Învățământ universitar (licență)	Student(ă)	Prefer să nu precizez	Nu
P096	27	169	68	23.81	Feminin	Urban	Timiș	Învățământ universitar (licență)	Salariat(ă) în sectorul public	5.001–8.000 lei	Da
P097	27	163	50	18.82	Feminin	Urban	Bihor	Studii postuniversitare (masterat/doctorat)	Salariat(ă) în sectorul public	3.000–5.000 lei	Nu
P098	27	168	58	20.55	Feminin	Urban	Bihor	Învățământ universitar (licență)	Salariat(ă) în sectorul public	3.000–5.000 lei	Nu
P099	27	164	53	19.71	Feminin	Urban	Bihor	Învățământ universitar (licență)	Salariat(ă) în sectorul public	3.000–5.000 lei	Nu
P100	27	171	62	21.2	Feminin	Urban	Bihor	Învățământ universitar (licență)	Casnic(ă)	Peste 12.000 lei	Nu
P101	27	170	62	21.45	Feminin	Urban	Timiș	Învățământ universitar (licență)	Salariat(ă) în sectorul public	5.001–8.000 lei	Nu
P102	27	164	60	22.31	Feminin	Urban	Bihor	Studii postuniversitare (masterat/doctorat)	Salariat(ă) în sectorul public	Peste 12.000 lei	Nu
P103	27	165	51	18.73	Feminin	Urban	Timiș	Învățământ universitar (licență)	Salariat(ă) în sectorul public	3.000–5.000 lei	Nu
P104	27	168	66	23.38	Feminin	Urban	Timiș	Învățământ universitar (licență)	Salariat(ă) în sectorul public	5.001–8.000 lei	Nu
P105	27	170	65	22.49	Feminin	Urban	Municipiul București	Studii postuniversitare (masterat/doctorat)	Salariat(ă) în sectorul privat	Prefer să nu precizez	Nu
P106	27	173	63	21.05	Feminin	Urban	Bihor	Studii postuniversitare (masterat/doctorat)	Salariat(ă) în sectorul privat	3.000–5.000 lei	Nu
P107	28	162	55	20.96	Feminin	Rural	Bihor	Învățământ universitar (licență)	Salariat(ă) în sectorul public	5.001–8.000 lei	Nu
P108	28	161	58	22.38	Feminin	Urban	Bihor	Studii postuniversitare (masterat/doctorat)	Salariat(ă) în sectorul privat	Sub 3.000 lei	Nu
P109	28	170	70	24.22	Feminin	Rural	Timiș	Studii postuniversitare (masterat/doctorat)	Salariat(ă) în sectorul public	5.001–8.000 lei	Nu
P110	28	165	63	23.14	Feminin	Urban	Bihor	Învățământ universitar (licență)	Salariat(ă) în sectorul public	5.001–8.000 lei	Nu
P111	28	163	55	20.7	Feminin	Rural	Bihor	Studii postuniversitare (masterat/doctorat)	Salariat(ă) în sectorul public	5.001–8.000 lei	Da
P112	28	160	56	21.87	Feminin	Urban	Dolj	Studii postuniversitare (masterat/doctorat)	Salariat(ă) în sectorul privat	5.001–8.000 lei	Nu
P113	29	162	55	20.96	Feminin	Urban	Bihor	Studii postuniversitare (masterat/doctorat)	Salariat(ă) în sectorul public	5.001–8.000 lei	Nu
P114	29	170	55	19.03	Feminin	Urban	Bihor	Învățământ universitar (licență)	Salariat(ă) în sectorul privat	5.001–8.000 lei	Nu
P115	29	170	65	22.49	Feminin	Urban	Arad	Învățământ universitar (licență)	Liber-profesionist(ă)/antreprenor(oare)	5.001–8.000 lei	Nu
P116	29	165	82	30.12	Feminin	Urban	Bihor	Învățământ universitar (licență)	Salariat(ă) în sectorul privat	Sub 3.000 lei	Da
P117	29	163	57	21.45	Feminin	Urban	Arad	Studii postuniversitare (masterat/doctorat)	Salariat(ă) în sectorul public	5.001–8.000 lei	Nu
P118	30	167	58	20.8	Feminin	Urban	Municipiul București	Studii postuniversitare (masterat/doctorat)	Salariat(ă) în sectorul public	5.001–8.000 lei	Nu
P119	30	170	56	19.38	Feminin	Urban	Bihor	Învățământ universitar (licență)	Salariat(ă) în sectorul public	5.001–8.000 lei	Nu
P120	30	178	75	23.67	Masculin	Urban	Timiș	Studii postuniversitare (masterat/doctorat)	Salariat(ă) în sectorul public	5.001–8.000 lei	Nu
P121	30	160	60	23.44	Feminin	Urban	Timiș	Studii postuniversitare (masterat/doctorat)	Salariat(ă) în sectorul privat	Peste 12.000 lei	Nu
P122	30	160	47	18.36	Feminin	Urban	Timiș	Studii postuniversitare (masterat/doctorat)	Salariat(ă) în sectorul public	5.001–8.000 lei	Nu
P123	30	185	101	29.51	Masculin	Urban	Arad	Studii postuniversitare (masterat/doctorat)	Liber-profesionist(ă)/antreprenor(oare)	3.000–5.000 lei	Nu
P124	30	164	73	27.14	Feminin	Urban	Bihor	Studii postuniversitare (masterat/doctorat)	Salariat(ă) în sectorul privat	5.001–8.000 lei	Nu
P125	30	161	58	22.38	Feminin	Rural	Bihor	Învățământ universitar (licență)	Salariat(ă) în sectorul privat	3.000–5.000 lei	Nu
P126	31	158	66	26.44	Feminin	Urban	Bihor	Învățământ universitar (licență)	Liber-profesionist(ă)/antreprenor(oare)	8.001–12.000 lei	Nu
P127	31	168	88	31.18	Feminin	Urban	Bihor	Studii postuniversitare (masterat/doctorat)	Salariat(ă) în sectorul public	5.001–8.000 lei	Nu
P128	31	190	98	27.15	Masculin	Urban	Timiș	Studii postuniversitare (masterat/doctorat)	Salariat(ă) în sectorul public	Prefer să nu precizez	Nu
P129	31	171	77	26.33	Feminin	Urban	Arad	Învățământ universitar (licență)	Salariat(ă) în sectorul public	5.001–8.000 lei	Da
P130	31	157	46	18.66	Feminin	Urban	Bihor	Învățământ gimnazial/liceal/profesional	Student(ă)	5.001–8.000 lei	Nu
P131	31	158	49	19.63	Feminin	Urban	Arad	Învățământ universitar (licență)	Liber-profesionist(ă)/antreprenor(oare)	5.001–8.000 lei	Da
P132	31	172	72	24.34	Masculin	Rural	Cluj	Studii postuniversitare (masterat/doctorat)	Salariat(ă) în sectorul privat	Peste 12.000 lei	Nu
P133	31	168	59	20.9	Feminin	Urban	Bihor	Învățământ universitar (licență)	Salariat(ă) în sectorul privat	Prefer să nu precizez	Nu
P134	31	162	67	25.53	Feminin	Urban	Arad	Învățământ universitar (licență)	Salariat(ă) în sectorul privat	8.001–12.000 lei	Nu
P135	31	167	64	22.95	Feminin	Urban	Bihor	Învățământ postliceal	Salariat(ă) în sectorul privat	3.000–5.000 lei	Nu
P136	31	160	58	22.66	Feminin	Urban	Mureș	Studii postuniversitare (masterat/doctorat)	Salariat(ă) în sectorul public	Peste 12.000 lei	Nu
P137	31	163	59	22.21	Feminin	Urban	Ilfov	Învățământ universitar (licență)	Liber-profesionist(ă)/antreprenor(oare)	Peste 12.000 lei	Nu
P138	31	160	47	18.36	Feminin	Urban	Dolj	Studii postuniversitare (masterat/doctorat)	Șomer(ă)/în căutarea unui loc de muncă	Prefer să nu precizez	Nu

Participant_ID	A1	A2	A3	BMI	A4	A5	A6	A7	A8	A9	A10
P139	31	168	51	18.07	Feminin	Urban	Dolj	Învățământ universitar (licență)	Salariat(ă) în sectorul privat	Prefer să nu precizez	Nu
P140	31	184	72	21.27	Masculin	Urban	Dolj	Studii postuniversitare (masterat/doctorat)	Salariat(ă) în sectorul privat	8.001–12.000 lei	Nu
P141	31	155	53	22.06	Feminin	Urban	Dolj	Învățământ universitar (licență)	Salariat(ă) în sectorul public	8.001–12.000 lei	Nu
P142	31	183	87	25.98	Masculin	Urban	Dolj	Studii postuniversitare (masterat/doctorat)	Salariat(ă) în sectorul public	5.001–8.000 lei	Nu
P143	31	157	47	19.07	Feminin	Urban	Timiș	Învățământ universitar (licență)	Salariat(ă) în sectorul public	5.001–8.000 lei	Nu
P144	32	166	68	24.68	Feminin	Urban	Timiș	Studii postuniversitare (masterat/doctorat)	Salariat(ă) în sectorul public	8.001–12.000 lei	Nu
P145	32	164	54	20.08	Feminin	Urban	Timiș	Studii postuniversitare (masterat/doctorat)	Salariat(ă) în sectorul public	Peste 12.000 lei	Nu
P146	32	168	54	19.13	Feminin	Urban	Bihor	Studii postuniversitare (masterat/doctorat)	Salariat(ă) în sectorul public	5.001–8.000 lei	Nu
P147	32	175	60	19.59	Feminin	Urban	Maramureș	Studii postuniversitare (masterat/doctorat)	Liber-profesionist(ă)/antreprenor(oare)	8.001–12.000 lei	Da
P148	32	163	53	19.95	Feminin	Urban	Bihor	Studii postuniversitare (masterat/doctorat)	Liber-profesionist(ă)/antreprenor(oare)	Peste 12.000 lei	Nu
P149	32	165	56	20.57	Feminin	Urban	Bihor	Studii postuniversitare (masterat/doctorat)	Salariat(ă) în sectorul privat	Peste 12.000 lei	Nu
P150	32	173	61	20.38	Feminin	Urban	Bihor	Învățământ universitar (licență)	Salariat(ă) în sectorul public	5.001–8.000 lei	Nu
P151	32	164	62	23.05	Feminin	Urban	Bihor	Studii postuniversitare (masterat/doctorat)	Liber-profesionist(ă)/antreprenor(oare)	5.001–8.000 lei	Nu
P152	32	188	115	32.54	Masculin	Urban	Bihor	Studii postuniversitare (masterat/doctorat)	Salariat(ă) în sectorul privat	8.001–12.000 lei	Nu
P153	32	155	90	37.46	Feminin	Rural	Bihor	Învățământ postliceal	Student(ă)	Prefer să nu precizez	Nu
P154	32	156	53	21.78	Feminin	Urban	Bihor	Învățământ postliceal	Casnic(ă)	Prefer să nu precizez	Nu
P155	32	169	58	20.31	Feminin	Urban	Dâmbovița	Învățământ universitar (licență)	Salariat(ă) în sectorul privat	5.001–8.000 lei	Nu
P156	32	180	80	24.69	Masculin	Urban	Cluj	Studii postuniversitare (masterat/doctorat)	Salariat(ă) în sectorul privat	8.001–12.000 lei	Nu
P157	32	163	60	22.58	Feminin	Rural	Cluj	Studii postuniversitare (masterat/doctorat)	Salariat(ă) în sectorul public	8.001–12.000 lei	Nu
P158	32	190	85	23.55	Masculin	Urban	Bihor	Învățământ universitar (licență)	Salariat(ă) în sectorul public	8.001–12.000 lei	Nu
P159	32	160	60	23.44	Feminin	Urban	Bihor	Învățământ universitar (licență)	Student(ă)	3.000–5.000 lei	Da
P160	32	160	55	21.48	Feminin	Urban	Bihor	Învățământ universitar (licență)	Liber-profesionist(ă)/antreprenor(oare)	5.001–8.000 lei	Nu
P161	32	170	68	23.53	Feminin	Urban	Timiș	Studii postuniversitare (masterat/doctorat)	Salariat(ă) în sectorul public	5.001–8.000 lei	Nu
P162	32	195	95	24.98	Masculin	Urban	Timiș	Învățământ universitar (licență)	Liber-profesionist(ă)/antreprenor(oare)	Peste 12.000 lei	Nu
P163	32	178	78	24.62	Feminin	Urban	Maramureș	Învățământ postliceal	Salariat(ă) în sectorul public	5.001–8.000 lei	Nu
P164	32	181	95	29.0	Masculin	Urban	Dolj	Învățământ universitar (licență)	Salariat(ă) în sectorul public	8.001–12.000 lei	Da
P165	33	165	54	19.83	Feminin	Urban	Bihor	Învățământ universitar (licență)	Salariat(ă) în sectorul privat	8.001–12.000 lei	Nu
P166	33	165	73	26.81	Feminin	Rural	Bihor	Învățământ postliceal	Salariat(ă) în sectorul privat	5.001–8.000 lei	Nu
P167	33	172	64	21.63	Feminin	Urban	Bihor	Studii postuniversitare (masterat/doctorat)	Salariat(ă) în sectorul public	8.001–12.000 lei	Da
P168	33	192	86	23.33	Masculin	Urban	Bihor	Studii postuniversitare (masterat/doctorat)	Liber-profesionist(ă)/antreprenor(oare)	8.001–12.000 lei	Nu
P169	33	170	55	19.03	Feminin	Urban	Galați	Învățământ universitar (licență)	Salariat(ă) în sectorul public	5.001–8.000 lei	Nu
P170	33	192	105	28.48	Masculin	Urban	Bihor	Învățământ universitar (licență)	Salariat(ă) în sectorul privat	8.001–12.000 lei	Nu
P171	33	166	63	22.86	Feminin	Urban	Bihor	Învățământ universitar (licență)	Salariat(ă) în sectorul public	Peste 12.000 lei	Nu
P172	33	162	90	34.29	Feminin	Urban	Bihor	Studii postuniversitare (masterat/doctorat)	Salariat(ă) în sectorul public	5.001–8.000 lei	Nu
P173	33	162	61	23.24	Feminin	Urban	Bihor	Studii postuniversitare (masterat/doctorat)	Salariat(ă) în sectorul privat	8.001–12.000 lei	Nu
P174	33	172	63	21.3	Feminin	Urban	Bihor	Învățământ universitar (licență)	Liber-profesionist(ă)/antreprenor(oare)	5.001–8.000 lei	Nu
P175	33	176	84	27.12	Masculin	Urban	Maramureș	Învățământ gimnazial/liceal/profesional	Salariat(ă) în sectorul public	8.001–12.000 lei	Nu
P176	33	170	70	24.22	Feminin	Urban	Hunedoara	Învățământ postliceal	Salariat(ă) în sectorul privat	Sub 3.000 lei	Nu
P177	33	167	74	26.53	Feminin	Urban	Bihor	Învățământ postliceal	Salariat(ă) în sectorul privat	Sub 3.000 lei	Nu
P178	33	195	105	27.61	Masculin	Urban	Dolj	Studii postuniversitare (masterat/doctorat)	Salariat(ă) în sectorul public	5.001–8.000 lei	Nu
P179	33	184	96	28.36	Masculin	Urban	Sibiu	Învățământ universitar (licență)	Liber-profesionist(ă)/antreprenor(oare)	5.001–8.000 lei	Nu
P180	33	160	76	29.69	Feminin	Urban	Dolj	Învățământ universitar (licență)	Salariat(ă) în sectorul public	5.001–8.000 lei	Nu
P181	34	165	90	33.06	Prefer să nu precizez	Urban	Bihor	Studii postuniversitare (masterat/doctorat)	Casnic(ă)	Prefer să nu precizez	Nu
P182	34	185	87	25.42	Masculin	Urban	Bihor	Studii postuniversitare (masterat/doctorat)	Salariat(ă) în sectorul public	5.001–8.000 lei	Nu
P183	34	165	64	23.51	Feminin	Urban	Brașov	Studii postuniversitare (masterat/doctorat)	Salariat(ă) în sectorul privat	3.000–5.000 lei	Nu
P184	34	170	79	27.34	Masculin	Urban	Bihor	Învățământ universitar (licență)	Student(ă)	3.000–5.000 lei	Nu
P185	34	168	84	29.76	Feminin	Rural	Arad	Învățământ universitar (licență)	Salariat(ă) în sectorul public	Peste 12.000 lei	Nu
P186	34	183	101	30.16	Masculin	Urban	Arad	Învățământ universitar (licență)	Salariat(ă) în sectorul public	5.001–8.000 lei	Nu
P187	34	176	64	20.66	Feminin	Urban	Bihor	Învățământ gimnazial/liceal/profesional	Liber-profesionist(ă)/antreprenor(oare)	Prefer să nu precizez	Nu

Participant_ ID	A1	A2	A3	BMI	A4	A5	A6	A7	A8	A9	A10
P188	34	170	63	21.8	Feminin	Urban	Bihor	Învățământ universitar (licență)	Salariat(ă) în sectorul public	8.001–12.000 lei	Nu
P189	34	167	57	20.44	Feminin	Urban	Timiș	Studii postuniversitare (masterat/doctorat)	Salariat(ă) în sectorul public	8.001–12.000 lei	Nu
P190	34	159	60	23.73	Feminin	Urban	Gorj	Învățământ universitar (licență)	Salariat(ă) în sectorul privat	8.001–12.000 lei	Nu
P191	35	164	51	18.96	Feminin	Urban	Bihor	Studii postuniversitare (masterat/doctorat)	Casnic(ă)	5.001–8.000 lei	Nu
P192	35	172	95	32.11	Feminin	Rural	Bihor	Învățământ universitar (licență)	Casnic(ă)	Sub 3.000 lei	Nu
P193	35	162	69	26.29	Feminin	Urban	Timiș	Învățământ universitar (licență)	Salariat(ă) în sectorul privat	Sub 3.000 lei	Nu
P194	35	172	93	31.44	Feminin	Urban	Bihor	Învățământ universitar (licență)	Salariat(ă) în sectorul privat	5.001–8.000 lei	Nu
P195	35	164	73	27.14	Feminin	Urban	Timiș	Învățământ universitar (licență)	Salariat(ă) în sectorul privat	3.000–5.000 lei	Nu
P196	35	163	94	35.38	Feminin	Urban	Hunedoara	Învățământ postliceal	Casnic(ă)	Sub 3.000 lei	Nu
P197	35	163	68	25.59	Feminin	Urban	Dolj	Studii postuniversitare (masterat/doctorat)	Salariat(ă) în sectorul public	Peste 12.000 lei	Nu
P198	35	180	65	20.06	Feminin	Urban	Dolj	Studii postuniversitare (masterat/doctorat)	Salariat(ă) în sectorul privat	Peste 12.000 lei	Nu
P199	36	195	87	22.88	Masculin	Urban	Bihor	Studii postuniversitare (masterat/doctorat)	Liber-profesionist(ă)/antrepren or(oare)	Peste 12.000 lei	Da
P200	36	172	102	34.48	Feminin	Urban	Bihor	Învățământ universitar (licență)	Salariat(ă) în sectorul privat	3.000–5.000 lei	Nu
P201	36	168	63	22.32	Feminin	Urban	Timiș	Studii postuniversitare (masterat/doctorat)	Salariat(ă) în sectorul public	8.001–12.000 lei	Nu
P202	36	150	60	26.67	Feminin	Urban	Dolj	Studii postuniversitare (masterat/doctorat)	Salariat(ă) în sectorul public	Peste 12.000 lei	Nu
P203	36	182	73	22.04	Masculin	Urban	Dolj	Învățământ universitar (licență)	Salariat(ă) în sectorul privat	Peste 12.000 lei	Nu
P204	36	165	80	29.38	Feminin	Urban	Dolj	Studii postuniversitare (masterat/doctorat)	Salariat(ă) în sectorul privat	5.001–8.000 lei	Nu
P205	36	180	91	28.09	Masculin	Urban	Arad	Învățământ universitar (licență)	Salariat(ă) în sectorul privat	8.001–12.000 lei	Nu
P206	36	163	75	28.23	Feminin	Urban	Dolj	Studii postuniversitare (masterat/doctorat)	Salariat(ă) în sectorul privat	8.001–12.000 lei	Nu
P207	37	173	90	30.07	Feminin	Urban	Bihor	Învățământ postliceal	Salariat(ă) în sectorul privat	8.001–12.000 lei	Nu
P208	37	158	62	24.84	Feminin	Urban	Timiș	Învățământ gimnazial/liceal/profesional	Salariat(ă) în sectorul public	3.000–5.000 lei	Nu
P209	38	170	68	23.53	Feminin	Urban	Bihor	Studii postuniversitare (masterat/doctorat)	Salariat(ă) în sectorul privat	8.001–12.000 lei	Nu
P210	38	174	93	30.72	Masculin	Urban	Bihor	Învățământ universitar (licență)	Salariat(ă) în sectorul public	5.001–8.000 lei	Nu
P211	38	167	60	21.51	Feminin	Rural	Bihor	Studii postuniversitare (masterat/doctorat)	Salariat(ă) în sectorul privat	Prefer să nu precizez	Nu
P212	38	164	52	19.33	Feminin	Urban	Cluj	Studii postuniversitare (masterat/doctorat)	Salariat(ă) în sectorul public	5.001–8.000 lei	Nu
P213	39	172	72	24.34	Feminin	Rural	Bihor	Învățământ universitar (licență)	Salariat(ă) în sectorul privat	Sub 3.000 lei	Nu
P214	39	167	68	24.38	Feminin	Urban	Bihor	Studii postuniversitare (masterat/doctorat)	Liber-profesionist(ă)/antrepren or(oare)	5.001–8.000 lei	Nu
P215	39	166	85	30.85	Feminin	Rural	Arad	Învățământ universitar (licență)	Salariat(ă) în sectorul privat	8.001–12.000 lei	Nu
P216	39	150	52	23.11	Feminin	Urban	Dolj	Studii postuniversitare (masterat/doctorat)	Salariat(ă) în sectorul public	Sub 3.000 lei	Nu
P217	40	174	58	19.16	Feminin	Urban	Bihor	Studii postuniversitare (masterat/doctorat)	Salariat(ă) în sectorul privat	Prefer să nu precizez	Nu
P218	40	165	56	20.57	Feminin	Urban	Bihor	Învățământ universitar (licență)	Salariat(ă) în sectorul privat	Prefer să nu precizez	Nu
P219	40	159	64	25.32	Feminin	Rural	Bihor	Învățământ universitar (licență)	Liber-profesionist(ă)/antrepren or(oare)	8.001–12.000 lei	Nu
P220	40	180	78	24.07	Masculin	Urban	Arad	Studii postuniversitare (masterat/doctorat)	Salariat(ă) în sectorul public	Peste 12.000 lei	Nu
P221	40	171	98	33.51	Feminin	Urban	Bihor	Învățământ universitar (licență)	Salariat(ă) în sectorul privat	5.001–8.000 lei	Nu
P222	41	162	61	23.24	Feminin	Urban	Hunedoara	Învățământ universitar (licență)	Salariat(ă) în sectorul public	8.001–12.000 lei	Nu
P223	41	158	67	26.84	Feminin	Rural	Timiș	Studii postuniversitare (masterat/doctorat)	Salariat(ă) în sectorul public	8.001–12.000 lei	Nu
P224	41	153	44	18.8	Feminin	Rural	Bihor	Studii postuniversitare (masterat/doctorat)	Liber-profesionist(ă)/antrepren or(oare)	5.001–8.000 lei	Nu
P225	41	157	68	27.59	Feminin	Rural	Dolj	Învățământ universitar (licență)	Salariat(ă) în sectorul privat	3.000–5.000 lei	Nu
P226	42	162	61	23.24	Feminin	Urban	Bihor	Învățământ postliceal	Salariat(ă) în sectorul privat	3.000–5.000 lei	Da
P227	42	150	62	27.56	Feminin	Urban	Dolj	Învățământ universitar (licență)	Salariat(ă) în sectorul privat	3.000–5.000 lei	Nu
P228	42	167	64	22.95	Feminin	Urban	Dolj	Studii postuniversitare (masterat/doctorat)	Liber-profesionist(ă)/antrepren or(oare)	Peste 12.000 lei	Da
P229	43	176	135	43.58	Masculin	Urban	Bihor	Studii postuniversitare (masterat/doctorat)	Salariat(ă) în sectorul public	8.001–12.000 lei	Nu
P230	43	167	80	28.69	Feminin	Urban	Timiș	Studii postuniversitare (masterat/doctorat)	Salariat(ă) în sectorul public	Prefer să nu precizez	Nu
P231	43	168	75	26.57	Feminin	Urban	Timiș	Studii postuniversitare (masterat/doctorat)	Liber-profesionist(ă)/antrepren or(oare)	3.000–5.000 lei	Nu
P232	43	165	90	33.06	Feminin	Urban	Dolj	Învățământ universitar (licență)	Salariat(ă) în sectorul privat	8.001–12.000 lei	Nu
P233	44	156	70	28.76	Feminin	Urban	Bihor	Învățământ gimnazial/liceal/profesional	Salariat(ă) în sectorul privat	5.001–8.000 lei	Da
P234	45	170	68	23.53	Feminin	Urban	Bihor	Studii postuniversitare (masterat/doctorat)	Liber-profesionist(ă)/antrepren or(oare)	8.001–12.000 lei	Nu

Participant_ID	A1	A2	A3	BMI	A4	A5	A6	A7	A8	A9	A10
P235	45	166	53	19.23	Feminin	Urban	Bihor	Studii postuniversitare (masterat/doctorat)	Liber-profesionist(ă)/antreprenor(oare)	Prefer să nu precizez	Nu
P236	45	165	63	23.14	Feminin	Urban	Timiș	Studii postuniversitare (masterat/doctorat)	Salariat(ă) în sectorul privat	Prefer să nu precizez	Nu
P237	45	168	69	24.45	Feminin	Rural	Maramureș	Studii postuniversitare (masterat/doctorat)	Salariat(ă) în sectorul public	8.001–12.000 lei	Nu
P238	45	168	64	22.68	Masculin	Urban	Dolj	Învățământ universitar (licență)	Salariat(ă) în sectorul privat	5.001–8.000 lei	Nu
P239	46	165	68	24.98	Feminin	Rural	Hunedoara	Învățământ postliceal	Salariat(ă) în sectorul public	3.000–5.000 lei	Nu
P240	47	170	65	22.49	Feminin	Urban	Bihor	Învățământ universitar (licență)	Liber-profesionist(ă)/antreprenor(oare)	3.000–5.000 lei	Nu
P241	47	170	78	26.99	Feminin	Urban	Timiș	Studii postuniversitare (masterat/doctorat)	Salariat(ă) în sectorul privat	Sub 3.000 lei	Nu
P242	47	172	110	37.18	Masculin	Urban	Dolj	Învățământ universitar (licență)	Salariat(ă) în sectorul privat	3.000–5.000 lei	Nu
P243	47	165	79	29.02	Feminin	Urban	Dolj	Învățământ universitar (licență)	Salariat(ă) în sectorul public	3.000–5.000 lei	Nu
P244	47	162	78	29.72	Feminin	Urban	Dolj	Învățământ universitar (licență)	Salariat(ă) în sectorul privat	3.000–5.000 lei	Nu
P245	48	48	85	—	Feminin	Rural	Bihor	Învățământ gimnazial/liceal/profesional	Salariat(ă) în sectorul privat	Sub 3.000 lei	Nu
P246	48	161	85	32.79	Feminin	Urban	Alba	Învățământ postliceal	Salariat(ă) în sectorul public	3.000–5.000 lei	Nu
P247	48	160	56	21.87	Feminin	Urban	Hunedoara	Învățământ universitar (licență)	Salariat(ă) în sectorul privat	Sub 3.000 lei	Nu
P248	48	170	69	23.88	Feminin	Urban	Dolj	Învățământ universitar (licență)	Salariat(ă) în sectorul privat	Sub 3.000 lei	Nu
P249	49	165	68	24.98	Feminin	Rural	Bihor	Învățământ gimnazial/liceal/profesional	Salariat(ă) în sectorul public	3.000–5.000 lei	Nu
P250	50	165	85	31.22	Feminin	Urban	Arad	Studii postuniversitare (masterat/doctorat)	Salariat(ă) în sectorul public	8.001–12.000 lei	Nu
P251	50	170	80	27.68	Feminin	Urban	Timiș	Învățământ postliceal	Salariat(ă) în sectorul privat	3.000–5.000 lei	Nu
P252	50	176	60	19.37	Feminin	Urban	Dolj	Studii postuniversitare (masterat/doctorat)	Salariat(ă) în sectorul public	5.001–8.000 lei	Nu
P253	51	183	82	24.49	Masculin	Urban	Timiș	Studii postuniversitare (masterat/doctorat)	Salariat(ă) în sectorul public	Prefer să nu precizez	Nu
P254	52	160	70	27.34	Feminin	Urban	Timiș	Studii postuniversitare (masterat/doctorat)	Salariat(ă) în sectorul public	8.001–12.000 lei	Nu
P255	53	170	62	21.45	Feminin	Urban	Timiș	Învățământ universitar (licență)	Liber-profesionist(ă)/antreprenor(oare)	3.000–5.000 lei	Nu
P256	53	165	56	20.57	Feminin	Urban	Municipiul București	Studii postuniversitare (masterat/doctorat)	Liber-profesionist(ă)/antreprenor(oare)	5.001–8.000 lei	Nu
P257	54	187	96	27.45	Masculin	Urban	Arad	Învățământ universitar (licență)	Liber-profesionist(ă)/antreprenor(oare)	Prefer să nu precizez	Nu
P258	54	60	63	—	Feminin	Urban	Dolj	Învățământ universitar (licență)	Salariat(ă) în sectorul privat	3.000–5.000 lei	Da
P259	54	172	95	32.11	Feminin	Urban	Dolj	Învățământ postliceal	Salariat(ă) în sectorul privat	3.000–5.000 lei	Nu
P260	54	181	87	26.56	Masculin	Urban	Arad	Învățământ universitar (licență)	Salariat(ă) în sectorul public	Prefer să nu precizez	Nu
P261	55	170	64	22.15	Feminin	Urban	Bihor	Studii postuniversitare (masterat/doctorat)	Liber-profesionist(ă)/antreprenor(oare)	5.001–8.000 lei	Nu
P262	55	168	60	21.26	Feminin	Urban	Arad	Studii postuniversitare (masterat/doctorat)	Liber-profesionist(ă)/antreprenor(oare)	5.001–8.000 lei	Nu
P263	55	163	67	25.22	Feminin	Rural	Bihor	Învățământ universitar (licență)	Salariat(ă) în sectorul privat	Sub 3.000 lei	Da
P264	55	180	85	26.23	Masculin	Urban	Dolj	Învățământ universitar (licență)	Salariat(ă) în sectorul privat	Peste 12.000 lei	Nu
P265	56	169	75	26.26	Feminin	Urban	Bihor	Studii postuniversitare (masterat/doctorat)	Salariat(ă) în sectorul privat	8.001–12.000 lei	Nu
P266	56	174	98	32.37	Feminin	Urban	Bihor	Studii postuniversitare (masterat/doctorat)	Salariat(ă) în sectorul public	5.001–8.000 lei	Nu
P267	56	177	103	32.88	Masculin	Urban	Dolj	Învățământ postliceal	Salariat(ă) în sectorul privat	5.001–8.000 lei	Nu
P268	56	161	115	44.37	Feminin	Urban	Dolj	Învățământ universitar (licență)	Șomer(ă)/în căutarea unui loc de muncă	Sub 3.000 lei	Nu
P269	57	162	66	25.15	Feminin	Urban	Municipiul București	Învățământ universitar (licență)	Salariat(ă) în sectorul public	3.000–5.000 lei	Da
P270	57	168	80	28.34	Feminin	Urban	Arad	Studii postuniversitare (masterat/doctorat)	Salariat(ă) în sectorul public	Peste 12.000 lei	Nu
P271	57	155	82	34.13	Feminin	Urban	Dolj	Învățământ gimnazial/liceal/profesional	Salariat(ă) în sectorul privat	3.000–5.000 lei	Nu
P272	57	167	101	36.21	Masculin	Urban	Dolj	Învățământ gimnazial/liceal/profesional	Salariat(ă) în sectorul privat	3.000–5.000 lei	Nu
P273	58	170	62	21.45	Feminin	Urban	Arad	Studii postuniversitare (masterat/doctorat)	Salariat(ă) în sectorul public	Peste 12.000 lei	Nu
P274	58	182	81	24.45	Masculin	Urban	Arad	Învățământ universitar (licență)	Salariat(ă) în sectorul privat	3.000–5.000 lei	Nu
P275	58	176	90	29.05	Masculin	Urban	Satu Mare	Studii postuniversitare (masterat/doctorat)	Salariat(ă) în sectorul public	8.001–12.000 lei	Nu
P276	58	162	60	22.86	Feminin	Urban	Arad	Studii postuniversitare (masterat/doctorat)	Salariat(ă) în sectorul public	Peste 12.000 lei	Nu
P277	58	162	68	25.91	Feminin	Urban	Satu Mare	Studii postuniversitare (masterat/doctorat)	Salariat(ă) în sectorul public	5.001–8.000 lei	Nu
P278	58	165	79	29.02	Feminin	Urban	Timiș	Studii postuniversitare (masterat/doctorat)	Salariat(ă) în sectorul public	Peste 12.000 lei	Da
P279	58	164	57	21.19	Feminin	Urban	Arad	Învățământ universitar (licență)	Pensionar(ă)	Prefer să nu precizez	Nu
P280	59	170	79	27.34	Masculin	Urban	Timiș	Studii postuniversitare (masterat/doctorat)	Salariat(ă) în sectorul public	Peste 12.000 lei	Nu
P281	59	167	60	21.51	Feminin	Urban	Bihor	Studii postuniversitare (masterat/doctorat)	Salariat(ă) în sectorul public	Peste 12.000 lei	Nu
P282	59	169	93	32.56	Feminin	Urban	Bihor	Studii postuniversitare (masterat/doctorat)	Salariat(ă) în sectorul public	5.001–8.000 lei	Nu

Participant_ID	A1	A2	A3	BMI	A4	A5	A6	A7	A8	A9	A10
P283	59	160	69	26.95	Feminin	Urban	Dolj	Studii postuniversitare (masterat/doctorat)	Salariat(ă) în sectorul public	8.001–12.000 lei	Da
P284	60	60	102	—	Feminin	Rural	Dolj	Învățământ gimnazial/liceal/profesional	Casnic(ă)	Prefer să nu precizez	Nu
P285	60	155	102	42.46	Feminin	Rural	Dolj	Învățământ gimnazial/liceal/profesional	Casnic(ă)	Prefer să nu precizez	Nu
P286	61	160	70	27.34	Feminin	Urban	Bihor	Învățământ gimnazial/liceal/profesional	Liber-profesionist(ă)/antreprenor(oare)	3.000–5.000 lei	Nu
P287	61	171	74	25.31	Feminin	Urban	Arad	Învățământ universitar (licență)	Salariat(ă) în sectorul public	5.001–8.000 lei	Nu
P288	61	183	114	34.04	Masculin	Urban	Timiş	Studii postuniversitare (masterat/doctorat)	Salariat(ă) în sectorul public	8.001–12.000 lei	Nu
P289	62	173	88	29.4	Masculin	Rural	Dolj	Învățământ universitar (licență)	Pensionar(ă)	3.000–5.000 lei	Nu
P290	63	178	83	26.2	Masculin	Urban	Bihor	Învățământ gimnazial/liceal/profesional	Liber-profesionist(ă)/antreprenor(oare)	8.001–12.000 lei	Nu
P291	63	173	99	33.08	Masculin	Urban	Bihor	Studii postuniversitare (masterat/doctorat)	Salariat(ă) în sectorul public	Prefer să nu precizez	Nu
P292	63	162	62	23.62	Feminin	Urban	Dolj	Învățământ gimnazial/liceal/profesional	Pensionar(ă)	3.000–5.000 lei	Da
P293	64	165	100	36.73	Feminin	Urban	Bihor	Învățământ gimnazial/liceal/profesional	Pensionar(ă)	Sub 3.000 lei	Nu
P294	65	178	96	30.3	Masculin	Rural	Arad	Învățământ universitar (licență)	Pensionar(ă)	5.001–8.000 lei	Nu
P295	66	156	63	25.89	Feminin	Rural	Bihor	Studii postuniversitare (masterat/doctorat)	Pensionar(ă)	5.001–8.000 lei	Nu
P296	67	158	80	32.05	Feminin	Urban	Bihor	Învățământ universitar (licență)	Pensionar(ă)	3.000–5.000 lei	Nu
P297	67	155	79	32.88	Feminin	Urban	Dolj	Învățământ gimnazial/liceal/profesional	Pensionar(ă)	Sub 3.000 lei	Nu
P298	68	150	65	28.89	Feminin	Urban	Dolj	Învățământ gimnazial/liceal/profesional	Pensionar(ă)	Sub 3.000 lei	Nu
P299	69	17098	92	—	Feminin	Urban	Dolj	Învățământ gimnazial/liceal/profesional	Pensionar(ă)	Sub 3.000 lei	Nu
P300	71	179	76	23.72	Masculin	Urban	Arad	Învățământ universitar (licență)	Pensionar(ă)	3.000–5.000 lei	Nu
P301	71	170	80	27.68	Masculin	Urban	Dolj	Învățământ gimnazial/liceal/profesional	Pensionar(ă)	Sub 3.000 lei	Nu

Panel B — Baseline awareness of colorectal cancer and screening

Participant_ID	B1	B2	B3	B4	B5
P001	Locul 2	Nu știu	Sângerare rectală / sânge în scaun, Scădere bruscă și inexplicabilă în greutate, Modificări persistente ale tranzitului intestinal	Nu	Nu
P002	Locul 2	40 ani	Sângerare rectală / sânge în scaun, Scădere bruscă și inexplicabilă în greutate, Greață / vărsături persistente, Modificări persistente ale tranzitului intestinal	Nu	Nu
P003	Între locurile 5–10	40 ani	Sângerare rectală / sânge în scaun, Greață / vărsături persistente	Nu	Nu
P004	Locul 2	40 ani	Sângerare rectală / sânge în scaun, Scădere bruscă și inexplicabilă în greutate, Greață / vărsături persistente, Arsuri gastrice frecvente, Modificări persistente ale tranzitului intestinal	Nu	Nu
P005	Locul 1	Nu știu	Sângerare rectală / sânge în scaun, Modificări persistente ale tranzitului intestinal	Nu	Nu
P006	Locul 3	40 ani	Sângerare rectală / sânge în scaun, Scădere bruscă și inexplicabilă în greutate, Căderea părului	Nu	Nu
P007	Între locurile 5–10	Nu știu	Sângerare rectală / sânge în scaun, Modificări persistente ale tranzitului intestinal	Nu	Nu
P008	Între locurile 5–10	40 ani	Disfagie (dificultate la înghițire)	Nu	Nu
P009	Locul 4	30 ani	Sângerare rectală / sânge în scaun, Modificări persistente ale tranzitului intestinal	Nu	Nu
P010	Locul 1	30 ani	Sângerare rectală / sânge în scaun	Da, am participat	Da, FIT
P011	Locul 2	30 ani	Sângerare rectală / sânge în scaun, Scădere bruscă și inexplicabilă în greutate	Nu	Nu
P012	Locul 3	40 ani	Sângerare rectală / sânge în scaun, Scădere bruscă și inexplicabilă în greutate, Greață / vărsături persistente, Arsuri gastrice frecvente, Disfagie (dificultate la înghițire)	Nu	Nu
P013	Locul 3	50 ani	Scădere bruscă și inexplicabilă în greutate, Modificări persistente ale tranzitului intestinal, Căderea părului	Nu	Nu
P014	Locul 4	30 ani	Sângerare rectală / sânge în scaun, Scădere bruscă și inexplicabilă în greutate, Greață / vărsături persistente, Arsuri gastrice frecvente, Disfagie (dificultate la înghițire), Modificări persistente ale tranzitului intestinal, Sete excesivă, Căderea părului, Dureri articulare	Nu	Nu
P015	Locul 3	40 ani	Sângerare rectală / sânge în scaun, Greață / vărsături persistente	Nu	Nu
P016	Locul 3	40 ani	Sângerare rectală / sânge în scaun, Scădere bruscă și inexplicabilă în greutate, Modificări persistente ale tranzitului intestinal	Nu	Nu
P017	Locul 2	30 ani	Sângerare rectală / sânge în scaun, Scădere bruscă și inexplicabilă în greutate, Modificări persistente ale tranzitului intestinal	Nu	Nu
P018	Locul 2	30 ani	Scădere bruscă și inexplicabilă în greutate, Căderea părului	Nu	Nu
P019	Locul 2	40 ani	Sângerare rectală / sânge în scaun, Greață / vărsături persistente, Arsuri gastrice frecvente, Modificări persistente ale tranzitului intestinal	Nu	Nu
P020	Nu știu	30 ani	Sângerare rectală / sânge în scaun, Modificări persistente ale tranzitului intestinal	Nu	Nu
P021	Locul 4	40 ani	Sângerare rectală / sânge în scaun, Scădere bruscă și inexplicabilă în greutate, Modificări persistente ale tranzitului intestinal	Nu	Nu
P022	Locul 3	30 ani	Sângerare rectală / sânge în scaun, Scădere bruscă și inexplicabilă în greutate, Greață / vărsături persistente, Modificări persistente ale tranzitului intestinal	Nu	Nu
P023	Locul 3	30 ani	Sângerare rectală / sânge în scaun, Scădere bruscă și inexplicabilă în greutate, Greață / vărsături persistente, Modificări persistente ale tranzitului intestinal, Sete excesivă, Căderea părului, Dureri articulare	Nu	Nu
P024	Nu știu	Nu știu	Nu știu	Nu	Nu
P025	Locul 2	40 ani	Sângerare rectală / sânge în scaun, Scădere bruscă și inexplicabilă în greutate, Modificări persistente ale tranzitului intestinal	Nu	Nu
P026	Locul 2	50 ani	Sângerare rectală / sânge în scaun, Scădere bruscă și inexplicabilă în greutate, Modificări persistente ale tranzitului intestinal	Nu	Nu
P027	Locul 2	50 ani	Sângerare rectală / sânge în scaun, Scădere bruscă și inexplicabilă în greutate, Greață / vărsături persistente, Modificări persistente ale tranzitului intestinal	Mi-a fost recomandat să particip	Nu
P028	Locul 3	40 ani	Sângerare rectală / sânge în scaun, Scădere bruscă și inexplicabilă în greutate, Greață / vărsături persistente, Modificări persistente ale tranzitului intestinal	Nu	Nu
P029	Locul 3	40 ani	Sângerare rectală / sânge în scaun, Modificări persistente ale tranzitului intestinal	Nu	Nu
P030	Locul 3	40 ani	Sângerare rectală / sânge în scaun, Scădere bruscă și inexplicabilă în greutate, Arsuri gastrice frecvente	Nu	Nu
P031	Locul 3	40 ani	Sângerare rectală / sânge în scaun, Scădere bruscă și inexplicabilă în greutate, Greață / vărsături persistente	Nu	Nu
P032	Nu știu	Nu știu	Sângerare rectală / sânge în scaun, Greață / vărsături persistente, Arsuri gastrice frecvente, Căderea părului, Dureri articulare	Nu	Nu
P033	Locul 3	30 ani	Scădere bruscă și inexplicabilă în greutate	Da, dar am întâmpinat dificultăți în accesare	Nu
P034	Locul 3	Nu știu	Sângerare rectală / sânge în scaun, Scădere bruscă și inexplicabilă în greutate, Greață / vărsături persistente	Nu	Nu
P035	Locul 2	30 ani	Sângerare rectală / sânge în scaun, Scădere bruscă și inexplicabilă în greutate, Greață / vărsături persistente, Arsuri gastrice frecvente, Modificări persistente ale tranzitului intestinal	Nu	Nu
P036	Locul 3	30 ani	Sângerare rectală / sânge în scaun, Scădere bruscă și inexplicabilă în greutate, Greață / vărsături persistente, Modificări persistente ale tranzitului intestinal	Nu	Nu
P037	Locul 2	40 ani	Sângerare rectală / sânge în scaun, Scădere bruscă și inexplicabilă în greutate	Nu	Nu
P038	Între locurile 5–10	30 ani	Sângerare rectală / sânge în scaun	Nu	Nu
P039	Locul 3	30 ani	Sângerare rectală / sânge în scaun, Scădere bruscă și inexplicabilă în greutate, Modificări persistente ale tranzitului intestinal	Nu	Nu
P040	Locul 2	30 ani	Sângerare rectală / sânge în scaun, Scădere bruscă și inexplicabilă în greutate, Modificări persistente ale tranzitului intestinal	Nu	Nu
P041	Locul 3	40 ani	Sângerare rectală / sânge în scaun, Modificări persistente ale tranzitului intestinal, Nu știu	Nu	Nu
P042	Locul 4	40 ani	Sângerare rectală / sânge în scaun, Scădere bruscă și inexplicabilă în greutate, Greață / vărsături persistente	Nu	Nu
P043	Nu știu	40 ani	Sângerare rectală / sânge în scaun, Scădere bruscă și inexplicabilă în greutate, Greață / vărsături persistente	Nu	Nu

Participant_ID	B1	B2	B3	B4	B5
P044	Nu știu	Nu știu	Sângerare rectală / sânge în scaun, Scădere bruscă și inexplicabilă în greutate, Greață / vărsături persistente, Modificări persistente ale tranzitului intestinal	Nu	Nu
P045	Locul 3	40 ani	Scădere bruscă și inexplicabilă în greutate, Greață / vărsături persistente, Arsuri gastrice frecvente, Modificări persistente ale tranzitului intestinal	Nu	Nu
P046	Locul 4	30 ani	Sângerare rectală / sânge în scaun, Scădere bruscă și inexplicabilă în greutate, Modificări persistente ale tranzitului intestinal	Nu	Nu
P047	Locul 2	40 ani	Scădere bruscă și inexplicabilă în greutate, Greață / vărsături persistente, Arsuri gastrice frecvente, Modificări persistente ale tranzitului intestinal	Nu	Nu
P048	Locul 2	30 ani	Sângerare rectală / sânge în scaun, Scădere bruscă și inexplicabilă în greutate, Greață / vărsături persistente, Arsuri gastrice frecvente, Disfagie (dificultate la înghițire), Modificări persistente ale tranzitului intestinal	Nu	Nu
P049	Locul 2	50 ani	Sângerare rectală / sânge în scaun, Scădere bruscă și inexplicabilă în greutate, Greață / vărsături persistente, Modificări persistente ale tranzitului intestinal	Nu	Nu
P050	Locul 3	40 ani	Sângerare rectală / sânge în scaun, Modificări persistente ale tranzitului intestinal, Dureri articulare	Nu	Nu
P051	Locul 2	50 ani	Sângerare rectală / sânge în scaun, Scădere bruscă și inexplicabilă în greutate, Greață / vărsături persistente, Modificări persistente ale tranzitului intestinal	Nu	Nu
P052	Locul 2	40 ani	Sângerare rectală / sânge în scaun, Scădere bruscă și inexplicabilă în greutate, Modificări persistente ale tranzitului intestinal	Mi-a fost recomandat să particip	Nu
P053	Între locurile 5–10	40 ani	Sângerare rectală / sânge în scaun, Scădere bruscă și inexplicabilă în greutate, Modificări persistente ale tranzitului intestinal	Nu	Nu
P054	Locul 3	40 ani	Nu știu	Mi-a fost recomandat să particip	Nu
P055	Locul 4	Nu știu	Sângerare rectală / sânge în scaun, Greață / vărsături persistente, Căderea părului	Nu	Nu
P056	Locul 2	50 ani	Sângerare rectală / sânge în scaun, Scădere bruscă și inexplicabilă în greutate, Modificări persistente ale tranzitului intestinal, Dureri articulare	Nu	Nu
P057	Nu știu	Nu știu	Sângerare rectală / sânge în scaun, Scădere bruscă și inexplicabilă în greutate, Greață / vărsături persistente, Modificări persistente ale tranzitului intestinal	Nu	Nu
P058	Între locurile 11–15	30 ani	Sângerare rectală / sânge în scaun, Scădere bruscă și inexplicabilă în greutate, Modificări persistente ale tranzitului intestinal	Nu	Nu
P059	Nu știu	30 ani	Sângerare rectală / sânge în scaun, Arsuri gastrice frecvente, Modificări persistente ale tranzitului intestinal	Nu	Nu
P060	Locul 3	50 ani	Greață / vărsături persistente, Modificări persistente ale tranzitului intestinal, Căderea părului	Nu	Nu
P061	Locul 3	50 ani	Sângerare rectală / sânge în scaun, Scădere bruscă și inexplicabilă în greutate	Nu	Nu
P062	Locul 2	40 ani	Sângerare rectală / sânge în scaun, Scădere bruscă și inexplicabilă în greutate, Greață / vărsături persistente	Nu	Nu
P063	Locul 2	30 ani	Sângerare rectală / sânge în scaun, Scădere bruscă și inexplicabilă în greutate, Greață / vărsături persistente, Arsuri gastrice frecvente, Sete excesivă	Nu	Nu
P064	Locul 3	40 ani	Sângerare rectală / sânge în scaun, Scădere bruscă și inexplicabilă în greutate, Modificări persistente ale tranzitului intestinal	Nu	Nu
P065	Locul 2	50 ani	Sângerare rectală / sânge în scaun, Scădere bruscă și inexplicabilă în greutate, Greață / vărsături persistente, Modificări persistente ale tranzitului intestinal	Mi-a fost recomandat să particip	Nu
P066	Locul 2	40 ani	Sângerare rectală / sânge în scaun, Scădere bruscă și inexplicabilă în greutate, Modificări persistente ale tranzitului intestinal	Nu	Nu
P067	Nu știu	Nu știu	Nu știu	Nu	Nu
P068	Locul 2	40 ani	Sângerare rectală / sânge în scaun, Scădere bruscă și inexplicabilă în greutate, Modificări persistente ale tranzitului intestinal	Nu	Nu
P069	Locul 2	40 ani	Sângerare rectală / sânge în scaun, Scădere bruscă și inexplicabilă în greutate, Modificări persistente ale tranzitului intestinal	Mi-a fost recomandat să particip	Nu
P070	Locul 1	40 ani	Scădere bruscă și inexplicabilă în greutate, Modificări persistente ale tranzitului intestinal	Nu	Nu
P071	Locul 3	40 ani	Sângerare rectală / sânge în scaun, Modificări persistente ale tranzitului intestinal	Nu	Nu
P072	Locul 2	40 ani	Sângerare rectală / sânge în scaun, Scădere bruscă și inexplicabilă în greutate, Modificări persistente ale tranzitului intestinal	Mi-a fost recomandat să particip	Nu
P073	Locul 3	40 ani	Sângerare rectală / sânge în scaun, Scădere bruscă și inexplicabilă în greutate, Modificări persistente ale tranzitului intestinal	Mi-a fost recomandat să particip	Nu
P074	Locul 4	40 ani	Scădere bruscă și inexplicabilă în greutate, Modificări persistente ale tranzitului intestinal	Nu	Nu
P075	Locul 2	30 ani	Sângerare rectală / sânge în scaun, Scădere bruscă și inexplicabilă în greutate, Greață / vărsături persistente	Nu	Nu
P076	Între locurile 11–15	40 ani	Sângerare rectală / sânge în scaun, Scădere bruscă și inexplicabilă în greutate, Modificări persistente ale tranzitului intestinal	Nu	Nu
P077	Locul 4	40 ani	Sângerare rectală / sânge în scaun, Scădere bruscă și inexplicabilă în greutate, Modificări persistente ale tranzitului intestinal, Nu știu	Nu	Nu
P078	Locul 3	30 ani	Sângerare rectală / sânge în scaun, Scădere bruscă și inexplicabilă în greutate, Greață / vărsături persistente, Arsuri gastrice frecvente, Modificări persistente ale tranzitului intestinal	Nu	Nu
P079	Locul 2	50 ani	Sângerare rectală / sânge în scaun, Scădere bruscă și inexplicabilă în greutate, Greață / vărsături persistente, Disfagie (dificultate la înghițire), Modificări persistente ale tranzitului intestinal	Nu	Nu
P080	Locul 2	50 ani	Sângerare rectală / sânge în scaun, Scădere bruscă și inexplicabilă în greutate, Modificări persistente ale tranzitului intestinal	Nu	Nu
P081	Locul 3	50 ani	Sângerare rectală / sânge în scaun, Scădere bruscă și inexplicabilă în greutate, Greață / vărsături persistente, Modificări persistente ale tranzitului intestinal	Nu	Nu
P082	Locul 2	40 ani	Sângerare rectală / sânge în scaun, Scădere bruscă și inexplicabilă în greutate, Modificări persistente ale tranzitului intestinal	Nu	Nu
P083	Locul 2	50 ani	Sângerare rectală / sânge în scaun, Scădere bruscă și inexplicabilă în greutate, Modificări persistente ale tranzitului intestinal	Nu	Da, FIT
P084	Locul 4	50 ani	Sângerare rectală / sânge în scaun, Scădere bruscă și inexplicabilă în greutate, Modificări persistente ale tranzitului intestinal	Nu	Nu

Participant_ID	B1	B2	B3	B4	B5
P085	Locul 3	40 ani	Sângerare rectală / sânge în scaun, Scădere bruscă și inexplicabilă în greutate, Modificări persistente ale tranzitului intestinal	Nu	Nu
P086	Locul 3	30 ani	Sângerare rectală / sânge în scaun, Scădere bruscă și inexplicabilă în greutate, Greață / vărsături persistente	Mi-a fost recomandat să particip	Nu
P087	Locul 2	50 ani	Sângerare rectală / sânge în scaun, Scădere bruscă și inexplicabilă în greutate, Modificări persistente ale tranzitului intestinal	Nu	Nu
P088	Locul 3	30 ani	Sângerare rectală / sânge în scaun, Scădere bruscă și inexplicabilă în greutate, Căderea părului	Mi-a fost recomandat să particip	Nu
P089	Locul 2	40 ani	Sângerare rectală / sânge în scaun, Scădere bruscă și inexplicabilă în greutate, Greață / vărsături persistente, Modificări persistente ale tranzitului intestinal	Nu	Nu
P090	Locul 1	30 ani	Sângerare rectală / sânge în scaun, Scădere bruscă și inexplicabilă în greutate, Arsuri gastrice frecvente	Nu	Nu
P091	Nu știu	Nu știu	Nu știu	Nu	Nu
P092	Locul 1	50 ani	Sângerare rectală / sânge în scaun, Scădere bruscă și inexplicabilă în greutate, Modificări persistente ale tranzitului intestinal	Nu	Nu
P093	Locul 2	30 ani	Sângerare rectală / sânge în scaun, Scădere bruscă și inexplicabilă în greutate, Greață / vărsături persistente, Modificări persistente ale tranzitului intestinal	Nu	Nu
P094	Locul 3	40 ani	Sângerare rectală / sânge în scaun, Scădere bruscă și inexplicabilă în greutate	Nu	Nu
P095	Nu știu	Nu știu	Sângerare rectală / sânge în scaun, Scădere bruscă și inexplicabilă în greutate, Greață / vărsături persistente, Arsuri gastrice frecvente, Disfagie (dificultate la înghițire), Modificări persistente ale tranzitului intestinal, Sete excesivă, Căderea părului, Dureri articulare	Nu	Nu
P096	Locul 3	40 ani	Sângerare rectală / sânge în scaun, Scădere bruscă și inexplicabilă în greutate, Modificări persistente ale tranzitului intestinal	Nu	Nu
P097	Locul 3	30 ani	Sângerare rectală / sânge în scaun, Scădere bruscă și inexplicabilă în greutate, Greață / vărsături persistente, Arsuri gastrice frecvente	Nu	Nu
P098	Locul 2	50 ani	Sângerare rectală / sânge în scaun, Scădere bruscă și inexplicabilă în greutate, Greață / vărsături persistente	Nu	Nu
P099	Locul 1	50 ani	Sângerare rectală / sânge în scaun, Scădere bruscă și inexplicabilă în greutate, Greață / vărsături persistente, Modificări persistente ale tranzitului intestinal	Da, am participat	Nu
P100	Locul 1	40 ani	Sângerare rectală / sânge în scaun, Scădere bruscă și inexplicabilă în greutate, Greață / vărsături persistente, Arsuri gastrice frecvente, Modificări persistente ale tranzitului intestinal	Nu	Nu
P101	Locul 1	50 ani	Sângerare rectală / sânge în scaun, Scădere bruscă și inexplicabilă în greutate, Modificări persistente ale tranzitului intestinal	Da, am participat	Nu
P102	Locul 1	50 ani	Sângerare rectală / sânge în scaun, Scădere bruscă și inexplicabilă în greutate, Modificări persistente ale tranzitului intestinal	Da, am participat	Nu
P103	Locul 4	30 ani	Sângerare rectală / sânge în scaun, Scădere bruscă și inexplicabilă în greutate, Modificări persistente ale tranzitului intestinal	Nu	Nu
P104	Locul 3	50 ani	Sângerare rectală / sânge în scaun, Scădere bruscă și inexplicabilă în greutate, Greață / vărsături persistente, Arsuri gastrice frecvente, Disfagie (dificultate la înghițire), Modificări persistente ale tranzitului intestinal, Căderea părului, Dureri articulare	Mi-a fost recomandat să particip	Nu
P105	Nu știu	Nu știu	Nu știu	Nu	Nu
P106	Nu știu	40 ani	Sângerare rectală / sânge în scaun, Scădere bruscă și inexplicabilă în greutate, Arsuri gastrice frecvente	Nu	Nu
P107	Locul 1	40 ani	Sângerare rectală / sânge în scaun	Da, am participat	Nu
P108	Locul 2	Nu știu	Sângerare rectală / sânge în scaun, Scădere bruscă și inexplicabilă în greutate, Modificări persistente ale tranzitului intestinal	Nu	Nu
P109	Locul 2	40 ani	Sângerare rectală / sânge în scaun, Scădere bruscă și inexplicabilă în greutate, Modificări persistente ale tranzitului intestinal	Nu	Nu
P110	Locul 2	50 ani	Sângerare rectală / sânge în scaun, Scădere bruscă și inexplicabilă în greutate, Modificări persistente ale tranzitului intestinal	Nu	Nu
P111	Locul 3	40 ani	Sângerare rectală / sânge în scaun, Scădere bruscă și inexplicabilă în greutate, Arsuri gastrice frecvente, Modificări persistente ale tranzitului intestinal	Nu	Nu
P112	Între locurile 5–10	40 ani	Sângerare rectală / sânge în scaun, Scădere bruscă și inexplicabilă în greutate, Greață / vărsături persistente, Modificări persistente ale tranzitului intestinal	Nu	Nu
P113	Locul 2	50 ani	Sângerare rectală / sânge în scaun, Scădere bruscă și inexplicabilă în greutate	Nu	Nu
P114	Nu știu	Nu știu	Sângerare rectală / sânge în scaun, Scădere bruscă și inexplicabilă în greutate	Mi-a fost recomandat să particip	Nu
P115	Locul 2	40 ani	Sângerare rectală / sânge în scaun, Scădere bruscă și inexplicabilă în greutate, Modificări persistente ale tranzitului intestinal	Nu	Nu
P116	Locul 2	30 ani	Sângerare rectală / sânge în scaun, Scădere bruscă și inexplicabilă în greutate, Modificări persistente ale tranzitului intestinal	Nu	Nu
P117	Locul 4	40 ani	Sângerare rectală / sânge în scaun, Scădere bruscă și inexplicabilă în greutate, Greață / vărsături persistente	Nu	Nu
P118	Locul 3	40 ani	Sângerare rectală / sânge în scaun, Scădere bruscă și inexplicabilă în greutate, Modificări persistente ale tranzitului intestinal	Nu	Nu
P119	Locul 2	50 ani	Sângerare rectală / sânge în scaun, Scădere bruscă și inexplicabilă în greutate, Greață / vărsături persistente, Modificări persistente ale tranzitului intestinal	Nu	Nu
P120	Locul 2	50 ani	Sângerare rectală / sânge în scaun, Scădere bruscă și inexplicabilă în greutate, Modificări persistente ale tranzitului intestinal	Nu	Nu
P121	Locul 1	40 ani	Sângerare rectală / sânge în scaun, Scădere bruscă și inexplicabilă în greutate, Greață / vărsături persistente, Modificări persistente ale tranzitului intestinal	Nu	Nu
P122	Locul 2	Nu știu	Sângerare rectală / sânge în scaun, Scădere bruscă și inexplicabilă în greutate, Greață / vărsături persistente, Arsuri gastrice frecvente, Disfagie (dificultate la înghițire), Modificări persistente ale tranzitului intestinal	Nu	Nu
P123	Locul 3	30 ani	Sângerare rectală / sânge în scaun, Scădere bruscă și inexplicabilă în greutate, Modificări persistente ale tranzitului intestinal, Dureri articulare	Nu	Nu
P124	Nu știu	Nu știu	Modificări persistente ale tranzitului intestinal	Nu	Nu
P125	Locul 2	40 ani	Sângerare rectală / sânge în scaun, Scădere bruscă și inexplicabilă în greutate, Greață / vărsături persistente, Modificări persistente ale tranzitului intestinal	Nu	Nu

Participant_ID	B1	B2	B3	B4	B5
P126	Locul 2	30 ani	Sângerare rectală / sânge în scaun, Scădere bruscă și inexplicabilă în greutate, Modificări persistente ale tranzitului intestinal	Nu	Nu
P127	Locul 2	50 ani	Sângerare rectală / sânge în scaun, Scădere bruscă și inexplicabilă în greutate, Modificări persistente ale tranzitului intestinal	Nu	Nu
P128	Locul 4	40 ani	Sângerare rectală / sânge în scaun, Scădere bruscă și inexplicabilă în greutate, Modificări persistente ale tranzitului intestinal	Mi-a fost recomandat să particip	Nu
P129	Locul 3	40 ani	Sângerare rectală / sânge în scaun, Scădere bruscă și inexplicabilă în greutate, Modificări persistente ale tranzitului intestinal	Nu	Nu
P130	Nu știu	40 ani	Sângerare rectală / sânge în scaun, Scădere bruscă și inexplicabilă în greutate, Modificări persistente ale tranzitului intestinal	Nu	Nu
P131	Locul 2	40 ani	Sângerare rectală / sânge în scaun, Scădere bruscă și inexplicabilă în greutate, Modificări persistente ale tranzitului intestinal	Nu	Nu
P132	Nu știu	Nu știu	Sângerare rectală / sânge în scaun, Modificări persistente ale tranzitului intestinal	Nu	Nu
P133	Locul 1	50 ani	Sângerare rectală / sânge în scaun, Modificări persistente ale tranzitului intestinal	Mi-a fost recomandat să particip	Da, colonoscopie
P134	Locul 3	40 ani	Sângerare rectală / sânge în scaun, Scădere bruscă și inexplicabilă în greutate, Modificări persistente ale tranzitului intestinal	Nu	Nu
P135	Locul 3	50 ani	Sângerare rectală / sânge în scaun, Scădere bruscă și inexplicabilă în greutate, Modificări persistente ale tranzitului intestinal	Nu	Nu
P136	Locul 3	50 ani	Sângerare rectală / sânge în scaun, Scădere bruscă și inexplicabilă în greutate, Modificări persistente ale tranzitului intestinal	Da, dar am întâmpinat dificultăți în accesare	Nu
P137	Locul 4	40 ani	Sângerare rectală / sânge în scaun, Scădere bruscă și inexplicabilă în greutate, Arsuri gastrice frecvente	Nu	Nu
P138	Locul 2	30 ani	Sângerare rectală / sânge în scaun, Scădere bruscă și inexplicabilă în greutate, Greață / vărsături persistente, Modificări persistente ale tranzitului intestinal, Dureri articulare	Nu	Nu
P139	Nu știu	50 ani	Sângerare rectală / sânge în scaun, Scădere bruscă și inexplicabilă în greutate, Greață / vărsături persistente	Nu	Da, colonoscopie
P140	Nu știu	Nu știu	Nu știu	Nu	Nu
P141	Locul 2	30 ani	Sângerare rectală / sânge în scaun, Scădere bruscă și inexplicabilă în greutate, Greață / vărsături persistente, Modificări persistente ale tranzitului intestinal	Da, dar am întâmpinat dificultăți în accesare	Nu
P142	Locul 3	40 ani	Sângerare rectală / sânge în scaun, Scădere bruscă și inexplicabilă în greutate	Nu	Nu
P143	Locul 4	40 ani	Sângerare rectală / sânge în scaun, Scădere bruscă și inexplicabilă în greutate, Greață / vărsături persistente, Modificări persistente ale tranzitului intestinal	Mi-a fost recomandat să particip	Nu
P144	Locul 3	50 ani	Sângerare rectală / sânge în scaun, Scădere bruscă și inexplicabilă în greutate, Greață / vărsături persistente, Modificări persistente ale tranzitului intestinal	Nu	Nu
P145	Locul 3	50 ani	Sângerare rectală / sânge în scaun, Scădere bruscă și inexplicabilă în greutate	Da, am participat	Nu
P146	Locul 3	30 ani	Sângerare rectală / sânge în scaun, Scădere bruscă și inexplicabilă în greutate, Greață / vărsături persistente, Modificări persistente ale tranzitului intestinal	Da, am participat	Nu
P147	Locul 2	40 ani	Sângerare rectală / sânge în scaun, Scădere bruscă și inexplicabilă în greutate, Greață / vărsături persistente, Arsuri gastrice frecvente, Modificări persistente ale tranzitului intestinal, Sete excesivă, Dureri articulare	Nu	Nu
P148	Nu știu	40 ani	Sângerare rectală / sânge în scaun, Scădere bruscă și inexplicabilă în greutate, Modificări persistente ale tranzitului intestinal	Nu	Da, FIT
P149	Locul 2	40 ani	Sângerare rectală / sânge în scaun, Scădere bruscă și inexplicabilă în greutate, Modificări persistente ale tranzitului intestinal	Mi-a fost recomandat să particip	Nu
P150	Locul 2	50 ani	Sângerare rectală / sânge în scaun, Scădere bruscă și inexplicabilă în greutate, Greață / vărsături persistente, Modificări persistente ale tranzitului intestinal	Nu	Nu
P151	Între locurile 5–10	Nu știu	Sângerare rectală / sânge în scaun, Greață / vărsături persistente, Modificări persistente ale tranzitului intestinal	Nu	Nu
P152	Locul 4	50 ani	Sângerare rectală / sânge în scaun, Scădere bruscă și inexplicabilă în greutate, Modificări persistente ale tranzitului intestinal	Nu	Nu
P153	Nu știu	30 ani	Sângerare rectală / sânge în scaun, Scădere bruscă și inexplicabilă în greutate, Modificări persistente ale tranzitului intestinal, Căderea părului	Nu	Nu
P154	Locul 4	Nu știu	Sângerare rectală / sânge în scaun, Scădere bruscă și inexplicabilă în greutate, Greață / vărsături persistente, Modificări persistente ale tranzitului intestinal	Nu	Nu
P155	Locul 3	50 ani	Sângerare rectală / sânge în scaun, Scădere bruscă și inexplicabilă în greutate, Modificări persistente ale tranzitului intestinal	Nu	Nu
P156	Între locurile 5–10	40 ani	Sângerare rectală / sânge în scaun, Scădere bruscă și inexplicabilă în greutate, Greață / vărsături persistente, Nu știu	Mi-a fost recomandat să particip	Nu
P157	Locul 2	30 ani	Sângerare rectală / sânge în scaun, Modificări persistente ale tranzitului intestinal, Sete excesivă	Nu	Nu
P158	Locul 1	40 ani	Nu știu	Nu	Nu
P159	Locul 1	40 ani	Sângerare rectală / sânge în scaun, Scădere bruscă și inexplicabilă în greutate, Disfagie (dificultate la înghițire), Modificări persistente ale tranzitului intestinal	Nu	Nu
P160	Locul 2	50 ani	Sângerare rectală / sânge în scaun, Scădere bruscă și inexplicabilă în greutate	Nu	Nu
P161	Locul 2	50 ani	Sângerare rectală / sânge în scaun, Scădere bruscă și inexplicabilă în greutate, Modificări persistente ale tranzitului intestinal	Nu	Nu
P162	Nu știu	Nu știu	Sângerare rectală / sânge în scaun, Nu știu	Nu	Nu
P163	Locul 2	40 ani	Scădere bruscă și inexplicabilă în greutate	Nu	Nu
P164	Locul 3	50 ani	Sângerare rectală / sânge în scaun, Scădere bruscă și inexplicabilă în greutate, Modificări persistente ale tranzitului intestinal	Da, dar am întâmpinat dificultăți în accesare	Nu
P165	Locul 1	50 ani	Sângerare rectală / sânge în scaun, Scădere bruscă și inexplicabilă în greutate, Modificări persistente ale tranzitului intestinal	Nu	Nu
P166	Locul 2	50 ani	Sângerare rectală / sânge în scaun, Scădere bruscă și inexplicabilă în greutate, Greață / vărsături persistente, Modificări persistente ale tranzitului intestinal	Nu	Nu
P167	Locul 3	40 ani	Sângerare rectală / sânge în scaun, Scădere bruscă și inexplicabilă în greutate, Modificări persistente ale tranzitului intestinal	Nu	Nu
P168	Locul 2	Nu știu	Sângerare rectală / sânge în scaun, Modificări persistente ale tranzitului intestinal	Nu	Nu

Participant_ID	B1	B2	B3	B4	B5
P169	Locul 2	30 ani	Sângerare rectală / sânge în scaun, Scădere bruscă și inexplicabilă în greutate, Greață / vărsături persistente, Modificări persistente ale tranzitului intestinal	Nu	Nu
P170	Locul 2	40 ani	Sângerare rectală / sânge în scaun, Scădere bruscă și inexplicabilă în greutate	Nu	Da, colonoscopie
P171	Locul 3	40 ani	Sângerare rectală / sânge în scaun, Scădere bruscă și inexplicabilă în greutate, Modificări persistente ale tranzitului intestinal	Nu	Nu
P172	Locul 2	40 ani	Sângerare rectală / sânge în scaun	Nu	Nu
P173	Între locurile 5–10	50 ani	Sângerare rectală / sânge în scaun, Modificări persistente ale tranzitului intestinal, Nu știu	Nu	Nu
P174	Locul 4	40 ani	Sângerare rectală / sânge în scaun, Scădere bruscă și inexplicabilă în greutate, Modificări persistente ale tranzitului intestinal	Nu	Nu
P175	Locul 1	30 ani	Sângerare rectală / sânge în scaun, Scădere bruscă și inexplicabilă în greutate, Greață / vărsături persistente, Arsuri gastrice frecvente	Mi-a fost recomandat să particip	Nu
P176	Locul 4	50 ani	Sângerare rectală / sânge în scaun, Scădere bruscă și inexplicabilă în greutate, Modificări persistente ale tranzitului intestinal	Nu	Nu
P177	Nu știu	50 ani	Sângerare rectală / sânge în scaun, Scădere bruscă și inexplicabilă în greutate, Greață / vărsături persistente, Arsuri gastrice frecvente, Disfagie (dificultate la înghițire), Modificări persistente ale tranzitului intestinal	Nu	Nu
P178	Locul 3	40 ani	Sângerare rectală / sânge în scaun, Scădere bruscă și inexplicabilă în greutate	Mi-a fost recomandat să particip	Nu
P179	Locul 3	50 ani	Sângerare rectală / sânge în scaun	Nu	Nu
P180	Locul 3	50 ani	Sângerare rectală / sânge în scaun, Scădere bruscă și inexplicabilă în greutate, Modificări persistente ale tranzitului intestinal	Da, am participat	Nu
P181	Nu știu	Nu știu	Nu știu	Nu	Nu
P182	Locul 2	40 ani	Sângerare rectală / sânge în scaun, Scădere bruscă și inexplicabilă în greutate, Modificări persistente ale tranzitului intestinal	Mi-a fost recomandat să particip	Nu
P183	Locul 4	Nu știu	Sângerare rectală / sânge în scaun, Scădere bruscă și inexplicabilă în greutate, Sete excesivă, Căderea părului	Nu	Nu
P184	Locul 4	40 ani	Sângerare rectală / sânge în scaun, Scădere bruscă și inexplicabilă în greutate, Modificări persistente ale tranzitului intestinal	Mi-a fost recomandat să particip	Nu
P185	Locul 3	50 ani	Sângerare rectală / sânge în scaun, Scădere bruscă și inexplicabilă în greutate	Nu	Nu
P186	Locul 2	50 ani	Sângerare rectală / sânge în scaun, Scădere bruscă și inexplicabilă în greutate, Modificări persistente ale tranzitului intestinal	Nu	Nu
P187	Locul 4	Nu știu	Sângerare rectală / sânge în scaun, Scădere bruscă și inexplicabilă în greutate	Nu	Nu
P188	Locul 4	40 ani	Sângerare rectală / sânge în scaun, Scădere bruscă și inexplicabilă în greutate, Greață / vărsături persistente, Modificări persistente ale tranzitului intestinal	Nu	Nu
P189	Locul 3	50 ani	Sângerare rectală / sânge în scaun, Scădere bruscă și inexplicabilă în greutate	Nu	Nu
P190	Locul 2	40 ani	Sângerare rectală / sânge în scaun, Scădere bruscă și inexplicabilă în greutate, Modificări persistente ale tranzitului intestinal	Nu	Nu
P191	Nu știu	Nu știu	Nu știu	Nu	Nu
P192	Locul 3	Nu știu	Nu știu	Nu	Nu
P193	Locul 3	40 ani	Sângerare rectală / sânge în scaun, Scădere bruscă și inexplicabilă în greutate, Modificări persistente ale tranzitului intestinal	Nu	Nu
P194	Locul 3	40 ani	Sângerare rectală / sânge în scaun, Scădere bruscă și inexplicabilă în greutate, Arsuri gastrice frecvente, Modificări persistente ale tranzitului intestinal	Nu	Nu
P195	Locul 2	50 ani	Sângerare rectală / sânge în scaun, Scădere bruscă și inexplicabilă în greutate, Modificări persistente ale tranzitului intestinal	Nu	Nu
P196	Locul 2	40 ani	Sângerare rectală / sânge în scaun, Scădere bruscă și inexplicabilă în greutate, Modificări persistente ale tranzitului intestinal	Nu	Nu
P197	Locul 1	50 ani	Sângerare rectală / sânge în scaun, Scădere bruscă și inexplicabilă în greutate, Modificări persistente ale tranzitului intestinal	Da, dar am întâmpinat dificultăți în accesare	Da, colonoscopie
P198	Nu știu	Nu știu	Nu știu	Nu	Nu
P199	Locul 2	40 ani	Sângerare rectală / sânge în scaun, Scădere bruscă și inexplicabilă în greutate, Modificări persistente ale tranzitului intestinal	Nu	Da, ambele
P200	Locul 3	Nu știu	Sângerare rectală / sânge în scaun, Scădere bruscă și inexplicabilă în greutate, Greață / vărsături persistente, Modificări persistente ale tranzitului intestinal	Nu	Nu
P201	Locul 4	30 ani	Sângerare rectală / sânge în scaun, Scădere bruscă și inexplicabilă în greutate, Greață / vărsături persistente, Arsuri gastrice frecvente, Modificări persistente ale tranzitului intestinal, Căderea părului	Nu	Nu
P202	Locul 2	30 ani	Sângerare rectală / sânge în scaun, Scădere bruscă și inexplicabilă în greutate, Greață / vărsături persistente, Arsuri gastrice frecvente, Modificări persistente ale tranzitului intestinal	Nu	Nu
P203	Nu știu	30 ani	Nu știu	Mi-a fost recomandat să particip	Nu
P204	Locul 2	30 ani	Scădere bruscă și inexplicabilă în greutate, Modificări persistente ale tranzitului intestinal, Sete excesivă, Căderea părului	Nu	Nu
P205	Locul 3	50 ani	Sângerare rectală / sânge în scaun	Nu	Nu
P206	Locul 3	40 ani	Sângerare rectală / sânge în scaun, Scădere bruscă și inexplicabilă în greutate, Arsuri gastrice frecvente, Modificări persistente ale tranzitului intestinal	Nu	Nu
P207	Locul 3	40 ani	Sângerare rectală / sânge în scaun, Scădere bruscă și inexplicabilă în greutate, Greață / vărsături persistente, Arsuri gastrice frecvente, Modificări persistente ale tranzitului intestinal	Nu	Nu
P208	Locul 3	40 ani	Sângerare rectală / sânge în scaun, Scădere bruscă și inexplicabilă în greutate, Greață / vărsături persistente, Modificări persistente ale tranzitului intestinal	Mi-a fost recomandat să particip	Nu
P209	Locul 2	40 ani	Sângerare rectală / sânge în scaun, Scădere bruscă și inexplicabilă în greutate, Greață / vărsături persistente, Modificări persistente ale tranzitului intestinal	Nu	Nu
P210	Locul 4	40 ani	Sângerare rectală / sânge în scaun, Scădere bruscă și inexplicabilă în greutate, Modificări persistente ale tranzitului intestinal	Da, dar am întâmpinat dificultăți în accesare	Nu
P211	Locul 3	40 ani	Sângerare rectală / sânge în scaun, Modificări persistente ale tranzitului intestinal	Nu	Nu
P212	Nu știu	Nu știu	Sângerare rectală / sânge în scaun, Scădere bruscă și inexplicabilă în greutate, Greață / vărsături persistente, Arsuri gastrice frecvente, Modificări persistente ale tranzitului intestinal	Nu	Nu

Participant_ID	B1	B2	B3	B4	B5
P213	Locul 3	40 ani	Sângerare rectală / sânge în scaun, Scădere bruscă și inexplicabilă în greutate, Modificări persistente ale tranzitului intestinal	Nu	Nu
P214	Locul 2	40 ani	Sângerare rectală / sânge în scaun, Scădere bruscă și inexplicabilă în greutate, Greață / vărsături persistente, Modificări persistente ale tranzitului intestinal	Nu	Nu
P215	Nu știu	Nu știu	Nu știu	Nu	Nu
P216	Nu știu	Nu știu	Sângerare rectală / sânge în scaun, Scădere bruscă și inexplicabilă în greutate, Modificări persistente ale tranzitului intestinal, Nu știu	Nu	Nu
P217	Nu știu	Nu știu	Sângerare rectală / sânge în scaun	Nu	Nu
P218	Locul 3	Nu știu	Sângerare rectală / sânge în scaun	Nu	Nu
P219	Nu știu	Nu știu	Sângerare rectală / sânge în scaun, Scădere bruscă și inexplicabilă în greutate, Modificări persistente ale tranzitului intestinal	Nu	Nu
P220	Locul 2	50 ani	Sângerare rectală / sânge în scaun, Scădere bruscă și inexplicabilă în greutate, Greață / vărsături persistente, Modificări persistente ale tranzitului intestinal	Nu	Nu
P221	Nu știu	Nu știu	Sângerare rectală / sânge în scaun, Scădere bruscă și inexplicabilă în greutate, Greață / vărsături persistente, Modificări persistente ale tranzitului intestinal	Nu	Nu
P222	Nu știu	30 ani	Sângerare rectală / sânge în scaun, Scădere bruscă și inexplicabilă în greutate, Greață / vărsături persistente, Modificări persistente ale tranzitului intestinal	Nu	Nu
P223	Locul 2	40 ani	Sângerare rectală / sânge în scaun, Scădere bruscă și inexplicabilă în greutate, Modificări persistente ale tranzitului intestinal	Nu	Nu
P224	Nu știu	Nu știu	Sângerare rectală / sânge în scaun, Scădere bruscă și inexplicabilă în greutate, Arsuri gastrice frecvente, Modificări persistente ale tranzitului intestinal	Nu	Da, FIT
P225	Locul 4	40 ani	Sângerare rectală / sânge în scaun, Scădere bruscă și inexplicabilă în greutate, Greață / vărsături persistente, Arsuri gastrice frecvente, Modificări persistente ale tranzitului intestinal, Căderea părului	Nu	Nu
P226	Locul 2	40 ani	Sângerare rectală / sânge în scaun, Modificări persistente ale tranzitului intestinal	Nu	Nu
P227	Nu știu	40 ani	Sângerare rectală / sânge în scaun, Scădere bruscă și inexplicabilă în greutate, Greață / vărsături persistente, Arsuri gastrice frecvente, Modificări persistente ale tranzitului intestinal	Nu	Nu
P228	Locul 3	40 ani	Sângerare rectală / sânge în scaun, Scădere bruscă și inexplicabilă în greutate, Modificări persistente ale tranzitului intestinal	Mi-a fost recomandat să particip	Nu
P229	Locul 3	40 ani	Sângerare rectală / sânge în scaun, Scădere bruscă și inexplicabilă în greutate	Nu	Nu
P230	Locul 2	40 ani	Sângerare rectală / sânge în scaun, Scădere bruscă și inexplicabilă în greutate, Modificări persistente ale tranzitului intestinal	Nu	Nu
P231	Locul 4	30 ani	Sângerare rectală / sânge în scaun, Scădere bruscă și inexplicabilă în greutate, Modificări persistente ale tranzitului intestinal	Nu	Nu
P232	Locul 2	40 ani	Sângerare rectală / sânge în scaun, Scădere bruscă și inexplicabilă în greutate, Modificări persistente ale tranzitului intestinal	Nu	Nu
P233	Locul 3	50 ani	Sângerare rectală / sânge în scaun	Nu	Nu
P234	Nu știu	Nu știu	Modificări persistente ale tranzitului intestinal	Nu	Nu
P235	Între locurile 5–10	40 ani	Sângerare rectală / sânge în scaun, Scădere bruscă și inexplicabilă în greutate, Greață / vărsături persistente, Modificări persistente ale tranzitului intestinal	Nu	Nu
P236	Locul 3	40 ani	Sângerare rectală / sânge în scaun, Scădere bruscă și inexplicabilă în greutate, Modificări persistente ale tranzitului intestinal	Nu	Nu
P237	Locul 2	30 ani	Scădere bruscă și inexplicabilă în greutate, Greață / vărsături persistente, Modificări persistente ale tranzitului intestinal	Nu	Nu
P238	Între locurile 5–10	50 ani	Sângerare rectală / sânge în scaun, Scădere bruscă și inexplicabilă în greutate, Arsuri gastrice frecvente, Modificări persistente ale tranzitului intestinal	Nu	Nu
P239	Locul 3	40 ani	Sângerare rectală / sânge în scaun, Scădere bruscă și inexplicabilă în greutate, Modificări persistente ale tranzitului intestinal	Nu	Nu
P240	Nu știu	Nu știu	Sângerare rectală / sânge în scaun, Scădere bruscă și inexplicabilă în greutate, Greață / vărsături persistente, Arsuri gastrice frecvente	Nu	Nu
P241	Locul 2	50 ani	Sângerare rectală / sânge în scaun, Scădere bruscă și inexplicabilă în greutate, Greață / vărsături persistente, Modificări persistente ale tranzitului intestinal	Nu	Nu
P242	Locul 3	50 ani	Sângerare rectală / sânge în scaun, Scădere bruscă și inexplicabilă în greutate, Modificări persistente ale tranzitului intestinal	Nu	Nu
P243	Locul 1	40 ani	Sângerare rectală / sânge în scaun, Scădere bruscă și inexplicabilă în greutate, Greață / vărsături persistente, Arsuri gastrice frecvente, Modificări persistente ale tranzitului intestinal	Nu	Nu
P244	Nu știu	30 ani	Sângerare rectală / sânge în scaun, Scădere bruscă și inexplicabilă în greutate, Greață / vărsături persistente, Modificări persistente ale tranzitului intestinal	Nu	Nu
P245	Locul 1	40 ani	Sângerare rectală / sânge în scaun	Mi-a fost recomandat să particip	Nu
P246	Locul 2	50 ani	Sângerare rectală / sânge în scaun, Scădere bruscă și inexplicabilă în greutate, Modificări persistente ale tranzitului intestinal	Nu	Nu
P247	Locul 4	50 ani	Sângerare rectală / sânge în scaun, Scădere bruscă și inexplicabilă în greutate, Modificări persistente ale tranzitului intestinal	Nu	Nu
P248	Nu știu	Nu știu	Nu știu	Nu	Nu
P249	Nu știu	40 ani	Sângerare rectală / sânge în scaun, Scădere bruscă și inexplicabilă în greutate	Nu	Nu
P250	Locul 2	50 ani	Sângerare rectală / sânge în scaun, Scădere bruscă și inexplicabilă în greutate, Modificări persistente ale tranzitului intestinal	Nu	Nu
P251	Locul 1	40 ani	Sângerare rectală / sânge în scaun, Scădere bruscă și inexplicabilă în greutate, Modificări persistente ale tranzitului intestinal	Nu	Nu
P252	Nu știu	40 ani	Arsuri gastrice frecvente	Nu	Nu
P253	Locul 3	50 ani	Sângerare rectală / sânge în scaun, Scădere bruscă și inexplicabilă în greutate, Modificări persistente ale tranzitului intestinal	Da, am participat	Nu
P254	Locul 3	30 ani	Sângerare rectală / sânge în scaun, Modificări persistente ale tranzitului intestinal	Da, dar am întâmpinat dificultăți în accesare	Nu
P255	Locul 3	30 ani	Sângerare rectală / sânge în scaun, Scădere bruscă și inexplicabilă în greutate, Greață / vărsături persistente, Modificări persistente ale tranzitului intestinal	Nu	Nu

Participant_ID	B1	B2	B3	B4	B5
P256	Nu știu	40 ani	Sângerare rectală / sânge în scaun, Scădere bruscă și inexplicabilă în greutate, Greață / vărsături persistente, Modificări persistente ale tranzitului intestinal	Nu	Da, FIT
P257	Locul 3	50 ani	Sângerare rectală / sânge în scaun, Scădere bruscă și inexplicabilă în greutate, Modificări persistente ale tranzitului intestinal	Nu	Nu
P258	Locul 1	40 ani	Sângerare rectală / sânge în scaun	Mi-a fost recomandat să particip	Nu
P259	Locul 3	50 ani	Sângerare rectală / sânge în scaun, Scădere bruscă și inexplicabilă în greutate	Nu	Nu
P260	Locul 3	50 ani	Sângerare rectală / sânge în scaun, Scădere bruscă și inexplicabilă în greutate, Modificări persistente ale tranzitului intestinal	Mi-a fost recomandat să particip	Da, FIT
P261	Locul 3	40 ani	Sângerare rectală / sânge în scaun, Scădere bruscă și inexplicabilă în greutate, Modificări persistente ale tranzitului intestinal	Nu	Nu
P262	Locul 3	40 ani	Sângerare rectală / sânge în scaun, Scădere bruscă și inexplicabilă în greutate, Modificări persistente ale tranzitului intestinal	Nu	Nu
P263	Nu știu	Nu știu	Nu știu	Nu	Nu
P264	Locul 2	40 ani	Sângerare rectală / sânge în scaun, Scădere bruscă și inexplicabilă în greutate, Greață / vărsături persistente, Arsuri gastrice frecvente, Disfagie (dificultate la înghițire), Modificări persistente ale tranzitului intestinal, Sete excesivă, Căderea părului, Dureri articulare	Nu	Nu
P265	Locul 1	30 ani	Sângerare rectală / sânge în scaun, Scădere bruscă și inexplicabilă în greutate, Disfagie (dificultate la înghițire)	Mi-a fost recomandat să particip	Da, colonoscopie
P266	Locul 3	50 ani	Sângerare rectală / sânge în scaun, Scădere bruscă și inexplicabilă în greutate	Nu	Nu
P267	Între locurile 5–10	65 ani	Sângerare rectală / sânge în scaun, Scădere bruscă și inexplicabilă în greutate, Modificări persistente ale tranzitului intestinal, Căderea părului	Nu	Nu
P268	Nu știu	50 ani	Sângerare rectală / sânge în scaun, Scădere bruscă și inexplicabilă în greutate, Modificări persistente ale tranzitului intestinal	Nu	Nu
P269	Locul 2	40 ani	Sângerare rectală / sânge în scaun, Scădere bruscă și inexplicabilă în greutate, Modificări persistente ale tranzitului intestinal	Nu	Da, colonoscopie
P270	Locul 3	50 ani	Sângerare rectală / sânge în scaun, Scădere bruscă și inexplicabilă în greutate, Modificări persistente ale tranzitului intestinal	Da, am participat	Nu
P271	Locul 3	40 ani	Sângerare rectală / sânge în scaun, Scădere bruscă și inexplicabilă în greutate, Arsuri gastrice frecvente, Modificări persistente ale tranzitului intestinal	Nu	Nu
P272	Între locurile 5–10	50 ani	Scădere bruscă și inexplicabilă în greutate	Nu	Nu
P273	Locul 3	40 ani	Sângerare rectală / sânge în scaun, Scădere bruscă și inexplicabilă în greutate, Modificări persistente ale tranzitului intestinal	Da, dar am întâmpinat dificultăți în accesare	Nu
P274	Nu știu	50 ani	Sângerare rectală / sânge în scaun, Scădere bruscă și inexplicabilă în greutate, Modificări persistente ale tranzitului intestinal	Nu	Nu
P275	Locul 2	50 ani	Sângerare rectală / sânge în scaun, Scădere bruscă și inexplicabilă în greutate, Greață / vărsături persistente, Modificări persistente ale tranzitului intestinal, Sete excesivă	Nu	Nu
P276	Nu știu	Nu știu	Sângerare rectală / sânge în scaun, Modificări persistente ale tranzitului intestinal, Nu știu	Nu	Da, colonoscopie
P277	Locul 3	40 ani	Sângerare rectală / sânge în scaun, Scădere bruscă și inexplicabilă în greutate, Modificări persistente ale tranzitului intestinal	Da, am participat	Da, FIT
P278	Locul 3	50 ani	Sângerare rectală / sânge în scaun, Scădere bruscă și inexplicabilă în greutate, Modificări persistente ale tranzitului intestinal	Mi-a fost recomandat să particip	Da, colonoscopie
P279	Nu știu	50 ani	Scădere bruscă și inexplicabilă în greutate, Modificări persistente ale tranzitului intestinal	Nu	Nu
P280	Locul 2	40 ani	Sângerare rectală / sânge în scaun, Scădere bruscă și inexplicabilă în greutate, Modificări persistente ale tranzitului intestinal	Da, am participat	Da, colonoscopie
P281	Nu știu	30 ani	Sângerare rectală / sânge în scaun, Modificări persistente ale tranzitului intestinal	Nu	Nu
P282	Locul 3	50 ani	Sângerare rectală / sânge în scaun, Arsuri gastrice frecvente, Modificări persistente ale tranzitului intestinal	Nu	Nu
P283	Locul 3	30 ani	Sângerare rectală / sânge în scaun, Scădere bruscă și inexplicabilă în greutate, Modificări persistente ale tranzitului intestinal	Da, am participat	Da, ambele
P284	Nu știu	50 ani	Sângerare rectală / sânge în scaun	Da, am participat	Nu
P285	Nu știu	50 ani	Sângerare rectală / sânge în scaun	Da, am participat	Da, FIT
P286	Locul 3	40 ani	Sângerare rectală / sânge în scaun, Scădere bruscă și inexplicabilă în greutate, Modificări persistente ale tranzitului intestinal	Nu	Nu
P287	Locul 3	50 ani	Sângerare rectală / sânge în scaun, Scădere bruscă și inexplicabilă în greutate	Nu	Da, ambele
P288	Locul 3	50 ani	Sângerare rectală / sânge în scaun, Scădere bruscă și inexplicabilă în greutate, Modificări persistente ale tranzitului intestinal	Nu	Nu
P289	Locul 2	30 ani	Sângerare rectală / sânge în scaun, Modificări persistente ale tranzitului intestinal	Da, am participat	Da, ambele
P290	Locul 2	50 ani	Sângerare rectală / sânge în scaun, Scădere bruscă și inexplicabilă în greutate, Greață / vărsături persistente, Arsuri gastrice frecvente	Nu	Nu
P291	Locul 1	50 ani	Sângerare rectală / sânge în scaun	Da, am participat	Nu
P292	Locul 1	40 ani	Modificări persistente ale tranzitului intestinal	Mi-a fost recomandat să particip	Nu
P293	Locul 3	50 ani	Sângerare rectală / sânge în scaun, Greață / vărsături persistente, Modificări persistente ale tranzitului intestinal, Nu știu	Nu	Nu
P294	Locul 4	50 ani	Sângerare rectală / sânge în scaun, Scădere bruscă și inexplicabilă în greutate, Greață / vărsături persistente, Arsuri gastrice frecvente, Sete excesivă	Nu	Nu
P295	Nu știu	50 ani	Sângerare rectală / sânge în scaun, Modificări persistente ale tranzitului intestinal	Nu	Nu
P296	Locul 3	65 ani	Sângerare rectală / sânge în scaun	Nu	Nu
P297	Locul 1	50 ani	Sângerare rectală / sânge în scaun, Scădere bruscă și inexplicabilă în greutate, Arsuri gastrice frecvente, Disfagie (dificultate la înghițire), Modificări persistente ale tranzitului intestinal	Nu	Nu
P298	Locul 3	40 ani	Sângerare rectală / sânge în scaun, Scădere bruscă și inexplicabilă în greutate, Greață / vărsături persistente, Arsuri gastrice frecvente, Modificări persistente ale tranzitului intestinal	Nu	Nu
P299	Locul 1	30 ani	Sângerare rectală / sânge în scaun, Scădere bruscă și inexplicabilă în greutate, Greață / vărsături persistente, Arsuri gastrice frecvente, Disfagie (dificultate la înghițire), Modificări persistente ale tranzitului intestinal, Sete excesivă, Căderea părului, Dureri articulare	Nu	Nu
P300	Între locurile 5–10	40 ani	Sângerare rectală / sânge în scaun, Scădere bruscă și inexplicabilă în greutate, Modificări persistente ale tranzitului intestinal	Mi-a fost recomandat să particip	Da, colonoscopie

Participant_ ID	B1	B2	B3	B4	B5
P301	Nu știu	Nu știu	Disfagie (dificultate la înghițire), Sete excesivă, Căderea părului	Nu	Da, colonoscopie

Panel C — Knowledge of nutritional risk and protective factors (C1–C9 composite; C10 misconception probe)

Participant_ID	C1	C2	C3	C4	C5	C6	C7	C8	C9	C10
P001	Adevărat	Nu știu	Adevărat	Fals	Adevărat	Fals	Adevărat	Adevărat	Nu știu	Fals
P002	Adevărat	Adevărat	Fals	Adevărat	Adevărat	Adevărat	Adevărat	Adevărat	Adevărat	Adevărat
P003	Fals	Nu știu	Adevărat	Fals	Fals	Nu știu	Adevărat	Adevărat	Nu știu	Nu știu
P004	Adevărat	Adevărat	Nu știu	Adevărat	Adevărat	Adevărat	Adevărat	Adevărat	Fals	Fals
P005	Adevărat	Adevărat	Adevărat	Adevărat	Adevărat	Adevărat	Adevărat	Adevărat	Adevărat	Adevărat
P006	Adevărat	Nu știu	Fals	Nu știu	Adevărat	Adevărat	Adevărat	Adevărat	Adevărat	Nu știu
P007	Adevărat	Fals	Adevărat	Nu știu	Nu știu	Nu știu	Adevărat	Fals	Nu știu	Fals
P008	Fals	Nu știu	Adevărat	Fals	Nu știu	Adevărat	Nu știu	Adevărat	Nu știu	Fals
P009	Adevărat	Nu știu	Fals	Fals	Nu știu	Adevărat	Adevărat	Adevărat	Adevărat	Fals
P010	Adevărat	Fals	Adevărat	Fals	Adevărat	Fals	Adevărat	Fals	Adevărat	Fals
P011	Nu știu	Fals	Fals	Nu știu	Nu știu	Nu știu	Adevărat	Adevărat	Adevărat	Fals
P012	Nu știu	Nu știu	Fals	Adevărat	Adevărat	Adevărat	Adevărat	Adevărat	Nu știu	Nu știu
P013	Nu știu	Fals	Adevărat	Nu știu	Fals	Fals	Adevărat	Nu știu	Adevărat	Nu știu
P014	Adevărat	Nu știu	Nu știu	Adevărat	Adevărat	Nu știu	Adevărat	Adevărat	Adevărat	Nu știu
P015	Fals	Fals	Fals	Nu știu	Nu știu	Adevărat	Fals	Adevărat	Adevărat	Fals
P016	Adevărat	Adevărat	Nu știu	Nu știu	Nu știu	Adevărat	Adevărat	Adevărat	Nu știu	Nu știu
P017	Fals	Adevărat	Fals	Adevărat	Adevărat	Adevărat	Adevărat	Adevărat	Nu știu	Fals
P018	Adevărat	Nu știu	Fals	Adevărat	Adevărat	Nu știu	Fals	Adevărat	Nu știu	Nu știu
P019	Adevărat	Adevărat	Fals	Adevărat	Adevărat	Adevărat	Adevărat	Adevărat	Adevărat	Adevărat
P020	Adevărat	Nu știu	Nu știu	Nu știu	Nu știu	Adevărat	Adevărat	Adevărat	Nu știu	Nu știu
P021	Adevărat	Adevărat	Adevărat	Adevărat	Adevărat	Adevărat	Adevărat	Adevărat	Adevărat	Nu știu
P022	Nu știu	Adevărat	Fals	Adevărat	Nu știu	Adevărat	Fals	Nu știu	Nu știu	Nu știu
P023	Nu știu	Fals	Adevărat	Adevărat	Adevărat	Adevărat	Adevărat	Adevărat	Fals	Fals
P024	Adevărat	Adevărat	Nu știu	Adevărat	Nu știu	Nu știu	Fals	Adevărat	Nu știu	Nu știu
P025	Adevărat	Adevărat	Fals	Nu știu	Adevărat	Adevărat	Fals	Adevărat	Nu știu	Nu știu
P026	Adevărat	Adevărat	Adevărat	Adevărat	Adevărat	Adevărat	Adevărat	Adevărat	Adevărat	Fals
P027	Adevărat	Adevărat	Adevărat	Adevărat	Adevărat	Adevărat	Adevărat	Adevărat	Adevărat	Fals
P028	Adevărat	Nu știu	Adevărat	Adevărat	Adevărat	Nu știu	Adevărat	Nu știu	Nu știu	Nu știu
P029	Adevărat	Nu știu	Nu știu	Adevărat	Nu știu	Adevărat	Nu știu	Nu știu	Nu știu	Nu știu
P030	Nu știu	Nu știu	Adevărat	Adevărat	Adevărat	Adevărat	Adevărat	Adevărat	Nu știu	Nu știu
P031	Fals	Nu știu	Adevărat	Nu știu	Nu știu	Nu știu	Adevărat	Nu știu	Fals	Fals
P032	Adevărat	Nu știu	Nu știu	Nu știu	Nu știu	Nu știu	Nu știu	Nu știu	Adevărat	Nu știu
P033	Adevărat	Fals	Fals	Fals	Adevărat	Nu știu	Adevărat	Adevărat	Fals	Fals
P034	Nu știu	Nu știu	Nu știu	Nu știu	Nu știu	Nu știu	Nu știu	Nu știu	Nu știu	Nu știu
P035	Fals	Adevărat	Fals	Adevărat	Adevărat	Adevărat	Fals	Fals	Nu știu	Fals
P036	Adevărat	Fals	Adevărat	Adevărat	Adevărat	Adevărat	Adevărat	Adevărat	Nu știu	Nu știu
P037	Adevărat	Fals	Fals	Adevărat	Adevărat	Adevărat	Adevărat	Adevărat	Adevărat	Adevărat
P038	Adevărat	Nu știu	Adevărat	Nu știu	Nu știu	Adevărat	Adevărat	Adevărat	Adevărat	Nu știu
P039	Adevărat	Fals	Adevărat	Nu știu	Adevărat	Adevărat	Adevărat	Adevărat	Adevărat	Fals
P040	Adevărat	Adevărat	Adevărat	Adevărat	Adevărat	Adevărat	Adevărat	Adevărat	Adevărat	Adevărat
P041	Adevărat	Nu știu	Adevărat	Adevărat	Adevărat	Nu știu	Nu știu	Nu știu	Fals	Nu știu
P042	Nu știu	Nu știu	Nu știu	Adevărat	Adevărat	Nu știu	Adevărat	Adevărat	Adevărat	Nu știu
P043	Adevărat	Adevărat	Adevărat	Adevărat	Adevărat	Adevărat	Adevărat	Nu știu	Nu știu	Nu știu
P044	Fals	Adevărat	Nu știu	Adevărat	Fals	Adevărat	Adevărat	Adevărat	Fals	Fals
P045	Adevărat	Adevărat	Adevărat	Adevărat	Adevărat	Adevărat	Adevărat	Adevărat	Adevărat	Nu știu
P046	Adevărat	Adevărat	Adevărat	Nu știu	Nu știu	Adevărat	Adevărat	Adevărat	Nu știu	Nu știu
P047	Adevărat	Adevărat	Adevărat	Adevărat	Adevărat	Adevărat	Adevărat	Adevărat	Adevărat	Nu știu
P048	Adevărat	Adevărat	Adevărat	Adevărat	Nu știu	Adevărat	Adevărat	Adevărat	Nu știu	Nu știu
P049	Adevărat	Nu știu	Adevărat	Adevărat	Adevărat	Nu știu	Adevărat	Adevărat	Nu știu	Nu știu
P050	Adevărat	Nu știu	Adevărat	Adevărat	Adevărat	Nu știu	Adevărat	Nu știu	Nu știu	Fals
P051	Adevărat	Adevărat	Adevărat	Nu știu	Adevărat	Nu știu	Nu știu	Adevărat	Nu știu	Fals
P052	Adevărat	Adevărat	Fals	Fals	Adevărat	Adevărat	Adevărat	Adevărat	Adevărat	Fals
P053	Adevărat	Adevărat	Adevărat	Adevărat	Adevărat	Adevărat	Adevărat	Adevărat	Adevărat	Adevărat
P054	Nu știu	Nu știu	Nu știu	Nu știu	Nu știu	Nu știu	Nu știu	Nu știu	Nu știu	Nu știu
P055	Fals	Adevărat	Nu știu	Fals	Adevărat	Adevărat	Fals	Fals	Fals	Fals
P056	Adevărat	Nu știu	Adevărat	Adevărat	Fals	Nu știu	Adevărat	Nu știu	Nu știu	Nu știu
P057	Adevărat	Nu știu	Fals	Adevărat	Adevărat	Adevărat	Fals	Fals	Fals	Fals
P058	Adevărat	Nu știu	Fals	Nu știu	Nu știu	Adevărat	Adevărat	Adevărat	Adevărat	Fals
P059	Fals	Adevărat	Fals	Adevărat	Adevărat	Adevărat	Adevărat	Adevărat	Adevărat	Fals
P060	Nu știu	Adevărat	Fals	Adevărat	Adevărat	Adevărat	Adevărat	Nu știu	Adevărat	Nu știu
P061	Adevărat	Adevărat	Adevărat	Adevărat	Adevărat	Nu știu	Adevărat	Adevărat	Nu știu	Nu știu
P062	Adevărat	Nu știu	Fals	Adevărat	Adevărat	Adevărat	Nu știu	Fals	Nu știu	Fals
P063	Adevărat	Adevărat	Adevărat	Adevărat	Adevărat	Adevărat	Adevărat	Adevărat	Adevărat	Adevărat
P064	Adevărat	Adevărat	Fals	Adevărat	Fals	Fals	Adevărat	Adevărat	Adevărat	Fals
P065	Adevărat	Fals	Fals	Adevărat	Adevărat	Adevărat	Adevărat	Adevărat	Adevărat	Fals
P066	Adevărat	Nu știu	Adevărat	Adevărat	Nu știu	Nu știu	Adevărat	Adevărat	Nu știu	Fals
P067	Adevărat	Fals	Adevărat	Fals	Adevărat	Adevărat	Nu știu	Adevărat	Nu știu	Nu știu
P068	Adevărat	Adevărat	Adevărat	Adevărat	Adevărat	Adevărat	Nu știu	Adevărat	Nu știu	Nu știu
P069	Adevărat	Adevărat	Adevărat	Adevărat	Adevărat	Adevărat	Adevărat	Adevărat	Adevărat	Nu știu
P070	Adevărat	Adevărat	Adevărat	Nu știu	Adevărat	Adevărat	Adevărat	Nu știu	Nu știu	Fals
P071	Nu știu	Nu știu	Adevărat	Nu știu	Nu știu	Adevărat	Nu știu	Adevărat	Adevărat	Nu știu
P072	Adevărat	Adevărat	Adevărat	Fals	Adevărat	Adevărat	Adevărat	Adevărat	Adevărat	Nu știu
P073	Adevărat	Nu știu	Fals	Nu știu	Adevărat	Nu știu	Adevărat	Adevărat	Adevărat	Nu știu
P074	Nu știu	Fals	Fals	Adevărat	Adevărat	Nu știu	Adevărat	Adevărat	Adevărat	Fals
P075	Adevărat	Adevărat	Adevărat	Adevărat	Adevărat	Fals	Fals	Adevărat	Adevărat	Nu știu
P076	Adevărat	Fals	Adevărat	Adevărat	Fals	Adevărat	Adevărat	Adevărat	Fals	Fals
P077	Adevărat	Nu știu	Adevărat	Adevărat	Nu știu	Adevărat	Adevărat	Adevărat	Adevărat	Nu știu
P078	Adevărat	Adevărat	Adevărat	Fals	Fals	Adevărat	Adevărat	Adevărat	Adevărat	Fals
P079	Fals	Adevărat	Adevărat	Adevărat	Adevărat	Adevărat	Nu știu	Adevărat	Nu știu	Nu știu
P080	Adevărat	Adevărat	Adevărat	Adevărat	Adevărat	Adevărat	Adevărat	Adevărat	Adevărat	Nu știu

Participant_ID	C1	C2	C3	C4	C5	C6	C7	C8	C9	C10
P081	Adevărat	Adevărat	Fals	Adevărat	Adevărat	Adevărat	Fals	Fals	Fals	Fals
P082	Adevărat	Adevărat	Adevărat	Adevărat	Adevărat	Adevărat	Adevărat	Adevărat	Nu știu	Nu știu
P083	Adevărat	Adevărat	Adevărat	Adevărat	Adevărat	Adevărat	Adevărat	Adevărat	Adevărat	Adevărat
P084	Adevărat	Adevărat	Fals	Adevărat	Adevărat	Adevărat	Fals	Fals	Fals	Fals
P085	Adevărat	Adevărat	Adevărat	Adevărat	Adevărat	Adevărat	Adevărat	Adevărat	Adevărat	Nu știu
P086	Adevărat	Nu știu	Adevărat	Adevărat	Adevărat	Adevărat	Adevărat	Adevărat	Adevărat	Fals
P087	Adevărat	Fals	Fals	Fals	Adevărat	Adevărat	Adevărat	Nu știu	Nu știu	Fals
P088	Adevărat	Fals	Fals	Adevărat	Adevărat	Adevărat	Adevărat	Adevărat	Adevărat	Adevărat
P089	Fals	Fals	Fals	Adevărat	Adevărat	Adevărat	Adevărat	Adevărat	Fals	Nu știu
P090	Adevărat	Fals	Adevărat	Fals	Adevărat	Adevărat	Adevărat	Adevărat	Fals	Fals
P091	Adevărat	Fals	Adevărat	Adevărat	Adevărat	Adevărat	Adevărat	Adevărat	Nu știu	Nu știu
P092	Adevărat	Adevărat	Adevărat	Adevărat	Adevărat	Adevărat	Adevărat	Adevărat	Nu știu	Nu știu
P093	Adevărat	Adevărat	Fals	Adevărat	Adevărat	Adevărat	Fals	Adevărat	Fals	Fals
P094	Nu știu	Fals	Fals	Adevărat	Adevărat	Nu știu	Adevărat	Adevărat	Adevărat	Fals
P095	Nu știu	Nu știu	Nu știu	Adevărat	Adevărat	Nu știu	Nu știu	Nu știu	Nu știu	Adevărat
P096	Adevărat	Adevărat	Adevărat	Fals	Adevărat	Adevărat	Adevărat	Adevărat	Fals	Fals
P097	Nu știu	Adevărat	Fals	Adevărat	Adevărat	Adevărat	Fals	Adevărat	Adevărat	Nu știu
P098	Adevărat	Adevărat	Fals	Adevărat	Adevărat	Adevărat	Nu știu	Fals	Nu știu	Nu știu
P099	Adevărat	Adevărat	Adevărat	Adevărat	Adevărat	Adevărat	Adevărat	Adevărat	Adevărat	Adevărat
P100	Adevărat	Adevărat	Adevărat	Adevărat	Adevărat	Adevărat	Adevărat	Adevărat	Fals	Fals
P101	Adevărat	Fals	Fals	Adevărat	Adevărat	Adevărat	Adevărat	Adevărat	Fals	Nu știu
P102	Adevărat	Adevărat	Adevărat	Adevărat	Adevărat	Adevărat	Adevărat	Adevărat	Adevărat	Fals
P103	Adevărat	Fals	Adevărat	Adevărat	Adevărat	Adevărat	Adevărat	Adevărat	Adevărat	Nu știu
P104	Adevărat	Adevărat	Adevărat	Adevărat	Adevărat	Adevărat	Adevărat	Adevărat	Nu știu	Fals
P105	Nu știu	Nu știu	Nu știu	Nu știu	Nu știu	Nu știu	Nu știu	Nu știu	Nu știu	Nu știu
P106	Nu știu	Fals	Fals	Fals	Fals	Nu știu	Adevărat	Adevărat	Nu știu	Nu știu
P107	Adevărat	Adevărat	Adevărat	Adevărat	Adevărat	Adevărat	Adevărat	Adevărat	Adevărat	Fals
P108	Adevărat	Adevărat	Adevărat	Nu știu	Nu știu	Nu știu	Adevărat	Adevărat	Fals	Nu știu
P109	Adevărat	Fals	Adevărat	Adevărat	Adevărat	Adevărat	Adevărat	Adevărat	Nu știu	Fals
P110	Nu știu	Nu știu	Adevărat	Adevărat	Adevărat	Adevărat	Adevărat	Adevărat	Adevărat	Fals
P111	Adevărat	Adevărat	Adevărat	Adevărat	Adevărat	Adevărat	Adevărat	Adevărat	Adevărat	Fals
P112	Adevărat	Fals	Fals	Nu știu	Adevărat	Adevărat	Adevărat	Adevărat	Nu știu	Nu știu
P113	Adevărat	Adevărat	Adevărat	Adevărat	Adevărat	Nu știu	Adevărat	Adevărat	Adevărat	Nu știu
P114	Fals	Adevărat	Adevărat	Adevărat	Adevărat	Adevărat	Fals	Adevărat	Adevărat	Fals
P115	Adevărat	Adevărat	Fals	Adevărat	Nu știu	Adevărat	Adevărat	Adevărat	Adevărat	Nu știu
P116	Adevărat	Nu știu	Fals	Adevărat	Adevărat	Nu știu	Adevărat	Nu știu	Nu știu	Nu știu
P117	Adevărat	Fals	Nu știu	Adevărat	Adevărat	Adevărat	Adevărat	Adevărat	Adevărat	Fals
P118	Adevărat	Adevărat	Adevărat	Adevărat	Adevărat	Adevărat	Adevărat	Nu știu	Fals	Nu știu
P119	Adevărat	Adevărat	Adevărat	Adevărat	Adevărat	Adevărat	Adevărat	Adevărat	Nu știu	Nu știu
P120	Adevărat	Adevărat	Adevărat	Adevărat	Adevărat	Adevărat	Adevărat	Adevărat	Adevărat	Nu știu
P121	Adevărat	Adevărat	Adevărat	Nu știu	Adevărat	Adevărat	Adevărat	Adevărat	Nu știu	Nu știu
P122	Adevărat	Adevărat	Adevărat	Adevărat	Adevărat	Nu știu	Adevărat	Adevărat	Fals	Nu știu
P123	Adevărat	Fals	Adevărat	Nu știu	Nu știu	Nu știu	Nu știu	Nu știu	Nu știu	Fals
P124	Nu știu	Nu știu	Adevărat	Nu știu	Adevărat	Nu știu	Adevărat	Adevărat	Nu știu	Nu știu
P125	Adevărat	Adevărat	Adevărat	Adevărat	Adevărat	Adevărat	Nu știu	Nu știu	Nu știu	Nu știu
P126	Adevărat	Adevărat	Adevărat	Adevărat	Nu știu	Adevărat	Adevărat	Nu știu	Adevărat	Fals
P127	Nu știu	Adevărat	Adevărat	Adevărat	Adevărat	Adevărat	Fals	Fals	Fals	Fals
P128	Adevărat	Adevărat	Adevărat	Adevărat	Adevărat	Adevărat	Adevărat	Adevărat	Fals	Fals
P129	Adevărat	Adevărat	Adevărat	Adevărat	Nu știu	Fals	Nu știu	Nu știu	Fals	Fals
P130	Adevărat	Fals	Adevărat	Fals	Adevărat	Adevărat	Adevărat	Adevărat	Adevărat	Nu știu
P131	Adevărat	Adevărat	Adevărat	Adevărat	Adevărat	Adevărat	Adevărat	Adevărat	Nu știu	Fals
P132	Nu știu	Nu știu	Nu știu	Nu știu	Nu știu	Nu știu	Nu știu	Nu știu	Nu știu	Nu știu
P133	Adevărat	Adevărat	Fals	Adevărat	Adevărat	Fals	Adevărat	Adevărat	Adevărat	Fals
P134	Adevărat	Adevărat	Adevărat	Adevărat	Adevărat	Adevărat	Adevărat	Adevărat	Adevărat	Fals
P135	Adevărat	Adevărat	Fals	Adevărat	Adevărat	Adevărat	Fals	Adevărat	Adevărat	Fals
P136	Adevărat	Adevărat	Adevărat	Adevărat	Adevărat	Adevărat	Adevărat	Adevărat	Adevărat	Fals
P137	Adevărat	Adevărat	Adevărat	Adevărat	Adevărat	Adevărat	Adevărat	Adevărat	Fals	Nu știu
P138	Adevărat	Adevărat	Adevărat	Adevărat	Adevărat	Adevărat	Adevărat	Adevărat	Adevărat	Nu știu
P139	Adevărat	Nu știu	Adevărat	Adevărat	Adevărat	Adevărat	Adevărat	Adevărat	Nu știu	Nu știu
P140	Nu știu	Nu știu	Nu știu	Nu știu	Nu știu	Nu știu	Nu știu	Nu știu	Nu știu	Nu știu
P141	Adevărat	Adevărat	Adevărat	Adevărat	Adevărat	Adevărat	Adevărat	Adevărat	Fals	Fals
P142	Adevărat	Adevărat	Fals	Fals	Adevărat	Adevărat	Nu știu	Adevărat	Nu știu	Nu știu
P143	Adevărat	Adevărat	Adevărat	Adevărat	Adevărat	Adevărat	Adevărat	Adevărat	Adevărat	Fals
P144	Adevărat	Adevărat	Adevărat	Adevărat	Adevărat	Adevărat	Adevărat	Adevărat	Fals	Fals
P145	Adevărat	Adevărat	Adevărat	Adevărat	Adevărat	Adevărat	Adevărat	Adevărat	Adevărat	Fals
P146	Adevărat	Adevărat	Adevărat	Adevărat	Adevărat	Adevărat	Adevărat	Adevărat	Nu știu	Nu știu
P147	Adevărat	Adevărat	Adevărat	Adevărat	Adevărat	Adevărat	Adevărat	Adevărat	Adevărat	Nu știu
P148	Nu știu	Adevărat	Fals	Nu știu	Adevărat	Adevărat	Adevărat	Adevărat	Fals	Nu știu
P149	Adevărat	Fals	Fals	Adevărat	Adevărat	Adevărat	Fals	Adevărat	Fals	Fals
P150	Adevărat	Adevărat	Adevărat	Adevărat	Adevărat	Adevărat	Adevărat	Adevărat	Nu știu	Nu știu
P151	Adevărat	Adevărat	Adevărat	Adevărat	Adevărat	Adevărat	Adevărat	Adevărat	Nu știu	Nu știu
P152	Adevărat	Adevărat	Adevărat	Adevărat	Adevărat	Adevărat	Adevărat	Adevărat	Nu știu	Nu știu
P153	Adevărat	Adevărat	Adevărat	Adevărat	Adevărat	Fals	Fals	Fals	Fals	Fals
P154	Adevărat	Fals	Adevărat	Fals	Fals	Adevărat	Nu știu	Nu știu	Nu știu	Fals
P155	Adevărat	Fals	Adevărat	Fals	Fals	Fals	Fals	Fals	Fals	Fals
P156	Adevărat	Adevărat	Adevărat	Adevărat	Adevărat	Nu știu	Adevărat	Adevărat	Fals	Nu știu
P157	Fals	Fals	Adevărat	Fals	Fals	Adevărat	Adevărat	Adevărat	Nu știu	Nu știu
P158	Adevărat	Adevărat	Adevărat	Adevărat	Adevărat	Fals	Adevărat	Adevărat	Adevărat	Fals
P159	Nu știu	Nu știu	Adevărat	Adevărat	Adevărat	Adevărat	Adevărat	Nu știu	Adevărat	Fals
P160	Adevărat	Nu știu	Fals	Adevărat	Nu știu	Nu știu	Adevărat	Adevărat	Fals	Fals
P161	Adevărat	Nu știu	Adevărat	Adevărat	Adevărat	Adevărat	Adevărat	Adevărat	Adevărat	Nu știu
P162	Adevărat	Nu știu	Adevărat	Adevărat	Adevărat	Adevărat	Adevărat	Adevărat	Nu știu	Nu știu

Participant_ ID	C1	C2	C3	C4	C5	C6	C7	C8	C9	C10
P163	Adevărat	Nu ştiu	Adevărat	Fals	Nu ştiu	Adevărat	Adevărat	Adevărat	Adevărat	Fals
P164	Adevărat	Adevărat	Adevărat	Adevărat	Adevărat	Adevărat	Adevărat	Adevărat	Adevărat	Nu ştiu
P165	Adevărat	Adevărat	Adevărat	Adevărat	Adevărat	Adevărat	Adevărat	Adevărat	Fals	Nu ştiu
P166	Adevărat	Nu ştiu	Adevărat	Adevărat	Adevărat	Adevărat	Adevărat	Adevărat	Adevărat	Nu ştiu
P167	Adevărat	Adevărat	Adevărat	Adevărat	Adevărat	Adevărat	Adevărat	Adevărat	Nu ştiu	Nu ştiu
P168	Adevărat	Fals	Fals	Adevărat	Adevărat	Adevărat	Adevărat	Adevărat	Adevărat	Fals
P169	Adevărat	Adevărat	Fals	Fals	Adevărat	Adevărat	Adevărat	Adevărat	Adevărat	Fals
P170	Adevărat	Adevărat	Fals	Adevărat	Adevărat	Adevărat	Adevărat	Adevărat	Nu ştiu	Nu ştiu
P171	Adevărat	Adevărat	Fals	Nu ştiu	Adevărat	Adevărat	Adevărat	Adevărat	Adevărat	Nu ştiu
P172	Adevărat	Adevărat	Adevărat	Nu ştiu	Nu ştiu	Fals	Adevărat	Fals	Nu ştiu	Nu ştiu
P173	Adevărat	Nu ştiu	Adevărat	Adevărat	Adevărat	Adevărat	Adevărat	Adevărat	Adevărat	Fals
P174	Adevărat	Adevărat	Adevărat	Adevărat	Adevărat	Adevărat	Adevărat	Adevărat	Adevărat	Fals
P175	Adevărat	Fals	Fals	Adevărat	Adevărat	Adevărat	Fals	Adevărat	Nu ştiu	Fals
P176	Nu ştiu	Adevărat	Fals	Adevărat	Nu ştiu	Adevărat	Adevărat	Adevărat	Adevărat	Fals
P177	Adevărat	Adevărat	Adevărat	Adevărat	Adevărat	Adevărat	Adevărat	Adevărat	Fals	Fals
P178	Adevărat	Adevărat	Adevărat	Adevărat	Adevărat	Adevărat	Adevărat	Adevărat	Adevărat	Adevărat
P179	Adevărat	Nu ştiu	Adevărat	Nu ştiu	Adevărat	Adevărat	Adevărat	Adevărat	Nu ştiu	Nu ştiu
P180	Adevărat	Adevărat	Adevărat	Adevărat	Adevărat	Adevărat	Adevărat	Adevărat	Nu ştiu	Nu ştiu
P181	Nu ştiu	Nu ştiu	Nu ştiu	Nu ştiu	Nu ştiu	Nu ştiu	Nu ştiu	Nu ştiu	Nu ştiu	Nu ştiu
P182	Adevărat	Nu ştiu	Fals	Adevărat	Adevărat	Adevărat	Fals	Adevărat	Nu ştiu	Fals
P183	Nu ştiu	Adevărat	Fals	Adevărat	Adevărat	Adevărat	Nu ştiu	Nu ştiu	Adevărat	Nu ştiu
P184	Adevărat	Nu ştiu	Adevărat	Nu ştiu	Nu ştiu	Adevărat	Adevărat	Adevărat	Adevărat	Fals
P185	Adevărat	Adevărat	Fals	Adevărat	Fals	Adevărat	Fals	Adevărat	Adevărat	Fals
P186	Adevărat	Adevărat	Adevărat	Nu ştiu	Adevărat	Adevărat	Nu ştiu	Adevărat	Nu ştiu	Nu ştiu
P187	Adevărat	Nu ştiu	Adevărat	Adevărat	Fals	Adevărat	Adevărat	Adevărat	Fals	Nu ştiu
P188	Adevărat	Fals	Adevărat	Adevărat	Adevărat	Nu ştiu	Adevărat	Adevărat	Adevărat	Nu ştiu
P189	Adevărat	Adevărat	Fals	Nu ştiu	Adevărat	Fals	Adevărat	Adevărat	Adevărat	Nu ştiu
P190	Adevărat	Adevărat	Fals	Adevărat	Adevărat	Adevărat	Fals	Adevărat	Adevărat	Fals
P191	Adevărat	Nu ştiu	Adevărat	Adevărat	Adevărat	Adevărat	Adevărat	Adevărat	Nu ştiu	Nu ştiu
P192	Adevărat	Nu ştiu	Adevărat	Nu ştiu	Nu ştiu	Nu ştiu	Nu ştiu	Nu ştiu	Nu ştiu	Fals
P193	Adevărat	Adevărat	Fals	Adevărat	Nu ştiu	Adevărat	Adevărat	Adevărat	Nu ştiu	Nu ştiu
P194	Adevărat	Nu ştiu	Fals	Adevărat	Nu ştiu	Adevărat	Adevărat	Nu ştiu	Adevărat	Fals
P195	Fals	Adevărat	Fals	Fals	Adevărat	Adevărat	Fals	Nu ştiu	Fals	Fals
P196	Adevărat	Adevărat	Adevărat	Nu ştiu	Adevărat	Adevărat	Adevărat	Adevărat	Nu ştiu	Nu ştiu
P197	Adevărat	Adevărat	Adevărat	Adevărat	Adevărat	Adevărat	Adevărat	Adevărat	Adevărat	Fals
P198	Nu ştiu	Nu ştiu	Nu ştiu	Nu ştiu	Nu ştiu	Nu ştiu	Nu ştiu	Nu ştiu	Nu ştiu	Nu ştiu
P199	Adevărat	Adevărat	Fals	Adevărat	Adevărat	Adevărat	Fals	Fals	Nu ştiu	Nu ştiu
P200	Nu ştiu	Nu ştiu	Nu ştiu	Adevărat	Adevărat	Adevărat	Fals	Fals	Nu ştiu	Nu ştiu
P201	Adevărat	Nu ştiu	Adevărat	Nu ştiu	Adevărat	Adevărat	Adevărat	Adevărat	Fals	Fals
P202	Adevărat	Adevărat	Fals	Adevărat	Adevărat	Adevărat	Adevărat	Adevărat	Fals	Nu ştiu
P203	Nu ştiu	Nu ştiu	Nu ştiu	Adevărat	Adevărat	Adevărat	Fals	Fals	Nu ştiu	Nu ştiu
P204	Adevărat	Adevărat	Adevărat	Adevărat	Adevărat	Adevărat	Adevărat	Nu ştiu	Nu ştiu	Nu ştiu
P205	Adevărat	Adevărat	Fals	Fals	Fals	Nu ştiu	Adevărat	Adevărat	Adevărat	Fals
P206	Adevărat	Fals	Adevărat	Adevărat	Adevărat	Nu ştiu	Adevărat	Adevărat	Nu ştiu	Fals
P207	Adevărat	Adevărat	Adevărat	Fals	Nu ştiu	Nu ştiu	Adevărat	Nu ştiu	Nu ştiu	Fals
P208	Adevărat	Adevărat	Fals	Adevărat	Adevărat	Adevărat	Adevărat	Adevărat	Adevărat	Nu ştiu
P209	Adevărat	Adevărat	Adevărat	Adevărat	Adevărat	Adevărat	Adevărat	Adevărat	Adevărat	Fals
P210	Adevărat	Fals	Fals	Adevărat	Adevărat	Adevărat	Fals	Adevărat	Fals	Fals
P211	Adevărat	Nu ştiu	Adevărat	Adevărat	Adevărat	Adevărat	Adevărat	Adevărat	Adevărat	Adevărat
P212	Adevărat	Adevărat	Adevărat	Adevărat	Adevărat	Adevărat	Adevărat	Adevărat	Adevărat	Nu ştiu
P213	Adevărat	Adevărat	Adevărat	Nu ştiu	Nu ştiu	Adevărat	Adevărat	Adevărat	Adevărat	Nu ştiu
P214	Adevărat	Adevărat	Adevărat	Adevărat	Adevărat	Adevărat	Adevărat	Adevărat	Nu ştiu	Nu ştiu
P215	Nu ştiu	Nu ştiu	Nu ştiu	Nu ştiu	Nu ştiu	Nu ştiu	Nu ştiu	Nu ştiu	Nu ştiu	Nu ştiu
P216	Adevărat	Fals	Adevărat	Nu ştiu	Adevărat	Fals	Adevărat	Adevărat	Adevărat	Nu ştiu
P217	Adevărat	Adevărat	Adevărat	Adevărat	Adevărat	Nu ştiu	Adevărat	Adevărat	Nu ştiu	Nu ştiu
P218	Nu ştiu	Nu ştiu	Nu ştiu	Nu ştiu	Nu ştiu	Nu ştiu	Nu ştiu	Nu ştiu	Nu ştiu	Nu ştiu
P219	Adevărat	Nu ştiu	Adevărat	Adevărat	Nu ştiu	Nu ştiu	Adevărat	Adevărat	Nu ştiu	Fals
P220	Adevărat	Adevărat	Adevărat	Adevărat	Adevărat	Adevărat	Adevărat	Adevărat	Fals	Fals
P221	Nu ştiu	Fals	Adevărat	Adevărat	Nu ştiu	Adevărat	Adevărat	Adevărat	Nu ştiu	Fals
P222	Adevărat	Adevărat	Adevărat	Adevărat	Adevărat	Adevărat	Adevărat	Adevărat	Adevărat	Nu ştiu
P223	Adevărat	Adevărat	Adevărat	Adevărat	Nu ştiu	Nu ştiu	Adevărat	Adevărat	Adevărat	Fals
P224	Adevărat	Adevărat	Adevărat	Adevărat	Adevărat	Nu ştiu	Adevărat	Adevărat	Adevărat	Fals
P225	Adevărat	Adevărat	Adevărat	Adevărat	Adevărat	Adevărat	Adevărat	Adevărat	Adevărat	Adevărat
P226	Adevărat	Adevărat	Fals	Fals	Adevărat	Fals	Adevărat	Fals	Adevărat	Fals
P227	Adevărat	Nu ştiu	Nu ştiu	Adevărat	Adevărat	Adevărat	Adevărat	Adevărat	Nu ştiu	Nu ştiu
P228	Adevărat	Adevărat	Adevărat	Adevărat	Adevărat	Adevărat	Adevărat	Adevărat	Nu ştiu	Fals
P229	Adevărat	Adevărat	Adevărat	Nu ştiu	Adevărat	Adevărat	Adevărat	Adevărat	Adevărat	Nu ştiu
P230	Adevărat	Adevărat	Adevărat	Adevărat	Adevărat	Adevărat	Adevărat	Adevărat	Adevărat	Fals
P231	Adevărat	Nu ştiu	Adevărat	Nu ştiu	Adevărat	Adevărat	Nu ştiu	Adevărat	Fals	Fals
P232	Adevărat	Fals	Fals	Adevărat	Adevărat	Adevărat	Adevărat	Adevărat	Adevărat	Fals
P233	Adevărat	Nu ştiu	Nu ştiu	Nu ştiu	Nu ştiu	Adevărat	Nu ştiu	Nu ştiu	Nu ştiu	Nu ştiu
P234	Adevărat	Nu ştiu	Adevărat	Adevărat	Nu ştiu	Nu ştiu	Adevărat	Adevărat	Adevărat	Nu ştiu
P235	Nu ştiu	Adevărat	Adevărat	Nu ştiu	Adevărat	Nu ştiu	Nu ştiu	Nu ştiu	Nu ştiu	Nu ştiu
P236	Adevărat	Fals	Fals	Nu ştiu	Nu ştiu	Adevărat	Adevărat	Adevărat	Nu ştiu	Fals
P237	Adevărat	Adevărat	Adevărat	Adevărat	Adevărat	Adevărat	Adevărat	Adevărat	Adevărat	Adevărat
P238	Adevărat	Adevărat	Adevărat	Adevărat	Nu ştiu	Nu ştiu	Adevărat	Adevărat	Fals	Nu ştiu
P239	Adevărat	Adevărat	Adevărat	Nu ştiu	Adevărat	Nu ştiu	Adevărat	Adevărat	Nu ştiu	Nu ştiu
P240	Adevărat	Nu ştiu	Adevărat	Adevărat	Nu ştiu	Nu ştiu	Adevărat	Adevărat	Nu ştiu	Nu ştiu
P241	Adevărat	Adevărat	Adevărat	Nu ştiu	Nu ştiu	Adevărat	Adevărat	Adevărat	Nu ştiu	Fals
P242	Adevărat	Adevărat	Adevărat	Adevărat	Adevărat	Nu ştiu	Adevărat	Adevărat	Nu ştiu	Nu ştiu
P243	Adevărat	Adevărat	Adevărat	Adevărat	Fals	Nu ştiu	Adevărat	Adevărat	Nu ştiu	Fals
P244	Nu ştiu	Nu ştiu	Adevărat	Adevărat	Nu ştiu	Nu ştiu	Adevărat	Adevărat	Nu ştiu	Nu ştiu

Participant_ ID	C1	C2	C3	C4	C5	C6	C7	C8	C9	C10
P245	Adevărat	Nu știu	Adevărat	Fals	Nu știu	Adevărat	Nu știu	Nu știu	Nu știu	Nu știu
P246	Fals	Adevărat	Adevărat	Adevărat	Nu știu	Fals	Adevărat	Fals	Nu știu	Fals
P247	Nu știu	Adevărat	Fals	Adevărat	Fals	Adevărat	Adevărat	Adevărat	Nu știu	Nu știu
P248	Nu știu	Nu știu	Nu știu	Nu știu	Nu știu	Nu știu	Nu știu	Nu știu	Nu știu	Nu știu
P249	Adevărat	Fals	Adevărat	Nu știu	Fals	Fals	Adevărat	Nu știu	Adevărat	Adevărat
P250	Adevărat	Adevărat	Adevărat	Nu știu	Nu știu	Adevărat	Adevărat	Adevărat	Nu știu	Nu știu
P251	Adevărat	Adevărat	Adevărat	Adevărat	Adevărat	Adevărat	Adevărat	Adevărat	Adevărat	Fals
P252	Adevărat	Nu știu	Adevărat	Adevărat	Adevărat	Nu știu	Adevărat	Adevărat	Adevărat	Nu știu
P253	Adevărat	Adevărat	Adevărat	Adevărat	Adevărat	Adevărat	Adevărat	Adevărat	Adevărat	Fals
P254	Nu știu	Nu știu	Fals	Nu știu	Adevărat	Nu știu	Adevărat	Nu știu	Nu știu	Fals
P255	Fals	Adevărat	Fals	Nu știu	Adevărat	Adevărat	Adevărat	Adevărat	Nu știu	Nu știu
P256	Adevărat	Adevărat	Adevărat	Adevărat	Adevărat	Adevărat	Adevărat	Adevărat	Adevărat	Fals
P257	Adevărat	Adevărat	Adevărat	Adevărat	Adevărat	Adevărat	Adevărat	Adevărat	Adevărat	Nu știu
P258	Adevărat	Adevărat	Adevărat	Fals	Fals	Adevărat	Adevărat	Adevărat	Adevărat	Adevărat
P259	Adevărat	Fals	Adevărat	Adevărat	Adevărat	Adevărat	Adevărat	Adevărat	Fals	Fals
P260	Adevărat	Adevărat	Adevărat	Nu știu	Nu știu	Adevărat	Adevărat	Adevărat	Nu știu	Nu știu
P261	Adevărat	Adevărat	Adevărat	Adevărat	Adevărat	Adevărat	Adevărat	Adevărat	Adevărat	Adevărat
P262	Adevărat	Nu știu	Fals	Nu știu	Adevărat	Adevărat	Adevărat	Adevărat	Adevărat	Fals
P263	Nu știu	Nu știu	Nu știu	Nu știu	Nu știu	Nu știu	Nu știu	Adevărat	Nu știu	Nu știu
P264	Adevărat	Adevărat	Adevărat	Adevărat	Adevărat	Adevărat	Adevărat	Adevărat	Adevărat	Fals
P265	Adevărat	Adevărat	Adevărat	Adevărat	Adevărat	Adevărat	Adevărat	Nu știu	Fals	Nu știu
P266	Adevărat	Adevărat	Adevărat	Nu știu	Adevărat	Adevărat	Adevărat	Adevărat	Adevărat	Nu știu
P267	Adevărat	Adevărat	Nu știu	Adevărat	Fals	Adevărat	Fals	Fals	Fals	Nu știu
P268	Adevărat	Adevărat	Fals	Adevărat	Adevărat	Adevărat	Adevărat	Adevărat	Adevărat	Fals
P269	Adevărat	Adevărat	Adevărat	Adevărat	Adevărat	Adevărat	Adevărat	Adevărat	Adevărat	Fals
P270	Adevărat	Adevărat	Adevărat	Adevărat	Adevărat	Adevărat	Adevărat	Adevărat	Adevărat	Fals
P271	Adevărat	Nu știu	Nu știu	Adevărat	Nu știu	Adevărat	Adevărat	Nu știu	Nu știu	Nu știu
P272	Adevărat	Adevărat	Adevărat	Nu știu	Adevărat	Adevărat	Adevărat	Adevărat	Nu știu	Nu știu
P273	Adevărat	Adevărat	Adevărat	Adevărat	Adevărat	Adevărat	Adevărat	Adevărat	Adevărat	Adevărat
P274	Nu știu	Fals	Adevărat	Fals	Fals	Fals	Nu știu	Fals	Adevărat	Fals
P275	Adevărat	Adevărat	Adevărat	Adevărat	Adevărat	Adevărat	Adevărat	Adevărat	Adevărat	Fals
P276	Adevărat	Adevărat	Adevărat	Adevărat	Adevărat	Adevărat	Nu știu	Nu știu	Adevărat	Nu știu
P277	Fals	Fals	Adevărat	Nu știu	Fals	Nu știu	Adevărat	Adevărat	Nu știu	Fals
P278	Adevărat	Adevărat	Fals	Adevărat	Adevărat	Adevărat	Adevărat	Adevărat	Nu știu	Nu știu
P279	Adevărat	Adevărat	Adevărat	Nu știu	Adevărat	Adevărat	Adevărat	Adevărat	Adevărat	Fals
P280	Adevărat	Fals	Fals	Fals	Fals	Fals	Fals	Adevărat	Fals	Fals
P281	Fals	Fals	Nu știu	Fals	Adevărat	Fals	Adevărat	Adevărat	Fals	Fals
P282	Adevărat	Adevărat	Fals	Adevărat	Adevărat	Adevărat	Fals	Adevărat	Nu știu	Nu știu
P283	Adevărat	Adevărat	Adevărat	Adevărat	Adevărat	Adevărat	Adevărat	Nu știu	Adevărat	Fals
P284	Adevărat	Adevărat	Adevărat	Nu știu	Nu știu	Nu știu	Adevărat	Adevărat	Adevărat	Nu știu
P285	Adevărat	Adevărat	Adevărat	Nu știu	Nu știu	Nu știu	Adevărat	Adevărat	Adevărat	Nu știu
P286	Adevărat	Adevărat	Fals	Adevărat	Nu știu	Adevărat	Adevărat	Adevărat	Nu știu	Fals
P287	Adevărat	Fals	Fals	Nu știu	Adevărat	Adevărat	Adevărat	Adevărat	Adevărat	Nu știu
P288	Adevărat	Adevărat	Adevărat	Nu știu	Adevărat	Adevărat	Nu știu	Adevărat	Nu știu	Nu știu
P289	Adevărat	Adevărat	Adevărat	Adevărat	Adevărat	Adevărat	Adevărat	Adevărat	Adevărat	Adevărat
P290	Adevărat	Fals	Fals	Adevărat	Adevărat	Adevărat	Adevărat	Adevărat	Fals	Fals
P291	Adevărat	Adevărat	Adevărat	Adevărat	Adevărat	Adevărat	Adevărat	Adevărat	Adevărat	Nu știu
P292	Adevărat	Adevărat	Adevărat	Adevărat	Adevărat	Adevărat	Adevărat	Adevărat	Adevărat	Adevărat
P293	Fals	Adevărat	Adevărat	Nu știu	Nu știu	Fals	Adevărat	Adevărat	Nu știu	Nu știu
P294	Adevărat	Adevărat	Adevărat	Adevărat	Adevărat	Adevărat	Adevărat	Adevărat	Adevărat	Adevărat
P295	Adevărat	Fals	Adevărat	Adevărat	Adevărat	Adevărat	Adevărat	Adevărat	Nu știu	Nu știu
P296	Nu știu	Nu știu	Nu știu	Adevărat	Adevărat	Adevărat	Adevărat	Adevărat	Adevărat	Adevărat
P297	Adevărat	Adevărat	Adevărat	Adevărat	Adevărat	Fals	Adevărat	Adevărat	Fals	Adevărat
P298	Adevărat	Adevărat	Adevărat	Adevărat	Adevărat	Adevărat	Adevărat	Adevărat	Fals	Nu știu
P299	Adevărat	Adevărat	Fals	Adevărat	Adevărat	Adevărat	Adevărat	Adevărat	Adevărat	Adevărat
P300	Adevărat	Adevărat	Adevărat	Adevărat	Adevărat	Adevărat	Adevărat	Adevărat	Adevărat	Nu știu
P301	Adevărat	Adevărat	Adevărat	Adevărat	Adevărat	Adevărat	Adevărat	Adevărat	Fals	Adevărat

Panel D — Attitudes toward dietary change and perceived barriers

Participant_ID	D1	D2	D3	D4	D5	D6	D7
P001	Acord total	Acord total	Acord total	Acord parțial	Dezacord total	Dezacord total	Dezacord parțial
P002	Acord parțial	Acord total	Acord parțial	Acord total	Dezacord parțial	Dezacord total	Dezacord parțial
P003	Dezacord parțial	Neutru	Neutru	Neutru	Dezacord parțial	Neutru	Dezacord parțial
P004	Dezacord parțial	Dezacord total	Dezacord total	Dezacord total	Neutru	Neutru	Neutru
P005	Neutru	Neutru	Neutru	Neutru	Neutru	Neutru	Neutru
P006	Acord parțial	Acord total	Acord total	Acord total	Acord parțial	Acord parțial	Acord total
P007	Neutru	Neutru	Dezacord parțial	Neutru	Dezacord total	Dezacord total	Dezacord total
P008	Dezacord parțial	Dezacord parțial	Dezacord total	Neutru	Dezacord parțial	Dezacord total	Neutru
P009	Acord total	Neutru	Acord parțial	Acord total	Dezacord parțial	Dezacord parțial	Neutru
P010	Dezacord total	Dezacord parțial	Dezacord total	Dezacord total	Dezacord total	Dezacord parțial	Dezacord parțial
P011	Acord parțial	Acord total	Acord total	Acord total	Neutru	Neutru	Neutru
P012	Neutru	Neutru	Neutru	Neutru	Dezacord parțial	Neutru	Dezacord parțial
P013	Dezacord total	Dezacord parțial	Neutru	Dezacord parțial	Dezacord total	Neutru	Dezacord parțial
P014	Acord total	Acord total	Acord total	Acord total	Neutru	Dezacord parțial	Dezacord parțial
P015	Acord parțial	Acord parțial	Acord parțial	Acord parțial	Acord parțial	Acord parțial	Acord parțial
P016	Acord parțial	Acord total	Acord total	Acord total	Dezacord total	Dezacord total	Acord parțial
P017	Dezacord parțial	Acord parțial	Acord parțial	Neutru	Neutru	Acord parțial	Acord parțial
P018	Dezacord parțial	Dezacord total	Dezacord total	Dezacord total	Neutru	Dezacord parțial	Dezacord total
P019	Neutru	Acord total	Acord parțial	Neutru	Neutru	Acord parțial	Acord parțial
P020	Acord parțial	Acord total	Neutru	Acord parțial	Neutru	Neutru	Neutru
P021	Acord parțial	Acord total	Acord total	Acord total	Dezacord total	Acord parțial	Dezacord total
P022	Acord parțial	Acord total	Acord parțial	Acord parțial	Dezacord total	Acord total	Dezacord parțial
P023	Acord parțial	Acord total	Acord total	Acord total	Neutru	Neutru	Acord total
P024	Acord parțial	Acord total	Acord total	Acord parțial	Dezacord parțial	Dezacord parțial	Dezacord total
P025	Acord parțial	Acord total	Acord total	Acord parțial	Dezacord parțial	Dezacord parțial	Dezacord parțial
P026	Acord parțial	Acord total	Acord total	Acord parțial	Dezacord total	Dezacord parțial	Dezacord parțial
P027	Acord parțial	Acord total	Acord total	Acord parțial	Dezacord parțial	Dezacord parțial	Neutru
P028	Neutru	Dezacord parțial	Neutru	Neutru	Dezacord parțial	Dezacord parțial	Dezacord parțial
P029	Acord total	Neutru	Acord total	Acord total	Neutru	Acord total	Neutru
P030	Neutru	Acord parțial	Acord total	Neutru	Neutru	Acord parțial	Acord parțial
P031	Acord parțial	Neutru	Neutru	Neutru	Acord parțial	Dezacord total	Acord parțial
P032	Neutru	Neutru	Neutru	Neutru	Neutru	Neutru	Neutru
P033	Acord parțial	Acord parțial	Acord parțial	Acord parțial	Acord parțial	Acord parțial	Acord parțial
P034	Acord parțial	Acord parțial	Acord parțial	Acord parțial	Dezacord parțial	Dezacord parțial	Neutru
P035	Acord total	Acord total	Acord parțial	Acord parțial	Dezacord parțial	Neutru	Neutru
P036	Neutru	Acord parțial	Acord parțial	Acord total	Dezacord parțial	Acord parțial	Acord parțial
P037	Acord parțial	Acord total	Acord parțial	Acord total	Neutru	Acord total	Acord parțial
P038	Acord parțial	Acord parțial	Acord parțial	Acord parțial	Neutru	Acord parțial	Neutru
P039	Dezacord parțial	Dezacord total	Dezacord total	Dezacord parțial	Neutru	Dezacord parțial	Dezacord parțial
P040	Dezacord parțial	Neutru	Neutru	Dezacord parțial	Dezacord total	Neutru	Neutru
P041	Acord parțial	Acord total	Acord parțial	Acord parțial	Neutru	Neutru	Acord total
P042	Acord parțial	Acord total	Acord total	Acord total	Acord parțial	Dezacord parțial	Neutru
P043	Acord parțial	Acord total	Acord total	Acord total	Dezacord parțial	Dezacord total	Dezacord total
P044	Dezacord total	Neutru	Neutru	Dezacord total	Neutru	Neutru	Neutru
P045	Neutru	Acord total	Acord total	Acord total	Acord total	Neutru	Neutru
P046	Acord parțial	Acord parțial	Acord parțial	Neutru	Dezacord total	Neutru	Acord parțial
P047	Acord parțial	Acord total	Acord total	Acord parțial	Neutru	Acord total	Acord parțial
P048	Acord parțial	Neutru	Acord total	Acord parțial	Acord parțial	Dezacord total	Neutru
P049	Acord parțial	Acord parțial	Acord total	Acord parțial	Dezacord parțial	Neutru	Neutru
P050	Neutru	Neutru	Neutru	Acord total	Neutru	Neutru	Acord parțial
P051	Neutru	Acord total	Acord parțial	Dezacord parțial	Neutru	Neutru	Acord parțial
P052	Acord parțial	Acord total	Acord total	Acord total	Dezacord total	Dezacord total	Dezacord total
P053	Dezacord parțial	Acord total	Acord total	Acord total	Dezacord parțial	Dezacord total	Dezacord parțial
P054	Dezacord parțial	Neutru	Neutru	Neutru	Neutru	Neutru	Neutru
P055	Neutru	Dezacord parțial	Acord parțial	Acord total	Neutru	Acord total	Acord total
P056	Dezacord total	Dezacord total	Dezacord total	Dezacord parțial	Neutru	Neutru	Neutru
P057	Dezacord parțial	Acord parțial	Acord parțial	Neutru	Acord parțial	Neutru	Acord parțial
P058	Acord parțial	Acord total	Acord total	Acord total	Dezacord total	Dezacord total	Dezacord parțial
P059	Dezacord parțial	Dezacord total	Dezacord total	Dezacord parțial	Neutru	Dezacord parțial	Dezacord parțial
P060	Dezacord total	Dezacord parțial	Dezacord total	Neutru	Dezacord parțial	Dezacord total	Neutru
P061	Acord parțial	Acord total	Acord total	Acord parțial	Neutru	Acord parțial	Acord parțial
P062	Neutru	Acord total	Acord total	Acord parțial	Dezacord parțial	Dezacord total	Neutru
P063	Acord parțial	Acord total	Acord total	Dezacord parțial	Neutru	Acord total	Acord parțial
P064	Acord parțial	Acord total	Acord parțial	Acord parțial	Acord parțial	Neutru	Neutru
P065	Acord parțial	Acord parțial	Acord parțial	Acord parțial	Dezacord parțial	Dezacord total	Dezacord parțial
P066	Acord parțial	Acord total	Acord total	Acord parțial	Dezacord total	Dezacord parțial	Neutru
P067	Acord parțial	Acord parțial	Acord parțial	Neutru	Dezacord total	Neutru	Dezacord parțial
P068	Neutru	Acord parțial	Acord total	Neutru	Acord parțial	Acord total	Acord total
P069	Acord parțial	Acord total	Acord total	Acord total	Dezacord parțial	Dezacord total	Dezacord total
P070	Acord total	Neutru	Dezacord parțial	Acord total	Neutru	Dezacord total	Dezacord total
P071	Acord parțial	Acord total	Acord total	Acord total	Neutru	Dezacord total	Dezacord parțial
P072	Acord parțial	Acord parțial	Acord parțial	Acord parțial	Acord parțial	Acord parțial	Acord parțial
P073	Acord parțial	Acord parțial	Acord total	Acord total	Dezacord parțial	Acord parțial	Acord parțial
P074	Neutru	Acord parțial	Acord parțial	Neutru	Dezacord total	Dezacord total	Dezacord total
P075	Acord total	Acord total	Acord total	Acord total	Dezacord total	Acord parțial	Acord parțial
P076	Acord parțial	Acord total	Acord total	Acord total	Dezacord parțial	Dezacord parțial	Dezacord parțial
P077	Dezacord parțial	Neutru	Acord total	Acord parțial	Dezacord parțial	Acord parțial	Neutru
P078	Acord parțial	Acord parțial	Acord total	Acord parțial	Dezacord total	Dezacord parțial	Dezacord total
P079	Acord parțial	Acord total	Acord total	Acord total	Dezacord total	Neutru	Neutru
P080	Dezacord parțial	Dezacord total	Dezacord parțial	Dezacord total	Dezacord total	Dezacord parțial	Dezacord parțial

Participant_ ID	D1	D2	D3	D4	D5	D6	D7
P081	Acord total	Acord total	Acord total	Acord total	Neutru	Neutru	Neutru
P082	Neutru	Acord parțial	Acord parțial	Dezacord parțial	Neutru	Dezacord parțial	Acord parțial
P083	Neutru	Dezacord total	Dezacord total	Dezacord total	Dezacord parțial	Neutru	Dezacord total
P084	Acord parțial	Acord total	Acord total	Acord total	Dezacord parțial	Dezacord total	Dezacord total
P085	Dezacord parțial	Acord total	Acord total	Acord parțial	Neutru	Neutru	Acord parțial
P086	Dezacord parțial	Dezacord total	Dezacord total	Dezacord total	Dezacord total	Dezacord parțial	Dezacord parțial
P087	Acord total	Acord total	Acord total	Acord total	Dezacord parțial	Acord parțial	Acord parțial
P088	Neutru	Acord total	Acord total	Acord parțial	Acord parțial	Dezacord parțial	Dezacord parțial
P089	Neutru	Neutru	Neutru	Neutru	Neutru	Neutru	Neutru
P090	Neutru	Neutru	Neutru	Dezacord parțial	Neutru	Neutru	Neutru
P091	Acord parțial	Acord parțial	Acord total	Neutru	Acord total	Dezacord total	Neutru
P092	Dezacord parțial	Acord parțial	Neutru	Dezacord parțial	Neutru	Dezacord parțial	Dezacord parțial
P093	Dezacord total	Dezacord total	Dezacord total	Dezacord total	Dezacord parțial	Dezacord parțial	Dezacord total
P094	Acord parțial	Acord parțial	Neutru	Acord parțial	Neutru	Neutru	Neutru
P095	Neutru	Acord total	Acord total	Acord parțial	Neutru	Acord parțial	Acord total
P096	Dezacord parțial	Neutru	Neutru	Dezacord parțial	Neutru	Dezacord total	Dezacord parțial
P097	Neutru	Neutru	Neutru	Neutru	Neutru	Dezacord total	Neutru
P098	Dezacord parțial	Neutru	Dezacord parțial	Dezacord parțial	Dezacord total	Neutru	Neutru
P099	Acord total	Acord total	Acord total	Acord total	Acord total	Acord total	Acord total
P100	Acord parțial	Acord total	Acord parțial	Acord total	Neutru	Dezacord total	Dezacord total
P101	Acord total	Acord total	Acord total	Acord total	Dezacord parțial	Dezacord total	Dezacord total
P102	Acord parțial	Acord total	Acord total	Acord parțial	Dezacord parțial	Dezacord total	Acord parțial
P103	Acord total	Acord total	Acord total	Acord parțial	Dezacord total	Dezacord total	Acord parțial
P104	Acord total	Acord total	Acord total	Acord total	Dezacord parțial	Acord parțial	Acord parțial
P105	Acord total	Acord total	Acord total	Acord total	Acord total	Neutru	Neutru
P106	Dezacord total	Acord total	Acord parțial	Dezacord parțial	Dezacord total	Acord parțial	Acord total
P107	Neutru	Neutru	Neutru	Dezacord parțial	Dezacord parțial	Neutru	Neutru
P108	Dezacord total	Acord total	Neutru	Dezacord total	Acord total	Acord total	Acord total
P109	Dezacord parțial	Acord total	Acord total	Acord parțial	Neutru	Neutru	Acord parțial
P110	Dezacord parțial	Dezacord parțial	Dezacord parțial	Neutru	Neutru	Neutru	Neutru
P111	Neutru	Neutru	Neutru	Neutru	Dezacord parțial	Dezacord parțial	Dezacord parțial
P112	Acord parțial	Acord total	Acord total	Acord total	Dezacord parțial	Dezacord total	Acord total
P113	Acord parțial	Acord total	Acord total	Acord parțial	Acord parțial	Dezacord parțial	Acord parțial
P114	Neutru	Acord total	Acord total	Acord total	Dezacord total	Dezacord total	Acord total
P115	Acord parțial	Acord total	Acord total	Acord total	Neutru	Neutru	Neutru
P116	Dezacord parțial	Acord total	Acord parțial	Acord parțial	Neutru	Dezacord total	Acord total
P117	Neutru	Dezacord parțial	Neutru	Neutru	Neutru	Dezacord parțial	Neutru
P118	Acord parțial	Acord total	Acord total	Acord total	Neutru	Neutru	Acord total
P119	Acord parțial	Acord total	Acord total	Acord parțial	Dezacord parțial	Dezacord parțial	Dezacord parțial
P120	Acord parțial	Acord total	Acord total	Acord parțial	Dezacord total	Dezacord total	Acord total
P121	Acord parțial	Acord total	Acord parțial	Neutru	Dezacord parțial	Dezacord parțial	Acord total
P122	Neutru	Neutru	Neutru	Dezacord parțial	Dezacord parțial	Dezacord parțial	Dezacord parțial
P123	Neutru	Neutru	Neutru	Acord total	Acord parțial	Dezacord parțial	Neutru
P124	Acord parțial	Acord parțial	Acord total	Acord total	Neutru	Dezacord total	Dezacord parțial
P125	Neutru	Acord parțial	Acord total	Acord parțial	Acord parțial	Acord total	Acord total
P126	Neutru	Dezacord total	Dezacord total	Dezacord parțial	Dezacord parțial	Dezacord parțial	Dezacord parțial
P127	Dezacord parțial	Neutru	Acord parțial	Acord total	Neutru	Acord parțial	Dezacord total
P128	Acord parțial	Acord total	Acord total	Acord total	Dezacord total	Acord total	Dezacord total
P129	Dezacord parțial	Dezacord total	Dezacord total	Dezacord total	Dezacord parțial	Dezacord parțial	Dezacord parțial
P130	Dezacord parțial	Dezacord total	Dezacord total	Dezacord total	Dezacord total	Dezacord total	Dezacord total
P131	Acord parțial	Acord total	Acord parțial	Neutru	Dezacord parțial	Dezacord total	Neutru
P132	Dezacord parțial	Acord total	Acord parțial	Acord total	Neutru	Dezacord total	Acord total
P133	Acord total	Acord total	Acord total	Acord total	Dezacord total	Dezacord total	Dezacord total
P134	Neutru	Dezacord parțial	Neutru	Neutru	Neutru	Dezacord total	Dezacord parțial
P135	Dezacord parțial	Dezacord total	Dezacord total	Dezacord total	Dezacord parțial	Dezacord parțial	Dezacord total
P136	Dezacord parțial	Dezacord total	Dezacord total	Neutru	Dezacord parțial	Dezacord total	Acord parțial
P137	Acord parțial	Acord total	Acord parțial	Acord total	Acord parțial	Dezacord total	Dezacord parțial
P138	Neutru	Neutru	Neutru	Acord total	Dezacord total	Acord parțial	Dezacord total
P139	Neutru	Neutru	Neutru	Neutru	Neutru	Neutru	Neutru
P140	Neutru	Acord parțial	Acord parțial	Acord parțial	Acord parțial	Neutru	Acord parțial
P141	Dezacord parțial	Dezacord total	Dezacord total	Dezacord total	Dezacord parțial	Dezacord parțial	Dezacord parțial
P142	Acord parțial	Acord total	Neutru	Acord parțial	Neutru	Dezacord parțial	Neutru
P143	Acord parțial	Acord total	Acord total	Acord total	Acord parțial	Neutru	Dezacord parțial
P144	Acord parțial	Acord parțial	Acord total	Acord parțial	Acord total	Neutru	Acord parțial
P145	Neutru	Acord parțial	Neutru	Dezacord parțial	Acord parțial	Dezacord total	Acord total
P146	Neutru	Acord total	Acord total	Dezacord parțial	Dezacord parțial	Dezacord total	Dezacord total
P147	Acord parțial	Acord total	Acord total	Acord total	Dezacord total	Dezacord total	Dezacord parțial
P148	Neutru	Acord total	Acord parțial	Acord parțial	Neutru	Dezacord parțial	Neutru
P149	Neutru	Acord total	Acord total	Neutru	Acord parțial	Neutru	Acord total
P150	Dezacord parțial	Neutru	Dezacord parțial	Dezacord parțial	Neutru	Dezacord parțial	Dezacord parțial
P151	Acord total	Acord total	Acord total	Acord total	Dezacord total	Dezacord total	Dezacord total
P152	Dezacord parțial	Acord total	Acord total	Neutru	Acord parțial	Acord parțial	Acord parțial
P153	Neutru	Neutru	Dezacord parțial	Neutru	Dezacord parțial	Dezacord parțial	Neutru
P154	Acord parțial	Acord total	Acord parțial	Acord parțial	Acord parțial	Acord parțial	Acord parțial
P155	Dezacord total	Dezacord parțial	Dezacord parțial	Dezacord parțial	Dezacord parțial	Dezacord parțial	Dezacord parțial
P156	Dezacord parțial	Dezacord parțial	Dezacord parțial	Dezacord parțial	Neutru	Neutru	Neutru
P157	Acord parțial	Acord parțial	Acord total	Neutru	Neutru	Dezacord total	Acord parțial
P158	Dezacord parțial	Dezacord parțial	Dezacord parțial	Dezacord parțial	Dezacord parțial	Dezacord parțial	Dezacord parțial
P159	Dezacord parțial	Dezacord total	Dezacord total	Dezacord total	Dezacord total	Dezacord parțial	Dezacord total
P160	Dezacord parțial	Dezacord parțial	Neutru	Neutru	Dezacord total	Neutru	Neutru
P161	Acord parțial	Acord parțial	Acord total	Neutru	Neutru	Dezacord parțial	Acord parțial
P162	Acord total	Acord total	Acord total	Acord parțial	Dezacord total	Dezacord total	Neutru

Participant_ID	D1	D2	D3	D4	D5	D6	D7
P163	Acord total	Acord parțial	Acord total	Acord total	Neutru	Dezacord parțial	Dezacord parțial
P164	Dezacord total	Acord total	Neutru	Dezacord total	Neutru	Dezacord total	Acord parțial
P165	Dezacord parțial	Acord total	Acord parțial	Acord parțial	Dezacord parțial	Dezacord parțial	Neutru
P166	Neutru	Dezacord parțial	Dezacord total	Dezacord total	Dezacord total	Dezacord total	Dezacord total
P167	Acord parțial	Acord total	Acord total	Acord parțial	Neutru	Acord parțial	Dezacord parțial
P168	Acord parțial	Acord parțial	Acord parțial	Acord total	Acord total	Acord parțial	Acord parțial
P169	Neutru	Neutru	Neutru	Neutru	Dezacord parțial	Neutru	Neutru
P170	Acord parțial	Acord total	Acord total	Acord total	Dezacord parțial	Acord total	Neutru
P171	Acord parțial	Acord total	Acord total	Acord parțial	Dezacord total	Dezacord total	Dezacord total
P172	Dezacord parțial	Acord parțial	Acord parțial	Acord parțial	Acord parțial	Neutru	Neutru
P173	Neutru	Acord total	Acord total	Neutru	Dezacord total	Dezacord total	Neutru
P174	Acord parțial	Acord total	Acord total	Acord total	Acord parțial	Neutru	Neutru
P175	Dezacord parțial	Dezacord total	Dezacord total	Dezacord total	Neutru	Dezacord parțial	Dezacord parțial
P176	Acord parțial	Acord parțial	Acord total	Acord parțial	Neutru	Neutru	Acord total
P177	Acord parțial	Dezacord parțial	Acord total	Acord parțial	Acord total	Acord total	Acord total
P178	Acord parțial	Acord parțial	Acord parțial	Acord parțial	Acord parțial	Acord parțial	Acord parțial
P179	Neutru	Acord total	Acord parțial	Neutru	Dezacord parțial	Dezacord total	Dezacord total
P180	Dezacord total	Acord total	Acord total	Acord parțial	Acord total	Dezacord total	Acord total
P181	Acord parțial	Acord parțial	Acord total	Acord total	Neutru	Neutru	Neutru
P182	Acord parțial	Acord total	Acord total	Acord total	Dezacord parțial	Dezacord total	Dezacord parțial
P183	Acord parțial	Acord total	Acord total	Acord total	Acord parțial	Acord total	Acord total
P184	Acord parțial	Acord parțial	Acord total	Acord parțial	Neutru	Acord parțial	Acord parțial
P185	Acord parțial	Acord parțial	Acord parțial	Acord parțial	Acord parțial	Acord parțial	Acord parțial
P186	Acord parțial	Acord total	Acord total	Acord total	Acord parțial	Neutru	Acord parțial
P187	Neutru	Acord total	Neutru	Dezacord parțial	Neutru	Neutru	Neutru
P188	Dezacord parțial	Dezacord total	Dezacord total	Neutru	Dezacord parțial	Dezacord parțial	Dezacord parțial
P189	Dezacord parțial	Acord total	Acord total	Neutru	Neutru	Dezacord total	Acord parțial
P190	Acord parțial	Acord parțial	Acord total	Acord total	Acord parțial	Acord parțial	Neutru
P191	Neutru	Acord parțial	Acord total	Acord parțial	Acord parțial	Neutru	Neutru
P192	Acord parțial	Neutru	Acord parțial	Dezacord parțial	Dezacord parțial	Neutru	Dezacord parțial
P193	Dezacord parțial	Dezacord total	Dezacord total	Dezacord parțial	Dezacord parțial	Dezacord parțial	Dezacord parțial
P194	Acord parțial	Acord parțial	Acord total	Acord total	Neutru	Acord parțial	Neutru
P195	Dezacord parțial	Neutru	Neutru	Neutru	Dezacord total	Dezacord total	Dezacord parțial
P196	Dezacord parțial	Dezacord total	Acord total	Acord total	Dezacord parțial	Acord total	Acord total
P197	Neutru	Acord parțial	Acord parțial	Dezacord total	Neutru	Dezacord total	Acord total
P198	Dezacord parțial	Acord parțial	Acord total	Acord parțial	Acord total	Dezacord parțial	Acord total
P199	Neutru	Acord parțial	Acord parțial	Acord parțial	Acord parțial	Neutru	Acord parțial
P200	Dezacord parțial	Neutru	Dezacord parțial	Acord parțial	Neutru	Acord parțial	Acord parțial
P201	Neutru	Acord total	Acord total	Acord parțial	Dezacord parțial	Dezacord parțial	Acord parțial
P202	Acord parțial	Acord total	Acord parțial	Acord parțial	Dezacord total	Dezacord parțial	Acord parțial
P203	Dezacord parțial	Acord total	Acord total	Acord parțial	Dezacord total	Dezacord total	Dezacord parțial
P204	Dezacord parțial	Dezacord parțial	Dezacord parțial	Neutru	Dezacord parțial	Dezacord parțial	Dezacord parțial
P205	Dezacord parțial	Acord parțial	Neutru	Acord parțial	Neutru	Dezacord total	Neutru
P206	Acord parțial	Acord total	Acord total	Acord parțial	Neutru	Dezacord total	Dezacord total
P207	Dezacord parțial	Acord total	Acord total	Acord parțial	Dezacord parțial	Acord parțial	Acord total
P208	Acord total	Acord total	Acord total	Acord total	Acord total	Acord total	Acord total
P209	Acord parțial	Acord parțial	Acord parțial	Acord parțial	Neutru	Dezacord parțial	Acord parțial
P210	Dezacord parțial	Dezacord total	Dezacord total	Dezacord total	Dezacord parțial	Dezacord parțial	Dezacord parțial
P211	Neutru	Neutru	Neutru	Dezacord parțial	Dezacord parțial	Dezacord parțial	Dezacord parțial
P212	Acord parțial	Acord total	Acord total	Acord parțial	Acord parțial	Neutru	Acord parțial
P213	Neutru	Neutru	Dezacord parțial	Dezacord parțial	Dezacord parțial	Dezacord parțial	Dezacord parțial
P214	Acord parțial	Acord total	Acord total	Acord parțial	Dezacord parțial	Dezacord parțial	Acord parțial
P215	Acord total	Acord total	Acord total	Acord total	Acord total	Acord total	Acord total
P216	Dezacord parțial	Dezacord parțial	Neutru	Neutru	Dezacord total	Dezacord total	Neutru
P217	Acord parțial	Acord parțial	Acord total	Acord total	Acord parțial	Dezacord total	Dezacord parțial
P218	Acord total	Acord parțial	Acord total	Acord total	Acord parțial	Dezacord total	Neutru
P219	Neutru	Acord parțial	Acord parțial	Acord parțial	Neutru	Neutru	Neutru
P220	Dezacord parțial	Dezacord parțial	Dezacord total	Dezacord parțial	Dezacord parțial	Dezacord parțial	Dezacord parțial
P221	Neutru	Acord parțial	Neutru	Acord parțial	Acord parțial	Dezacord parțial	Acord parțial
P222	Dezacord parțial	Acord parțial	Acord total	Acord parțial	Acord parțial	Neutru	Neutru
P223	Dezacord parțial	Neutru	Neutru	Neutru	Neutru	Dezacord total	Dezacord total
P224	Acord parțial	Acord total	Acord total	Acord total	Acord parțial	Dezacord total	Acord parțial
P225	Dezacord parțial	Neutru	Dezacord parțial	Dezacord parțial	Neutru	Neutru	Neutru
P226	Acord parțial	Acord total	Acord total	Acord total	Dezacord total	Dezacord parțial	Dezacord parțial
P227	Neutru	Dezacord parțial	Dezacord parțial	Dezacord parțial	Neutru	Neutru	Neutru
P228	Acord parțial	Acord total	Acord total	Acord parțial	Neutru	Dezacord total	Dezacord parțial
P229	Acord total	Acord total	Acord total	Acord total	Acord total	Acord total	Acord total
P230	Acord parțial	Acord total	Acord parțial	Dezacord parțial	Dezacord parțial	Dezacord total	Acord total
P231	Acord total	Acord total	Acord total	Acord total	Dezacord total	Dezacord total	Dezacord total
P232	Dezacord parțial	Dezacord total	Dezacord total	Dezacord parțial	Dezacord total	Dezacord total	Dezacord total
P233	Neutru	Dezacord parțial	Dezacord parțial	Dezacord parțial	Neutru	Neutru	Dezacord parțial
P234	Dezacord parțial	Acord parțial	Acord total	Acord parțial	Acord parțial	Dezacord total	Acord parțial
P235	Acord parțial	Acord parțial	Dezacord total	Acord parțial	Acord parțial	Dezacord total	Dezacord total
P236	Acord parțial	Acord total	Acord total	Acord total	Acord parțial	Acord parțial	Acord parțial
P237	Neutru	Dezacord parțial	Dezacord total	Dezacord total	Dezacord parțial	Neutru	Neutru
P238	Acord parțial	Acord parțial	Acord total	Acord parțial	Dezacord total	Dezacord total	Acord parțial
P239	Acord parțial	Acord total	Acord total	Acord total	Acord parțial	Acord parțial	Acord parțial
P240	Neutru	Dezacord parțial	Dezacord parțial	Neutru	Dezacord parțial	Dezacord parțial	Dezacord parțial
P241	Acord parțial	Acord total	Acord parțial	Acord parțial	Neutru	Neutru	Dezacord parțial
P242	Dezacord parțial	Dezacord parțial	Dezacord parțial	Dezacord parțial	Dezacord parțial	Dezacord parțial	Dezacord parțial
P243	Dezacord total	Acord total	Acord total	Acord total	Dezacord total	Dezacord total	Dezacord total
P244	Acord parțial	Acord total	Acord total	Acord total	Neutru	Dezacord total	Dezacord total

Participant_ID	D1	D2	D3	D4	D5	D6	D7
P245	Dezacord total	Dezacord parțial	Dezacord parțial	Neutru	Neutru	Dezacord total	Neutru
P246	Dezacord parțial	Dezacord parțial	Neutru	Dezacord parțial	Dezacord parțial	Dezacord parțial	Dezacord parțial
P247	Neutru	Acord total	Acord total	Neutru	Acord parțial	Neutru	Neutru
P248	Neutru	Neutru	Neutru	Neutru	Neutru	Neutru	Neutru
P249	Acord parțial	Acord parțial	Acord total	Acord total	Dezacord total	Dezacord total	Dezacord total
P250	Neutru	Neutru	Neutru	Neutru	Neutru	Dezacord parțial	Dezacord parțial
P251	Dezacord parțial	Neutru	Neutru	Neutru	Neutru	Neutru	Neutru
P252	Neutru	Dezacord parțial	Dezacord parțial	Neutru	Dezacord parțial	Dezacord parțial	Dezacord parțial
P253	Acord parțial	Acord parțial	Acord parțial	Neutru	Dezacord parțial	Dezacord total	Acord parțial
P254	Neutru	Dezacord parțial	Neutru	Neutru	Neutru	Neutru	Dezacord parțial
P255	Neutru	Dezacord total	Dezacord parțial	Neutru	Neutru	Neutru	Acord parțial
P256	Neutru	Dezacord total	Dezacord total	Dezacord total	Dezacord parțial	Dezacord parțial	Neutru
P257	Acord parțial	Acord parțial	Acord parțial	Acord parțial	Acord parțial	Dezacord total	Dezacord total
P258	Dezacord parțial	Dezacord parțial	Dezacord total	Dezacord parțial	Dezacord parțial	Dezacord parțial	Dezacord parțial
P259	Acord parțial	Acord total	Acord parțial	Acord parțial	Acord parțial	Acord parțial	Acord parțial
P260	Acord parțial	Acord total	Acord total	Acord parțial	Dezacord parțial	Neutru	Neutru
P261	Acord total	Acord total	Acord total	Acord parțial	Neutru	Neutru	Acord parțial
P262	Acord parțial	Acord parțial	Acord total	Acord total	Neutru	Neutru	Acord parțial
P263	Neutru	Dezacord parțial	Dezacord parțial	Neutru	Neutru	Dezacord parțial	Dezacord parțial
P264	Dezacord parțial	Acord parțial	Acord parțial	Acord parțial	Acord parțial	Acord parțial	Neutru
P265	Dezacord total	Dezacord total	Dezacord total	Dezacord total	Dezacord total	Dezacord parțial	Dezacord parțial
P266	Neutru	Acord total	Acord total	Acord parțial	Acord parțial	Acord parțial	Acord parțial
P267	Neutru	Dezacord total	Dezacord total	Acord parțial	Dezacord parțial	Neutru	Dezacord parțial
P268	Acord parțial	Neutru	Neutru	Neutru	Neutru	Neutru	Neutru
P269	Dezacord parțial	Dezacord total	Dezacord total	Dezacord total	Dezacord parțial	Dezacord parțial	Dezacord parțial
P270	Acord parțial	Acord total	Acord parțial	Acord parțial	Neutru	Dezacord total	Dezacord parțial
P271	Acord parțial	Acord parțial	Neutru	Acord parțial	Acord parțial	Acord parțial	Acord total
P272	Acord parțial	Acord parțial	Acord parțial	Neutru	Dezacord total	Neutru	Neutru
P273	Dezacord parțial	Dezacord total	Dezacord total	Dezacord parțial	Dezacord total	Dezacord parțial	Dezacord parțial
P274	Acord parțial	Neutru	Acord parțial	Dezacord parțial	Acord parțial	Dezacord parțial	Acord parțial
P275	Neutru	Acord total	Acord total	Neutru	Dezacord total	Neutru	Neutru
P276	Dezacord total	Acord parțial	Acord parțial	Dezacord parțial	Dezacord parțial	Dezacord parțial	Dezacord parțial
P277	Acord parțial	Acord parțial	Acord total	Acord total	Dezacord parțial	Neutru	Dezacord parțial
P278	Dezacord parțial	Neutru	Neutru	Neutru	Dezacord parțial	Dezacord total	Dezacord parțial
P279	Acord total	Acord total	Acord total	Acord total	Acord total	Dezacord total	Dezacord total
P280	Acord parțial	Acord parțial	Acord parțial	Acord parțial	Acord parțial	Acord parțial	Acord parțial
P281	Acord total	Acord parțial	Acord total	Acord total	Acord total	Dezacord total	Dezacord total
P282	Dezacord parțial	Dezacord parțial	Dezacord parțial	Dezacord parțial	Dezacord parțial	Dezacord parțial	Dezacord parțial
P283	Dezacord total	Neutru	Dezacord total	Dezacord total	Dezacord parțial	Dezacord parțial	Neutru
P284	Acord parțial	Acord total	Acord total	Acord total	Neutru	Dezacord parțial	Dezacord parțial
P285	Acord parțial	Acord parțial	Acord total	Acord total	Acord parțial	Dezacord total	Dezacord parțial
P286	Neutru	Neutru	Neutru	Neutru	Dezacord parțial	Neutru	Dezacord parțial
P287	Acord parțial	Acord parțial	Acord total	Acord total	Acord total	Dezacord parțial	Dezacord parțial
P288	Neutru	Neutru	Acord parțial	Acord parțial	Acord parțial	Neutru	Neutru
P289	Dezacord parțial	Neutru	Dezacord parțial	Dezacord parțial	Neutru	Neutru	Dezacord parțial
P290	Acord parțial	Acord total	Acord parțial	Acord total	Acord parțial	Neutru	Acord parțial
P291	Acord parțial	Acord parțial	Acord parțial	Acord parțial	Acord parțial	Dezacord total	Dezacord total
P292	Neutru	Neutru	Neutru	Neutru	Neutru	Neutru	Neutru
P293	Dezacord parțial	Dezacord parțial	Dezacord parțial	Dezacord parțial	Dezacord total	Dezacord parțial	Dezacord parțial
P294	Acord parțial	Acord parțial	Acord parțial	Neutru	Acord total	Neutru	Neutru
P295	Neutru	Acord parțial	Acord parțial	Acord parțial	Dezacord total	Neutru	Neutru
P296	Dezacord parțial	Neutru	Dezacord parțial	Neutru	Neutru	Neutru	Dezacord total
P297	Acord parțial	Acord total	Acord total	Acord total	Acord parțial	Acord total	Acord total
P298	Acord parțial	Acord total	Acord total	Acord parțial	Acord parțial	Acord total	Acord total
P299	Acord total	Acord total	Acord total	Acord total	Acord parțial	Acord total	Acord total
P300	Acord parțial	Acord parțial	Dezacord total	Dezacord total	Acord total	Acord total	Dezacord total
P301	Dezacord total	Acord total	Acord total	Acord total	Acord total	Acord parțial	Dezacord parțial

Panel E — Self-reported food-frequency and smoking behaviour

Participant_ID	E1	E2	E3	E4	E5	E6	E7	E8	E9	E10	E11	E12	E13
P001	Niciodată / aproape niciodată	Rar (sub o dată pe lună)	Zilnic	De câteva ori pe săptămână	Zilnic	Zilnic	Zilnic	De câteva ori pe săptămână	De câteva ori pe lună	De câteva ori pe lună	Niciodată / aproape niciodată	Zilnic	Nu fumez deloc
P002	De câteva ori pe lună	Rar (sub o dată pe lună)	De câteva ori pe săptămână	Rar (sub o dată pe lună)	De câteva ori pe săptămână	De câteva ori pe săptămână	De câteva ori pe săptămână	Zilnic	De câteva ori pe săptămână	De câteva ori pe săptămână	De câteva ori pe lună	Zilnic	Nu fumez deloc
P003	De câteva ori pe săptămână	Niciodată / aproape niciodată	De câteva ori pe săptămână	Niciodată / aproape niciodată	Niciodată / aproape niciodată	De câteva ori pe lună	De câteva ori pe săptămână	De câteva ori pe săptămână	De câteva ori pe lună	De câteva ori pe săptămână	Rar (sub o dată pe lună)	Zilnic	Nu fumez deloc
P004	Rar (sub o dată pe lună)	De câteva ori pe săptămână	Zilnic	Zilnic	Zilnic	Zilnic	Zilnic	Zilnic	Niciodată / aproape niciodată	Niciodată / aproape niciodată	Niciodată / aproape niciodată	Zilnic	Nu fumez deloc
P005	De câteva ori pe săptămână	De câteva ori pe săptămână	De câteva ori pe săptămână	Rar (sub o dată pe lună)	Zilnic	De câteva ori pe săptămână	Zilnic	Zilnic	Zilnic	Zilnic	Niciodată / aproape niciodată	De câteva ori pe lună	Nu fumez deloc
P006	De câteva ori pe lună	Rar (sub o dată pe lună)	De câteva ori pe săptămână	Rar (sub o dată pe lună)	De câteva ori pe săptămână	De câteva ori pe săptămână	De câteva ori pe săptămână	De câteva ori pe săptămână	Rar (sub o dată pe lună)	De câteva ori pe lună	Niciodată / aproape niciodată	De câteva ori pe săptămână	Nu fumez deloc
P007	De câteva ori pe săptămână	De câteva ori pe săptămână	De câteva ori pe săptămână	De câteva ori pe lună	De câteva ori pe săptămână	De câteva ori pe săptămână	Zilnic	De câteva ori pe săptămână	Zilnic	De câteva ori pe săptămână	De câteva ori pe lună	De câteva ori pe săptămână	Nu fumez deloc
P008	De câteva ori pe săptămână	De câteva ori pe lună	Zilnic	De câteva ori pe lună	Zilnic	De câteva ori pe lună	De câteva ori pe lună	Zilnic	De câteva ori pe lună	Zilnic	De câteva ori pe lună	Zilnic	Nu fumez deloc
P009	De câteva ori pe săptămână	De câteva ori pe lună	De câteva ori pe săptămână	Rar (sub o dată pe lună)	Zilnic	De câteva ori pe lună	Zilnic	Zilnic	De câteva ori pe lună	De câteva ori pe lună	Rar (sub o dată pe lună)	Zilnic	Nu fumez deloc
P010	De câteva ori pe lună	De câteva ori pe săptămână	Zilnic	De câteva ori pe săptămână	Zilnic	De câteva ori pe săptămână	De câteva ori pe lună	De câteva ori pe lună	De câteva ori pe săptămână	De câteva ori pe lună	Zilnic	Zilnic	Nu fumez deloc
P011	Zilnic	De câteva ori pe lună	De câteva ori pe săptămână	De câteva ori pe lună	De câteva ori pe lună	De câteva ori pe lună	De câteva ori pe săptămână	Zilnic	De câteva ori pe săptămână	De câteva ori pe săptămână	De câteva ori pe lună	Zilnic	Nu fumez deloc
P012	De câteva ori pe săptămână	De câteva ori pe săptămână	De câteva ori pe săptămână	De câteva ori pe lună	Zilnic	Zilnic	De câteva ori pe săptămână	Zilnic	Zilnic	De câteva ori pe săptămână	De câteva ori pe lună	Zilnic	Mai puțin de o țigară/zi (ocazional)
P013	Zilnic	Rar (sub o dată pe lună)	Zilnic	De câteva ori pe lună	Rar (sub o dată pe lună)	De câteva ori pe lună	Rar (sub o dată pe lună)	Zilnic	De câteva ori pe săptămână	Rar (sub o dată pe lună)	Niciodată / aproape niciodată	Rar (sub o dată pe lună)	Mai puțin de o țigară/zi (ocazional)
P014	Zilnic	De câteva ori pe săptămână	De câteva ori pe săptămână	De câteva ori pe lună	De câteva ori pe săptămână	De câteva ori pe săptămână	De câteva ori pe săptămână	De câteva ori pe săptămână	De câteva ori pe lună	De câteva ori pe lună	De câteva ori pe lună	Zilnic	Mai puțin de o țigară/zi (ocazional)
P015	De câteva ori pe lună	Zilnic	Zilnic	Rar (sub o dată pe lună)	De câteva ori pe lună	De câteva ori pe lună	De câteva ori pe săptămână	De câteva ori pe săptămână	Niciodată / aproape niciodată	De câteva ori pe lună	De câteva ori pe lună	Zilnic	11-20 țigări/zi
P016	De câteva ori pe săptămână	Zilnic	Zilnic	Niciodată / aproape niciodată	De câteva ori pe lună	De câteva ori pe lună	De câteva ori pe săptămână	De câteva ori pe săptămână	Zilnic	Niciodată / aproape niciodată	Niciodată / aproape niciodată	Zilnic	Nu fumez deloc
P017	De câteva ori pe lună	De câteva ori pe lună	De câteva ori pe săptămână	Rar (sub o dată pe lună)	De câteva ori pe săptămână	De câteva ori pe lună	De câteva ori pe săptămână	De câteva ori pe săptămână	De câteva ori pe lună	De câteva ori pe săptămână	Rar (sub o dată pe lună)	Zilnic	Peste 20 țigări/zi
P018	De câteva ori pe lună	Rar (sub o dată pe lună)	De câteva ori pe săptămână	Rar (sub o dată pe lună)	De câteva ori pe săptămână	De câteva ori pe lună	Zilnic	Zilnic	De câteva ori pe săptămână	De câteva ori pe săptămână	Niciodată / aproape niciodată	Zilnic	Nu fumez deloc
P019	De câteva ori pe săptămână	De câteva ori pe lună	De câteva ori pe săptămână	Rar (sub o dată pe lună)	De câteva ori pe săptămână	De câteva ori pe săptămână	De câteva ori pe săptămână	De câteva ori pe săptămână	De câteva ori pe lună	De câteva ori pe lună	Niciodată / aproape niciodată	Zilnic	Nu fumez deloc
P020	De câteva ori pe lună	De câteva ori pe lună	De câteva ori pe săptămână	De câteva ori pe lună	De câteva ori pe săptămână	De câteva ori pe săptămână	De câteva ori pe săptămână	De câteva ori pe săptămână	Zilnic	De câteva ori pe săptămână	Rar (sub o dată pe lună)	De câteva ori pe săptămână	1-10 țigări/zi
P021	De câteva ori pe lună	De câteva ori pe lună	De câteva ori pe săptămână	De câteva ori pe lună	Zilnic	De câteva ori pe săptămână	Zilnic	Zilnic	De câteva ori pe săptămână	De câteva ori pe lună	Rar (sub o dată pe lună)	Zilnic	Nu fumez deloc
P022	Rar (sub o dată pe lună)	De câteva ori pe lună	Zilnic	De câteva ori pe lună	De câteva ori pe lună	De câteva ori pe săptămână	Zilnic	Zilnic	De câteva ori pe lună	Zilnic	Niciodată / aproape niciodată	Zilnic	Nu fumez deloc
P023	De câteva ori pe săptămână	Rar (sub o dată pe lună)	De câteva ori pe săptămână	De câteva ori pe lună	De câteva ori pe săptămână	De câteva ori pe săptămână	De câteva ori pe săptămână	De câteva ori pe săptămână	De câteva ori pe săptămână	De câteva ori pe săptămână	Rar (sub o dată pe lună)	De câteva ori pe săptămână	11-20 țigări/zi
P024	De câteva ori pe lună	De câteva ori pe săptămână	Zilnic	De câteva ori pe lună	Zilnic	De câteva ori pe săptămână	De câteva ori pe săptămână	De câteva ori pe săptămână	De câteva ori pe săptămână	De câteva ori pe lună	Rar (sub o dată pe lună)	Zilnic	Nu fumez deloc
P025	De câteva ori pe săptămână	Zilnic	Zilnic	De câteva ori pe lună	De câteva ori pe săptămână	De câteva ori pe săptămână	Zilnic	De câteva ori pe săptămână	De câteva ori pe lună	Niciodată / aproape niciodată	Niciodată / aproape niciodată	Zilnic	Nu fumez deloc
P026	Rar (sub o dată pe lună)	De câteva ori pe lună	De câteva ori pe săptămână	De câteva ori pe lună	De câteva ori pe săptămână	De câteva ori pe lună	De câteva ori pe săptămână	Zilnic	De câteva ori pe săptămână	De câteva ori pe lună	Rar (sub o dată pe lună)	Zilnic	Nu fumez deloc
P027	De câteva ori pe lună	Rar (sub o dată pe lună)	De câteva ori pe săptămână	Rar (sub o dată pe lună)	De câteva ori pe săptămână	De câteva ori pe săptămână	De câteva ori pe săptămână	De câteva ori pe săptămână	Rar (sub o dată pe lună)	De câteva ori pe săptămână	Rar (sub o dată pe lună)	Zilnic	Nu fumez deloc
P028	De câteva ori pe săptămână	De câteva ori pe lună	Zilnic	De câteva ori pe lună	Zilnic	De câteva ori pe săptămână	Zilnic	De câteva ori pe săptămână	Zilnic	De câteva ori pe lună	De câteva ori pe lună	Zilnic	Nu fumez deloc
P029	Niciodată / aproape niciodată	De câteva ori pe săptămână	De câteva ori pe săptămână	Niciodată / aproape niciodată	Zilnic	De câteva ori pe lună	Zilnic	Zilnic	Zilnic	De câteva ori pe săptămână	Niciodată / aproape niciodată	Zilnic	Nu fumez deloc
P030	De câteva ori pe săptămână	Niciodată / aproape niciodată	Zilnic	De câteva ori pe săptămână	De câteva ori pe săptămână	De câteva ori pe săptămână	De câteva ori pe săptămână	De câteva ori pe săptămână	De câteva ori pe lună	De câteva ori pe lună	Niciodată / aproape niciodată	Zilnic	Nu fumez deloc
P031	De câteva ori pe lună	De câteva ori pe săptămână	De câteva ori pe lună	Niciodată / aproape niciodată	Niciodată / aproape niciodată	Rar (sub o dată pe lună)	De câteva ori pe săptămână	De câteva ori pe săptămână	De câteva ori pe lună	Rar (sub o dată pe lună)	Niciodată / aproape niciodată	De câteva ori pe săptămână	Nu fumez deloc
P032	De câteva ori pe lună	Rar (sub o dată pe lună)	De câteva ori pe săptămână	Niciodată / aproape niciodată	Zilnic	Zilnic	Zilnic	Zilnic	De câteva ori pe lună	Rar (sub o dată pe lună)	De câteva ori pe lună	Zilnic	Nu fumez deloc
P033	Zilnic	De câteva ori pe lună	De câteva ori pe săptămână	De câteva ori pe lună	De câteva ori pe lună	Zilnic	Zilnic	De câteva ori pe lună	De câteva ori pe lună	De câteva ori pe lună	De câteva ori pe lună	Zilnic	Mai puțin de o țigară/zi (ocazional)
P034	Zilnic	De câteva ori pe săptămână	De câteva ori pe săptămână	Niciodată / aproape niciodată	Zilnic	Zilnic	De câteva ori pe săptămână	Zilnic	De câteva ori pe săptămână	De câteva ori pe săptămână	De câteva ori pe lună	Zilnic	Peste 20 țigări/zi
P035	De câteva ori pe săptămână	De câteva ori pe săptămână	De câteva ori pe săptămână	De câteva ori pe lună	Zilnic	De câteva ori pe săptămână	De câteva ori pe săptămână	De câteva ori pe săptămână	Zilnic	Zilnic	Niciodată / aproape niciodată	Zilnic	Nu fumez deloc
P036	De câteva ori pe săptămână	De câteva ori pe săptămână	De câteva ori pe săptămână	Niciodată / aproape niciodată	Rar (sub o dată pe lună)	De câteva ori pe lună	De câteva ori pe lună	De câteva ori pe săptămână	De câteva ori pe săptămână	Rar (sub o dată pe lună)	Rar (sub o dată pe lună)	De câteva ori pe lună	Mai puțin de o țigară/zi (ocazional)
P037	De câteva ori pe săptămână	De câteva ori pe săptămână	De câteva ori pe săptămână	De câteva ori pe săptămână	Zilnic	De câteva ori pe lună	Zilnic	Zilnic	De câteva ori pe săptămână	De câteva ori pe săptămână	Rar (sub o dată pe lună)	Zilnic	Mai puțin de o țigară/zi (ocazional)
P038	De câteva ori pe săptămână	Rar (sub o dată pe lună)	De câteva ori pe săptămână	Rar (sub o dată pe lună)	Zilnic	De câteva ori pe săptămână	Zilnic	De câteva ori pe săptămână	De câteva ori pe săptămână	De câteva ori pe lună	De câteva ori pe lună	De câteva ori pe săptămână	1-10 țigări/zi
P039	De câteva ori pe săptămână	De câteva ori pe săptămână	Zilnic	De câteva ori pe lună	De câteva ori pe săptămână	De câteva ori pe săptămână	Zilnic	Zilnic	De câteva ori pe săptămână	De câteva ori pe săptămână	De câteva ori pe lună	Zilnic	Nu fumez deloc
P040	De câteva ori pe lună	De câteva ori pe lună	De câteva ori pe săptămână	De câteva ori pe lună	De câteva ori pe săptămână	De câteva ori pe săptămână	Zilnic	De câteva ori pe săptămână	Zilnic	Zilnic	De câteva ori pe lună	Zilnic	1-10 țigări/zi
P041	De câteva ori pe săptămână	De câteva ori pe lună	De câteva ori pe săptămână	Niciodată / aproape niciodată	De câteva ori pe săptămână	De câteva ori pe lună	De câteva ori pe săptămână	De câteva ori pe săptămână	Zilnic	Zilnic	Niciodată / aproape niciodată	Zilnic	Nu fumez deloc
P042	De câteva ori pe săptămână	De câteva ori pe lună	De câteva ori pe săptămână	De câteva ori pe lună	De câteva ori pe săptămână	De câteva ori pe săptămână	De câteva ori pe săptămână	De câteva ori pe săptămână	De câteva ori pe săptămână	De câteva ori pe săptămână	De câteva ori pe săptămână	Zilnic	1-10 țigări/zi
P043	Rar (sub o dată pe lună)	Rar (sub o dată pe lună)	De câteva ori pe săptămână	De câteva ori pe lună	De câteva ori pe săptămână	De câteva ori pe săptămână	Zilnic	Zilnic	Rar (sub o dată pe lună)	Rar (sub o dată pe lună)	Niciodată / aproape niciodată	Zilnic	1-10 țigări/zi
P044	Zilnic	De câteva ori pe lună	Zilnic	Zilnic	De câteva ori pe lună	De câteva ori pe săptămână	De câteva ori pe lună	De câteva ori pe săptămână	Zilnic	Zilnic	De câteva ori pe lună	Zilnic	1-10 țigări/zi
P045	De câteva ori pe săptămână	De câteva ori pe lună	De câteva ori pe săptămână	De câteva ori pe lună	De câteva ori pe lună	De câteva ori pe lună	De câteva ori pe săptămână	De câteva ori pe săptămână	De câteva ori pe săptămână	De câteva ori pe săptămână	Niciodată / aproape niciodată	Zilnic	Nu fumez deloc

Participant_ID	E1	E2	E3	E4	E5	E6	E7	E8	E9	E10	E11	E12	E13
P046	De câteva ori pe săptămână	Niciodată / aproape niciodată	De câteva ori pe săptămână	De câteva ori pe lună	De câteva ori pe săptămână	De câteva ori pe lună	Zilnic	De câteva ori pe săptămână	De câteva ori pe lună	De câteva ori pe lună	Niciodată / aproape niciodată	Zilnic	Nu fumez deloc
P047	Rar (sub o dată pe lună)	De câteva ori pe săptămână	Zilnic	De câteva ori pe săptămână	De câteva ori pe săptămână	De câteva ori pe săptămână	De câteva ori pe săptămână	De câteva ori pe lună	Zilnic	De câteva ori pe lună	Niciodată / aproape niciodată	Zilnic	Nu fumez deloc
P048	Zilnic	De câteva ori pe lună	Zilnic	De câteva ori pe lună	Zilnic	Zilnic	Zilnic	Zilnic	De câteva ori pe lună	Rar (sub o dată pe lună)	Niciodată / aproape niciodată	Zilnic	Nu fumez deloc
P049	De câteva ori pe săptămână	Niciodată / aproape niciodată	De câteva ori pe săptămână	De câteva ori pe lună	De câteva ori pe săptămână	Niciodată / aproape niciodată	Zilnic	Zilnic	De câteva ori pe săptămână	De câteva ori pe săptămână	Niciodată / aproape niciodată	Zilnic	Nu fumez deloc
P050	De câteva ori pe săptămână	Niciodată / aproape niciodată	De câteva ori pe lună	Rar (sub o dată pe lună)	De câteva ori pe lună	Rar (sub o dată pe lună)	De câteva ori pe lună	Rar (sub o dată pe lună)	De câteva ori pe săptămână	De câteva ori pe lună	Niciodată / aproape niciodată	Zilnic	1-10 țigări/zi
P051	Zilnic	De câteva ori pe săptămână	Zilnic	De câteva ori pe lună	De câteva ori pe lună	De câteva ori pe lună	De câteva ori pe lună	De câteva ori pe săptămână	Zilnic	De câteva ori pe săptămână	De câteva ori pe lună	De câteva ori pe săptămână	Nu fumez deloc
P052	De câteva ori pe lună	Rar (sub o dată pe lună)	Zilnic	De câteva ori pe lună	Rar (sub o dată pe lună)	De câteva ori pe săptămână	De câteva ori pe săptămână	Zilnic	De câteva ori pe lună	Rar (sub o dată pe lună)	Niciodată / aproape niciodată	Zilnic	Nu fumez deloc
P053	De câteva ori pe săptămână	De câteva ori pe săptămână	De câteva ori pe săptămână	De câteva ori pe săptămână	De câteva ori pe săptămână	De câteva ori pe săptămână	Zilnic	De câteva ori pe săptămână	De câteva ori pe săptămână	De câteva ori pe săptămână	De câteva ori pe lună	Zilnic	11-20 țigări/zi
P054	De câteva ori pe lună	De câteva ori pe săptămână	De câteva ori pe săptămână	De câteva ori pe săptămână	Zilnic	Zilnic	Zilnic	Zilnic	De câteva ori pe săptămână	De câteva ori pe săptămână	De câteva ori pe lună	Zilnic	Peste 20 țigări/zi
P055	De câteva ori pe lună	De câteva ori pe lună	De câteva ori pe lună	De câteva ori pe lună	De câteva ori pe săptămână	Rar (sub o dată pe lună)	Zilnic	De câteva ori pe săptămână	De câteva ori pe săptămână	De câteva ori pe săptămână	De câteva ori pe lună	Zilnic	1-10 țigări/zi
P056	De câteva ori pe lună	De câteva ori pe săptămână	De câteva ori pe săptămână	De câteva ori pe lună	Zilnic	De câteva ori pe lună	De câteva ori pe săptămână	De câteva ori pe săptămână	De câteva ori pe lună	Zilnic	De câteva ori pe lună	Zilnic	1-10 țigări/zi
P057	De câteva ori pe săptămână	Niciodată / aproape niciodată	De câteva ori pe săptămână	De câteva ori pe lună	Zilnic	De câteva ori pe săptămână	Zilnic	Zilnic	Zilnic	Zilnic	Niciodată / aproape niciodată	Zilnic	Nu fumez deloc
P058	De câteva ori pe lună	Rar (sub o dată pe lună)	De câteva ori pe săptămână	De câteva ori pe săptămână	Zilnic	De câteva ori pe săptămână	Zilnic	Zilnic	De câteva ori pe săptămână	Zilnic	Niciodată / aproape niciodată	Zilnic	Nu fumez deloc
P059	De câteva ori pe săptămână	De câteva ori pe lună	De câteva ori pe săptămână	De câteva ori pe lună	Zilnic	De câteva ori pe lună	Zilnic	De câteva ori pe săptămână	Zilnic	De câteva ori pe lună	De câteva ori pe lună	Zilnic	Nu fumez deloc
P060	De câteva ori pe săptămână	De câteva ori pe săptămână	Zilnic	De câteva ori pe săptămână	De câteva ori pe săptămână	Zilnic	Zilnic	De câteva ori pe săptămână	De câteva ori pe săptămână	Zilnic	De câteva ori pe săptămână	De câteva ori pe lună	Nu fumez deloc
P061	De câteva ori pe lună	De câteva ori pe săptămână	De câteva ori pe săptămână	De câteva ori pe săptămână	Zilnic	De câteva ori pe săptămână	Zilnic	De câteva ori pe săptămână	De câteva ori pe săptămână	Rar (sub o dată pe lună)	Rar (sub o dată pe lună)	Zilnic	Nu fumez deloc
P062	De câteva ori pe săptămână	De câteva ori pe lună	De câteva ori pe săptămână	De câteva ori pe lună	De câteva ori pe săptămână	De câteva ori pe săptămână	De câteva ori pe săptămână	De câteva ori pe săptămână	De câteva ori pe săptămână	Zilnic	Niciodată / aproape niciodată	Zilnic	Nu fumez deloc
P063	De câteva ori pe săptămână	De câteva ori pe lună	De câteva ori pe lună	De câteva ori pe lună	Zilnic	De câteva ori pe lună	De câteva ori pe lună	De câteva ori pe lună	Zilnic	Zilnic	Niciodată / aproape niciodată	Zilnic	11-20 țigări/zi
P064	De câteva ori pe săptămână	De câteva ori pe lună	De câteva ori pe săptămână	Rar (sub o dată pe lună)	Zilnic	Zilnic	Zilnic	De câteva ori pe săptămână	De câteva ori pe săptămână	De câteva ori pe săptămână	Niciodată / aproape niciodată	De câteva ori pe săptămână	Nu fumez deloc
P065	De câteva ori pe lună	De câteva ori pe lună	De câteva ori pe săptămână	De câteva ori pe lună	De câteva ori pe săptămână	De câteva ori pe lună	De câteva ori pe săptămână	De câteva ori pe săptămână	Rar (sub o dată pe lună)	De câteva ori pe săptămână	Niciodată / aproape niciodată	Zilnic	1-10 țigări/zi
P066	De câteva ori pe lună	Rar (sub o dată pe lună)	Zilnic	De câteva ori pe lună	De câteva ori pe lună	De câteva ori pe săptămână	De câteva ori pe săptămână	De câteva ori pe lună	De câteva ori pe săptămână	De câteva ori pe săptămână	Rar (sub o dată pe lună)	Zilnic	Mai puțin de o țigară/zi (ocazional)
P067	Niciodată / aproape niciodată	De câteva ori pe săptămână	De câteva ori pe lună	Niciodată / aproape niciodată	Zilnic	De câteva ori pe lună	Zilnic	Zilnic	Niciodată / aproape niciodată	Niciodată / aproape niciodată	Niciodată / aproape niciodată	Zilnic	Nu fumez deloc
P068	De câteva ori pe lună	De câteva ori pe săptămână	De câteva ori pe săptămână	Rar (sub o dată pe lună)	Rar (sub o dată pe lună)	De câteva ori pe lună	Zilnic	De câteva ori pe săptămână	De câteva ori pe săptămână	De câteva ori pe lună	Niciodată / aproape niciodată	De câteva ori pe săptămână	Nu fumez deloc
P069	De câteva ori pe lună	De câteva ori pe săptămână	De câteva ori pe săptămână	De câteva ori pe lună	De câteva ori pe săptămână	De câteva ori pe săptămână	Zilnic	Zilnic	De câteva ori pe lună	Rar (sub o dată pe lună)	Niciodată / aproape niciodată	Zilnic	Nu fumez deloc
P070	De câteva ori pe săptămână	De câteva ori pe săptămână	Zilnic	De câteva ori pe lună	Zilnic	De câteva ori pe săptămână	Zilnic	Zilnic	De câteva ori pe lună	Rar (sub o dată pe lună)	Niciodată / aproape niciodată	Zilnic	Mai puțin de o țigară/zi (ocazional)
P071	De câteva ori pe lună	De câteva ori pe săptămână	De câteva ori pe săptămână	De câteva ori pe lună	Rar (sub o dată pe lună)	De câteva ori pe săptămână	De câteva ori pe săptămână	De câteva ori pe săptămână	De câteva ori pe lună	Rar (sub o dată pe lună)	Rar (sub o dată pe lună)	Zilnic	Nu fumez deloc
P072	De câteva ori pe lună	Rar (sub o dată pe lună)	De câteva ori pe lună	De câteva ori pe lună	De câteva ori pe săptămână	De câteva ori pe săptămână	Zilnic	Zilnic	De câteva ori pe lună	Niciodată / aproape niciodată	Niciodată / aproape niciodată	Zilnic	Nu fumez deloc
P073	De câteva ori pe săptămână	De câteva ori pe săptămână	Zilnic	De câteva ori pe lună	Zilnic	De câteva ori pe săptămână	De câteva ori pe săptămână	De câteva ori pe săptămână	Zilnic	Zilnic	De câteva ori pe lună	Zilnic	Nu fumez deloc
P074	De câteva ori pe săptămână	Niciodată / aproape niciodată	De câteva ori pe lună	De câteva ori pe lună	Zilnic	Zilnic	Zilnic	De câteva ori pe săptămână	De câteva ori pe lună	De câteva ori pe lună	Rar (sub o dată pe lună)	Zilnic	Mai puțin de o țigară/zi (ocazional)
P075	Zilnic	Niciodată / aproape niciodată	Zilnic	De câteva ori pe lună	Zilnic	Zilnic	Zilnic	Zilnic	Rar (sub o dată pe lună)	Niciodată / aproape niciodată	Niciodată / aproape niciodată	Zilnic	Nu fumez deloc
P076	De câteva ori pe lună	De câteva ori pe lună	Zilnic	De câteva ori pe săptămână	De câteva ori pe săptămână	Zilnic	Zilnic	Zilnic	De câteva ori pe lună	De câteva ori pe lună	Rar (sub o dată pe lună)	Zilnic	Nu fumez deloc
P077	De câteva ori pe lună	Rar (sub o dată pe lună)	De câteva ori pe săptămână	De câteva ori pe lună	De câteva ori pe săptămână	De câteva ori pe săptămână	De câteva ori pe săptămână	De câteva ori pe săptămână	De câteva ori pe săptămână	De câteva ori pe lună	Niciodată / aproape niciodată	Zilnic	Nu fumez deloc
P078	De câteva ori pe lună	De câteva ori pe lună	De câteva ori pe lună	De câteva ori pe lună	De câteva ori pe săptămână	Rar (sub o dată pe lună)	De câteva ori pe lună	De câteva ori pe săptămână	De câteva ori pe săptămână	De câteva ori pe săptămână	Zilnic	Niciodată / aproape niciodată	Nu fumez deloc
P079	De câteva ori pe săptămână	De câteva ori pe lună	De câteva ori pe săptămână	De câteva ori pe lună	Zilnic	De câteva ori pe săptămână	Zilnic	Zilnic	De câteva ori pe săptămână	De câteva ori pe lună	Rar (sub o dată pe lună)	Zilnic	Nu fumez deloc
P080	De câteva ori pe săptămână	De câteva ori pe săptămână	De câteva ori pe săptămână	De câteva ori pe lună	Zilnic	De câteva ori pe săptămână	Zilnic	Zilnic	Zilnic	De câteva ori pe săptămână	De câteva ori pe săptămână	Zilnic	Nu fumez deloc
P081	De câteva ori pe săptămână	Rar (sub o dată pe lună)	De câteva ori pe săptămână	De câteva ori pe săptămână	Zilnic	Zilnic	Zilnic	Zilnic	De câteva ori pe săptămână	De câteva ori pe lună	Niciodată / aproape niciodată	Zilnic	11-20 țigări/zi
P082	De câteva ori pe lună	De câteva ori pe lună	De câteva ori pe săptămână	Rar (sub o dată pe lună)	De câteva ori pe lună	Rar (sub o dată pe lună)	De câteva ori pe săptămână	De câteva ori pe săptămână	Rar (sub o dată pe lună)	De câteva ori pe lună	Rar (sub o dată pe lună)	Zilnic	11-20 țigări/zi
P083	Rar (sub o dată pe lună)	De câteva ori pe lună	De câteva ori pe săptămână	De câteva ori pe lună	De câteva ori pe săptămână	De câteva ori pe săptămână	De câteva ori pe lună	De câteva ori pe săptămână	Rar (sub o dată pe lună)	De câteva ori pe lună	Niciodată / aproape niciodată	Zilnic	11-20 țigări/zi
P084	De câteva ori pe lună	Rar (sub o dată pe lună)	De câteva ori pe lună	Niciodată / aproape niciodată	De câteva ori pe săptămână	De câteva ori pe săptămână	De câteva ori pe săptămână	De câteva ori pe săptămână	De câteva ori pe lună	Rar (sub o dată pe lună)	Niciodată / aproape niciodată	Zilnic	Nu fumez deloc
P085	De câteva ori pe lună	De câteva ori pe lună	De câteva ori pe lună	Rar (sub o dată pe lună)	De câteva ori pe săptămână	De câteva ori pe lună	De câteva ori pe săptămână	Zilnic	De câteva ori pe săptămână	De câteva ori pe săptămână	Rar (sub o dată pe lună)	Zilnic	Nu fumez deloc
P086	De câteva ori pe săptămână	De câteva ori pe săptămână	De câteva ori pe săptămână	De câteva ori pe săptămână	De câteva ori pe săptămână	De câteva ori pe săptămână	De câteva ori pe săptămână	De câteva ori pe săptămână	De câteva ori pe săptămână	Zilnic	De câteva ori pe lună	De câteva ori pe săptămână	Nu fumez deloc
P087	De câteva ori pe lună	De câteva ori pe lună	Zilnic	Niciodată / aproape niciodată	De câteva ori pe săptămână	De câteva ori pe lună	De câteva ori pe săptămână	Zilnic	De câteva ori pe lună	Rar (sub o dată pe lună)	De câteva ori pe lună	Zilnic	1-10 țigări/zi
P088	De câteva ori pe lună	De câteva ori pe săptămână	De câteva ori pe săptămână	De câteva ori pe săptămână	Zilnic	De câteva ori pe lună	Zilnic	Zilnic	De câteva ori pe săptămână	De câteva ori pe săptămână	Niciodată / aproape niciodată	Zilnic	Nu fumez deloc
P089	De câteva ori pe săptămână	De câteva ori pe săptămână	De câteva ori pe săptămână	De câteva ori pe lună	Zilnic	Zilnic	Zilnic	Zilnic	Zilnic	Zilnic	Zilnic	Zilnic	1-10 țigări/zi
P090	De câteva ori pe săptămână	De câteva ori pe lună	De câteva ori pe săptămână	De câteva ori pe lună	Zilnic	De câteva ori pe lună	De câteva ori pe săptămână	De câteva ori pe săptămână	Zilnic	De câteva ori pe lună	De câteva ori pe săptămână	Zilnic	1-10 țigări/zi
P091	Rar (sub o dată pe lună)	De câteva ori pe săptămână	De câteva ori pe săptămână	De câteva ori pe lună	Zilnic	Zilnic	Zilnic	Zilnic	Zilnic	De câteva ori pe săptămână	Rar (sub o dată pe lună)	Zilnic	Nu fumez deloc

Participant_ ID	E1	E2	E3	E4	E5	E6	E7	E8	E9	E10	E11	E12	E13
P092	Zilnic	De câteva ori pe săptămână	De câteva ori pe săptămână	De câteva ori pe lună	Zilnic	De câteva ori pe lună	De câteva ori pe săptămână	De câteva ori pe lună	Zilnic	De câteva ori pe lună	De câteva ori pe lună	De câteva ori pe săptămână	1-10 țigări/zi
P093	De câteva ori pe săptămână	Zilnic	Zilnic	De câteva ori pe lună	Zilnic	Zilnic	Zilnic	Zilnic	Zilnic	Zilnic	De câteva ori pe săptămână	Zilnic	11-20 țigări/zi
P094	De câteva ori pe săptămână	De câteva ori pe lună	De câteva ori pe săptămână	De câteva ori pe lună	Zilnic	De câteva ori pe săptămână	De câteva ori pe săptămână	Zilnic	Zilnic	De câteva ori pe săptămână	De câteva ori pe lună	Zilnic	1-10 țigări/zi
P095	De câteva ori pe săptămână	Rar (sub o dată pe lună)	De câteva ori pe săptămână	Niciodată / aproape niciodată	Zilnic	De câteva ori pe săptămână	De câteva ori pe săptămână	De câteva ori pe săptămână	De câteva ori pe lună	Rar (sub o dată pe lună)	Rar (sub o dată pe lună)	Zilnic	11-20 țigări/zi
P096	De câteva ori pe săptămână	De câteva ori pe săptămână	De câteva ori pe săptămână	De câteva ori pe săptămână	De câteva ori pe săptămână	De câteva ori pe săptămână	Zilnic	De câteva ori pe săptămână	De câteva ori pe săptămână	De câteva ori pe lună	De câteva ori pe lună	De câteva ori pe săptămână	Nu fumez deloc
P097	De câteva ori pe săptămână	De câteva ori pe săptămână	De câteva ori pe săptămână	De câteva ori pe săptămână	De câteva ori pe săptămână	De câteva ori pe săptămână	De câteva ori pe săptămână	De câteva ori pe săptămână	De câteva ori pe săptămână	De câteva ori pe lună	De câteva ori pe săptămână	Zilnic	Nu fumez deloc
P098	De câteva ori pe săptămână	De câteva ori pe săptămână	De câteva ori pe săptămână	De câteva ori pe săptămână	De câteva ori pe săptămână	De câteva ori pe săptămână	De câteva ori pe săptămână	De câteva ori pe săptămână	De câteva ori pe lună	De câteva ori pe lună	De câteva ori pe lună	De câteva ori pe săptămână	Nu fumez deloc
P099	Rar (sub o dată pe lună)	De câteva ori pe lună	De câteva ori pe săptămână	De câteva ori pe lună	De câteva ori pe săptămână	De câteva ori pe săptămână	De câteva ori pe săptămână	De câteva ori pe săptămână	De câteva ori pe lună	Rar (sub o dată pe lună)	Rar (sub o dată pe lună)	Zilnic	Nu fumez deloc
P100	De câteva ori pe lună	De câteva ori pe lună	Zilnic	Niciodată / aproape niciodată	De câteva ori pe săptămână	De câteva ori pe lună	Zilnic	Zilnic	De câteva ori pe săptămână	De câteva ori pe lună	Niciodată / aproape niciodată	Zilnic	Nu fumez deloc
P101	De câteva ori pe lună	De câteva ori pe lună	De câteva ori pe săptămână	De câteva ori pe săptămână	Zilnic	Zilnic	De câteva ori pe lună	De câteva ori pe săptămână	De câteva ori pe săptămână	De câteva ori pe lună	Niciodată / aproape niciodată	Zilnic	Nu fumez deloc
P102	De câteva ori pe săptămână	De câteva ori pe lună	De câteva ori pe săptămână	De câteva ori pe lună	Zilnic	De câteva ori pe lună	Zilnic	De câteva ori pe săptămână	De câteva ori pe lună	Rar (sub o dată pe lună)	Niciodată / aproape niciodată	Zilnic	Nu fumez deloc
P103	De câteva ori pe săptămână	De câteva ori pe săptămână	De câteva ori pe săptămână	De câteva ori pe săptămână	De câteva ori pe săptămână	De câteva ori pe săptămână	De câteva ori pe săptămână	De câteva ori pe săptămână	De câteva ori pe săptămână	De câteva ori pe săptămână	De câteva ori pe lună	Zilnic	Nu fumez deloc
P104	De câteva ori pe lună	De câteva ori pe lună	De câteva ori pe săptămână	De câteva ori pe lună	De câteva ori pe săptămână	De câteva ori pe săptămână	Zilnic	Zilnic	De câteva ori pe lună	De câteva ori pe lună	De câteva ori pe lună	Zilnic	Nu fumez deloc
P105	De câteva ori pe lună	De câteva ori pe lună	Zilnic	De câteva ori pe săptămână	De câteva ori pe săptămână	Zilnic	Zilnic	Zilnic	De câteva ori pe lună	Rar (sub o dată pe lună)	De câteva ori pe lună	Zilnic	1-10 țigări/zi
P106	De câteva ori pe săptămână	De câteva ori pe lună	De câteva ori pe săptămână	Rar (sub o dată pe lună)	De câteva ori pe săptămână	Zilnic	Zilnic	De câteva ori pe săptămână	De câteva ori pe săptămână	De câteva ori pe săptămână	Rar (sub o dată pe lună)	Zilnic	1-10 țigări/zi
P107	De câteva ori pe săptămână	De câteva ori pe săptămână	De câteva ori pe săptămână	De câteva ori pe săptămână	Zilnic	De câteva ori pe săptămână	Zilnic	De câteva ori pe săptămână	De câteva ori pe săptămână	De câteva ori pe săptămână	De câteva ori pe lună	Zilnic	Nu fumez deloc
P108	De câteva ori pe săptămână	Rar (sub o dată pe lună)	Zilnic	Niciodată / aproape niciodată	Niciodată / aproape niciodată	De câteva ori pe săptămână	De câteva ori pe săptămână	Zilnic	De câteva ori pe săptămână	De câteva ori pe săptămână	Rar (sub o dată pe lună)	De câteva ori pe săptămână	Nu fumez deloc
P109	De câteva ori pe lună	Zilnic	Zilnic	De câteva ori pe lună	Zilnic	De câteva ori pe lună	De câteva ori pe săptămână	De câteva ori pe săptămână	Rar (sub o dată pe lună)	Niciodată / aproape niciodată	Niciodată / aproape niciodată	Zilnic	Nu fumez deloc
P110	De câteva ori pe săptămână	De câteva ori pe lună	De câteva ori pe săptămână	De câteva ori pe lună	De câteva ori pe săptămână	De câteva ori pe săptămână	Zilnic	Zilnic	De câteva ori pe săptămână	De câteva ori pe săptămână	De câteva ori pe lună	De câteva ori pe săptămână	Nu fumez deloc
P111	De câteva ori pe lună	De câteva ori pe săptămână	Zilnic	De câteva ori pe lună	De câteva ori pe săptămână	Zilnic	Zilnic	Zilnic	De câteva ori pe săptămână	De câteva ori pe săptămână	De câteva ori pe lună	Zilnic	Nu fumez deloc
P112	De câteva ori pe săptămână	De câteva ori pe lună	De câteva ori pe săptămână	Rar (sub o dată pe lună)	De câteva ori pe săptămână	De câteva ori pe lună	De câteva ori pe săptămână	De câteva ori pe lună	De câteva ori pe săptămână	De câteva ori pe lună	De câteva ori pe săptămână	Zilnic	Nu fumez deloc
P113	De câteva ori pe lună	De câteva ori pe lună	Zilnic	De câteva ori pe săptămână	De câteva ori pe săptămână	De câteva ori pe săptămână	Zilnic	Zilnic	De câteva ori pe lună	Rar (sub o dată pe lună)	Rar (sub o dată pe lună)	Zilnic	Nu fumez deloc
P114	Rar (sub o dată pe lună)	Rar (sub o dată pe lună)	Zilnic	De câteva ori pe lună	Zilnic	Zilnic	Zilnic	Zilnic	Niciodată / aproape niciodată	Niciodată / aproape niciodată	Niciodată / aproape niciodată	Zilnic	Nu fumez deloc
P115	Niciodată / aproape niciodată	De câteva ori pe lună	De câteva ori pe săptămână	De câteva ori pe săptămână	De câteva ori pe săptămână	Zilnic	Zilnic	Zilnic	Niciodată / aproape niciodată	Niciodată / aproape niciodată	De câteva ori pe săptămână	Zilnic	Peste 20 țigări/zi
P116	De câteva ori pe lună	Zilnic	De câteva ori pe săptămână	Rar (sub o dată pe lună)	Rar (sub o dată pe lună)	De câteva ori pe săptămână	Zilnic	Zilnic	De câteva ori pe săptămână	De câteva ori pe săptămână	Rar (sub o dată pe lună)	De câteva ori pe săptămână	Nu fumez deloc
P117	De câteva ori pe săptămână	De câteva ori pe săptămână	De câteva ori pe săptămână	De câteva ori pe săptămână	De câteva ori pe săptămână	De câteva ori pe săptămână	De câteva ori pe săptămână	De câteva ori pe săptămână	De câteva ori pe lună	De câteva ori pe lună	De câteva ori pe săptămână	De câteva ori pe săptămână	1-10 țigări/zi
P118	Rar (sub o dată pe lună)	De câteva ori pe lună	De câteva ori pe săptămână	De câteva ori pe săptămână	Zilnic	De câteva ori pe săptămână	De câteva ori pe săptămână	Zilnic	De câteva ori pe lună	De câteva ori pe lună	De câteva ori pe lună	Zilnic	1-10 țigări/zi
P119	Rar (sub o dată pe lună)	Rar (sub o dată pe lună)	De câteva ori pe săptămână	De câteva ori pe săptămână	De câteva ori pe săptămână	De câteva ori pe lună	Zilnic	De câteva ori pe săptămână	De câteva ori pe lună	Rar (sub o dată pe lună)	Rar (sub o dată pe lună)	Zilnic	Nu fumez deloc
P120	De câteva ori pe lună	De câteva ori pe săptămână	De câteva ori pe săptămână	De câteva ori pe lună	Zilnic	De câteva ori pe săptămână	Zilnic	Zilnic	De câteva ori pe lună	Niciodată / aproape niciodată	Rar (sub o dată pe lună)	Zilnic	Nu fumez deloc
P121	De câteva ori pe lună	De câteva ori pe săptămână	De câteva ori pe săptămână	De câteva ori pe săptămână	Zilnic	Zilnic	Zilnic	Zilnic	De câteva ori pe săptămână	De câteva ori pe săptămână	De câteva ori pe lună	Zilnic	Nu fumez deloc
P122	De câteva ori pe lună	De câteva ori pe săptămână	De câteva ori pe săptămână	Rar (sub o dată pe lună)	De câteva ori pe lună	De câteva ori pe lună	Zilnic	Zilnic	Zilnic	Zilnic	Niciodată / aproape niciodată	Zilnic	Nu fumez deloc
P123	De câteva ori pe săptămână	Zilnic	De câteva ori pe săptămână	De câteva ori pe lună	De câteva ori pe săptămână	De câteva ori pe lună	De câteva ori pe lună	Zilnic	Rar (sub o dată pe lună)	Niciodată / aproape niciodată	De câteva ori pe săptămână	Zilnic	Nu fumez deloc
P124	Rar (sub o dată pe lună)	De câteva ori pe lună	De câteva ori pe săptămână	Rar (sub o dată pe lună)	Niciodată / aproape niciodată	De câteva ori pe lună	De câteva ori pe săptămână	De câteva ori pe săptămână	De câteva ori pe săptămână	Rar (sub o dată pe lună)	Rar (sub o dată pe lună)	Zilnic	Nu fumez deloc
P125	De câteva ori pe săptămână	De câteva ori pe săptămână	De câteva ori pe lună	De câteva ori pe lună	Zilnic	De câteva ori pe lună	De câteva ori pe săptămână	De câteva ori pe săptămână	Zilnic	De câteva ori pe lună	Rar (sub o dată pe lună)	Zilnic	Nu fumez deloc
P126	De câteva ori pe lună	De câteva ori pe lună	De câteva ori pe săptămână	De câteva ori pe lună	Zilnic	De câteva ori pe săptămână	Zilnic	Zilnic	De câteva ori pe lună	De câteva ori pe lună	De câteva ori pe lună	Zilnic	Nu fumez deloc
P127	De câteva ori pe săptămână	Niciodată / aproape niciodată	De câteva ori pe săptămână	Niciodată / aproape niciodată	De câteva ori pe lună	De câteva ori pe lună	De câteva ori pe lună	De câteva ori pe lună	Zilnic	De câteva ori pe lună	Niciodată / aproape niciodată	Zilnic	Mai puțin de o țigară/zi (ocazional)
P128	De câteva ori pe săptămână	De câteva ori pe lună	De câteva ori pe săptămână	De câteva ori pe săptămână	De câteva ori pe săptămână	De câteva ori pe săptămână	De câteva ori pe săptămână	De câteva ori pe săptămână	Rar (sub o dată pe lună)	Niciodată / aproape niciodată	Niciodată / aproape niciodată	Zilnic	Nu fumez deloc
P129	De câteva ori pe lună	De câteva ori pe săptămână	De câteva ori pe săptămână	De câteva ori pe lună	De câteva ori pe săptămână	De câteva ori pe săptămână	Zilnic	Zilnic	De câteva ori pe săptămână	De câteva ori pe lună	De câteva ori pe lună	Zilnic	Nu fumez deloc
P130	De câteva ori pe lună	De câteva ori pe lună	De câteva ori pe săptămână	De câteva ori pe lună	De câteva ori pe săptămână	De câteva ori pe lună	Zilnic	Zilnic	De câteva ori pe lună	Rar (sub o dată pe lună)	Niciodată / aproape niciodată	Zilnic	Nu fumez deloc
P131	Rar (sub o dată pe lună)	De câteva ori pe săptămână	De câteva ori pe săptămână	Rar (sub o dată pe lună)	De câteva ori pe săptămână	De câteva ori pe lună	Zilnic	Zilnic	De câteva ori pe lună	Rar (sub o dată pe lună)	Niciodată / aproape niciodată	De câteva ori pe săptămână	Nu fumez deloc
P132	De câteva ori pe lună	De câteva ori pe lună	Zilnic	De câteva ori pe lună	De câteva ori pe săptămână	De câteva ori pe lună	De câteva ori pe săptămână	De câteva ori pe lună	Zilnic	De câteva ori pe săptămână	De câteva ori pe lună	De câteva ori pe lună	Mai puțin de o țigară/zi (ocazional)
P133	Rar (sub o dată pe lună)	Rar (sub o dată pe lună)	Rar (sub o dată pe lună)	De câteva ori pe lună	Zilnic	De câteva ori pe lună	Zilnic	Zilnic	Rar (sub o dată pe lună)	Rar (sub o dată pe lună)	Niciodată / aproape niciodată	Zilnic	Nu fumez deloc
P134	Zilnic	De câteva ori pe săptămână	Zilnic	De câteva ori pe lună	De câteva ori pe săptămână	De câteva ori pe săptămână	De câteva ori pe săptămână	De câteva ori pe săptămână	De câteva ori pe săptămână	De câteva ori pe săptămână	De câteva ori pe lună	De câteva ori pe săptămână	Nu fumez deloc
P135	De câteva ori pe săptămână	De câteva ori pe săptămână	De câteva ori pe săptămână	De câteva ori pe săptămână	De câteva ori pe săptămână	De câteva ori pe săptămână	De câteva ori pe săptămână	De câteva ori pe săptămână	De câteva ori pe săptămână	De câteva ori pe săptămână	De câteva ori pe săptămână	De câteva ori pe săptămână	Nu fumez deloc
P136	De câteva ori pe lună	De câteva ori pe lună	De câteva ori pe săptămână	De câteva ori pe săptămână	De câteva ori pe săptămână	De câteva ori pe săptămână	De câteva ori pe săptămână	De câteva ori pe săptămână	De câteva ori pe săptămână	De câteva ori pe lună	De câteva ori pe lună	De câteva ori pe lună	1-10 țigări/zi
P137	De câteva ori pe lună	De câteva ori pe săptămână	Zilnic	De câteva ori pe săptămână	De câteva ori pe săptămână	Zilnic	De câteva ori pe săptămână	De câteva ori pe săptămână	De câteva ori pe lună	De câteva ori pe lună	De câteva ori pe săptămână	De câteva ori pe săptămână	11-20 țigări/zi

Participant_ID	E1	E2	E3	E4	E5	E6	E7	E8	E9	E10	E11	E12	E13
P138	Rar (sub o dată pe lună)	De câteva ori pe săptămână	De câteva ori pe săptămână	De câteva ori pe săptămână	Zilnic	De câteva ori pe săptămână	Zilnic	De câteva ori pe săptămână	Rar (sub o dată pe lună)	Rar (sub o dată pe lună)	De câteva ori pe lună	Zilnic	Nu fumez deloc
P139	De câteva ori pe lună	De câteva ori pe lună	De câteva ori pe lună	De câteva ori pe lună	De câteva ori pe săptămână	De câteva ori pe săptămână	Zilnic	Zilnic	De câteva ori pe săptămână	De câteva ori pe săptămână	De câteva ori pe lună	Zilnic	Nu fumez deloc
P140	De câteva ori pe săptămână	De câteva ori pe săptămână	De câteva ori pe săptămână	De câteva ori pe lună	De câteva ori pe săptămână	De câteva ori pe săptămână	De câteva ori pe săptămână	De câteva ori pe săptămână	De câteva ori pe săptămână	De câteva ori pe săptămână	De câteva ori pe săptămână	Zilnic	11-20 țigări/zi
P141	Rar (sub o dată pe lună)	De câteva ori pe săptămână	De câteva ori pe săptămână	De câteva ori pe săptămână	De câteva ori pe săptămână	De câteva ori pe lună	Zilnic	De câteva ori pe săptămână	Zilnic	De câteva ori pe lună	Niciodată / aproape niciodată	De câteva ori pe săptămână	Nu fumez deloc
P142	Rar (sub o dată pe lună)	Niciodată / aproape niciodată	Niciodată / aproape niciodată	Zilnic	Zilnic	Zilnic	De câteva ori pe lună	De câteva ori pe săptămână	De câteva ori pe lună	Rar (sub o dată pe lună)	De câteva ori pe săptămână	Zilnic	11-20 țigări/zi
P143	De câteva ori pe săptămână	De câteva ori pe lună	De câteva ori pe săptămână	De câteva ori pe săptămână	De câteva ori pe săptămână	De câteva ori pe săptămână	Zilnic	Zilnic	De câteva ori pe lună	De câteva ori pe lună	De câteva ori pe lună	Zilnic	1-10 țigări/zi
P144	De câteva ori pe săptămână	De câteva ori pe săptămână	De câteva ori pe săptămână	De câteva ori pe lună	De câteva ori pe lună	De câteva ori pe lună	Zilnic	De câteva ori pe săptămână	De câteva ori pe lună	De câteva ori pe lună	De câteva ori pe lună	De câteva ori pe săptămână	Nu fumez deloc
P145	De câteva ori pe săptămână	De câteva ori pe săptămână	De câteva ori pe săptămână	De câteva ori pe lună	Niciodată / aproape niciodată	Rar (sub o dată pe lună)	De câteva ori pe săptămână	De câteva ori pe săptămână	Zilnic	De câteva ori pe săptămână	Niciodată / aproape niciodată	De câteva ori pe săptămână	Peste 20 țigări/zi
P146	Niciodată / aproape niciodată	De câteva ori pe săptămână	De câteva ori pe săptămână	De câteva ori pe săptămână	De câteva ori pe săptămână	Zilnic	Zilnic	Zilnic	Niciodată / aproape niciodată	Niciodată / aproape niciodată	Niciodată / aproape niciodată	Zilnic	1-10 țigări/zi
P147	De câteva ori pe lună	De câteva ori pe lună	De câteva ori pe lună	Rar (sub o dată pe lună)	De câteva ori pe săptămână	De câteva ori pe săptămână	Zilnic	Zilnic	De câteva ori pe săptămână	Niciodată / aproape niciodată	De câteva ori pe lună	Zilnic	1-10 țigări/zi
P148	De câteva ori pe săptămână	De câteva ori pe săptămână	De câteva ori pe săptămână	De câteva ori pe lună	De câteva ori pe săptămână	De câteva ori pe săptămână	De câteva ori pe săptămână	De câteva ori pe săptămână	De câteva ori pe lună	De câteva ori pe săptămână	Rar (sub o dată pe lună)	Zilnic	Nu fumez deloc
P149	De câteva ori pe lună	De câteva ori pe lună	De câteva ori pe săptămână	De câteva ori pe lună	De câteva ori pe lună	De câteva ori pe săptămână	De câteva ori pe lună	De câteva ori pe lună	De câteva ori pe săptămână	De câteva ori pe săptămână	De câteva ori pe lună	Zilnic	Nu fumez deloc
P150	De câteva ori pe lună	De câteva ori pe săptămână	Zilnic	Zilnic	Zilnic	Zilnic	Zilnic	Zilnic	De câteva ori pe săptămână	Zilnic	De câteva ori pe lună	Zilnic	Nu fumez deloc
P151	Zilnic	De câteva ori pe săptămână	De câteva ori pe săptămână	De câteva ori pe lună	De câteva ori pe săptămână	De câteva ori pe săptămână	De câteva ori pe săptămână	Zilnic	De câteva ori pe lună	De câteva ori pe săptămână	Niciodată / aproape niciodată	De câteva ori pe lună	Nu fumez deloc
P152	De câteva ori pe săptămână	De câteva ori pe săptămână	De câteva ori pe săptămână	Rar (sub o dată pe lună)	De câteva ori pe săptămână	De câteva ori pe lună	De câteva ori pe lună	De câteva ori pe lună	De câteva ori pe săptămână	De câteva ori pe săptămână	Niciodată / aproape niciodată	De câteva ori pe lună	11-20 țigări/zi
P153	De câteva ori pe săptămână	De câteva ori pe lună	Zilnic	De câteva ori pe lună	Zilnic	Zilnic	Zilnic	Zilnic	De câteva ori pe lună	De câteva ori pe lună	De câteva ori pe lună	Zilnic	Nu fumez deloc
P154	De câteva ori pe lună	De câteva ori pe lună	De câteva ori pe săptămână	De câteva ori pe lună	Rar (sub o dată pe lună)	De câteva ori pe săptămână	Zilnic	De câteva ori pe săptămână	De câteva ori pe săptămână	De câteva ori pe lună	De câteva ori pe lună	De câteva ori pe săptămână	11-20 țigări/zi
P155	Zilnic	De câteva ori pe lună	De câteva ori pe săptămână	De câteva ori pe lună	De câteva ori pe săptămână	De câteva ori pe lună	Zilnic	Zilnic	De câteva ori pe lună	De câteva ori pe lună	De câteva ori pe lună	Zilnic	Nu fumez deloc
P156	Niciodată / aproape niciodată	Niciodată / aproape niciodată	Niciodată / aproape niciodată	Niciodată / aproape niciodată	De câteva ori pe săptămână	De câteva ori pe săptămână	De câteva ori pe săptămână	De câteva ori pe săptămână	De câteva ori pe săptămână	De câteva ori pe săptămână	De câteva ori pe săptămână	De câteva ori pe săptămână	Mai puțin de o țigară/zi (ocazional)
P157	De câteva ori pe lună	De câteva ori pe lună	Zilnic	De câteva ori pe lună	De câteva ori pe săptămână	De câteva ori pe săptămână	De câteva ori pe săptămână	De câteva ori pe săptămână	De câteva ori pe săptămână	De câteva ori pe lună	De câteva ori pe lună	Zilnic	1-10 țigări/zi
P158	De câteva ori pe săptămână	De câteva ori pe săptămână	De câteva ori pe săptămână	De câteva ori pe săptămână	De câteva ori pe săptămână	De câteva ori pe săptămână	De câteva ori pe săptămână	De câteva ori pe săptămână	Zilnic	Zilnic	Zilnic	De câteva ori pe săptămână	Nu fumez deloc
P159	De câteva ori pe lună	De câteva ori pe lună	De câteva ori pe săptămână	De câteva ori pe lună	Zilnic	De câteva ori pe lună	De câteva ori pe lună	De câteva ori pe săptămână	De câteva ori pe lună	De câteva ori pe lună	De câteva ori pe lună	Zilnic	Nu fumez deloc
P160	De câteva ori pe lună	De câteva ori pe săptămână	De câteva ori pe săptămână	De câteva ori pe săptămână	Zilnic	De câteva ori pe săptămână	Zilnic	Zilnic	De câteva ori pe săptămână	De câteva ori pe lună	De câteva ori pe săptămână	Zilnic	Nu fumez deloc
P161	De câteva ori pe săptămână	De câteva ori pe lună	De câteva ori pe săptămână	De câteva ori pe lună	Zilnic	De câteva ori pe lună	Zilnic	De câteva ori pe săptămână	De câteva ori pe săptămână	De câteva ori pe lună	De câteva ori pe lună	Zilnic	1-10 țigări/zi
P162	Niciodată / aproape niciodată	De câteva ori pe lună	De câteva ori pe săptămână	De câteva ori pe săptămână	Zilnic	De câteva ori pe săptămână	De câteva ori pe săptămână	Zilnic	De câteva ori pe lună	Rar (sub o dată pe lună)	Niciodată / aproape niciodată	Zilnic	Nu fumez deloc
P163	De câteva ori pe săptămână	De câteva ori pe săptămână	Zilnic	De câteva ori pe lună	Zilnic	Zilnic	Zilnic	Zilnic	De câteva ori pe lună	De câteva ori pe lună	De câteva ori pe lună	Zilnic	1-10 țigări/zi
P164	De câteva ori pe săptămână	De câteva ori pe săptămână	De câteva ori pe săptămână	De câteva ori pe săptămână	De câteva ori pe săptămână	De câteva ori pe săptămână	De câteva ori pe săptămână	De câteva ori pe săptămână	De câteva ori pe săptămână	De câteva ori pe săptămână	Niciodată / aproape niciodată	Zilnic	Nu fumez deloc
P165	De câteva ori pe lună	De câteva ori pe săptămână	De câteva ori pe săptămână	De câteva ori pe săptămână	Zilnic	De câteva ori pe săptămână	Zilnic	De câteva ori pe săptămână	De câteva ori pe lună	Rar (sub o dată pe lună)	Rar (sub o dată pe lună)	De câteva ori pe săptămână	Nu fumez deloc
P166	De câteva ori pe săptămână	De câteva ori pe lună	De câteva ori pe săptămână	De câteva ori pe lună	Zilnic	De câteva ori pe săptămână	Zilnic	Zilnic	De câteva ori pe săptămână	De câteva ori pe săptămână	De câteva ori pe lună	Zilnic	Nu fumez deloc
P167	De câteva ori pe săptămână	De câteva ori pe săptămână	De câteva ori pe săptămână	De câteva ori pe săptămână	Zilnic	De câteva ori pe lună	Zilnic	Zilnic	De câteva ori pe săptămână	De câteva ori pe săptămână	De câteva ori pe lună	Zilnic	Mai puțin de o țigară/zi (ocazional)
P168	De câteva ori pe lună	De câteva ori pe săptămână	De câteva ori pe săptămână	De câteva ori pe săptămână	Zilnic	De câteva ori pe săptămână	Zilnic	Zilnic	De câteva ori pe lună	De câteva ori pe lună	De câteva ori pe săptămână	De câteva ori pe săptămână	Nu fumez deloc
P169	De câteva ori pe lună	De câteva ori pe săptămână	De câteva ori pe săptămână	De câteva ori pe săptămână	De câteva ori pe săptămână	De câteva ori pe săptămână	Zilnic	Zilnic	De câteva ori pe lună	De câteva ori pe lună	De câteva ori pe lună	Zilnic	Nu fumez deloc
P170	De câteva ori pe săptămână	De câteva ori pe săptămână	Zilnic	Rar (sub o dată pe lună)	De câteva ori pe săptămână	Zilnic	De câteva ori pe săptămână	De câteva ori pe săptămână	De câteva ori pe săptămână	Niciodată / aproape niciodată	De câteva ori pe săptămână	Zilnic	Peste 20 țigări/zi
P171	De câteva ori pe lună	De câteva ori pe lună	De câteva ori pe săptămână	De câteva ori pe lună	De câteva ori pe săptămână	De câteva ori pe lună	De câteva ori pe săptămână	De câteva ori pe săptămână	De câteva ori pe săptămână	De câteva ori pe lună	De câteva ori pe lună	Zilnic	11-20 țigări/zi
P172	De câteva ori pe săptămână	Rar (sub o dată pe lună)	De câteva ori pe săptămână	De câteva ori pe lună	De câteva ori pe săptămână	De câteva ori pe săptămână	Rar (sub o dată pe lună)	Zilnic	De câteva ori pe săptămână	Zilnic	Niciodată / aproape niciodată	De câteva ori pe săptămână	Nu fumez deloc
P173	De câteva ori pe săptămână	De câteva ori pe lună	De câteva ori pe lună	De câteva ori pe lună	De câteva ori pe lună	Rar (sub o dată pe lună)	De câteva ori pe lună	De câteva ori pe lună	Rar (sub o dată pe lună)	Rar (sub o dată pe lună)	Rar (sub o dată pe lună)	Zilnic	Mai puțin de o țigară/zi (ocazional)
P174	Rar (sub o dată pe lună)	De câteva ori pe lună	De câteva ori pe săptămână	De câteva ori pe lună	De câteva ori pe lună	Rar (sub o dată pe lună)	Zilnic	De câteva ori pe săptămână	De câteva ori pe lună	De câteva ori pe lună	De câteva ori pe săptămână	De câteva ori pe săptămână	Nu fumez deloc
P175	De câteva ori pe lună	De câteva ori pe săptămână	De câteva ori pe săptămână	De câteva ori pe săptămână	De câteva ori pe săptămână	De câteva ori pe săptămână	De câteva ori pe săptămână	De câteva ori pe săptămână	De câteva ori pe lună	De câteva ori pe lună	De câteva ori pe lună	Zilnic	11-20 țigări/zi
P176	De câteva ori pe săptămână	Niciodată / aproape niciodată	De câteva ori pe lună	Rar (sub o dată pe lună)	Rar (sub o dată pe lună)	De câteva ori pe lună	De câteva ori pe lună	De câteva ori pe lună	Zilnic	Zilnic	Niciodată / aproape niciodată	Zilnic	Nu fumez deloc
P177	De câteva ori pe săptămână	De câteva ori pe lună	De câteva ori pe săptămână	Rar (sub o dată pe lună)	De câteva ori pe lună	Rar (sub o dată pe lună)	Zilnic	Zilnic	Zilnic	De câteva ori pe săptămână	Niciodată / aproape niciodată	Zilnic	Nu fumez deloc
P178	De câteva ori pe săptămână	De câteva ori pe săptămână	De câteva ori pe săptămână	De câteva ori pe săptămână	De câteva ori pe săptămână	De câteva ori pe săptămână	De câteva ori pe săptămână	De câteva ori pe săptămână	De câteva ori pe săptămână	De câteva ori pe săptămână	De câteva ori pe săptămână	De câteva ori pe săptămână	Nu fumez deloc
P179	De câteva ori pe săptămână	De câteva ori pe săptămână	Rar (sub o dată pe lună)	De câteva ori pe lună	Zilnic	Zilnic	De câteva ori pe săptămână	Zilnic	De câteva ori pe săptămână	Niciodată / aproape niciodată	Zilnic	Zilnic	Nu fumez deloc
P180	De câteva ori pe lună	Niciodată / aproape niciodată	De câteva ori pe săptămână	De câteva ori pe lună	De câteva ori pe lună	De câteva ori pe săptămână	De câteva ori pe săptămână	Zilnic	De câteva ori pe săptămână	De câteva ori pe săptămână	De câteva ori pe lună	Zilnic	Nu fumez deloc
P181	Rar (sub o dată pe lună)	Niciodată / aproape niciodată	De câteva ori pe lună	Rar (sub o dată pe lună)	De câteva ori pe lună	De câteva ori pe lună	De câteva ori pe săptămână	De câteva ori pe săptămână	De câteva ori pe lună	Niciodată / aproape niciodată	Niciodată / aproape niciodată	Zilnic	Nu fumez deloc
P182	De câteva ori pe săptămână	Rar (sub o dată pe lună)	De câteva ori pe săptămână	De câteva ori pe săptămână	De câteva ori pe săptămână	Zilnic	De câteva ori pe săptămână	Zilnic	De câteva ori pe lună	De câteva ori pe săptămână	De câteva ori pe lună	Zilnic	1-10 țigări/zi
P183	De câteva ori pe lună	De câteva ori pe lună	De câteva ori pe lună	De câteva ori pe lună	Rar (sub o dată pe lună)	De câteva ori pe săptămână	De câteva ori pe lună	De câteva ori pe săptămână	De câteva ori pe lună	De câteva ori pe lună	Rar (sub o dată pe lună)	De câteva ori pe lună	Nu fumez deloc

Participant_ID	E1	E2	E3	E4	E5	E6	E7	E8	E9	E10	E11	E12	E13
P184	De câteva ori pe lună	De câteva ori pe săptămână	De câteva ori pe săptămână	De câteva ori pe săptămână	De câteva ori pe săptămână	De câteva ori pe lună	Zilnic	De câteva ori pe săptămână	De câteva ori pe săptămână	Niciodată / aproape niciodată	Niciodată / aproape niciodată	De câteva ori pe săptămână	Nu fumez deloc
P185	De câteva ori pe săptămână	De câteva ori pe săptămână	De câteva ori pe săptămână	De câteva ori pe lună	De câteva ori pe săptămână	De câteva ori pe săptămână	De câteva ori pe săptămână	De câteva ori pe săptămână	De câteva ori pe săptămână	Zilnic	De câteva ori pe lună	De câteva ori pe lună	Nu fumez deloc
P186	Niciodată / aproape niciodată	Niciodată / aproape niciodată	Niciodată / aproape niciodată	Niciodată / aproape niciodată	De câteva ori pe săptămână	De câteva ori pe lună	Zilnic	Zilnic	De câteva ori pe săptămână	De câteva ori pe săptămână	Niciodată / aproape niciodată	Zilnic	Nu fumez deloc
P187	De câteva ori pe săptămână	Rar (sub o dată pe lună)	Rar (sub o dată pe lună)	Niciodată / aproape niciodată	Rar (sub o dată pe lună)	De câteva ori pe lună	De câteva ori pe săptămână	Rar (sub o dată pe lună)	Zilnic	De câteva ori pe lună	De câteva ori pe lună	Zilnic	11-20 țigări/zi
P188	De câteva ori pe lună	De câteva ori pe săptămână	De câteva ori pe săptămână	De câteva ori pe lună	De câteva ori pe lună	De câteva ori pe săptămână	De câteva ori pe săptămână	De câteva ori pe săptămână	De câteva ori pe lună	Zilnic	De câteva ori pe lună	De câteva ori pe săptămână	1-10 țigări/zi
P189	De câteva ori pe săptămână	De câteva ori pe lună	De câteva ori pe săptămână	Niciodată / aproape niciodată	De câteva ori pe săptămână	De câteva ori pe lună	Zilnic	De câteva ori pe săptămână	Rar (sub o dată pe lună)	Rar (sub o dată pe lună)	De câteva ori pe săptămână	Zilnic	Nu fumez deloc
P190	De câteva ori pe săptămână	De câteva ori pe săptămână	De câteva ori pe săptămână	De câteva ori pe săptămână	Zilnic	De câteva ori pe săptămână	Zilnic	Zilnic	Zilnic	De câteva ori pe lună	De câteva ori pe lună	Zilnic	Nu fumez deloc
P191	De câteva ori pe lună	De câteva ori pe lună	De câteva ori pe săptămână	De câteva ori pe lună	De câteva ori pe lună	De câteva ori pe săptămână	Zilnic	De câteva ori pe săptămână	De câteva ori pe lună	De câteva ori pe săptămână	Niciodată / aproape niciodată	Zilnic	Nu fumez deloc
P192	De câteva ori pe lună	De câteva ori pe săptămână	De câteva ori pe săptămână	De câteva ori pe lună	Zilnic	De câteva ori pe lună	De câteva ori pe săptămână	De câteva ori pe săptămână	Zilnic	De câteva ori pe lună	Niciodată / aproape niciodată	Zilnic	Nu fumez deloc
P193	De câteva ori pe săptămână	De câteva ori pe lună	De câteva ori pe săptămână	De câteva ori pe lună	De câteva ori pe săptămână	De câteva ori pe lună	Zilnic	Zilnic	De câteva ori pe săptămână	De câteva ori pe lună	De câteva ori pe lună	Zilnic	1-10 țigări/zi
P194	De câteva ori pe săptămână	De câteva ori pe săptămână	De câteva ori pe săptămână	Rar (sub o dată pe lună)	De câteva ori pe săptămână	De câteva ori pe săptămână	De câteva ori pe săptămână	De câteva ori pe săptămână	Rar (sub o dată pe lună)	De câteva ori pe lună	Niciodată / aproape niciodată	Zilnic	1-10 țigări/zi
P195	De câteva ori pe săptămână	De câteva ori pe lună	De câteva ori pe săptămână	De câteva ori pe săptămână	De câteva ori pe lună	De câteva ori pe lună	De câteva ori pe săptămână	De câteva ori pe săptămână	De câteva ori pe săptămână	De câteva ori pe săptămână	De câteva ori pe lună	Zilnic	1-10 țigări/zi
P196	De câteva ori pe săptămână	De câteva ori pe lună	De câteva ori pe lună	Rar (sub o dată pe lună)	Zilnic	Zilnic	Zilnic	Zilnic	De câteva ori pe săptămână	De câteva ori pe săptămână	Zilnic	Zilnic	1-10 țigări/zi
P197	De câteva ori pe lună	De câteva ori pe lună	De câteva ori pe săptămână	De câteva ori pe lună	De câteva ori pe lună	De câteva ori pe lună	De câteva ori pe lună	De câteva ori pe lună	De câteva ori pe săptămână	Niciodată / aproape niciodată	Niciodată / aproape niciodată	Zilnic	Nu fumez deloc
P198	Rar (sub o dată pe lună)	Niciodată / aproape niciodată	De câteva ori pe lună	De câteva ori pe săptămână	De câteva ori pe lună	De câteva ori pe lună	Zilnic	De câteva ori pe săptămână	Rar (sub o dată pe lună)	De câteva ori pe lună	Rar (sub o dată pe lună)	Zilnic	Nu fumez deloc
P199	De câteva ori pe lună	De câteva ori pe săptămână	De câteva ori pe săptămână	De câteva ori pe lună	De câteva ori pe săptămână	De câteva ori pe săptămână	De câteva ori pe săptămână	De câteva ori pe săptămână	De câteva ori pe lună	De câteva ori pe lună	De câteva ori pe lună	De câteva ori pe săptămână	Nu fumez deloc
P200	De câteva ori pe săptămână	De câteva ori pe săptămână	De câteva ori pe săptămână	Niciodată / aproape niciodată	De câteva ori pe lună	De câteva ori pe lună	De câteva ori pe săptămână	De câteva ori pe săptămână	De câteva ori pe săptămână	De câteva ori pe săptămână	Niciodată / aproape niciodată	Zilnic	11-20 țigări/zi
P201	De câteva ori pe săptămână	De câteva ori pe lună	De câteva ori pe săptămână	De câteva ori pe lună	Zilnic	De câteva ori pe lună	Zilnic	De câteva ori pe săptămână	De câteva ori pe săptămână	Rar (sub o dată pe lună)	Rar (sub o dată pe lună)	Zilnic	Nu fumez deloc
P202	Zilnic	De câteva ori pe săptămână	De câteva ori pe săptămână	De câteva ori pe lună	Rar (sub o dată pe lună)	Rar (sub o dată pe lună)	Rar (sub o dată pe lună)	Zilnic	De câteva ori pe săptămână	De câteva ori pe lună	Rar (sub o dată pe lună)	De câteva ori pe lună	11-20 țigări/zi
P203	De câteva ori pe săptămână	Zilnic	Zilnic	Rar (sub o dată pe lună)	Zilnic	De câteva ori pe lună	Zilnic	De câteva ori pe săptămână	De câteva ori pe lună	De câteva ori pe săptămână	Niciodată / aproape niciodată	Zilnic	11-20 țigări/zi
P204	De câteva ori pe lună	De câteva ori pe lună	De câteva ori pe lună	De câteva ori pe lună	De câteva ori pe lună	De câteva ori pe lună	De câteva ori pe lună	De câteva ori pe lună	De câteva ori pe lună	De câteva ori pe lună	De câteva ori pe lună	De câteva ori pe lună	1-10 țigări/zi
P205	De câteva ori pe săptămână	De câteva ori pe lună	De câteva ori pe săptămână	Rar (sub o dată pe lună)	Rar (sub o dată pe lună)	De câteva ori pe săptămână	De câteva ori pe săptămână	Rar (sub o dată pe lună)	Zilnic	Zilnic	De câteva ori pe lună	Rar (sub o dată pe lună)	Peste 20 țigări/zi
P206	Rar (sub o dată pe lună)	De câteva ori pe săptămână	De câteva ori pe săptămână	De câteva ori pe săptămână	Zilnic	De câteva ori pe săptămână	Zilnic	Zilnic	De câteva ori pe săptămână	De câteva ori pe lună	De câteva ori pe lună	Zilnic	Nu fumez deloc
P207	De câteva ori pe lună	De câteva ori pe săptămână	De câteva ori pe săptămână	De câteva ori pe săptămână	Zilnic	De câteva ori pe săptămână	Zilnic	De câteva ori pe săptămână	Zilnic	Zilnic	Niciodată / aproape niciodată	De câteva ori pe săptămână	Nu fumez deloc
P208	Rar (sub o dată pe lună)	De câteva ori pe săptămână	De câteva ori pe săptămână	De câteva ori pe săptămână	De câteva ori pe săptămână	Zilnic	Zilnic	Zilnic	De câteva ori pe săptămână	De câteva ori pe lună	Niciodată / aproape niciodată	Zilnic	Nu fumez deloc
P209	De câteva ori pe săptămână	De câteva ori pe săptămână	De câteva ori pe lună	De câteva ori pe săptămână	Niciodată / aproape niciodată	Rar (sub o dată pe lună)	De câteva ori pe săptămână	De câteva ori pe săptămână	Zilnic	De câteva ori pe săptămână	De câteva ori pe săptămână	Zilnic	Nu fumez deloc
P210	De câteva ori pe lună	De câteva ori pe săptămână	De câteva ori pe săptămână	De câteva ori pe săptămână	Zilnic	De câteva ori pe săptămână	De câteva ori pe săptămână	De câteva ori pe săptămână	De câteva ori pe lună	De câteva ori pe lună	De câteva ori pe lună	Zilnic	Nu fumez deloc
P211	De câteva ori pe lună	De câteva ori pe săptămână	Zilnic	De câteva ori pe săptămână	Zilnic	De câteva ori pe săptămână	Zilnic	Zilnic	De câteva ori pe lună	De câteva ori pe lună	De câteva ori pe lună	De câteva ori pe săptămână	Nu fumez deloc
P212	Niciodată / aproape niciodată	Niciodată / aproape niciodată	Niciodată / aproape niciodată	De câteva ori pe lună	De câteva ori pe săptămână	De câteva ori pe lună	Zilnic	Zilnic	Rar (sub o dată pe lună)	Rar (sub o dată pe lună)	Niciodată / aproape niciodată	Zilnic	Nu fumez deloc
P213	De câteva ori pe lună	De câteva ori pe săptămână	De câteva ori pe săptămână	De câteva ori pe săptămână	De câteva ori pe săptămână	De câteva ori pe săptămână	De câteva ori pe săptămână	De câteva ori pe săptămână	De câteva ori pe săptămână	De câteva ori pe săptămână	De câteva ori pe lună	De câteva ori pe săptămână	Nu fumez deloc
P214	Niciodată / aproape niciodată	De câteva ori pe lună	De câteva ori pe lună	De câteva ori pe săptămână	Rar (sub o dată pe lună)	De câteva ori pe săptămână	Zilnic	De câteva ori pe săptămână	Rar (sub o dată pe lună)	Rar (sub o dată pe lună)	Rar (sub o dată pe lună)	Zilnic	Nu fumez deloc
P215	Niciodată / aproape niciodată	De câteva ori pe săptămână	Zilnic	De câteva ori pe săptămână	De câteva ori pe lună	De câteva ori pe săptămână	Zilnic	Zilnic	Niciodată / aproape niciodată	Niciodată / aproape niciodată	Niciodată / aproape niciodată	Niciodată / aproape niciodată	Nu fumez deloc
P216	De câteva ori pe lună	De câteva ori pe lună	De câteva ori pe săptămână	De câteva ori pe lună	De câteva ori pe săptămână	Zilnic	De câteva ori pe săptămână	Zilnic	Zilnic	De câteva ori pe săptămână	De câteva ori pe lună	Zilnic	Nu fumez deloc
P217	De câteva ori pe lună	De câteva ori pe lună	De câteva ori pe lună	De câteva ori pe săptămână	De câteva ori pe săptămână	De câteva ori pe săptămână	De câteva ori pe săptămână	Zilnic	De câteva ori pe săptămână	De câteva ori pe săptămână	De câteva ori pe lună	De câteva ori pe lună	Nu fumez deloc
P218	Rar (sub o dată pe lună)	De câteva ori pe săptămână	Zilnic	De câteva ori pe lună	Rar (sub o dată pe lună)	De câteva ori pe săptămână	De câteva ori pe săptămână	De câteva ori pe săptămână	Niciodată / aproape niciodată	Rar (sub o dată pe lună)	De câteva ori pe săptămână	Zilnic	1-10 țigări/zi
P219	De câteva ori pe lună	De câteva ori pe lună	De câteva ori pe săptămână	Rar (sub o dată pe lună)	Zilnic	De câteva ori pe săptămână	De câteva ori pe săptămână	De câteva ori pe săptămână	De câteva ori pe lună	De câteva ori pe lună	Rar (sub o dată pe lună)	De câteva ori pe săptămână	Nu fumez deloc
P220	De câteva ori pe săptămână	De câteva ori pe săptămână	De câteva ori pe săptămână	De câteva ori pe lună	Zilnic	De câteva ori pe săptămână	De câteva ori pe săptămână	Zilnic	De câteva ori pe săptămână	De câteva ori pe săptămână	De câteva ori pe lună	Zilnic	Nu fumez deloc
P221	De câteva ori pe lună	De câteva ori pe săptămână	De câteva ori pe săptămână	De câteva ori pe lună	De câteva ori pe lună	De câteva ori pe lună	Zilnic	De câteva ori pe lună	De câteva ori pe lună	De câteva ori pe săptămână	Rar (sub o dată pe lună)	De câteva ori pe săptămână	11-20 țigări/zi
P222	De câteva ori pe lună	De câteva ori pe lună	De câteva ori pe săptămână	De câteva ori pe lună	De câteva ori pe lună	De câteva ori pe lună	Zilnic	Zilnic	De câteva ori pe săptămână	Rar (sub o dată pe lună)	Rar (sub o dată pe lună)	Zilnic	1-10 țigări/zi
P223	De câteva ori pe lună	De câteva ori pe lună	De câteva ori pe săptămână	De câteva ori pe lună	De câteva ori pe lună	De câteva ori pe lună	Zilnic	De câteva ori pe săptămână	De câteva ori pe săptămână	De câteva ori pe lună	De câteva ori pe săptămână	Zilnic	Nu fumez deloc
P224	Niciodată / aproape niciodată	Niciodată / aproape niciodată	Niciodată / aproape niciodată	Niciodată / aproape niciodată	De câteva ori pe săptămână	Zilnic	Zilnic	Zilnic	De câteva ori pe lună	Rar (sub o dată pe lună)	Niciodată / aproape niciodată	Zilnic	Nu fumez deloc
P225	De câteva ori pe lună	De câteva ori pe lună	De câteva ori pe săptămână	De câteva ori pe lună	De câteva ori pe săptămână	De câteva ori pe lună	Zilnic	Zilnic	De câteva ori pe lună	De câteva ori pe lună	De câteva ori pe lună	Zilnic	1-10 țigări/zi
P226	Niciodată / aproape niciodată	Niciodată / aproape niciodată	De câteva ori pe lună	Niciodată / aproape niciodată	Niciodată / aproape niciodată	Niciodată / aproape niciodată	Niciodată / aproape niciodată	Niciodată / aproape niciodată	Niciodată / aproape niciodată	Niciodată / aproape niciodată	Niciodată / aproape niciodată	De câteva ori pe săptămână	Nu fumez deloc
P227	De câteva ori pe lună	De câteva ori pe lună	De câteva ori pe săptămână	De câteva ori pe săptămână	Zilnic	De câteva ori pe lună	Zilnic	De câteva ori pe săptămână	De câteva ori pe săptămână	De câteva ori pe lună	De câteva ori pe lună	Zilnic	Nu fumez deloc
P228	De câteva ori pe săptămână	De câteva ori pe săptămână	De câteva ori pe săptămână	De câteva ori pe lună	De câteva ori pe săptămână	De câteva ori pe săptămână	De câteva ori pe lună	De câteva ori pe săptămână	De câteva ori pe săptămână	De câteva ori pe săptămână	Niciodată / aproape niciodată	Zilnic	Nu fumez deloc
P229	De câteva ori pe săptămână	De câteva ori pe săptămână	De câteva ori pe săptămână	De câteva ori pe lună	De câteva ori pe săptămână	De câteva ori pe lună	De câteva ori pe săptămână	De câteva ori pe săptămână	De câteva ori pe săptămână	De câteva ori pe săptămână	Rar (sub o dată pe lună)	Zilnic	Nu fumez deloc
P230	De câteva ori pe lună	De câteva ori pe lună	De câteva ori pe săptămână	Rar (sub o dată pe lună)	Rar (sub o dată pe lună)	De câteva ori pe lună	De câteva ori pe săptămână	De câteva ori pe săptămână	De câteva ori pe săptămână	Rar (sub o dată pe lună)	Rar (sub o dată pe lună)	De câteva ori pe săptămână	Nu fumez deloc

Participant_ ID	E1	E2	E3	E4	E5	E6	E7	E8	E9	E10	E11	E12	E13
P231	De câteva ori pe lună	De câteva ori pe lună	De câteva ori pe săptămână	De câteva ori pe lună	Zilnic	De câteva ori pe săptămână	Zilnic	Zilnic	Rar (sub o dată pe lună)	Niciodată / aproape niciodată	Niciodată / aproape niciodată	Zilnic	Nu fumez deloc
P232	De câteva ori pe săptămână	Zilnic	De câteva ori pe săptămână	Zilnic	De câteva ori pe săptămână	Zilnic	De câteva ori pe săptămână	De câteva ori pe săptămână	Zilnic	Zilnic	Zilnic	Zilnic	Nu fumez deloc
P233	De câteva ori pe săptămână	De câteva ori pe săptămână	Zilnic	De câteva ori pe lună	De câteva ori pe săptămână	De câteva ori pe săptămână	Zilnic	Zilnic	De câteva ori pe lună	Rar (sub o dată pe lună)	Rar (sub o dată pe lună)	Zilnic	11-20 țigări/zi
P234	Rar (sub o dată pe lună)	Rar (sub o dată pe lună)	Rar (sub o dată pe lună)	Rar (sub o dată pe lună)	De câteva ori pe lună	De câteva ori pe lună	De câteva ori pe săptămână	De câteva ori pe săptămână	Niciodată / aproape niciodată	Rar (sub o dată pe lună)	De câteva ori pe lună	De câteva ori pe lună	Nu fumez deloc
P235	De câteva ori pe lună	De câteva ori pe săptămână	De câteva ori pe lună	Rar (sub o dată pe lună)	Rar (sub o dată pe lună)	Rar (sub o dată pe lună)	Rar (sub o dată pe lună)	Rar (sub o dată pe lună)	De câteva ori pe săptămână	Niciodată / aproape niciodată	Rar (sub o dată pe lună)	Zilnic	Peste 20 țigări/zi
P236	De câteva ori pe lună	De câteva ori pe lună	De câteva ori pe lună	De câteva ori pe lună	Rar (sub o dată pe lună)	De câteva ori pe lună	De câteva ori pe săptămână	De câteva ori pe săptămână	Rar (sub o dată pe lună)	Rar (sub o dată pe lună)	Rar (sub o dată pe lună)	Zilnic	1-10 țigări/zi
P237	De câteva ori pe lună	De câteva ori pe lună	De câteva ori pe lună	De câteva ori pe săptămână	De câteva ori pe săptămână	De câteva ori pe săptămână	De câteva ori pe săptămână	Zilnic	De câteva ori pe lună	De câteva ori pe lună	De câteva ori pe lună	Zilnic	Nu fumez deloc
P238	De câteva ori pe lună	De câteva ori pe lună	De câteva ori pe lună	De câteva ori pe lună	De câteva ori pe lună	De câteva ori pe lună	Rar (sub o dată pe lună)	De câteva ori pe săptămână	Rar (sub o dată pe lună)	Niciodată / aproape niciodată	De câteva ori pe săptămână	De câteva ori pe lună	1-10 țigări/zi
P239	De câteva ori pe lună	De câteva ori pe lună	De câteva ori pe lună	De câteva ori pe lună	Zilnic	De câteva ori pe lună	Zilnic	Zilnic	Zilnic	De câteva ori pe lună	De câteva ori pe lună	De câteva ori pe săptămână	Nu fumez deloc
P240	De câteva ori pe lună	De câteva ori pe săptămână	De câteva ori pe lună	De câteva ori pe săptămână	Zilnic	De câteva ori pe săptămână	Zilnic	Zilnic	De câteva ori pe săptămână	De câteva ori pe lună	Rar (sub o dată pe lună)	Zilnic	Nu fumez deloc
P241	Rar (sub o dată pe lună)	Rar (sub o dată pe lună)	De câteva ori pe lună	De câteva ori pe lună	De câteva ori pe săptămână	De câteva ori pe lună	Zilnic	De câteva ori pe săptămână	De câteva ori pe lună	Rar (sub o dată pe lună)	De câteva ori pe lună	De câteva ori pe lună	11-20 țigări/zi
P242	De câteva ori pe săptămână	De câteva ori pe săptămână	De câteva ori pe săptămână	De câteva ori pe lună	De câteva ori pe lună	De câteva ori pe săptămână	De câteva ori pe lună	De câteva ori pe săptămână	De câteva ori pe săptămână	De câteva ori pe săptămână	De câteva ori pe săptămână	Zilnic	Mai puțin de o țigară/zi (ocazional)
P243	Rar (sub o dată pe lună)	Rar (sub o dată pe lună)	De câteva ori pe lună	Rar (sub o dată pe lună)	Rar (sub o dată pe lună)	Rar (sub o dată pe lună)	Zilnic	Zilnic	Rar (sub o dată pe lună)	Niciodată / aproape niciodată	De câteva ori pe lună	Zilnic	Nu fumez deloc
P244	De câteva ori pe săptămână	De câteva ori pe lună	De câteva ori pe lună	De câteva ori pe lună	De câteva ori pe săptămână	De câteva ori pe lună	Zilnic	De câteva ori pe săptămână	De câteva ori pe lună	Niciodată / aproape niciodată	Niciodată / aproape niciodată	Zilnic	Nu fumez deloc
P245	Zilnic	De câteva ori pe lună	De câteva ori pe lună	De câteva ori pe lună	De câteva ori pe lună	De câteva ori pe lună	De câteva ori pe săptămână	De câteva ori pe lună	De câteva ori pe lună	De câteva ori pe lună	De câteva ori pe lună	De câteva ori pe săptămână	11-20 țigări/zi
P246	De câteva ori pe săptămână	De câteva ori pe lună	De câteva ori pe săptămână	De câteva ori pe lună	Zilnic	De câteva ori pe lună	De câteva ori pe săptămână	De câteva ori pe săptămână	De câteva ori pe săptămână	De câteva ori pe săptămână	De câteva ori pe lună	Zilnic	Nu fumez deloc
P247	De câteva ori pe lună	De câteva ori pe lună	De câteva ori pe săptămână	De câteva ori pe lună	De câteva ori pe lună	De câteva ori pe lună	Zilnic	De câteva ori pe săptămână	De câteva ori pe săptămână	Rar (sub o dată pe lună)	Rar (sub o dată pe lună)	Zilnic	Nu fumez deloc
P248	Rar (sub o dată pe lună)	De câteva ori pe lună	De câteva ori pe săptămână	Rar (sub o dată pe lună)	Zilnic	Zilnic	Zilnic	Zilnic	Zilnic	De câteva ori pe lună	Niciodată / aproape niciodată	Zilnic	11-20 țigări/zi
P249	De câteva ori pe lună	De câteva ori pe săptămână	De câteva ori pe săptămână	De câteva ori pe lună	Zilnic	Zilnic	Zilnic	Zilnic	Rar (sub o dată pe lună)	Rar (sub o dată pe lună)	Niciodată / aproape niciodată	Zilnic	1-10 țigări/zi
P250	De câteva ori pe săptămână	De câteva ori pe săptămână	De câteva ori pe săptămână	De câteva ori pe lună	De câteva ori pe săptămână	De câteva ori pe lună	De câteva ori pe săptămână	De câteva ori pe săptămână	De câteva ori pe lună	De câteva ori pe lună	De câteva ori pe lună	Zilnic	Peste 20 țigări/zi
P251	De câteva ori pe lună	De câteva ori pe săptămână	De câteva ori pe săptămână	De câteva ori pe lună	De câteva ori pe lună	De câteva ori pe săptămână	De câteva ori pe săptămână	De câteva ori pe săptămână	De câteva ori pe lună	Zilnic	De câteva ori pe lună	De câteva ori pe săptămână	1-10 țigări/zi
P252	De câteva ori pe săptămână	De câteva ori pe săptămână	De câteva ori pe săptămână	De câteva ori pe lună	Zilnic	Zilnic	Zilnic	Zilnic	Zilnic	De câteva ori pe săptămână	De câteva ori pe lună	Zilnic	Nu fumez deloc
P253	De câteva ori pe lună	De câteva ori pe lună	De câteva ori pe lună	De câteva ori pe lună	De câteva ori pe săptămână	De câteva ori pe lună	Zilnic	Zilnic	De câteva ori pe lună	De câteva ori pe lună	De câteva ori pe lună	Zilnic	11-20 țigări/zi
P254	De câteva ori pe săptămână	De câteva ori pe săptămână	De câteva ori pe săptămână	De câteva ori pe lună	Zilnic	De câteva ori pe săptămână	De câteva ori pe săptămână	De câteva ori pe săptămână	Zilnic	Zilnic	De câteva ori pe lună	Zilnic	Mai puțin de o țigară/zi (ocazional)
P255	De câteva ori pe lună	Zilnic	Zilnic	De câteva ori pe lună	Zilnic	Zilnic	Zilnic	Zilnic	De câteva ori pe săptămână	De câteva ori pe lună	De câteva ori pe lună	Zilnic	Peste 20 țigări/zi
P256	De câteva ori pe lună	De câteva ori pe săptămână	De câteva ori pe lună	De câteva ori pe lună	De câteva ori pe lună	De câteva ori pe lună	Zilnic	De câteva ori pe săptămână	De câteva ori pe lună	De câteva ori pe săptămână	De câteva ori pe lună	Zilnic	11-20 țigări/zi
P257	De câteva ori pe lună	De câteva ori pe lună	De câteva ori pe lună	De câteva ori pe lună	Zilnic	De câteva ori pe lună	Zilnic	De câteva ori pe săptămână	Rar (sub o dată pe lună)	Rar (sub o dată pe lună)	Rar (sub o dată pe lună)	Zilnic	Mai puțin de o țigară/zi (ocazional)
P258	Zilnic	De câteva ori pe săptămână	De câteva ori pe săptămână	De câteva ori pe săptămână	De câteva ori pe săptămână	De câteva ori pe săptămână	De câteva ori pe săptămână	De câteva ori pe săptămână	Zilnic	Zilnic	De câteva ori pe săptămână	Zilnic	Nu fumez deloc
P259	Rar (sub o dată pe lună)	Rar (sub o dată pe lună)	De câteva ori pe săptămână	Rar (sub o dată pe lună)	Zilnic	Zilnic	De câteva ori pe săptămână	De câteva ori pe săptămână	De câteva ori pe săptămână	Niciodată / aproape niciodată	Niciodată / aproape niciodată	De câteva ori pe săptămână	11-20 țigări/zi
P260	De câteva ori pe lună	De câteva ori pe săptămână	De câteva ori pe săptămână	De câteva ori pe lună	Zilnic	De câteva ori pe săptămână	De câteva ori pe săptămână	De câteva ori pe săptămână	Zilnic	De câteva ori pe săptămână	De câteva ori pe lună	Zilnic	Nu fumez deloc
P261	Niciodată / aproape niciodată	Niciodată / aproape niciodată	Niciodată / aproape niciodată	De câteva ori pe lună	De câteva ori pe lună	De câteva ori pe săptămână	Zilnic	De câteva ori pe săptămână	Rar (sub o dată pe lună)	Niciodată / aproape niciodată	De câteva ori pe lună	Zilnic	Nu fumez deloc
P262	De câteva ori pe lună	De câteva ori pe lună	De câteva ori pe săptămână	De câteva ori pe lună	De câteva ori pe lună	Zilnic	Zilnic	Zilnic	De câteva ori pe lună	De câteva ori pe lună	De câteva ori pe săptămână	Zilnic	Nu fumez deloc
P263	De câteva ori pe lună	De câteva ori pe lună	De câteva ori pe săptămână	De câteva ori pe lună	De câteva ori pe săptămână	De câteva ori pe lună	De câteva ori pe săptămână	De câteva ori pe lună	De câteva ori pe lună	De câteva ori pe lună	De câteva ori pe lună	Zilnic	Nu fumez deloc
P264	De câteva ori pe săptămână	De câteva ori pe săptămână	De câteva ori pe lună	De câteva ori pe lună	De câteva ori pe săptămână	De câteva ori pe lună	De câteva ori pe săptămână	De câteva ori pe săptămână	De câteva ori pe săptămână	Zilnic	De câteva ori pe lună	De câteva ori pe săptămână	11-20 țigări/zi
P265	De câteva ori pe lună	De câteva ori pe săptămână	De câteva ori pe săptămână	Rar (sub o dată pe lună)	Rar (sub o dată pe lună)	Rar (sub o dată pe lună)	Rar (sub o dată pe lună)	De câteva ori pe lună	Rar (sub o dată pe lună)	De câteva ori pe săptămână	Niciodată / aproape niciodată	Zilnic	Mai puțin de o țigară/zi (ocazional)
P266	De câteva ori pe lună	De câteva ori pe lună	De câteva ori pe lună	Rar (sub o dată pe lună)	De câteva ori pe săptămână	De câteva ori pe săptămână	De câteva ori pe lună	De câteva ori pe lună	De câteva ori pe lună	Niciodată / aproape niciodată	Niciodată / aproape niciodată	De câteva ori pe săptămână	Nu fumez deloc
P267	De câteva ori pe săptămână	De câteva ori pe lună	De câteva ori pe săptămână	De câteva ori pe lună	De câteva ori pe lună	De câteva ori pe lună	De câteva ori pe lună	De câteva ori pe lună	Zilnic	Zilnic	Zilnic	De câteva ori pe săptămână	1-10 țigări/zi
P268	De câteva ori pe săptămână	De câteva ori pe săptămână	De câteva ori pe săptămână	De câteva ori pe săptămână	De câteva ori pe săptămână	De câteva ori pe săptămână	De câteva ori pe săptămână	De câteva ori pe săptămână	De câteva ori pe săptămână	De câteva ori pe săptămână	De câteva ori pe săptămână	Zilnic	Nu fumez deloc
P269	De câteva ori pe lună	De câteva ori pe lună	De câteva ori pe lună	De câteva ori pe lună	De câteva ori pe săptămână	De câteva ori pe lună	Zilnic	Zilnic	Niciodată / aproape niciodată	Rar (sub o dată pe lună)	Niciodată / aproape niciodată	Zilnic	Nu fumez deloc
P270	De câteva ori pe săptămână	De câteva ori pe lună	De câteva ori pe săptămână	De câteva ori pe lună	De câteva ori pe lună	De câteva ori pe lună	Zilnic	De câteva ori pe săptămână	De câteva ori pe lună	De câteva ori pe lună	De câteva ori pe lună	Zilnic	Nu fumez deloc
P271	De câteva ori pe lună	De câteva ori pe lună	De câteva ori pe săptămână	Rar (sub o dată pe lună)	Niciodată / aproape niciodată	De câteva ori pe lună	De câteva ori pe lună	De câteva ori pe lună	Zilnic	Rar (sub o dată pe lună)	Rar (sub o dată pe lună)	Zilnic	Nu fumez deloc
P272	De câteva ori pe săptămână	De câteva ori pe săptămână	De câteva ori pe săptămână	De câteva ori pe săptămână	De câteva ori pe săptămână	De câteva ori pe săptămână	Zilnic	De câteva ori pe săptămână	De câteva ori pe săptămână	De câteva ori pe săptămână	Zilnic	Zilnic	Nu fumez deloc
P273	De câteva ori pe săptămână	De câteva ori pe săptămână	De câteva ori pe săptămână	De câteva ori pe lună	De câteva ori pe lună	De câteva ori pe săptămână	De câteva ori pe săptămână	De câteva ori pe săptămână	De câteva ori pe lună	Rar (sub o dată pe lună)	De câteva ori pe lună	Zilnic	Nu fumez deloc
P274	De câteva ori pe lună	De câteva ori pe lună	De câteva ori pe lună	Rar (sub o dată pe lună)	Rar (sub o dată pe lună)	De câteva ori pe lună	De câteva ori pe lună	De câteva ori pe lună	De câteva ori pe săptămână	De câteva ori pe săptămână	De câteva ori pe săptămână	Zilnic	11-20 țigări/zi
P275	De câteva ori pe lună	De câteva ori pe lună	De câteva ori pe săptămână	Rar (sub o dată pe lună)	Rar (sub o dată pe lună)	De câteva ori pe lună	De câteva ori pe săptămână	De câteva ori pe săptămână	De câteva ori pe lună	De câteva ori pe lună	De câteva ori pe lună	Zilnic	Nu fumez deloc
P276	Zilnic	De câteva ori pe săptămână	De câteva ori pe săptămână	De câteva ori pe lună	De câteva ori pe săptămână	Rar (sub o dată pe lună)	Zilnic	De câteva ori pe săptămână	Zilnic	Rar (sub o dată pe lună)	De câteva ori pe săptămână	Rar (sub o dată pe lună)	Nu fumez deloc

Participant_ ID	E1	E2	E3	E4	E5	E6	E7	E8	E9	E10	E11	E12	E13
P277	De câteva ori pe lună	De câteva ori pe lună	De câteva ori pe săptămână	De câteva ori pe săptămână	De câteva ori pe săptămână	De câteva ori pe săptămână	Zilnic	Zilnic	De câteva ori pe lună	De câteva ori pe lună	De câteva ori pe lună	Zilnic	Nu fumez deloc
P278	De câteva ori pe săptămână	De câteva ori pe săptămână	De câteva ori pe săptămână	De câteva ori pe săptămână	De câteva ori pe săptămână	De câteva ori pe lună	De câteva ori pe săptămână	De câteva ori pe săptămână	De câteva ori pe săptămână	De câteva ori pe lună	De câteva ori pe lună	Zilnic	11-20 țigări/zi
P279	Niciodată / aproape niciodată	Rar (sub o dată pe lună)	De câteva ori pe săptămână	Zilnic	Zilnic	Zilnic	Zilnic	Zilnic	Niciodată / aproape niciodată	Niciodată / aproape niciodată	Niciodată / aproape niciodată	Zilnic	Nu fumez deloc
P280	De câteva ori pe săptămână	De câteva ori pe săptămână	De câteva ori pe săptămână	De câteva ori pe lună	De câteva ori pe săptămână	Zilnic	Zilnic	De câteva ori pe săptămână	De câteva ori pe lună	Rar (sub o dată pe lună)	De câteva ori pe lună	Zilnic	Nu fumez deloc
P281	Rar (sub o dată pe lună)	De câteva ori pe lună	Rar (sub o dată pe lună)	De câteva ori pe lună	Niciodată / aproape niciodată	Niciodată / aproape niciodată	Niciodată / aproape niciodată	Niciodată / aproape niciodată	Niciodată / aproape niciodată	Niciodată / aproape niciodată	Niciodată / aproape niciodată	Niciodată / aproape niciodată	Nu fumez deloc
P282	De câteva ori pe lună	De câteva ori pe lună	De câteva ori pe săptămână	De câteva ori pe lună	De câteva ori pe săptămână	De câteva ori pe săptămână	De câteva ori pe săptămână	De câteva ori pe săptămână	Zilnic	De câteva ori pe lună	De câteva ori pe lună	De câteva ori pe săptămână	11-20 țigări/zi
P283	De câteva ori pe lună	De câteva ori pe lună	De câteva ori pe lună	De câteva ori pe lună	De câteva ori pe săptămână	De câteva ori pe săptămână	Zilnic	Zilnic	De câteva ori pe săptămână	Niciodată / aproape niciodată	De câteva ori pe lună	Zilnic	Nu fumez deloc
P284	De câteva ori pe săptămână	De câteva ori pe săptămână	De câteva ori pe săptămână	De câteva ori pe lună	De câteva ori pe săptămână	De câteva ori pe săptămână	Zilnic	Zilnic	De câteva ori pe săptămână	De câteva ori pe săptămână	De câteva ori pe săptămână	Zilnic	Nu fumez deloc
P285	De câteva ori pe săptămână	De câteva ori pe săptămână	De câteva ori pe săptămână	De câteva ori pe lună	De câteva ori pe săptămână	De câteva ori pe lună	Zilnic	Zilnic	De câteva ori pe săptămână	De câteva ori pe săptămână	De câteva ori pe săptămână	Zilnic	Nu fumez deloc
P286	De câteva ori pe lună	De câteva ori pe lună	De câteva ori pe săptămână	De câteva ori pe lună	De câteva ori pe săptămână	De câteva ori pe săptămână	Zilnic	Zilnic	De câteva ori pe lună	De câteva ori pe lună	De câteva ori pe lună	Zilnic	Nu fumez deloc
P287	De câteva ori pe săptămână	De câteva ori pe săptămână	De câteva ori pe săptămână	De câteva ori pe lună	De câteva ori pe lună	De câteva ori pe lună	De câteva ori pe săptămână	De câteva ori pe săptămână	De câteva ori pe lună	De câteva ori pe lună	Niciodată / aproape niciodată	Zilnic	11-20 țigări/zi
P288	Zilnic	De câteva ori pe săptămână	De câteva ori pe săptămână	De câteva ori pe lună	De câteva ori pe lună	Rar (sub o dată pe lună)	De câteva ori pe săptămână	De câteva ori pe lună	De câteva ori pe săptămână	Niciodată / aproape niciodată	De câteva ori pe lună	De câteva ori pe lună	Nu fumez deloc
P289	De câteva ori pe săptămână	De câteva ori pe săptămână	Zilnic	De câteva ori pe lună	Zilnic	De câteva ori pe lună	Zilnic	De câteva ori pe săptămână	De câteva ori pe săptămână	De câteva ori pe săptămână	Zilnic	Zilnic	Nu fumez deloc
P290	Rar (sub o dată pe lună)	Rar (sub o dată pe lună)	De câteva ori pe săptămână	De câteva ori pe săptămână	Zilnic	Zilnic	Zilnic	Zilnic	De câteva ori pe lună	De câteva ori pe lună	De câteva ori pe lună	Zilnic	Nu fumez deloc
P291	De câteva ori pe săptămână	De câteva ori pe săptămână	De câteva ori pe săptămână	De câteva ori pe săptămână	De câteva ori pe săptămână	De câteva ori pe săptămână	De câteva ori pe săptămână	De câteva ori pe săptămână	De câteva ori pe săptămână	De câteva ori pe săptămână	De câteva ori pe săptămână	De câteva ori pe săptămână	Nu fumez deloc
P292	De câteva ori pe lună	De câteva ori pe lună	De câteva ori pe săptămână	De câteva ori pe săptămână	De câteva ori pe lună	De câteva ori pe lună	Zilnic	Zilnic	De câteva ori pe săptămână	De câteva ori pe lună	De câteva ori pe lună	Zilnic	Nu fumez deloc
P293	De câteva ori pe săptămână	De câteva ori pe săptămână	De câteva ori pe săptămână	De câteva ori pe lună	De câteva ori pe lună	De câteva ori pe lună	Zilnic	Zilnic	De câteva ori pe săptămână	De câteva ori pe lună	De câteva ori pe lună	Zilnic	Nu fumez deloc
P294	De câteva ori pe săptămână	De câteva ori pe săptămână	De câteva ori pe săptămână	Rar (sub o dată pe lună)	De câteva ori pe lună	De câteva ori pe lună	De câteva ori pe săptămână	De câteva ori pe săptămână	De câteva ori pe lună	De câteva ori pe săptămână	De câteva ori pe lună	De câteva ori pe săptămână	Nu fumez deloc
P295	De câteva ori pe săptămână	De câteva ori pe lună	De câteva ori pe săptămână	De câteva ori pe lună	Zilnic	De câteva ori pe săptămână	Zilnic	De câteva ori pe săptămână	De câteva ori pe lună	De câteva ori pe săptămână	Rar (sub o dată pe lună)	Zilnic	Nu fumez deloc
P296	Rar (sub o dată pe lună)	De câteva ori pe lună	De câteva ori pe săptămână	De câteva ori pe lună	De câteva ori pe lună	De câteva ori pe săptămână	Zilnic	Zilnic	De câteva ori pe lună	Niciodată / aproape niciodată	Niciodată / aproape niciodată	Zilnic	Nu fumez deloc
P297	De câteva ori pe săptămână	Rar (sub o dată pe lună)	De câteva ori pe lună	De câteva ori pe lună	De câteva ori pe săptămână	De câteva ori pe săptămână	Zilnic	Zilnic	Zilnic	Niciodată / aproape niciodată	Niciodată / aproape niciodată	Zilnic	Nu fumez deloc
P298	De câteva ori pe lună	De câteva ori pe lună	De câteva ori pe săptămână	Rar (sub o dată pe lună)	De câteva ori pe lună	De câteva ori pe lună	De câteva ori pe lună	De câteva ori pe lună	De câteva ori pe lună	Rar (sub o dată pe lună)	Niciodată / aproape niciodată	Zilnic	Nu fumez deloc
P299	Rar (sub o dată pe lună)	Rar (sub o dată pe lună)	De câteva ori pe lună	Rar (sub o dată pe lună)	Rar (sub o dată pe lună)	De câteva ori pe lună	De câteva ori pe săptămână	De câteva ori pe lună	Rar (sub o dată pe lună)	Rar (sub o dată pe lună)	Niciodată / aproape niciodată	De câteva ori pe lună	Nu fumez deloc
P300	De câteva ori pe săptămână	De câteva ori pe săptămână	De câteva ori pe săptămână	De câteva ori pe lună	Niciodată / aproape niciodată	Rar (sub o dată pe lună)	De câteva ori pe lună	Rar (sub o dată pe lună)	De câteva ori pe săptămână	De câteva ori pe lună	De câteva ori pe lună	De câteva ori pe săptămână	1-10 țigări/zi
P301	De câteva ori pe lună	De câteva ori pe lună	Rar (sub o dată pe lună)	Rar (sub o dată pe lună)	De câteva ori pe lună	Rar (sub o dată pe lună)	Zilnic	De câteva ori pe săptămână	De câteva ori pe lună	Rar (sub o dată pe lună)	De câteva ori pe lună	Zilnic	Nu fumez deloc

Panel F — Information sources and trust in sources

Participant_ID	F1	F2a	F2b	F2c	F2d	F2e	F2f	F2g
P001	Dietetician / nutriționist, Articole științifice / surse academice, Aplicații mobile dedicate sănătății, Rețele sociale (Facebook, Instagram, TikTok, YouTube), Site-uri de internet generale, Familie și prieteni	Încredere foarte ridicată	Încredere foarte ridicată	Neutru	Neutru	Încredere scăzută	Neutru	Încredere foarte ridicată
P002	Articole științifice / surse academice, Rețele sociale (Facebook, Instagram, TikTok, YouTube)	Încredere ridicată	Încredere foarte ridicată	Neutru	Neutru	Încredere scăzută	Încredere scăzută	Încredere foarte ridicată
P003	Rețele sociale (Facebook, Instagram, TikTok, YouTube)	Neutru	Încredere ridicată	Încredere ridicată	Încredere scăzută	Încredere foarte scăzută	Încredere ridicată	Încredere foarte ridicată
P004	Medicul specialist (oncolog, gastroenterolog, alt specialist), Dietetician / nutriționist, Articole științifice / surse academice, Aplicații mobile dedicate sănătății	Încredere scăzută	Încredere foarte ridicată	Încredere scăzută	Încredere scăzută	Încredere foarte scăzută	Încredere scăzută	Încredere ridicată
P005	Medicul de familie, Medicul specialist (oncolog, gastroenterolog, alt specialist), Farmacist, Site-uri de internet generale	Încredere foarte scăzută	Încredere foarte scăzută	Încredere foarte scăzută	Încredere foarte scăzută	Neutru	Neutru	Încredere foarte scăzută
P006	Aplicații mobile dedicate sănătății, Rețele sociale (Facebook, Instagram, TikTok, YouTube), Familie și prieteni	Încredere ridicată	Încredere foarte ridicată	Neutru	Neutru	Încredere scăzută	Încredere ridicată	Încredere foarte ridicată
P007	Rețele sociale (Facebook, Instagram, TikTok, YouTube), Familie și prieteni	Încredere foarte ridicată	Încredere foarte ridicată	Neutru	Neutru	Neutru	Neutru	Încredere foarte ridicată
P008	Dietetician / nutriționist, Rețele sociale (Facebook, Instagram, TikTok, YouTube), Site-uri de internet generale, Familie și prieteni	Încredere scăzută	Încredere foarte scăzută	Încredere scăzută	Neutru	Încredere foarte scăzută	Neutru	Neutru
P009	Articole științifice / surse academice, Rețele sociale (Facebook, Instagram, TikTok, YouTube), Site-uri de internet generale, Familie și prieteni	Încredere foarte ridicată	Încredere foarte ridicată	Încredere ridicată	Încredere ridicată	Încredere ridicată	Încredere ridicată	Încredere foarte ridicată
P010	Medicul de familie, Medicul specialist (oncolog, gastroenterolog, alt specialist), Farmacist	Neutru	Neutru	Neutru	Neutru	Neutru	Neutru	Neutru
P011	Rețele sociale (Facebook, Instagram, TikTok, YouTube)	Neutru	Încredere ridicată	Neutru	Neutru	Neutru	Neutru	Neutru
P012	Articole științifice / surse academice, Rețele sociale (Facebook, Instagram, TikTok, YouTube), Site-uri de internet generale	Neutru	Încredere ridicată	Încredere scăzută	Încredere foarte scăzută	Încredere scăzută	Încredere scăzută	Încredere ridicată
P013	Televiziune / radio	Încredere scăzută	Neutru	Încredere scăzută	Încredere foarte scăzută	Încredere scăzută	Neutru	Încredere foarte scăzută
P014	Articole științifice / surse academice, Televiziune / radio, Rețele sociale (Facebook, Instagram, TikTok, YouTube)	Încredere ridicată	Încredere ridicată	Încredere ridicată	Încredere ridicată	Încredere ridicată	Încredere ridicată	Încredere ridicată
P015	Site-uri de internet generale	Neutru	Neutru	Neutru	Neutru	Neutru	Neutru	Neutru
P016	Medicul specialist (oncolog, gastroenterolog, alt specialist), Articole științifice / surse academice, Rețele sociale (Facebook, Instagram, TikTok, YouTube), Reviste și ziare	Neutru	Neutru	Neutru	Încredere ridicată	Neutru	Neutru	Încredere foarte ridicată
P017	Site-uri de internet generale, Familie și prieteni	Încredere foarte ridicată	Încredere foarte ridicată	Neutru	Neutru	Neutru	Neutru	Încredere foarte ridicată
P018	Medicul specialist (oncolog, gastroenterolog, alt specialist), Dietetician / nutriționist, Articole științifice / surse academice, Familie și prieteni	Neutru	Încredere ridicată	Neutru	Neutru	Neutru	Încredere ridicată	Încredere ridicată
P019	Dietetician / nutriționist, Articole științifice / surse academice, Aplicații mobile dedicate sănătății, Televiziune / radio	Neutru	Încredere ridicată	Încredere scăzută	Încredere scăzută	Încredere foarte scăzută	Încredere foarte scăzută	Neutru
P020	Dietetician / nutriționist, Aplicații mobile dedicate sănătății, Rețele sociale (Facebook, Instagram, TikTok, YouTube)	Neutru	Încredere ridicată	Neutru	Neutru	Neutru	Încredere scăzută	Încredere foarte ridicată
P021	Aplicații mobile dedicate sănătății, Rețele sociale (Facebook, Instagram, TikTok, YouTube), Reviste și ziare, Site-uri de internet generale	Încredere foarte ridicată	Încredere foarte ridicată	Încredere ridicată	Neutru	Încredere ridicată	Încredere foarte ridicată	Încredere foarte ridicată
P022	Medicul de familie, Farmacist, Dietetician / nutriționist, Articole științifice / surse academice, Aplicații mobile dedicate sănătății	Încredere ridicată	Încredere foarte ridicată	Neutru	Neutru	Încredere scăzută	Neutru	Încredere foarte ridicată
P023	Medicul de familie, Articole științifice / surse academice, Aplicații mobile dedicate sănătății, Televiziune / radio, Rețele sociale (Facebook, Instagram, TikTok, YouTube), Site-uri de internet generale, Familie și prieteni	Încredere ridicată	Încredere foarte ridicată	Neutru	Neutru	Neutru	Încredere ridicată	Încredere foarte ridicată
P024	Dietetician / nutriționist, Aplicații mobile dedicate sănătății, Rețele sociale (Facebook, Instagram, TikTok, YouTube), Site-uri de internet generale	Încredere ridicată	Încredere foarte ridicată	Neutru	Neutru	Neutru	Neutru	Încredere foarte ridicată
P025	Dietetician / nutriționist, Aplicații mobile dedicate sănătății, Rețele sociale (Facebook, Instagram, TikTok, YouTube), Reviste și ziare, Site-uri de internet generale	Neutru	Încredere ridicată	Încredere scăzută	Neutru	Neutru	Neutru	Încredere foarte ridicată
P026	Articole științifice / surse academice	Neutru	Încredere foarte ridicată	Încredere scăzută	Încredere scăzută	Încredere scăzută	Încredere scăzută	Încredere foarte ridicată
P027	Medicul de familie, Medicul specialist (oncolog, gastroenterolog, alt specialist), Farmacist, Dietetician / nutriționist	Încredere foarte ridicată	Încredere foarte ridicată	Neutru	Neutru	Neutru	Neutru	Neutru
P028	Dietetician / nutriționist, Articole științifice / surse academice, Aplicații mobile dedicate sănătății, Rețele sociale (Facebook, Instagram, TikTok, YouTube), Site-uri de internet generale	Neutru	Încredere foarte ridicată	Încredere ridicată	Încredere ridicată	Neutru	Neutru	Încredere foarte ridicată

Participant_ID	F1	F2a	F2b	F2c	F2d	F2e	F2f	F2g
P029	Aplicații mobile dedicate sănătății, Televiziune / radio, Rețele sociale (Facebook, Instagram, TikTok, YouTube)	Încredere foarte ridicată	Încredere foarte ridicată	Neutru	Neutru	Neutru	Încredere foarte ridicată	Neutru
P030	Farmacist, Aplicații mobile dedicate sănătății, Rețele sociale (Facebook, Instagram, TikTok, YouTube), Site-uri de internet generale	Încredere foarte ridicată	Încredere foarte ridicată	Neutru	Neutru	Încredere scăzută	Neutru	Încredere foarte ridicată
P031	Articole științifice / surse academice, Rețele sociale (Facebook, Instagram, TikTok, YouTube), Site-uri de internet generale, Familie și prieteni	Neutru	Încredere ridicată	Neutru	Neutru	Neutru	Încredere ridicată	Încredere ridicată
P032	Medicul specialist (oncolog, gastroenterolog, alt specialist)	Neutru	Neutru	Neutru	Neutru	Neutru	Neutru	Neutru
P033	Dietetician / nutriționist, Rețele sociale (Facebook, Instagram, TikTok, YouTube), Site-uri de internet generale, Familie și prieteni	Încredere ridicată	Încredere ridicată	Neutru	Încredere ridicată	Neutru	Încredere scăzută	Încredere scăzută
P034	Medicul de familie, Rețele sociale (Facebook, Instagram, TikTok, YouTube), Familie și prieteni	Încredere foarte ridicată	Încredere foarte ridicată	Neutru	Neutru	Neutru	Încredere foarte ridicată	Neutru
P035	Televiziune / radio, Rețele sociale (Facebook, Instagram, TikTok, YouTube), Familie și prieteni	Încredere foarte ridicată	Încredere foarte ridicată	Neutru	Neutru	Neutru	Încredere foarte ridicată	Încredere ridicată
P036	Medicul specialist (oncolog, gastroenterolog, alt specialist), Articole științifice / surse academice, Aplicații mobile dedicate sănătății, Televiziune / radio, Rețele sociale (Facebook, Instagram, TikTok, YouTube)	Încredere foarte ridicată	Încredere foarte ridicată	Încredere ridicată	Încredere ridicată	Încredere ridicată	Neutru	Încredere foarte ridicată
P037	Dietetician / nutriționist, Articole științifice / surse academice, Aplicații mobile dedicate sănătății, Familie și prieteni	Încredere ridicată	Încredere foarte ridicată	Neutru	Încredere scăzută	Încredere scăzută	Neutru	Încredere ridicată
P038	Medicul de familie, Articole științifice / surse academice, Rețele sociale (Facebook, Instagram, TikTok, YouTube), Site-uri de internet generale, Familie și prieteni	Neutru	Încredere ridicată	Neutru	Neutru	Neutru	Încredere ridicată	Încredere foarte ridicată
P039	Medicul specialist (oncolog, gastroenterolog, alt specialist), Articole științifice / surse academice	Încredere ridicată	Încredere ridicată	Neutru	Neutru	Încredere scăzută	Încredere scăzută	Încredere ridicată
P040	Rețele sociale (Facebook, Instagram, TikTok, YouTube), Familie și prieteni	Încredere ridicată	Încredere ridicată	Neutru	Încredere scăzută	Încredere scăzută	Încredere ridicată	Încredere ridicată
P041	Dietetician / nutriționist, Articole științifice / surse academice, Aplicații mobile dedicate sănătății, Rețele sociale (Facebook, Instagram, TikTok, YouTube), Reviste și ziare, Site-uri de internet generale, Familie și prieteni	Încredere foarte ridicată	Încredere foarte ridicată	Încredere foarte ridicată	Neutru	Încredere scăzută	Încredere ridicată	Încredere foarte ridicată
P042	Medicul de familie, Medicul specialist (oncolog, gastroenterolog, alt specialist), Articole științifice / surse academice	Încredere foarte ridicată	Încredere foarte ridicată	Neutru	Neutru	Neutru	Neutru	Încredere ridicată
P043	Medicul specialist (oncolog, gastroenterolog, alt specialist), Dietetician / nutriționist, Articole științifice / surse academice	Încredere ridicată	Încredere ridicată	Încredere scăzută	Încredere scăzută	Încredere scăzută	Neutru	Încredere ridicată
P044	Rețele sociale (Facebook, Instagram, TikTok, YouTube)	Încredere foarte scăzută	Încredere foarte scăzută	Încredere foarte ridicată	Încredere foarte ridicată	Încredere foarte scăzută	Încredere foarte scăzută	Încredere foarte scăzută
P045	Articole științifice / surse academice, Aplicații mobile dedicate sănătății, Televiziune / radio, Rețele sociale (Facebook, Instagram, TikTok, YouTube), Familie și prieteni	Încredere ridicată	Încredere ridicată	Neutru	Neutru	Încredere scăzută	Încredere ridicată	Neutru
P046	Articole științifice / surse academice, Aplicații mobile dedicate sănătății, Rețele sociale (Facebook, Instagram, TikTok, YouTube), Site-uri de internet generale	Încredere ridicată	Încredere foarte ridicată	Încredere scăzută	Încredere foarte scăzută	Neutru	Neutru	Încredere ridicată
P047	Medicul de familie, Farmacist, Dietetician / nutriționist	Încredere ridicată	Neutru	Încredere scăzută	Încredere scăzută	Încredere scăzută	Încredere ridicată	Neutru
P048	Aplicații mobile dedicate sănătății, Televiziune / radio, Rețele sociale (Facebook, Instagram, TikTok, YouTube), Site-uri de internet generale, Familie și prieteni	Încredere ridicată	Încredere foarte ridicată	Neutru	Neutru	Neutru	Neutru	Neutru
P049	Medicul de familie, Articole științifice / surse academice, Rețele sociale (Facebook, Instagram, TikTok, YouTube), Familie și prieteni	Încredere foarte ridicată	Încredere foarte ridicată	Încredere scăzută	Neutru	Neutru	Neutru	Încredere foarte ridicată
P050	Articole științifice / surse academice, Aplicații mobile dedicate sănătății, Televiziune / radio, Rețele sociale (Facebook, Instagram, TikTok, YouTube), Site-uri de internet generale, Familie și prieteni	Încredere ridicată	Încredere ridicată	Încredere ridicată	Încredere ridicată	Încredere ridicată	Încredere ridicată	Încredere ridicată
P051	Farmacist, Articole științifice / surse academice, Familie și prieteni	Încredere ridicată	Încredere foarte ridicată	Neutru	Încredere scăzută	Neutru	Încredere ridicată	Încredere foarte ridicată
P052	Dietetician / nutriționist, Articole științifice / surse academice, Aplicații mobile dedicate sănătății, Televiziune / radio, Rețele sociale (Facebook, Instagram, TikTok, YouTube), Site-uri de internet generale	Încredere foarte ridicată	Încredere foarte ridicată	Încredere scăzută	Neutru	Încredere scăzută	Încredere scăzută	Încredere foarte ridicată
P053	Articole științifice / surse academice, Aplicații mobile dedicate sănătății, Rețele sociale (Facebook, Instagram, TikTok, YouTube), Familie și prieteni	Încredere foarte ridicată	Încredere foarte ridicată	Încredere ridicată	Încredere ridicată	Neutru	Încredere foarte ridicată	Încredere foarte ridicată
P054	Televiziune / radio, Rețele sociale (Facebook, Instagram, TikTok, YouTube)	Încredere foarte ridicată	Încredere foarte ridicată	Neutru	Neutru	Neutru	Încredere ridicată	Neutru

Participant_ID	F1	F2a	F2b	F2c	F2d	F2e	F2f	F2g
P055	Medicul de familie, Aplicații mobile dedicate sănătății, Reviste și ziare, Site-uri de internet generale	Încredere foarte scăzută	Neutru	Neutru	Neutru	Neutru	Neutru	Neutru
P056	Medicul de familie, Farmacist, Aplicații mobile dedicate sănătății, Rețele sociale (Facebook, Instagram, TikTok, YouTube), Site-uri de internet generale, Familie și prieteni	Încredere ridicată	Încredere foarte ridicată	Încredere scăzută	Încredere scăzută	Neutru	Încredere scăzută	Încredere foarte ridicată
P057	Familie și prieteni	Încredere scăzută	Încredere scăzută	Încredere foarte scăzută	Încredere foarte scăzută	Încredere foarte scăzută	Încredere foarte ridicată	Neutru
P058	Medicul specialist (oncolog, gastroenterolog, alt specialist), Farmacist, Dietetician / nutriționist, Articole științifice / surse academice	Neutru	Încredere ridicată	Încredere scăzută	Încredere scăzută	Încredere scăzută	Încredere scăzută	Încredere ridicată
P059	Articole științifice / surse academice, Rețele sociale (Facebook, Instagram, TikTok, YouTube), Site-uri de internet generale	Neutru	Încredere ridicată	Neutru	Neutru	Neutru	Încredere ridicată	Încredere ridicată
P060	Medicul specialist (oncolog, gastroenterolog, alt specialist), Articole științifice / surse academice, Rețele sociale (Facebook, Instagram, TikTok, YouTube)	Încredere foarte scăzută	Neutru	Încredere foarte scăzută	Încredere scăzută	Neutru	Încredere scăzută	Încredere foarte scăzută
P061	Medicul de familie, Rețele sociale (Facebook, Instagram, TikTok, YouTube), Site-uri de internet generale, Familie și prieteni	Încredere ridicată	Încredere ridicată	Neutru	Încredere scăzută	Neutru	Încredere ridicată	Încredere foarte ridicată
P062	Medicul de familie, Medicul specialist (oncolog, gastroenterolog, alt specialist), Dietetician / nutriționist, Articole științifice / surse academice, Rețele sociale (Facebook, Instagram, TikTok, YouTube), Site-uri de internet generale	Încredere ridicată	Încredere foarte ridicată	Încredere scăzută	Neutru	Încredere scăzută	Încredere ridicată	Încredere ridicată
P063	Articole științifice / surse academice, Televiziune / radio, Rețele sociale (Facebook, Instagram, TikTok, YouTube), Site-uri de internet generale, Familie și prieteni	Încredere foarte ridicată	Încredere foarte ridicată	Neutru	Neutru	Neutru	Încredere ridicată	Încredere foarte ridicată
P064	Articole științifice / surse academice, Aplicații mobile dedicate sănătății, Site-uri de internet generale	Încredere foarte ridicată	Încredere foarte ridicată	Neutru	Încredere foarte scăzută	Încredere foarte scăzută	Încredere foarte ridicată	Încredere foarte ridicată
P065	Medicul de familie, Farmacist, Articole științifice / surse academice, Aplicații mobile dedicate sănătății, Televiziune / radio, Rețele sociale (Facebook, Instagram, TikTok, YouTube), Site-uri de internet generale, Familie și prieteni	Încredere foarte ridicată	Încredere foarte ridicată	Neutru	Încredere scăzută	Neutru	Neutru	Încredere foarte ridicată
P066	Medicul de familie, Articole științifice / surse academice, Rețele sociale (Facebook, Instagram, TikTok, YouTube)	Încredere foarte ridicată	Încredere foarte ridicată	Încredere scăzută	Încredere scăzută	Încredere foarte scăzută	Încredere scăzută	Încredere foarte ridicată
P067	Articole științifice / surse academice, Rețele sociale (Facebook, Instagram, TikTok, YouTube), Site-uri de internet generale	Neutru	Încredere foarte ridicată	Neutru	Neutru	Încredere foarte scăzută	Încredere foarte scăzută	Încredere foarte ridicată
P068	Rețele sociale (Facebook, Instagram, TikTok, YouTube), Site-uri de internet generale, Familie și prieteni	Încredere ridicată	Încredere ridicată	Încredere ridicată	Încredere ridicată	Neutru	Încredere ridicată	Încredere foarte ridicată
P069	Medicul de familie, Medicul specialist (oncolog, gastroenterolog, alt specialist), Dietetician / nutriționist, Articole științifice / surse academice, Aplicații mobile dedicate sănătății, Site-uri de internet generale, Familie și prieteni	Încredere foarte ridicată	Încredere foarte ridicată	Neutru	Neutru	Neutru	Încredere ridicată	Încredere foarte ridicată
P070	Articole științifice / surse academice, Site-uri de internet generale	Neutru	Neutru	Neutru	Neutru	Neutru	Neutru	Încredere foarte ridicată
P071	Dietetician / nutriționist, Articole științifice / surse academice, Site-uri de internet generale, Familie și prieteni	Încredere ridicată	Încredere foarte ridicată	Neutru	Neutru	Încredere foarte scăzută	Încredere ridicată	Încredere foarte ridicată
P072	Articole științifice / surse academice, Site-uri de internet generale, Familie și prieteni	Încredere foarte ridicată	Încredere foarte ridicată	Neutru	Neutru	Neutru	Încredere foarte ridicată	Încredere ridicată
P073	Medicul specialist (oncolog, gastroenterolog, alt specialist), Articole științifice / surse academice, Aplicații mobile dedicate sănătății, Rețele sociale (Facebook, Instagram, TikTok, YouTube), Site-uri de internet generale	Încredere ridicată	Încredere foarte ridicată	Încredere scăzută	Încredere scăzută	Încredere scăzută	Neutru	Încredere foarte ridicată
P074	Medicul de familie, Articole științifice / surse academice, Televiziune / radio, Rețele sociale (Facebook, Instagram, TikTok, YouTube), Reviste și ziare, Site-uri de internet generale	Neutru	Neutru	Încredere scăzută	Încredere scăzută	Încredere scăzută	Neutru	Încredere ridicată
P075	Articole științifice / surse academice, Aplicații mobile dedicate sănătății, Rețele sociale (Facebook, Instagram, TikTok, YouTube), Site-uri de internet generale	Neutru	Încredere foarte ridicată	Încredere foarte ridicată	Încredere scăzută	Încredere scăzută	Încredere scăzută	Încredere foarte ridicată
P076	Articole științifice / surse academice, Aplicații mobile dedicate sănătății, Rețele sociale (Facebook, Instagram, TikTok, YouTube), Site-uri de internet generale, Familie și prieteni	Neutru	Încredere ridicată	Neutru	Neutru	Încredere scăzută	Neutru	Încredere foarte ridicată
P077	Rețele sociale (Facebook, Instagram, TikTok, YouTube), Site-uri de internet generale, Familie și prieteni	Încredere ridicată	Încredere ridicată	Încredere ridicată	Neutru	Neutru	Neutru	Încredere foarte ridicată
P078	Medicul de familie, Medicul specialist (oncolog, gastroenterolog, alt specialist), Articole științifice / surse academice	Încredere foarte ridicată	Încredere foarte ridicată	Încredere ridicată	Încredere ridicată	Încredere scăzută	Încredere ridicată	Încredere foarte ridicată

Participant_ ID	F1	F2a	F2b	F2c	F2d	F2e	F2f	F2g
P079	Articole științifice / surse academice, Aplicații mobile dedicate sănătății, Televiziune / radio, Rețele sociale (Facebook, Instagram, TikTok, YouTube), Site-uri de internet generale, Familie și prieteni	Neutru	Încredere foarte ridicată	Încredere ridicată	Încredere ridicată	Neutru	Încredere ridicată	Încredere foarte ridicată
P080	Medicul de familie, Dietetician / nutriționist, Articole științifice / surse academice, Aplicații mobile dedicate sănătății, Rețele sociale (Facebook, Instagram, TikTok, YouTube), Reviste și ziare	Neutru	Neutru	Neutru	Neutru	Neutru	Neutru	Neutru
P081	Medicul de familie, Aplicații mobile dedicate sănătății, Rețele sociale (Facebook, Instagram, TikTok, YouTube), Familie și prieteni	Încredere foarte ridicată	Încredere foarte ridicată	Neutru	Neutru	Neutru	Încredere foarte ridicată	Încredere foarte ridicată
P082	Articole științifice / surse academice, Televiziune / radio, Rețele sociale (Facebook, Instagram, TikTok, YouTube), Site-uri de internet generale, Familie și prieteni	Neutru	Neutru	Neutru	Încredere ridicată	Neutru	Încredere ridicată	Încredere ridicată
P083	Articole științifice / surse academice, Aplicații mobile dedicate sănătății, Televiziune / radio	Încredere foarte ridicată	Încredere foarte ridicată	Încredere scăzută	Încredere scăzută	Încredere scăzută	Încredere scăzută	Încredere foarte ridicată
P084	Medicul de familie, Articole științifice / surse academice, Reviste și ziare	Încredere ridicată	Încredere foarte ridicată	Neutru	Încredere scăzută	Neutru	Neutru	Încredere foarte ridicată
P085	Medicul de familie, Medicul specialist (oncolog, gastroenterolog, alt specialist), Articole științifice / surse academice, Aplicații mobile dedicate sănătății, Televiziune / radio	Încredere ridicată	Încredere foarte ridicată	Neutru	Neutru	Neutru	Încredere ridicată	Încredere foarte ridicată
P086	Articole științifice / surse academice	Încredere ridicată	Încredere ridicată	Încredere scăzută	Încredere foarte scăzută	Încredere foarte scăzută	Încredere foarte scăzută	Încredere ridicată
P087	Dietetician / nutriționist, Articole științifice / surse academice, Rețele sociale (Facebook, Instagram, TikTok, YouTube)	Încredere ridicată	Încredere ridicată	Neutru	Neutru	Neutru	Neutru	Încredere ridicată
P088	Medicul specialist (oncolog, gastroenterolog, alt specialist), Aplicații mobile dedicate sănătății, Televiziune / radio, Rețele sociale (Facebook, Instagram, TikTok, YouTube), Familie și prieteni	Neutru	Încredere ridicată	Încredere scăzută	Încredere scăzută	Încredere scăzută	Încredere scăzută	Neutru
P089	Farmacist, Dietetician / nutriționist, Articole științifice / surse academice, Televiziune / radio	Încredere foarte scăzută	Încredere ridicată	Încredere ridicată	Încredere foarte ridicată	Neutru	Neutru	Încredere foarte ridicată
P090	Medicul de familie, Dietetician / nutriționist	Încredere ridicată	Încredere foarte ridicată	Neutru	Încredere scăzută	Încredere scăzută	Neutru	Încredere foarte ridicată
P091	Medicul de familie, Televiziune / radio, Rețele sociale (Facebook, Instagram, TikTok, YouTube), Familie și prieteni	Încredere ridicată	Încredere ridicată	Neutru	Neutru	Neutru	Încredere scăzută	Încredere foarte ridicată
P092	Medicul specialist (oncolog, gastroenterolog, alt specialist), Articole științifice / surse academice, Televiziune / radio, Rețele sociale (Facebook, Instagram, TikTok, YouTube), Familie și prieteni	Încredere ridicată	Încredere foarte ridicată	Încredere ridicată	Încredere scăzută	Neutru	Neutru	Încredere foarte ridicată
P093	Dietetician / nutriționist, Rețele sociale (Facebook, Instagram, TikTok, YouTube), Reviste și ziare, Site-uri de internet generale, Familie și prieteni	Încredere ridicată	Încredere ridicată	Neutru	Neutru	Neutru	Încredere ridicată	Încredere ridicată
P094	Rețele sociale (Facebook, Instagram, TikTok, YouTube), Reviste și ziare, Site-uri de internet generale, Familie și prieteni	Încredere foarte ridicată	Încredere foarte ridicată	Neutru	Neutru	Încredere ridicată	Încredere ridicată	Încredere ridicată
P095	Medicul de familie, Medicul specialist (oncolog, gastroenterolog, alt specialist), Dietetician / nutriționist, Articole științifice / surse academice, Aplicații mobile dedicate sănătății, Rețele sociale (Facebook, Instagram, TikTok, YouTube), Familie și prieteni	Încredere ridicată	Încredere foarte ridicată	Neutru	Neutru	Neutru	Neutru	Încredere foarte ridicată
P096	Articole științifice / surse academice, Aplicații mobile dedicate sănătății, Rețele sociale (Facebook, Instagram, TikTok, YouTube)	Încredere ridicată	Încredere ridicată	Neutru	Neutru	Încredere scăzută	Neutru	Încredere ridicată
P097	Medicul de familie, Farmacist, Dietetician / nutriționist, Articole științifice / surse academice	Încredere foarte ridicată	Încredere foarte ridicată	Neutru	Neutru	Încredere ridicată	Încredere ridicată	Încredere ridicată
P098	Medicul de familie, Aplicații mobile dedicate sănătății, Site-uri de internet generale	Neutru	Neutru	Neutru	Neutru	Neutru	Neutru	Neutru
P099	Articole științifice / surse academice, Aplicații mobile dedicate sănătății, Rețele sociale (Facebook, Instagram, TikTok, YouTube), Site-uri de internet generale	Neutru	Neutru	Neutru	Neutru	Neutru	Neutru	Neutru
P100	Aplicații mobile dedicate sănătății	Neutru	Neutru	Neutru	Neutru	Neutru	Neutru	Încredere foarte ridicată
P101	Medicul de familie, Rețele sociale (Facebook, Instagram, TikTok, YouTube), Familie și prieteni	Încredere foarte ridicată	Încredere foarte ridicată	Neutru	Încredere scăzută	Neutru	Încredere foarte ridicată	Încredere foarte ridicată
P102	Medicul specialist (oncolog, gastroenterolog, alt specialist), Articole științifice / surse academice	Încredere foarte ridicată	Încredere foarte ridicată	Neutru	Neutru	Încredere scăzută	Încredere foarte scăzută	Încredere foarte ridicată
P103	Aplicații mobile dedicate sănătății, Rețele sociale (Facebook, Instagram, TikTok, YouTube), Familie și prieteni	Încredere ridicată	Încredere foarte ridicată	Neutru	Neutru	Neutru	Încredere ridicată	Încredere foarte ridicată
P104	Medicul de familie, Medicul specialist (oncolog, gastroenterolog, alt specialist), Dietetician / nutriționist, Articole științifice / surse academice	Neutru	Încredere ridicată	Încredere scăzută	Încredere scăzută	Încredere scăzută	Încredere scăzută	Încredere ridicată
P105	Aplicații mobile dedicate sănătății, Rețele sociale (Facebook, Instagram, TikTok, YouTube)	Încredere ridicată	Încredere ridicată	Încredere ridicată	Neutru	Neutru	Neutru	Încredere ridicată

Participant_ ID	F1	F2a	F2b	F2c	F2d	F2e	F2f	F2g
P106	Medicul de familie, Articole științifice / surse academice, Rețele sociale (Facebook, Instagram, TikTok, YouTube), Site-uri de internet generale, Familie și prieteni	Încredere ridicată	Încredere foarte ridicată	Neutru	Neutru	Încredere scăzută	Neutru	Încredere ridicată
P107	Site-uri de internet generale	Încredere ridicată	Încredere ridicată	Încredere ridicată	Încredere ridicată	Încredere ridicată	Încredere ridicată	Încredere ridicată
P108	Medicul de familie, Farmacist, Televiziune / radio, Rețele sociale (Facebook, Instagram, TikTok, YouTube)	Încredere ridicată	Încredere ridicată	Neutru	Neutru	Neutru	Neutru	Încredere ridicată
P109	Articole științifice / surse academice, Site-uri de internet generale	Încredere ridicată	Încredere ridicată	Neutru	Neutru	Neutru	Neutru	Încredere foarte ridicată
P110	Aplicații mobile dedicate sănătății, Televiziune / radio, Rețele sociale (Facebook, Instagram, TikTok, YouTube), Reviste și ziare, Site-uri de internet generale, Familie și prieteni	Încredere ridicată	Încredere ridicată	Neutru	Neutru	Neutru	Neutru	Încredere foarte ridicată
P111	Rețele sociale (Facebook, Instagram, TikTok, YouTube), Site-uri de internet generale, Familie și prieteni	Neutru	Încredere ridicată	Neutru	Încredere scăzută	Încredere scăzută	Neutru	Încredere ridicată
P112	Televiziune / radio, Rețele sociale (Facebook, Instagram, TikTok, YouTube)	Încredere foarte ridicată	Neutru	Neutru	Neutru	Neutru	Neutru	Încredere foarte ridicată
P113	Articole științifice / surse academice, Aplicații mobile dedicate sănătății, Rețele sociale (Facebook, Instagram, TikTok, YouTube)	Încredere foarte ridicată	Încredere foarte ridicată	Neutru	Neutru	Neutru	Încredere ridicată	Încredere foarte ridicată
P114	Medicul de familie, Medicul specialist (oncolog, gastroenterolog, alt specialist), Farmacist, Rețele sociale (Facebook, Instagram, TikTok, YouTube), Site-uri de internet generale	Încredere foarte ridicată	Încredere ridicată	Încredere scăzută	Încredere scăzută	Încredere scăzută	Încredere scăzută	Încredere foarte ridicată
P115	Dietetician / nutriționist, Articole științifice / surse academice	Neutru	Încredere foarte ridicată	Neutru	Neutru	Neutru	Neutru	Neutru
P116	Medicul de familie, Medicul specialist (oncolog, gastroenterolog, alt specialist), Articole științifice / surse academice, Site-uri de internet generale	Încredere ridicată	Neutru	Neutru	Încredere scăzută	Încredere scăzută	Neutru	Încredere ridicată
P117	Medicul de familie, Medicul specialist (oncolog, gastroenterolog, alt specialist), Dietetician / nutriționist, Articole științifice / surse academice, Reviste și ziare, Site-uri de internet generale, Familie și prieteni	Încredere ridicată	Încredere ridicată	Încredere scăzută	Încredere scăzută	Încredere scăzută	Încredere foarte ridicată	Încredere foarte ridicată
P118	Dietetician / nutriționist, Articole științifice / surse academice, Familie și prieteni	Încredere foarte ridicată	Încredere foarte ridicată	Neutru	Neutru	Neutru	Încredere ridicată	Încredere foarte ridicată
P119	Medicul de familie, Articole științifice / surse academice, Televiziune / radio	Încredere ridicată	Încredere ridicată	Încredere ridicată	Încredere scăzută	Neutru	Neutru	Încredere ridicată
P120	Medicul specialist (oncolog, gastroenterolog, alt specialist), Articole științifice / surse academice	Neutru	Neutru	Încredere foarte scăzută	Încredere foarte scăzută	Încredere scăzută	Încredere foarte scăzută	Încredere foarte ridicată
P121	Medicul specialist (oncolog, gastroenterolog, alt specialist), Articole științifice / surse academice	Neutru	Încredere ridicată	Neutru	Neutru	Neutru	Neutru	Neutru
P122	Dietetician / nutriționist, Articole științifice / surse academice, Familie și prieteni	Neutru	Încredere foarte ridicată	Încredere foarte scăzută	Încredere foarte scăzută	Încredere foarte scăzută	Neutru	Încredere foarte ridicată
P123	Articole științifice / surse academice	Încredere ridicată	Neutru	Încredere scăzută	Încredere foarte scăzută	Încredere scăzută	Încredere foarte scăzută	Încredere foarte ridicată
P124	Rețele sociale (Facebook, Instagram, TikTok, YouTube), Site-uri de internet generale	Încredere foarte ridicată	Încredere foarte ridicată	Neutru	Încredere ridicată	Neutru	Neutru	Încredere foarte ridicată
P125	Aplicații mobile dedicate sănătății, Rețele sociale (Facebook, Instagram, TikTok, YouTube), Site-uri de internet generale	Neutru	Încredere ridicată	Neutru	Neutru	Neutru	Neutru	Încredere ridicată
P126	Articole științifice / surse academice	Încredere foarte ridicată	Încredere foarte ridicată	Încredere scăzută	Încredere foarte scăzută	Încredere foarte scăzută	Neutru	Încredere foarte ridicată
P127	Rețele sociale (Facebook, Instagram, TikTok, YouTube), Site-uri de internet generale	Încredere ridicată	Încredere scăzută	Neutru	Încredere foarte scăzută	Neutru	Încredere ridicată	Încredere foarte ridicată
P128	Medicul specialist (oncolog, gastroenterolog, alt specialist), Articole științifice / surse academice	Neutru	Neutru	Neutru	Încredere foarte scăzută	Încredere foarte scăzută	Încredere scăzută	Încredere foarte ridicată
P129	Medicul de familie, Medicul specialist (oncolog, gastroenterolog, alt specialist), Dietetician / nutriționist, Articole științifice / surse academice	Încredere ridicată	Încredere foarte ridicată	Neutru	Încredere scăzută	Neutru	Neutru	Încredere foarte ridicată
P130	Dietetician / nutriționist, Rețele sociale (Facebook, Instagram, TikTok, YouTube), Site-uri de internet generale	Încredere foarte ridicată	Încredere foarte ridicată	Încredere scăzută	Încredere scăzută	Încredere ridicată	Neutru	Încredere foarte ridicată
P131	Articole științifice / surse academice, Televiziune / radio, Rețele sociale (Facebook, Instagram, TikTok, YouTube), Site-uri de internet generale, Familie și prieteni	Neutru	Încredere ridicată	Neutru	Neutru	Neutru	Încredere ridicată	Încredere foarte ridicată
P132	Rețele sociale (Facebook, Instagram, TikTok, YouTube), Familie și prieteni	Încredere ridicată	Încredere foarte ridicată	Încredere scăzută	Încredere ridicată	Încredere scăzută	Încredere ridicată	Încredere foarte ridicată
P133	Dietetician / nutriționist	Încredere ridicată	Încredere ridicată	Neutru	Încredere scăzută	Încredere scăzută	Neutru	Încredere ridicată
P134	Medicul de familie, Site-uri de internet generale, Familie și prieteni	Încredere ridicată	Neutru	Neutru	Neutru	Neutru	Încredere scăzută	Încredere foarte ridicată
P135	Reviste și ziare	Încredere scăzută	Încredere scăzută	Încredere scăzută	Încredere scăzută	Încredere scăzută	Încredere scăzută	Încredere scăzută
P136	Articole științifice / surse academice	Neutru	Încredere ridicată	Neutru	Încredere scăzută	Încredere scăzută	Încredere scăzută	Încredere foarte ridicată
P137	Articole științifice / surse academice	Încredere scăzută	Încredere ridicată	Încredere scăzută	Încredere foarte scăzută	Neutru	Neutru	Încredere foarte ridicată
P138	Articole științifice / surse academice, Site-uri de internet generale	Încredere ridicată	Încredere ridicată	Încredere ridicată	Neutru	Neutru	Încredere ridicată	Încredere foarte ridicată
P139	Articole științifice / surse academice, Aplicații mobile dedicate sănătății, Rețele sociale (Facebook, Instagram, TikTok, YouTube), Familie și prieteni	Neutru	Neutru	Neutru	Neutru	Neutru	Neutru	Neutru
P140	Articole științifice / surse academice, Familie și prieteni	Încredere ridicată	Încredere ridicată	Neutru	Încredere scăzută	Încredere scăzută	Încredere ridicată	Încredere foarte ridicată

Participant_ID	F1	F2a	F2b	F2c	F2d	F2e	F2f	F2g
P141	Medicul specialist (oncolog, gastroenterolog, alt specialist), Dietetician / nutriționist, Articole științifice / surse academice, Reviste și ziare, Familie și prieteni	Încredere ridicată	Încredere foarte ridicată	Încredere ridicată	Încredere ridicată	Neutru	Neutru	Încredere ridicată
P142	Medicul de familie, Articole științifice / surse academice, Familie și prieteni	Încredere ridicată	Neutru	Încredere foarte scăzută	Încredere foarte scăzută	Încredere foarte scăzută	Încredere foarte ridicată	Încredere foarte ridicată
P143	Dietetician / nutriționist, Rețele sociale (Facebook, Instagram, TikTok, YouTube)	Neutru	Încredere ridicată	Neutru	Încredere ridicată	Neutru	Neutru	Încredere ridicată
P144	Articole științifice / surse academice, Aplicații mobile dedicate sănătății, Rețele sociale (Facebook, Instagram, TikTok, YouTube), Familie și prieteni	Încredere foarte ridicată	Încredere ridicată	Neutru	Încredere scăzută	Încredere scăzută	Neutru	Încredere foarte ridicată
P145	Articole științifice / surse academice	Neutru	Neutru	Încredere foarte scăzută	Încredere foarte scăzută	Încredere foarte scăzută	Încredere scăzută	Încredere foarte ridicată
P146	Medicul specialist (oncolog, gastroenterolog, alt specialist), Farmacist, Dietetician / nutriționist, Articole științifice / surse academice, Aplicații mobile dedicate sănătății, Rețele sociale (Facebook, Instagram, TikTok, YouTube), Familie și prieteni	Încredere foarte ridicată	Încredere foarte ridicată	Încredere scăzută	Încredere foarte scăzută	Încredere foarte scăzută	Neutru	Încredere foarte ridicată
P147	Articole științifice / surse academice, Aplicații mobile dedicate sănătății, Rețele sociale (Facebook, Instagram, TikTok, YouTube), Site-uri de internet generale, Familie și prieteni	Încredere foarte scăzută	Neutru	Neutru	Neutru	Neutru	Neutru	Încredere ridicată
P148	Articole științifice / surse academice	Încredere ridicată	Încredere ridicată	Neutru	Neutru	Încredere scăzută	Neutru	Încredere ridicată
P149	Articole științifice / surse academice, Rețele sociale (Facebook, Instagram, TikTok, YouTube)	Neutru	Neutru	Neutru	Neutru	Încredere scăzută	Neutru	Încredere foarte ridicată
P150	Articole științifice / surse academice, Televiziune / radio, Rețele sociale (Facebook, Instagram, TikTok, YouTube), Reviste și ziare, Site-uri de internet generale	Încredere ridicată	Încredere ridicată	Încredere scăzută	Încredere scăzută	Încredere scăzută	Neutru	Încredere ridicată
P151	Rețele sociale (Facebook, Instagram, TikTok, YouTube), Site-uri de internet generale, Familie și prieteni	Încredere foarte scăzută	Încredere ridicată	Neutru	Încredere ridicată	Neutru	Neutru	Încredere foarte ridicată
P152	Medicul specialist (oncolog, gastroenterolog, alt specialist), Dietetician / nutriționist, Articole științifice / surse academice	Încredere ridicată	Încredere ridicată	Încredere foarte scăzută	Încredere foarte scăzută	Încredere foarte scăzută	Neutru	Încredere foarte ridicată
P153	Familie și prieteni	Încredere scăzută	Încredere scăzută	Încredere scăzută	Încredere scăzută	Încredere scăzută	Încredere scăzută	Neutru
P154	Aplicații mobile dedicate sănătății, Rețele sociale (Facebook, Instagram, TikTok, YouTube)	Neutru	Încredere ridicată	Neutru	Neutru	Neutru	Neutru	Încredere ridicată
P155	Televiziune / radio, Rețele sociale (Facebook, Instagram, TikTok, YouTube), Familie și prieteni	Încredere foarte ridicată	Încredere foarte ridicată	Neutru	Încredere ridicată	Încredere ridicată	Încredere ridicată	Încredere ridicată
P156	Farmacist, Dietetician / nutriționist, Articole științifice / surse academice, Aplicații mobile dedicate sănătății, Reviste și ziare, Site-uri de internet generale, Familie și prieteni	Neutru	Neutru	Neutru	Neutru	Neutru	Neutru	Neutru
P157	Dietetician / nutriționist, Rețele sociale (Facebook, Instagram, TikTok, YouTube)	Încredere scăzută	Încredere ridicată	Încredere ridicată	Încredere ridicată	Încredere scăzută	Încredere ridicată	Încredere ridicată
P158	Medicul specialist (oncolog, gastroenterolog, alt specialist), Dietetician / nutriționist, Site-uri de internet generale	Încredere foarte scăzută	Încredere foarte scăzută	Neutru	Încredere scăzută	Încredere scăzută	Neutru	Încredere foarte scăzută
P159	Dietetician / nutriționist, Aplicații mobile dedicate sănătății, Rețele sociale (Facebook, Instagram, TikTok, YouTube), Site-uri de internet generale, Familie și prieteni	Încredere ridicată	Încredere foarte ridicată	Neutru	Neutru	Neutru	Neutru	Încredere foarte ridicată
P160	Articole științifice / surse academice, Rețele sociale (Facebook, Instagram, TikTok, YouTube)	Încredere ridicată	Încredere ridicată	Încredere foarte scăzută	Neutru	Încredere scăzută	Neutru	Încredere foarte ridicată
P161	Aplicații mobile dedicate sănătății, Televiziune / radio, Rețele sociale (Facebook, Instagram, TikTok, YouTube), Reviste și ziare, Familie și prieteni	Încredere ridicată	Încredere ridicată	Încredere ridicată	Încredere ridicată	Încredere ridicată	Neutru	Încredere foarte ridicată
P162	Dietetician / nutriționist, Site-uri de internet generale	Încredere ridicată	Încredere ridicată	Încredere scăzută	Încredere scăzută	Neutru	Neutru	Încredere ridicată
P163	Articole științifice / surse academice	Încredere scăzută	Încredere ridicată	Încredere ridicată	Încredere ridicată	Încredere ridicată	Încredere ridicată	Încredere ridicată
P164	Articole științifice / surse academice	Neutru	Încredere scăzută	Încredere scăzută	Încredere scăzută	Încredere scăzută	Încredere scăzută	Neutru
P165	Medicul de familie, Medicul specialist (oncolog, gastroenterolog, alt specialist), Dietetician / nutriționist, Articole științifice / surse academice, Rețele sociale (Facebook, Instagram, TikTok, YouTube), Reviste și ziare	Încredere foarte ridicată	Încredere foarte ridicată	Neutru	Neutru	Încredere ridicată	Neutru	Încredere foarte ridicată
P166	Medicul de familie, Medicul specialist (oncolog, gastroenterolog, alt specialist)	Încredere ridicată	Încredere ridicată	Încredere scăzută	Încredere scăzută	Încredere scăzută	Încredere ridicată	Încredere ridicată
P167	Articole științifice / surse academice	Încredere ridicată	Încredere ridicată	Neutru	Neutru	Neutru	Încredere ridicată	Încredere ridicată
P168	Articole științifice / surse academice, Aplicații mobile dedicate sănătății, Televiziune / radio	Neutru	Încredere ridicată	Neutru	Neutru	Încredere ridicată	Neutru	Încredere ridicată
P169	Dietetician / nutriționist, Site-uri de internet generale, Familie și prieteni	Neutru	Încredere ridicată	Neutru	Neutru	Neutru	Încredere ridicată	Încredere ridicată
P170	Medicul specialist (oncolog, gastroenterolog, alt specialist), Dietetician / nutriționist	Încredere ridicată	Încredere ridicată	Neutru	Încredere scăzută	Încredere scăzută	Încredere ridicată	Încredere ridicată
P171	Televiziune / radio, Rețele sociale (Facebook, Instagram, TikTok, YouTube), Site-uri de internet generale, Familie și prieteni	Neutru	Încredere ridicată	Neutru	Neutru	Neutru	Neutru	Încredere foarte ridicată
P172	Articole științifice / surse academice	Neutru	Neutru	Încredere scăzută	Încredere scăzută	Încredere scăzută	Încredere ridicată	Încredere foarte ridicată

Participant_ ID	F1	F2a	F2b	F2c	F2d	F2e	F2f	F2g
P173	Medicul de familie, Farmacist, Dietetician / nutriționist, Site-uri de internet generale, Familie și prieteni	Încredere foarte ridicată	Neutru	Încredere ridicată	Încredere ridicată	Încredere ridicată	Neutru	Neutru
P174	Articole științifice / surse academice, Aplicații mobile dedicate sănătății, Rețele sociale (Facebook, Instagram, TikTok, YouTube), Reviste și ziare, Site-uri de internet generale, Familie și prieteni	Încredere ridicată	Încredere foarte ridicată	Încredere ridicată	Încredere ridicată	Încredere ridicată	Încredere ridicată	Încredere ridicată
P175	Articole științifice / surse academice, Aplicații mobile dedicate sănătății, Site-uri de internet generale	Încredere ridicată	Neutru	Încredere scăzută	Încredere scăzută	Încredere scăzută	Încredere scăzută	Încredere ridicată
P176	Aplicații mobile dedicate sănătății, Televiziune / radio, Reviste și ziare	Neutru	Neutru	Neutru	Neutru	Neutru	Neutru	Neutru
P177	Televiziune / radio, Familie și prieteni	Neutru	Încredere ridicată	Încredere scăzută	Încredere scăzută	Încredere scăzută	Neutru	Încredere ridicată
P178	Medicul specialist (oncolog, gastroenterolog, alt specialist), Farmacist, Televiziune / radio, Rețele sociale (Facebook, Instagram, TikTok, YouTube), Reviste și ziare, Site-uri de internet generale	Încredere ridicată	Încredere ridicată	Încredere ridicată	Încredere ridicată	Încredere ridicată	Încredere ridicată	Încredere ridicată
P179	Rețele sociale (Facebook, Instagram, TikTok, YouTube)	Încredere ridicată	Încredere ridicată	Neutru	Neutru	Neutru	Încredere ridicată	Încredere ridicată
P180	Medicul de familie	Încredere ridicată	Neutru	Neutru	Încredere foarte scăzută	Încredere foarte scăzută	Încredere foarte scăzută	Încredere ridicată
P181	Medicul specialist (oncolog, gastroenterolog, alt specialist)	Încredere ridicată	Neutru	Încredere scăzută	Neutru	Încredere scăzută	Neutru	Încredere ridicată
P182	Medicul de familie, Medicul specialist (oncolog, gastroenterolog, alt specialist), Dietetician / nutriționist, Reviste și ziare, Familie și prieteni	Încredere ridicată	Încredere ridicată	Încredere scăzută	Neutru	Încredere scăzută	Neutru	Încredere ridicată
P183	Medicul specialist (oncolog, gastroenterolog, alt specialist), Aplicații mobile dedicate sănătății	Neutru	Încredere ridicată	Încredere foarte scăzută	Încredere foarte scăzută	Încredere scăzută	Neutru	Încredere ridicată
P184	Medicul de familie, Medicul specialist (oncolog, gastroenterolog, alt specialist), Dietetician / nutriționist, Articole științifice / surse academice, Reviste și ziare, Site-uri de internet generale, Familie și prieteni	Încredere ridicată	Încredere ridicată	Neutru	Încredere scăzută	Încredere foarte scăzută	Încredere scăzută	Încredere ridicată
P185	Rețele sociale (Facebook, Instagram, TikTok, YouTube)	Neutru	Neutru	Neutru	Neutru	Neutru	Neutru	Neutru
P186	Dietetician / nutriționist, Articole științifice / surse academice, Aplicații mobile dedicate sănătății, Rețele sociale (Facebook, Instagram, TikTok, YouTube)	Neutru	Încredere ridicată	Neutru	Încredere ridicată	Neutru	Încredere scăzută	Încredere ridicată
P187	Aplicații mobile dedicate sănătății, Televiziune / radio, Rețele sociale (Facebook, Instagram, TikTok, YouTube), Familie și prieteni	Încredere ridicată	Încredere ridicată	Neutru	Neutru	Neutru	Neutru	Încredere ridicată
P188	Articole științifice / surse academice, Site-uri de internet generale	Încredere ridicată	Încredere ridicată	Neutru	Încredere foarte scăzută	Neutru	Încredere scăzută	Încredere foarte ridicată
P189	Articole științifice / surse academice	Încredere ridicată	Neutru	Neutru	Încredere foarte scăzută	Neutru	Neutru	Încredere foarte ridicată
P190	Articole științifice / surse academice, Aplicații mobile dedicate sănătății, Televiziune / radio, Rețele sociale (Facebook, Instagram, TikTok, YouTube), Site-uri de internet generale	Încredere ridicată	Încredere foarte ridicată	Neutru	Neutru	Neutru	Neutru	Încredere foarte ridicată
P191	Rețele sociale (Facebook, Instagram, TikTok, YouTube), Site-uri de internet generale, Familie și prieteni	Neutru	Încredere ridicată	Încredere ridicată	Încredere ridicată	Încredere ridicată	Încredere ridicată	Încredere ridicată
P192	Rețele sociale (Facebook, Instagram, TikTok, YouTube), Reviste și ziare, Familie și prieteni	Neutru	Neutru	Neutru	Neutru	Neutru	Neutru	Încredere ridicată
P193	Medicul de familie, Articole științifice / surse academice, Aplicații mobile dedicate sănătății	Încredere ridicată	Încredere ridicată	Încredere scăzută	Încredere foarte scăzută	Încredere scăzută	Neutru	Încredere ridicată
P194	Medicul de familie, Site-uri de internet generale, Familie și prieteni	Încredere foarte ridicată	Încredere foarte ridicată	Încredere ridicată	Neutru	Neutru	Neutru	Încredere foarte ridicată
P195	Articole științifice / surse academice, Aplicații mobile dedicate sănătății, Rețele sociale (Facebook, Instagram, TikTok, YouTube)	Încredere foarte ridicată	Încredere foarte ridicată	Încredere ridicată	Neutru	Neutru	Neutru	Încredere foarte ridicată
P196	Televiziune / radio, Rețele sociale (Facebook, Instagram, TikTok, YouTube), Site-uri de internet generale, Familie și prieteni	Neutru	Neutru	Neutru	Neutru	Neutru	Neutru	Neutru
P197	Medicul specialist (oncolog, gastroenterolog, alt specialist)	Încredere foarte ridicată	Încredere foarte ridicată	Neutru	Neutru	Neutru	Încredere ridicată	Încredere foarte ridicată
P198	Familie și prieteni	Încredere ridicată	Încredere ridicată	Neutru	Neutru	Neutru	Încredere foarte ridicată	Încredere foarte ridicată
P199	Televiziune / radio, Rețele sociale (Facebook, Instagram, TikTok, YouTube), Reviste și ziare, Site-uri de internet generale, Familie și prieteni	Încredere ridicată	Neutru	Neutru	Neutru	Neutru	Neutru	Încredere ridicată
P200	Medicul de familie, Medicul specialist (oncolog, gastroenterolog, alt specialist), Rețele sociale (Facebook, Instagram, TikTok, YouTube)	Neutru	Încredere ridicată	Neutru	Neutru	Neutru	Încredere ridicată	Încredere ridicată
P201	Articole științifice / surse academice, Aplicații mobile dedicate sănătății, Televiziune / radio, Rețele sociale (Facebook, Instagram, TikTok, YouTube), Reviste și ziare, Site-uri de internet generale	Încredere ridicată	Încredere ridicată	Încredere ridicată	Încredere scăzută	Încredere scăzută	Încredere scăzută	Încredere ridicată
P202	Medicul de familie, Aplicații mobile dedicate sănătății, Televiziune / radio, Rețele sociale (Facebook, Instagram, TikTok, YouTube), Familie și prieteni	Încredere foarte ridicată	Încredere foarte ridicată	Neutru	Neutru	Încredere ridicată	Încredere ridicată	Încredere foarte ridicată

Participant_ID	F1	F2a	F2b	F2c	F2d	F2e	F2f	F2g
P203	Medicul de familie, Medicul specialist (oncolog, gastroenterolog, alt specialist), Dietetician / nutriționist, Articole științifice / surse academice, Aplicații mobile dedicate sănătății	Încredere foarte ridicată	Încredere foarte ridicată	Neutru	Neutru	Încredere foarte scăzută	Neutru	Încredere foarte ridicată
P204	Dietetician / nutriționist	Încredere ridicată	Încredere ridicată	Încredere ridicată	Încredere ridicată	Încredere ridicată	Încredere ridicată	Încredere ridicată
P205	Medicul de familie	Încredere ridicată	Neutru	Încredere scăzută	Încredere foarte scăzută	Încredere scăzută	Încredere scăzută	Neutru
P206	Medicul de familie, Farmacist, Dietetician / nutriționist, Articole științifice / surse academice, Aplicații mobile dedicate sănătății	Încredere ridicată	Încredere ridicată	Încredere ridicată	Neutru	Neutru	Neutru	Încredere foarte ridicată
P207	Articole științifice / surse academice, Aplicații mobile dedicate sănătății, Rețele sociale (Facebook, Instagram, TikTok, YouTube), Site-uri de internet generale, Familie și prieteni	Încredere foarte ridicată	Încredere foarte ridicată	Neutru	Neutru	Neutru	Neutru	Încredere foarte ridicată
P208	Medicul de familie	Încredere ridicată	Încredere ridicată	Încredere scăzută	Încredere scăzută	Încredere scăzută	Încredere ridicată	Încredere ridicată
P209	Aplicații mobile dedicate sănătății, Reviste și ziare, Familie și prieteni	Încredere ridicată	Încredere ridicată	Încredere ridicată	Încredere ridicată	Încredere ridicată	Încredere ridicată	Încredere ridicată
P210	Aplicații mobile dedicate sănătății, Rețele sociale (Facebook, Instagram, TikTok, YouTube), Familie și prieteni	Încredere ridicată	Încredere ridicată	Încredere ridicată	Încredere scăzută	Încredere foarte scăzută	Încredere ridicată	Încredere ridicată
P211	Articole științifice / surse academice, Site-uri de internet generale, Familie și prieteni	Încredere scăzută	Neutru	Neutru	Neutru	Neutru	Neutru	Încredere ridicată
P212	Articole științifice / surse academice, Aplicații mobile dedicate sănătății, Televiziune / radio, Rețele sociale (Facebook, Instagram, TikTok, YouTube), Reviste și ziare, Site-uri de internet generale, Familie și prieteni	Neutru	Încredere ridicată	Neutru	Neutru	Neutru	Încredere ridicată	Încredere foarte ridicată
P213	Dietetician / nutriționist, Rețele sociale (Facebook, Instagram, TikTok, YouTube)	Încredere ridicată	Încredere ridicată	Încredere ridicată	Încredere ridicată	Încredere ridicată	Încredere ridicată	Încredere foarte ridicată
P214	Articole științifice / surse academice, Rețele sociale (Facebook, Instagram, TikTok, YouTube), Site-uri de internet generale	Neutru	Încredere ridicată	Încredere ridicată	Încredere scăzută	Încredere scăzută	Încredere scăzută	Încredere foarte ridicată
P215	Dietetician / nutriționist	Încredere foarte ridicată	Încredere foarte ridicată	Neutru	Neutru	Neutru	Neutru	Încredere foarte ridicată
P216	Medicul de familie, Articole științifice / surse academice, Aplicații mobile dedicate sănătății, Rețele sociale (Facebook, Instagram, TikTok, YouTube), Reviste și ziare, Site-uri de internet generale, Familie și prieteni	Încredere ridicată	Neutru	Încredere scăzută	Încredere scăzută	Încredere scăzută	Încredere ridicată	Încredere scăzută
P217	Medicul de familie, Articole științifice / surse academice, Rețele sociale (Facebook, Instagram, TikTok, YouTube), Site-uri de internet generale, Familie și prieteni	Încredere ridicată	Încredere foarte ridicată	Încredere ridicată	Încredere ridicată	Încredere ridicată	Încredere ridicată	Încredere foarte ridicată
P218	Medicul specialist (oncolog, gastroenterolog, alt specialist), Dietetician / nutriționist, Rețele sociale (Facebook, Instagram, TikTok, YouTube)	Încredere ridicată	Încredere foarte ridicată	Neutru	Neutru	Încredere scăzută	Încredere foarte ridicată	Încredere foarte ridicată
P219	Aplicații mobile dedicate sănătății, Televiziune / radio, Rețele sociale (Facebook, Instagram, TikTok, YouTube), Site-uri de internet generale, Familie și prieteni	Încredere ridicată	Încredere ridicată	Neutru	Neutru	Neutru	Neutru	Neutru
P220	Medicul specialist (oncolog, gastroenterolog, alt specialist), Articole științifice / surse academice	Neutru	Neutru	Încredere scăzută	Încredere foarte scăzută	Încredere scăzută	Încredere scăzută	Încredere foarte ridicată
P221	Dietetician / nutriționist, Aplicații mobile dedicate sănătății, Televiziune / radio, Rețele sociale (Facebook, Instagram, TikTok, YouTube), Reviste și ziare, Site-uri de internet generale, Familie și prieteni	Neutru	Încredere ridicată	Neutru	Încredere scăzută	Încredere scăzută	Neutru	Încredere ridicată
P222	Articole științifice / surse academice, Aplicații mobile dedicate sănătății, Site-uri de internet generale, Familie și prieteni	Încredere ridicată	Încredere foarte ridicată	Neutru	Neutru	Neutru	Încredere ridicată	Încredere foarte ridicată
P223	Articole științifice / surse academice	Încredere ridicată	Încredere ridicată	Încredere scăzută	Încredere foarte scăzută	Neutru	Neutru	Încredere foarte ridicată
P224	Medicul specialist (oncolog, gastroenterolog, alt specialist), Dietetician / nutriționist, Articole științifice / surse academice, Televiziune / radio, Rețele sociale (Facebook, Instagram, TikTok, YouTube), Site-uri de internet generale, Familie și prieteni	Încredere ridicată	Încredere ridicată	Încredere ridicată	Încredere ridicată	Încredere ridicată	Încredere ridicată	Încredere ridicată
P225	Medicul specialist (oncolog, gastroenterolog, alt specialist), Articole științifice / surse academice, Aplicații mobile dedicate sănătății, Rețele sociale (Facebook, Instagram, TikTok, YouTube), Site-uri de internet generale	Încredere foarte ridicată	Încredere foarte ridicată	Neutru	Neutru	Neutru	Neutru	Încredere ridicată
P226	Medicul de familie, Medicul specialist (oncolog, gastroenterolog, alt specialist)	Încredere foarte ridicată	Încredere foarte ridicată	Încredere ridicată	Încredere ridicată	Încredere ridicată	Încredere ridicată	Încredere ridicată
P227	Aplicații mobile dedicate sănătății, Rețele sociale (Facebook, Instagram, TikTok, YouTube)	Încredere scăzută	Neutru	Încredere foarte scăzută	Încredere scăzută	Neutru	Neutru	Neutru
P228	Medicul specialist (oncolog, gastroenterolog, alt specialist)	Neutru	Neutru	Încredere scăzută	Încredere foarte scăzută	Încredere foarte scăzută	Încredere foarte scăzută	Încredere ridicată
P229	Dietetician / nutriționist, Reviste și ziare, Site-uri de internet generale, Familie și prieteni	Încredere ridicată	Încredere ridicată	Neutru	Neutru	Neutru	Încredere ridicată	Încredere ridicată
P230	Articole științifice / surse academice	Încredere ridicată	Încredere ridicată	Neutru	Încredere scăzută	Încredere scăzută	Încredere ridicată	Încredere foarte ridicată
P231	Dietetician / nutriționist, Articole științifice / surse academice, Rețele sociale (Facebook, Instagram, TikTok, YouTube)	Încredere foarte scăzută	Încredere ridicată	Neutru	Neutru	Încredere foarte scăzută	Încredere scăzută	Încredere ridicată

Participant_ID	F1	F2a	F2b	F2c	F2d	F2e	F2f	F2g
P232	Televiziune / radio, Rețele sociale (Facebook, Instagram, TikTok, YouTube), Reviste și ziare	Neutru	Încredere ridicată	Neutru	Neutru	Încredere ridicată	Neutru	Încredere foarte ridicată
P233	Rețele sociale (Facebook, Instagram, TikTok, YouTube)	Încredere foarte ridicată	Încredere foarte ridicată	Încredere ridicată	Încredere ridicată	Încredere ridicată	Încredere foarte ridicată	Încredere foarte ridicată
P234	Rețele sociale (Facebook, Instagram, TikTok, YouTube), Reviste și ziare, Site-uri de internet generale	Încredere scăzută	Încredere ridicată	Încredere ridicată	Încredere ridicată	Încredere ridicată	Neutru	Încredere foarte scăzută
P235	Rețele sociale (Facebook, Instagram, TikTok, YouTube), Familie și prieteni	Încredere foarte scăzută	Încredere scăzută	Încredere scăzută	Încredere scăzută	Neutru	Neutru	Neutru
P236	Dietetician / nutriționist, Articole științifice / surse academice, Aplicații mobile dedicate sănătății, Reviste și ziare, Site-uri de internet generale	Încredere ridicată	Încredere ridicată	Încredere scăzută	Încredere foarte scăzută	Încredere foarte scăzută	Neutru	Încredere ridicată
P237	Dietetician / nutriționist, Articole științifice / surse academice	Încredere foarte ridicată	Încredere foarte ridicată	Încredere scăzută	Încredere scăzută	Neutru	Încredere scăzută	Încredere foarte ridicată
P238	Articole științifice / surse academice, Site-uri de internet generale, Familie și prieteni	Încredere foarte ridicată	Încredere foarte ridicată	Încredere ridicată	Încredere scăzută	Încredere foarte scăzută	Încredere foarte ridicată	Încredere foarte ridicată
P239	Rețele sociale (Facebook, Instagram, TikTok, YouTube), Site-uri de internet generale, Familie și prieteni	Încredere ridicată	Încredere ridicată	Încredere scăzută	Neutru	Neutru	Neutru	Încredere ridicată
P240	Articole științifice / surse academice, Televiziune / radio, Rețele sociale (Facebook, Instagram, TikTok, YouTube), Site-uri de internet generale	Neutru	Încredere ridicată	Neutru	Neutru	Neutru	Neutru	Încredere ridicată
P241	Articole științifice / surse academice, Aplicații mobile dedicate sănătății, Reviste și ziare	Neutru	Neutru	Încredere ridicată	Neutru	Neutru	Neutru	Încredere ridicată
P242	Medicul specialist (oncolog, gastroenterolog, alt specialist), Aplicații mobile dedicate sănătății, Familie și prieteni	Încredere scăzută	Neutru	Încredere foarte scăzută	Încredere foarte scăzută	Încredere foarte scăzută	Încredere scăzută	Neutru
P243	Aplicații mobile dedicate sănătății, Televiziune / radio, Rețele sociale (Facebook, Instagram, TikTok, YouTube), Familie și prieteni	Încredere foarte scăzută	Încredere foarte ridicată	Încredere ridicată	Încredere ridicată	Încredere ridicată	Încredere foarte ridicată	Încredere ridicată
P244	Medicul de familie, Farmacist, Articole științifice / surse academice, Aplicații mobile dedicate sănătății	Încredere ridicată	Încredere ridicată	Neutru	Neutru	Neutru	Neutru	Încredere ridicată
P245	Medicul de familie	Neutru	Încredere foarte scăzută	Neutru	Neutru	Neutru	Neutru	Neutru
P246	Medicul de familie, Articole științifice / surse academice, Aplicații mobile dedicate sănătății, Televiziune / radio, Rețele sociale (Facebook, Instagram, TikTok, YouTube), Reviste și ziare, Site-uri de internet generale, Familie și prieteni	Încredere foarte ridicată	Încredere ridicată	Încredere scăzută	Încredere scăzută	Încredere scăzută	Încredere scăzută	Încredere ridicată
P247	Dietetician / nutriționist, Aplicații mobile dedicate sănătății, Televiziune / radio, Reviste și ziare, Site-uri de internet generale	Neutru	Încredere ridicată	Încredere scăzută	Încredere scăzută	Neutru	Neutru	Încredere ridicată
P248	Articole științifice / surse academice	Încredere foarte ridicată	Încredere foarte ridicată	Neutru	Neutru	Neutru	Neutru	Încredere foarte ridicată
P249	Aplicații mobile dedicate sănătății, Rețele sociale (Facebook, Instagram, TikTok, YouTube)	Încredere ridicată	Încredere foarte ridicată	Încredere scăzută	Încredere ridicată	Încredere foarte scăzută	Încredere ridicată	Încredere ridicată
P250	Aplicații mobile dedicate sănătății, Rețele sociale (Facebook, Instagram, TikTok, YouTube), Familie și prieteni	Încredere scăzută	Încredere foarte ridicată	Încredere ridicată	Neutru	Încredere ridicată	Încredere ridicată	Încredere foarte ridicată
P251	Dietetician / nutriționist, Aplicații mobile dedicate sănătății	Încredere ridicată	Încredere foarte ridicată	Neutru	Neutru	Neutru	Încredere ridicată	Încredere foarte ridicată
P252	Articole științifice / surse academice, Aplicații mobile dedicate sănătății, Rețele sociale (Facebook, Instagram, TikTok, YouTube), Familie și prieteni	Încredere scăzută	Încredere ridicată	Încredere ridicată	Neutru	Încredere foarte scăzută	Neutru	Neutru
P253	Articole științifice / surse academice	Încredere ridicată	Încredere scăzută	Încredere scăzută	Încredere foarte scăzută	Încredere foarte scăzută	Neutru	Încredere foarte ridicată
P254	Aplicații mobile dedicate sănătății, Televiziune / radio, Rețele sociale (Facebook, Instagram, TikTok, YouTube), Reviste și ziare, Familie și prieteni	Încredere foarte scăzută	Încredere ridicată	Neutru	Neutru	Neutru	Neutru	Încredere ridicată
P255	Rețele sociale (Facebook, Instagram, TikTok, YouTube), Site-uri de internet generale, Familie și prieteni	Încredere ridicată	Încredere ridicată	Încredere scăzută	Încredere scăzută	Încredere scăzută	Încredere scăzută	Încredere ridicată
P256	Medicul specialist (oncolog, gastroenterolog, alt specialist), Articole științifice / surse academice, Reviste și ziare, Site-uri de internet generale	Încredere foarte ridicată	Încredere foarte ridicată	Neutru	Neutru	Încredere ridicată	Încredere foarte ridicată	Încredere foarte ridicată
P257	Dietetician / nutriționist, Articole științifice / surse academice	Încredere ridicată	Încredere ridicată	Neutru	Neutru	Neutru	Neutru	Încredere ridicată
P258	Medicul de familie	Încredere scăzută	Încredere scăzută	Încredere foarte scăzută	Încredere scăzută	Încredere foarte scăzută	Încredere scăzută	Încredere foarte scăzută
P259	Aplicații mobile dedicate sănătății, Rețele sociale (Facebook, Instagram, TikTok, YouTube)	Încredere ridicată	Încredere ridicată	Neutru	Neutru	Încredere scăzută	Încredere ridicată	Încredere ridicată
P260	Televiziune / radio, Rețele sociale (Facebook, Instagram, TikTok, YouTube), Familie și prieteni	Încredere ridicată	Încredere foarte ridicată	Încredere ridicată	Încredere ridicată	Încredere ridicată	Încredere ridicată	Încredere foarte ridicată
P261	Aplicații mobile dedicate sănătății	Neutru	Încredere ridicată	Neutru	Încredere scăzută	Încredere scăzută	Neutru	Încredere ridicată
P262	Aplicații mobile dedicate sănătății, Rețele sociale (Facebook, Instagram, TikTok, YouTube), Site-uri de internet generale	Încredere ridicată	Neutru	Încredere ridicată	Încredere ridicată	Neutru	Încredere ridicată	Neutru
P263	Dietetician / nutriționist, Familie și prieteni	Încredere ridicată	Încredere ridicată	Încredere scăzută	Neutru	Neutru	Încredere ridicată	Neutru
P264	Medicul specialist (oncolog, gastroenterolog, alt specialist), Dietetician / nutriționist, Aplicații mobile dedicate sănătății, Reviste și ziare, Site-uri de internet generale, Familie și prieteni	Încredere scăzută	Încredere foarte ridicată	Încredere ridicată	Încredere ridicată	Încredere scăzută	Încredere ridicată	Încredere foarte ridicată

Participant_ ID	F1	F2a	F2b	F2c	F2d	F2e	F2f	F2g
P265	Dietetician / nutriționist, Aplicații mobile dedicate sănătății, Televiziune / radio, Rețele sociale (Facebook, Instagram, TikTok, YouTube)	Neutru	Încredere ridicată	Încredere scăzută	Încredere ridicată	Încredere ridicată	Neutru	Neutru
P266	Medicul de familie, Medicul specialist (oncolog, gastroenterolog, alt specialist)	Încredere ridicată	Încredere ridicată	Încredere scăzută	Încredere foarte scăzută	Încredere foarte scăzută	Încredere foarte scăzută	Încredere foarte ridicată
P267	Medicul de familie, Televiziune / radio, Site-uri de internet generale	Încredere foarte ridicată	Încredere ridicată	Încredere scăzută	Încredere scăzută	Încredere ridicată	Neutru	Încredere foarte ridicată
P268	Rețele sociale (Facebook, Instagram, TikTok, YouTube), Site-uri de internet generale	Neutru	Neutru	Neutru	Neutru	Încredere scăzută	Neutru	Încredere foarte ridicată
P269	Medicul de familie, Medicul specialist (oncolog, gastroenterolog, alt specialist), Dietetician / nutriționist, Articole științifice / surse academice, Aplicații mobile dedicate sănătății, Televiziune / radio, Rețele sociale (Facebook, Instagram, TikTok, YouTube), Site-uri de internet generale, Familie și prieteni	Încredere ridicată	Încredere foarte ridicată	Neutru	Neutru	Încredere ridicată	Încredere scăzută	Încredere foarte ridicată
P270	Articole științifice / surse academice, Aplicații mobile dedicate sănătății, Site-uri de internet generale	Încredere ridicată	Încredere ridicată	Neutru	Încredere foarte scăzută	Încredere foarte scăzută	Încredere scăzută	Încredere foarte ridicată
P271	Medicul de familie, Farmacist, Televiziune / radio, Rețele sociale (Facebook, Instagram, TikTok, YouTube), Familie și prieteni	Încredere ridicată	Încredere ridicată	Neutru	Neutru	Neutru	Neutru	Neutru
P272	Aplicații mobile dedicate sănătății	Încredere ridicată	Neutru	Neutru	Neutru	Neutru	Neutru	Încredere ridicată
P273	Medicul de familie, Medicul specialist (oncolog, gastroenterolog, alt specialist), Dietetician / nutriționist, Articole științifice / surse academice	Încredere scăzută	Încredere ridicată	Încredere foarte scăzută	Încredere foarte scăzută	Încredere foarte scăzută	Încredere scăzută	Încredere foarte scăzută
P274	Medicul de familie, Televiziune / radio, Site-uri de internet generale, Familie și prieteni	Încredere ridicată	Neutru	Neutru	Neutru	Încredere ridicată	Încredere ridicată	Neutru
P275	Aplicații mobile dedicate sănătății, Televiziune / radio, Site-uri de internet generale	Încredere ridicată	Încredere foarte ridicată	Încredere foarte scăzută	Încredere foarte scăzută	Încredere scăzută	Încredere scăzută	Încredere ridicată
P276	Familie și prieteni	Încredere scăzută	Neutru	Neutru	Neutru	Neutru	Încredere ridicată	Neutru
P277	Articole științifice / surse academice, Rețele sociale (Facebook, Instagram, TikTok, YouTube), Reviste și ziare, Site-uri de internet generale	Încredere scăzută	Încredere ridicată	Neutru	Neutru	Neutru	Încredere ridicată	Încredere foarte ridicată
P278	Articole științifice / surse academice	Încredere ridicată	Încredere ridicată	Încredere foarte scăzută	Încredere foarte scăzută	Încredere foarte scăzută	Încredere scăzută	Încredere foarte ridicată
P279	Articole științifice / surse academice	Neutru	Neutru	Neutru	Neutru	Neutru	Neutru	Încredere ridicată
P280	Articole științifice / surse academice	Încredere ridicată	Neutru	Încredere foarte scăzută	Încredere foarte scăzută	Încredere scăzută	Neutru	Încredere foarte scăzută
P281	Farmacist, Dietetician / nutriționist	Încredere foarte ridicată	Încredere foarte ridicată	Încredere foarte scăzută	Încredere foarte scăzută	Încredere foarte scăzută	Încredere scăzută	Încredere foarte ridicată
P282	Rețele sociale (Facebook, Instagram, TikTok, YouTube), Site-uri de internet generale	Neutru	Neutru	Neutru	Neutru	Neutru	Neutru	Încredere ridicată
P283	Medicul de familie, Articole științifice / surse academice, Aplicații mobile dedicate sănătății, Televiziune / radio, Reviste și ziare, Site-uri de internet generale	Încredere foarte ridicată	Încredere foarte ridicată	Încredere ridicată	Încredere foarte scăzută	Încredere foarte ridicată	Neutru	Încredere foarte ridicată
P284	Medicul de familie, Televiziune / radio, Rețele sociale (Facebook, Instagram, TikTok, YouTube), Reviste și ziare, Site-uri de internet generale, Familie și prieteni	Încredere ridicată	Încredere ridicată	Încredere ridicată	Încredere ridicată	Încredere ridicată	Încredere ridicată	Încredere ridicată
P285	Medicul de familie, Medicul specialist (oncolog, gastroenterolog, alt specialist), Aplicații mobile dedicate sănătății, Televiziune / radio, Rețele sociale (Facebook, Instagram, TikTok, YouTube), Reviste și ziare, Site-uri de internet generale, Familie și prieteni	Încredere ridicată	Încredere ridicată	Încredere ridicată	Încredere scăzută	Încredere ridicată	Încredere ridicată	Neutru
P286	Medicul de familie, Farmacist	Încredere ridicată	Încredere ridicată	Neutru	Neutru	Încredere foarte scăzută	Încredere ridicată	Încredere foarte ridicată
P287	Medicul de familie, Medicul specialist (oncolog, gastroenterolog, alt specialist), Articole științifice / surse academice, Rețele sociale (Facebook, Instagram, TikTok, YouTube), Site-uri de internet generale	Încredere foarte ridicată	Încredere foarte ridicată	Neutru	Neutru	Neutru	Neutru	Încredere foarte ridicată
P288	Televiziune / radio, Familie și prieteni	Neutru	Neutru	Încredere scăzută	Încredere foarte scăzută	Neutru	Încredere ridicată	Încredere foarte ridicată
P289	Televiziune / radio	Încredere ridicată	Neutru	Neutru	Neutru	Încredere ridicată	Neutru	Încredere foarte ridicată
P290	Medicul de familie, Medicul specialist (oncolog, gastroenterolog, alt specialist), Farmacist, Dietetician / nutriționist, Articole științifice / surse academice	Încredere ridicată	Încredere ridicată	Încredere scăzută	Încredere scăzută	Încredere foarte scăzută	Încredere scăzută	Încredere foarte ridicată
P291	Articole științifice / surse academice	Neutru	Neutru	Neutru	Neutru	Neutru	Neutru	Încredere foarte ridicată
P292	Medicul de familie, Dietetician / nutriționist, Aplicații mobile dedicate sănătății	Încredere foarte ridicată	Încredere foarte ridicată	Încredere scăzută	Încredere foarte scăzută	Neutru	Neutru	Încredere foarte ridicată
P293	Medicul de familie, Rețele sociale (Facebook, Instagram, TikTok, YouTube), Familie și prieteni	Încredere ridicată	Neutru	Neutru	Încredere scăzută	Încredere scăzută	Neutru	Neutru
P294	Medicul de familie, Medicul specialist (oncolog, gastroenterolog, alt specialist), Farmacist, Reviste și ziare, Site-uri de internet generale	Încredere foarte ridicată	Încredere ridicată	Neutru	Neutru	Încredere ridicată	Încredere ridicată	Încredere foarte ridicată
P295	Medicul de familie, Aplicații mobile dedicate sănătății, Rețele sociale (Facebook, Instagram, TikTok, YouTube), Familie și prieteni	Încredere ridicată	Neutru	Încredere scăzută	Neutru	Neutru	Încredere ridicată	Încredere ridicată
P296	Medicul de familie, Televiziune / radio	Încredere ridicată	Neutru	Neutru	Neutru	Încredere ridicată	Neutru	Neutru
P297	Dietetician / nutriționist	Neutru	Încredere scăzută	Neutru	Încredere scăzută	Încredere foarte scăzută	Încredere scăzută	Încredere scăzută

Participant_ID	F1	F2a	F2b	F2c	F2d	F2e	F2f	F2g
P298	Medicul de familie, Medicul specialist (oncolog, gastroenterolog, alt specialist), Dietetician / nutriționist, Televiziune / radio, Reviste și ziare, Familie și prieteni	Încredere ridicată	Încredere ridicată	Încredere foarte scăzută	Încredere foarte scăzută	Neutru	Încredere ridicată	Încredere foarte ridicată
P299	Medicul de familie, Farmacist, Televiziune / radio	Încredere scăzută	Încredere ridicată	Încredere foarte scăzută	Încredere foarte scăzută	Încredere scăzută	Încredere foarte scăzută	Încredere foarte scăzută
P300	Medicul de familie, Medicul specialist (oncolog, gastroenterolog, alt specialist), Site-uri de internet generale, Familie și prieteni	Neutru	Neutru	Încredere ridicată	Încredere foarte ridicată	Încredere ridicată	Neutru	Încredere foarte scăzută
P301	Articole științifice / surse academice, Rețele sociale (Facebook, Instagram, TikTok, YouTube), Site-uri de internet generale	Încredere scăzută	Încredere scăzută	Neutru	Încredere ridicată	Încredere foarte ridicată	Încredere ridicată	Încredere ridicată

Panel G — Awareness of cancer prevention policies and prior counselling

Participant_ ID	G1	G2	G3	G4
P001	Nu	Nu	Nu	Nu
P002	Nu	Nu	Nu	Nu
P003	Da	Nu	Nu	Nu
P004	Da	Da	Nu	Da
P005	Da	Da	Da	Nu
P006	Nu	Nu	Da	Nu
P007	Nu	Nu	Nu	Nu
P008	Nu	Nu	Nu	Nu
P009	Da	Da	Nu	Nu
P010	Nu	Nu	Nu	Da
P011	Nu	Nu	Nu	Nu
P012	Nu	Nu	Nu	Nu
P013	Nu	Nu	Nu	Nu
P014	Da	Nu	Da	Da
P015	Nu	Nu	Nu	Nu
P016	Nu	Nu	Nu	Nu
P017	Da	Da	Nu	Nu
P018	Nu	Nu	Nu	Nu
P019	Nu	Nu	Nu	Nu
P020	Nu	Nu	Nu	Da
P021	Da	Nu	Nu	Nu
P022	Nu	Nu	Nu	Nu
P023	Da	Nu	Nu	Nu
P024	Nu	Da	Nu	Nu
P025	Nu	Nu	Nu	Nu
P026	Nu	Da	Nu	Nu
P027	Da	Da	Da	Nu
P028	Nu	Nu	Nu	Nu
P029	Da	Da	Nu	Nu
P030	Nu	Nu	Nu	Nu
P031	Nu	Nu	Nu	Nu
P032	Da	Da	Da	Da
P033	Nu	Nu	Nu	Nu
P034	Nu	Nu	Nu	Nu
P035	Nu	Nu	Nu	Nu
P036	Nu	Nu	Nu	Nu
P037	Nu	Da	Nu	Nu
P038	Nu	Nu	Nu	Nu
P039	Da	Da	Nu	Nu
P040	Nu	Nu	Nu	Nu
P041	Da	Da	Da	Nu
P042	Nu	Nu	Nu	Nu
P043	Nu	Nu	Nu	Nu
P044	Nu	Nu	Nu	Nu
P045	Nu	Nu	Nu	Nu
P046	Nu	Nu	Nu	Nu
P047	Nu	Nu	Nu	Nu
P048	Nu	Nu	Nu	Nu
P049	Nu	Da	Nu	Nu
P050	Nu	Nu	Nu	Nu
P051	Nu	Nu	Da	Nu
P052	Nu	Da	Nu	Da
P053	Da	Nu	Nu	Da
P054	Da	Da	Da	Da
P055	Nu	Nu	Nu	Nu
P056	Nu	Da	Nu	Nu
P057	Nu	Nu	Nu	Nu
P058	Nu	Da	Nu	Nu
P059	Nu	Nu	Nu	Nu
P060	Da	Da	Nu	Nu
P061	Nu	Nu	Nu	Da
P062	Nu	Nu	Nu	Nu
P063	Da	Nu	Nu	Nu
P064	Nu	Nu	Nu	Nu
P065	Da	Da	Da	Da
P066	Nu	Da	Nu	Nu
P067	Nu	Nu	Nu	Nu
P068	Nu	Nu	Nu	Nu
P069	Da	Da	Nu	Da
P070	Da	Da	Da	Da
P071	Da	Da	Nu	Da
P072	Nu	Nu	Nu	Nu
P073	Nu	Da	Nu	Da
P074	Da	Nu	Nu	Nu
P075	Nu	Da	Nu	Nu
P076	Nu	Nu	Nu	Nu
P077	Da	Da	Nu	Nu
P078	Da	Nu	Nu	Da
P079	Nu	Da	Nu	Nu
P080	Nu	Da	Nu	Da

Participant_ ID	G1	G2	G3	G4
P081	Da	Da	Da	Nu
P082	Nu	Nu	Nu	Nu
P083	Da	Da	Da	Da
P084	Nu	Nu	Nu	Nu
P085	Nu	Da	Da	Nu
P086	Nu	Nu	Nu	Nu
P087	Nu	Nu	Da	Da
P088	Nu	Nu	Nu	Nu
P089	Nu	Nu	Nu	Nu
P090	Da	Da	Da	Nu
P091	Nu	Nu	Nu	Nu
P092	Nu	Nu	Nu	Da
P093	Da	Da	Nu	Nu
P094	Nu	Nu	Nu	Nu
P095	Nu	Nu	Nu	Nu
P096	Nu	Nu	Nu	Da
P097	Nu	Nu	Nu	Da
P098	Nu	Nu	Nu	Nu
P099	Nu	Nu	Da	Da
P100	Nu	Da	Nu	Nu
P101	Da	Da	Da	Da
P102	Nu	Da	Nu	Nu
P103	Nu	Nu	Nu	Nu
P104	Nu	Nu	Nu	Nu
P105	Nu	Nu	Nu	Nu
P106	Da	Nu	Nu	Nu
P107	Da	Da	Da	Da
P108	Nu	Da	Nu	Nu
P109	Nu	Nu	Nu	Nu
P110	Nu	Da	Nu	Nu
P111	Nu	Nu	Nu	Nu
P112	Da	Nu	Nu	Nu
P113	Nu	Da	Nu	Da
P114	Nu	Nu	Nu	Nu
P115	Nu	Nu	Nu	Nu
P116	Nu	Nu	Nu	Nu
P117	Da	Da	Nu	Nu
P118	Da	Da	Da	Nu
P119	Nu	Nu	Nu	Da
P120	Nu	Da	Nu	Da
P121	Nu	Nu	Nu	Da
P122	Nu	Nu	Nu	Nu
P123	Nu	Da	Nu	Nu
P124	Nu	Nu	Nu	Nu
P125	Nu	Nu	Nu	Nu
P126	Nu	Da	Nu	Nu
P127	Nu	Da	Nu	Nu
P128	Da	Da	Da	Da
P129	Nu	Nu	Nu	Da
P130	Nu	Nu	Nu	Nu
P131	Nu	Nu	Nu	Da
P132	Nu	Da	Nu	Nu
P133	Da	Da	Da	Da
P134	Nu	Nu	Nu	Nu
P135	Da	Da	Da	Nu
P136	Da	Nu	Da	Da
P137	Nu	Nu	Nu	Nu
P138	Nu	Nu	Nu	Nu
P139	Nu	Nu	Nu	Nu
P140	Nu	Nu	Nu	Nu
P141	Nu	Da	Nu	Nu
P142	Nu	Da	Nu	Nu
P143	Nu	Da	Da	Da
P144	Nu	Nu	Nu	Da
P145	Da	Da	Da	Nu
P146	Da	Da	Da	Da
P147	Nu	Nu	Nu	Nu
P148	Nu	Da	Nu	Nu
P149	Nu	Nu	Nu	Nu
P150	Nu	Da	Nu	Nu
P151	Nu	Da	Nu	Nu
P152	Nu	Nu	Nu	Nu
P153	Nu	Nu	Nu	Da
P154	Nu	Da	Nu	Nu
P155	Nu	Da	Nu	Nu
P156	Da	Nu	Nu	Nu
P157	Nu	Da	Nu	Nu
P158	Nu	Nu	Nu	Nu
P159	Nu	Da	Nu	Nu
P160	Nu	Nu	Nu	Nu
P161	Nu	Da	Nu	Nu
P162	Nu	Nu	Nu	Nu

Participant_ID	G1	G2	G3	G4
P163	Da	Da	Nu	Nu
P164	Da	Da	Da	Da
P165	Nu	Da	Da	Da
P166	Nu	Nu	Nu	Nu
P167	Nu	Da	Nu	Da
P168	Nu	Da	Nu	Nu
P169	Da	Da	Nu	Nu
P170	Nu	Nu	Nu	Nu
P171	Da	Nu	Nu	Nu
P172	Nu	Nu	Nu	Nu
P173	Da	Nu	Nu	Nu
P174	Nu	Nu	Nu	Nu
P175	Nu	Nu	Nu	Nu
P176	Nu	Da	Nu	Da
P177	Nu	Nu	Nu	Nu
P178	Da	Da	Da	Da
P179	Nu	Nu	Nu	Nu
P180	Nu	Nu	Nu	Da
P181	Nu	Nu	Nu	Nu
P182	Nu	Nu	Nu	Da
P183	Nu	Nu	Nu	Nu
P184	Nu	Da	Nu	Da
P185	Da	Da	Da	Da
P186	Nu	Da	Nu	Da
P187	Nu	Nu	Nu	Nu
P188	Nu	Nu	Nu	Nu
P189	Nu	Da	Nu	Nu
P190	Da	Da	Nu	Da
P191	Nu	Nu	Nu	Nu
P192	Nu	Nu	Nu	Nu
P193	Nu	Da	Nu	Da
P194	Nu	Da	Nu	Nu
P195	Nu	Nu	Nu	Nu
P196	Nu	Nu	Nu	Nu
P197	Da	Nu	Da	Da
P198	Nu	Da	Nu	Nu
P199	Nu	Da	Nu	Da
P200	Nu	Nu	Nu	Nu
P201	Nu	Da	Nu	Nu
P202	Nu	Nu	Nu	Nu
P203	Da	Da	Da	Da
P204	Nu	Da	Nu	Nu
P205	Nu	Da	Nu	Nu
P206	Da	Nu	Nu	Nu
P207	Nu	Nu	Nu	Nu
P208	Da	Da	Da	Nu
P209	Nu	Nu	Nu	Nu
P210	Da	Nu	Nu	Nu
P211	Nu	Da	Nu	Nu
P212	Nu	Da	Nu	Nu
P213	Nu	Nu	Nu	Nu
P214	Da	Da	Nu	Da
P215	Nu	Da	Nu	Da
P216	Nu	Nu	Nu	Nu
P217	Nu	Nu	Nu	Nu
P218	Nu	Da	Nu	Da
P219	Nu	Nu	Nu	Nu
P220	Nu	Nu	Da	Nu
P221	Nu	Nu	Nu	Da
P222	Nu	Nu	Nu	Nu
P223	Nu	Nu	Nu	Nu
P224	Nu	Nu	Nu	Da
P225	Da	Nu	Nu	Nu
P226	Da	Nu	Nu	Da
P227	Nu	Nu	Nu	Nu
P228	Da	Nu	Nu	Da
P229	Nu	Nu	Nu	Da
P230	Nu	Da	Da	Da
P231	Nu	Da	Nu	Nu
P232	Nu	Nu	Nu	Nu
P233	Nu	Nu	Nu	Da
P234	Nu	Nu	Nu	Da
P235	Nu	Nu	Nu	Da
P236	Nu	Da	Nu	Nu
P237	Da	Da	Nu	Nu
P238	Nu	Da	Nu	Nu
P239	Nu	Nu	Nu	Nu
P240	Nu	Nu	Nu	Nu
P241	Nu	Da	Nu	Nu
P242	Nu	Nu	Nu	Nu
P243	Nu	Nu	Nu	Nu
P244	Da	Da	Nu	Da

Participant_ ID	G1	G2	G3	G4
P245	Da	Da	Da	Da
P246	Da	Da	Nu	Nu
P247	Da	Da	Nu	Nu
P248	Nu	Nu	Nu	Nu
P249	Nu	Nu	Nu	Nu
P250	Nu	Nu	Nu	Nu
P251	Da	Da	Da	Nu
P252	Nu	Nu	Nu	Nu
P253	Da	Da	Nu	Da
P254	Nu	Nu	Nu	Nu
P255	Da	Da	Nu	Da
P256	Da	Da	Nu	Da
P257	Nu	Nu	Nu	Da
P258	Da	Nu	Nu	Nu
P259	Nu	Nu	Nu	Nu
P260	Da	Da	Nu	Da
P261	Nu	Nu	Nu	Da
P262	Nu	Da	Nu	Nu
P263	Da	Nu	Nu	Nu
P264	Nu	Nu	Nu	Nu
P265	Nu	Nu	Nu	Da
P266	Nu	Nu	Nu	Nu
P267	Nu	Nu	Nu	Nu
P268	Nu	Nu	Nu	Nu
P269	Nu	Da	Nu	Da
P270	Da	Da	Da	Nu
P271	Nu	Nu	Nu	Nu
P272	Nu	Nu	Nu	Nu
P273	Da	Nu	Da	Da
P274	Nu	Da	Nu	Nu
P275	Nu	Nu	Nu	Da
P276	Nu	Nu	Nu	Nu
P277	Nu	Da	Nu	Nu
P278	Nu	Da	Da	Da
P279	Nu	Nu	Nu	Nu
P280	Nu	Da	Nu	Nu
P281	Da	Da	Da	Da
P282	Nu	Nu	Nu	Nu
P283	Nu	Da	Da	Da
P284	Nu	Nu	Nu	Nu
P285	Nu	Nu	Nu	Nu
P286	Da	Nu	Nu	Da
P287	Nu	Da	Nu	Da
P288	Nu	Nu	Nu	Nu
P289	Nu	Nu	Nu	Da
P290	Da	Da	Da	Da
P291	Nu	Nu	Da	Da
P292	Da	Da	Nu	Da
P293	Nu	Nu	Nu	Da
P294	Nu	Nu	Nu	Da
P295	Da	Da	Nu	Da
P296	Nu	Nu	Nu	Da
P297	Nu	Nu	Nu	Nu
P298	Nu	Nu	Nu	Nu
P299	Nu	Nu	Nu	Nu
P300	Da	Nu	Da	Da
P301	Nu	Nu	Nu	Nu
