# Supplementary material for: The EduNutriCRC Questionnaire: A Pilot Knowledge–Attitudes–Practices Study on Nutritional Prevention of Colorectal Cancer in Romanian Adults
Source: Nutrients. 2026 Jul 13;18(14):2293. doi: 10.3390/nu18142293 (PMC13414599; doi:10.3390/nu18142293)
Supplement: Supplementary file 1 [file nutrients-18-02293-s001.zip › Table_S2.pdf]

EduNutriCRC QUESTIONNAIRE

SECTION A. Sociodemographic and anthropometric data

2. A1. What is your age? *

In years, no decimals

____

3. A2. What is your height? *

In cm, no decimals

____

4. A3. What is your weight? *

In kg, no decimals

____

5. A4. Sex: *

Mark only one oval.

- ☐ Male
- ☐ Female
- ☐ Prefer not to say

6. A5. Place of residence: *

Mark only one oval.

- ☐ Urban
- ☐ Rural

7. A6. County of residence: *

Dropdown — Mark only one oval.

- ☐ Alba
- ☐ Arad
- ☐ Argeș
- ☐ Bacău
- ☐ Bihor
- ☐ Bistrița-Năsăud
- ☐ Botoșani
- ☐ Brașov
- ☐ Brăila
- ☐ Buzău
- ☐ Caraș-Severin
- ☐ Călărași
- ☐ Cluj

- ☐ Constanța
- ☐ Covasna
- ☐ Dâmbovița
- ☐ Dolj
- ☐ Galați
- ☐ Giurgiu
- ☐ Gorj
- ☐ Harghita
- ☐ Hunedoara
- ☐ Ialomița
- ☐ Iași
- ☐ Ilfov
- ☐ Maramureș
- ☐ Mehedinți
- ☐ Mureș
- ☐ Neamț
- ☐ Olt
- ☐ Prahova
- ☐ Sălaj
- ☐ Satu Mare
- ☐ Sibiu
- ☐ Suceava
- ☐ Teleorman
- ☐ Timiș
- ☐ Tulcea
- ☐ Vaslui
- ☐ Vâlcea
- ☐ Vrancea
- ☐ Bucharest Municipality

8. A7. Highest level of education completed: *

Mark only one oval.

- ☐ Secondary / high school / vocational education
- ☐ Post-secondary education
- ☐ University education (bachelor's)
- ☐ Postgraduate studies (master's / doctorate)

9. A8. Current occupational status: *

Mark only one oval.

- ☐ Employed in the public sector
- ☐ Employed in the private sector

- ☐ Self-employed / entrepreneur
- ☐ Retired
- ☐ Student
- ☐ Homemaker
- ☐ Unemployed / looking for work

10. A9. Net monthly household income per person (approximate): *

Mark only one oval.

- ☐ Below 3,000 RON
- ☐ 3,000–5,000 RON
- ☐ 5,001–8,000 RON
- ☐ 8,001–12,000 RON
- ☐ Above 12,000 RON
- ☐ Prefer not to say

11. A10. Have you had cases of colorectal cancer in your family (parents, siblings, children)? *

Mark only one oval.

- ☐ Yes
- ☐ No

SECTION B. General knowledge about colorectal cancer

12. B1. What position do you think colorectal cancer holds in terms of incidence (new cases per year) in the ranking of cancer types in Romania?

Mark only one oval.

- ☐ 1st place
- ☐ 2nd place
- ☐ 3rd place
- ☐ 4th place
- ☐ Between 5th–10th place
- ☐ Between 11th–15th place
- ☐ Don't know

13. B2. At what age is colorectal cancer screening generally recommended to begin?

Mark only one oval.

- ☐ 30 years
- ☐ 40 years
- ☐ 50 years
- ☐ 65 years
- ☐ Don't know

14. B3. Which of the following can be warning signs for colorectal cancer? *

Multiple response — check all variants you consider relevant.

- ☐ Rectal bleeding / blood in stool
- ☐ Sudden and unexplained weight loss
- ☐ Persistent nausea / vomiting
- ☐ Frequent heartburn
- ☐ Dysphagia (difficulty swallowing)
- ☐ Persistent changes in bowel habits
- ☐ Excessive thirst
- ☐ Hair loss
- ☐ Joint pain
- ☐ Don't know

15. B4. Have you heard of the National Screening Programme for colorectal cancer? *

Mark only one oval.

- ☐ Yes, I participated
- ☐ It was recommended that I participate
- ☐ Yes, but I encountered difficulties accessing it
- ☐ No

16. B5. Have you ever undergone a screening test for colorectal cancer (fecal occult blood test / FIT or colonoscopy)?

Mark only one oval.

- ☐ Yes, FIT
- ☐ Yes, colonoscopy
- ☐ Yes, both
- ☐ No

SECTION C. Specific nutritional knowledge regarding CRC

For each statement, indicate whether you consider it true, false, or you don't know.

17. Statements about nutrition and colorectal cancer *

Mark only one oval per row.

Statement	True	False	Don't know
C1. Regular consumption of processed meat (cold cuts, sausages, ham) increases the risk of colorectal cancer.	☐	☐	☐
C2. Excessive consumption of red meat (beef, pork, lamb) increases the risk of colorectal cancer.	☐	☐	☐
C3. Regular consumption of dietary fiber (whole grains, legumes) reduces the risk of colorectal cancer.	☐	☐	☐
C4. Alcohol consumption, even moderate, increases the risk of colorectal cancer.	☐	☐	☐

Statement	True	False	Don't know
C5. Smoking (including alternative tobacco products: e-cigarettes, heated tobacco devices) increases the risk of colorectal cancer.	☐	☐	☐
C6. Obesity is a risk factor for colorectal cancer.	☐	☐	☐
C7. Daily consumption of fruits and vegetables (at least 400 g) may reduce the risk of colorectal cancer.	☐	☐	☐
C8. Regular physical activity reduces the risk of colorectal cancer.	☐	☐	☐
C9. Moderate consumption of dairy products (especially milk and yogurt) is associated with a lower risk of colorectal cancer.	☐	☐	☐
C10. Carbonated water increases the risk of cancer.	☐	☐	☐

SECTION D. Attitudes and beliefs

Indicate your degree of agreement with each statement.

18. Attitudes towards nutrition and cancer prevention *

Mark only one oval per row.

Statement	Strongly disagree	Partially disagree	Neutral	Partially agree	Strongly agree
D1. I believe my current diet is healthy.	☐	☐	☐	☐	☐
D2. I believe that by changing my diet, I can significantly reduce my risk of getting cancer.	☐	☐	☐	☐	☐
D3. I am willing to make changes in my diet to reduce my oncological risk.	☐	☐	☐	☐	☐
D4. I feel capable of cooking healthy meals on a regular basis.	☐	☐	☐	☐	☐
D5. Information about nutrition is contradictory and difficult to apply.	☐	☐	☐	☐	☐
D6. The cost of a healthy diet represents a real barrier for me.	☐	☐	☐	☐	☐
D7. The time required to follow a healthy diet represents a real barrier for me.	☐	☐	☐	☐	☐

SECTION E. Food consumption frequency and tobacco use

How often do you consume the following foods / food groups? Check only one option per row.

19. Food consumption frequency *

Mark only one oval per row.

Food / Food group	Daily	Several times a week	Several times a month	Rarely (less than once a month)	Never / almost never
E1. Processed meat (cold cuts, sausages, salami, ham)	☐	☐	☐	☐	☐
E2. Red meat (beef, pork, lamb)	☐	☐	☐	☐	☐
E3. White meat (chicken, turkey)	☐	☐	☐	☐	☐
E4. Fish	☐	☐	☐	☐	☐
E5. Whole grains (whole grain bread, whole grain pasta, oats, brown rice, muesli)	☐	☐	☐	☐	☐
E6. Legumes (beans, lentils, chickpeas, peas)	☐	☐	☐	☐	☐
E7. Fresh fruit	☐	☐	☐	☐	☐
E8. Vegetables (excluding potatoes)	☐	☐	☐	☐	☐
E9. Ultra-processed foods / with added sugar (packaged snacks, sweets, cakes, pastries, fast food)	☐	☐	☐	☐	☐
E10. Sugar-sweetened beverages (juices, energy drinks)	☐	☐	☐	☐	☐
E11. Alcoholic beverages	☐	☐	☐	☐	☐
E12. Water (at least 1.5 liters / day)	☐	☐	☐	☐	☐

20. E13. How many cigarettes do you smoke, on average, per day? *

Include the equivalent of alternative tobacco products — e-cigarettes, heated tobacco devices.

Mark only one oval.

- ☐ I don’t smoke at all
- ☐ Less than one cigarette/day (occasional)
- ☐ 1–10 cigarettes/day
- ☐ 11–20 cigarettes/day
- ☐ Over 20 cigarettes/day

SECTION F. Sources of nutritional information

21. F1. What are your main sources of information about nutrition and health? *

You may check multiple options.

- ☐ Family doctor

- ☐ Specialist physician (oncologist, gastroenterologist, other specialist)
- ☐ Pharmacist
- ☐ Dietitian / nutritionist
- ☐ Scientific articles / academic sources
- ☐ Mobile applications dedicated to health
- ☐ Television / radio
- ☐ Social networks (Facebook, Instagram, TikTok, YouTube)
- ☐ Magazines and newspapers
- ☐ General internet websites
- ☐ Family and friends

22. F2. How great is your level of trust in the information received from each of the following sources?

Mark only one oval per row.

Source	Very low trust	Low trust	Neutral	High trust	Very high trust
Family doctor	☐	☐	☐	☐	☐
Dietitian / nutritionist	☐	☐	☐	☐	☐
Internet websites	☐	☐	☐	☐	☐
Social networks	☐	☐	☐	☐	☐
Television / radio	☐	☐	☐	☐	☐
Family and friends	☐	☐	☐	☐	☐
Scientific articles / academic sources	☐	☐	☐	☐	☐

SECTION G. Awareness of European and national policies and tools

23. G1. Have you heard of the European Code Against Cancer? *

Mark only one oval.

- ☐ Yes
- ☐ No

24. G2. Have you heard of the Nutri-Score nutritional labeling system? *

Mark only one oval.

- ☐ Yes
- ☐ No

25. G3. Are you familiar with the National Plan for the Prevention and Control of Cancer in Romania (2023–2030)?

Mark only one oval.

☐ Yes

☐ No

26. G4. Have you ever received advice on nutrition for cancer prevention from a healthcare professional?

Mark only one oval.

☐ Yes

☐ No
